# High-throughput proteomics reveal alarmins as amplifiers of tissue pathology and inflammation after spinal cord injury

**DOI:** 10.1038/srep21607

**Published:** 2016-02-22

**Authors:** Athanasios Didangelos, Michele Puglia, Michaela Iberl, Candela Sanchez-Bellot, Bernd Roschitzki, Elizabeth J. Bradbury

**Affiliations:** 1 Wolfson Center for Age Related Diseases, Guys Campus, King’s College London, United Kingdom; 2 Functional Genomics Center Zurich (FGCZ), ETH Zurich, University of Zurich, Switzerland

## Abstract

Spinal cord injury is characterized by acute cellular and axonal damage followed by aggressive inflammation and pathological tissue remodelling. The biological mediators underlying these processes are still largely unknown. Here we apply an innovative proteomics approach targeting the enriched extracellular proteome after spinal cord injury for the first time. Proteomics revealed multiple matrix proteins not previously associated with injured spinal tissue, including small proteoglycans involved in cell-matrix adhesion and collagen fibrillogenesis. Network analysis of transcriptomics and proteomics datasets uncovered persistent overexpression of extracellular alarmins that can trigger inflammation via pattern recognition receptors. In mechanistic experiments, inhibition of toll-like receptor-4 (TLR4) and the receptor for advanced glycation end-products (RAGE) revealed the involvement of alarmins in inflammatory gene expression, which was found to be dominated by IL1 and NFκΒ signalling. Extracellular high-mobility group box-1 (HMGB1) was identified as the likely endogenous regulator of IL1 expression after injury. These data reveal a novel tissue remodelling signature and identify endogenous alarmins as amplifiers of the inflammatory response that promotes tissue pathology and impedes neuronal repair after spinal cord injury.

At the molecular level spinal cord injury (SCI) is characterized by an aggressive inflammatory reaction^1^, neuronal degeneration and dynamic changes in the structure and composition of the extracellular matrix (ECM) leading to gliosis^2^. After SCI there is little functional repair. Tissue damage is so extensive that areas of white and grey matter are eventually replaced by cystic cavities edged by a fibrotic glial scar. Together with degenerating myelin-derived molecules^3^, the ECM is a potent inhibitor of axonal growth and blocks neuronal regeneration and growth through the injury epicentre. In particular, the accumulation of large chondroitin sulphate proteoglycans (CSPGs) in the glial scar inhibits regeneration and limits functional repair^4^, while removal of their sugar component (glycosaminoglycan; GAG chains) using chondroitinase ABC promotes plasticity^5^,^6^. Interestingly, excluding large CSPGs (aggrecan, brevican, neurocan, versican, phosphacan) and type IV collagen^7^,^8^, little is known about the composition and remodelling of the ECM after SCI, particularly with regards to the insoluble, collagen-rich fibrillar matrix that forms in chronic lesions^9^. Apart from its multivariate structural role, the matrix serves as a binding substrate and repository to a myriad of soluble extracellular factors with important bioactivity and different studies by us and others suggest that matrix remodelling after tissue injury might influence the immune response and tissue pathology^10^,^11^,^12^,^13^,^14^,^15^.

Here we adapted a recently developed high-throughput proteomics approach^16^,^17^ in order to target and characterize for the first time the composition of the extracellular matrix in the spinal cord and identify novel mediators of pathological tissue remodelling following spinal cord injury. Analysis of matrix molecules by proteomics offers an advantage over measuring transient mRNA expression, given that matrix proteins tend to accumulate over-time after injury and tissue remodelling, they are subjected to post-synthesis proteolytic processing and modification and have a longer half-life in comparison to cellular proteins. Using this proteomics approach we mapped global pathological changes in the injured spinal cord, identified and validated previously unknown extracellular matrix proteins in chronic spinal lesions and revealed biological targets involved in endogenous inflammatory regulation. These findings have important implications for understanding pathological mechanisms after SCI and could lead to the identification of novel targets for repair.

## Results

### Enriching for ECM proteins

**Figure 1:**
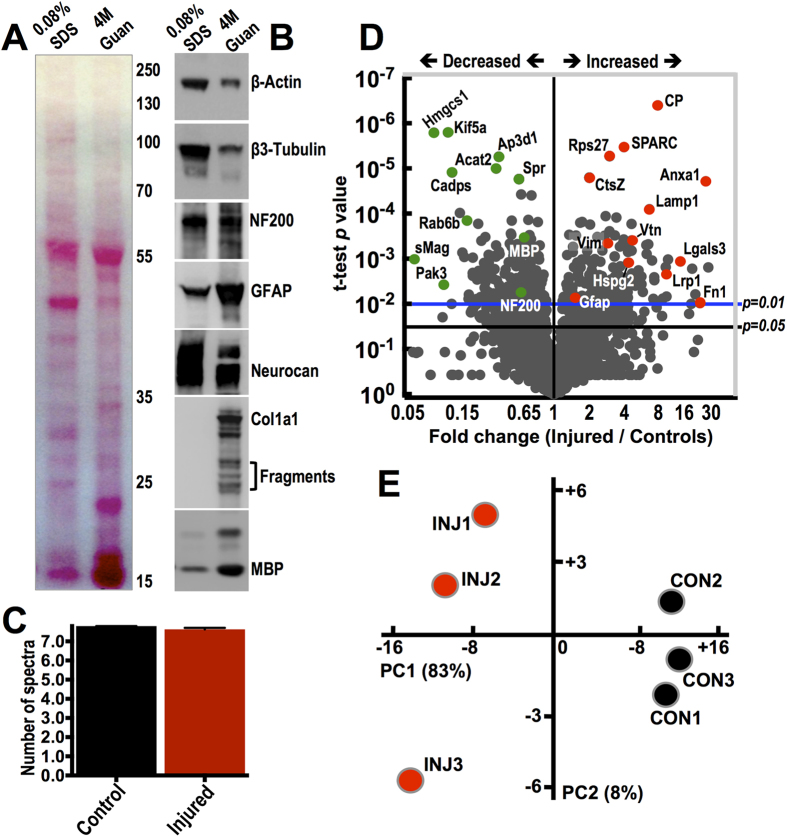
Proteomics analysis of ECM-enriched protein fraction of injured spinal tissue. (**A,B**) Protein composition in 0.08% SDS and 4 M guanidine extracts derived from T10 injury epicentre spinal cord explants, 8 weeks post-contusion. Ponceau staining (**A**) shows a clear difference in the protein content of the 2 extracts. 15 μg of protein was loaded in both lanes. Immunoblotting (**B**) depicts the relative abundance of selected proteins in SDS and guanidine extracts. (**C**) Average number of normalized spectral counts measured in 4 M guanidine extracts of 3 uninjured control and 3 injured T10 spinal cord specimens by shot-gun LC-MS/MS with a total of 2346 proteins identified, 10 ppM peptide mass accuracy tolerance, 1% false discovery rate and 95% peptide and protein identification probability. (**D**) Volcano plot shows differential protein expression measured by spectral counting in 3 uninjured control and 3 injured T10 spinal cord extracts. Proteins are separated according to their spectral count fold-change (Injured/Control; x-axis) and their two-tailed t-test *P* value (y-axis). *P* *=* *0.05* and *P* *=* *0.01* are indicated. Selected highly dysregulated proteins are highlighted (upregulated: red; downregulated: green). (**E**) Principal component analysis (PCA) clustering control and injured T10 spinal cord extracts based on spectral counting of the 2346 LC-MS/MS protein identifications.

To improve extraction, solubilization and enrichment of the matrix from established SCI lesions prior to proteomics, we adapted a proteomics-based methodology previously used in cardiovascular tissues^16^,^17^. Here, we use this approach for the first time to study the rat spinal cord extracellular proteome. The method is based on mild decellularization of spinal tissues to reduce their cellular content, prior to extracting the more insoluble matrix. Injury epicentre (T10) or uninjured control T10 spinal cord tissue was first incubated in low concentration SDS to solubilize cellular membranes and extract intracellular contents while preserving insoluble ECM proteins. The decellularized spinal cord explants were then incubated in a strongly denaturing 4 M guanidine buffer to extract the insoluble fraction of extracellular proteins remaining in the tissue. Fig. 1A compares the protein composition of SDS and guanidine sequential extracts derived from the epicentre of contused spinal cords, 8 weeks post-injury. Protein staining of the western-blotted membrane reveals a clear difference in protein content, although protein concentration is the same in both lanes (15 μg). Immunoblotting shows differential enrichment of proteins in these extracts (Fig. 1B). Cytoplasmic β-Actin and neuronal β3-Tubulin were enriched in SDS. Neurofilament 200 (NF200) did not show an obvious preference, while the astrocytic intermediate filament glial fibrillary acidic protein (GFAP) was enriched in guanidine extracts, likely reflecting the presence of astrocytic processes and membrane extensions projecting in the extracellular space and forming the glial scar^18^. Neurocan, a classic spinal cord injury proteoglycan, was immunoreactive in SDS and guanidine extracts, indicating that the proteoglycan exists in both easily soluble and insoluble populations 8 weeks post-injury. Unlike proteoglycans found in connective tissues (cartilage, skin, etc), large CSPGs of the healthy CNS exist predominantly in easily extractable pools or are membrane bound, pericellular or in microsome structures^19^. After injury, a less soluble collagen-associated pool is likely to develop, especially in mature lesions. Accordingly, the prototypical fibrillar collagen type I (Col1a1), was only extracted in 4 M guanidine (Fig. 1B) indicating the relative insolubility of the fibrotic matrix. Although type I collagen is the main culprit of tissue fibrosis in most organ pathologies, its expression and function in spinal cord lesions is only recently being studied^9^,^20^,^21^. Finally, myelin basic protein (MBP) was also enriched in the guanidine fraction (Fig. 1B), indicating the insolubility of axonal myelin sheath structures.

### Proteomics analysis of matrix-enriched spinal cord extracts

Next, matrix-enriched guanidine extracts from injured (8 weeks post-contusion; 150 kDyne) and uninjured control T10 spinal cord segments, were analyzed using shot-gun liquid chromatography tandem mass spectrometry (LC-MS/MS) on a high accuracy mass-spectrometer (Q-Exactive, Thermo). 2346 proteins were identified in total (10 ppM peptide mass accuracy tolerance and 1% false discovery rate; Supplementary Table S1) with a comparable average number of normalized spectra for both groups (Fig. 1C) at 2 μg of peptide loaded on the liquid chromatography column. Differential protein expression was estimated using normalized spectral counts (Supplementary Table S1). The volcano plot (Fig. 1D) highlights a number of differentially regulated proteins (t-test *P* value cut-off 0.05 & 0.01 are indicated). The accumulation of the acute phase reactant ceruloplasmin (CP, Fig. 1D) in 8-week old spinal cord lesions is notable. CP oxidizes ferrous iron after hemorrhage and has been shown to have an important role in SCI^22^. The glycoproteins SPARC (osteonectin), galectin-3 (Lgals3), vitronectin (Vtn), perlecan (Hspg2) and fibronectin (Fn1) were the most upregulated extracellular proteins (red dots; Fig. 1D). They are all involved in cell-to-matrix adhesion and interaction. On the other hand, downregulated proteins (green dots; Fig. 1D) include: Kif5a (kinesin family member 5a) a microtubule-dependent motor required for axonal transport of neurofilament proteins^23^, Cadps (calcium-dependent secretion activator) a neuronal membrane protein required for calcium-regulated exocytosis of neuropeptide vesicles^24^ and Ap3d1 (adaptor-related protein complex 3), involved in trafficking of neurotransmitter vesicles^25^. Proteomics also revealed characteristic and expected changes of injured spinal tissue including: upregulation of GFAP and vimentin (Vim) (red dots; Fig. 1D) and downregulation of myelin-associated glycoprotein (sMag; small isoform), MBP and NF200 (green dots; Fig. 1D). Principal component analysis (Fig. 1E) on the entire protein dataset (All 2346 identified proteins) shows a clear separation of injured and intact tissue protein profiles. Control samples are more homogeneous than injured samples.

### Network analysis of protein changes

**Figure 2:**
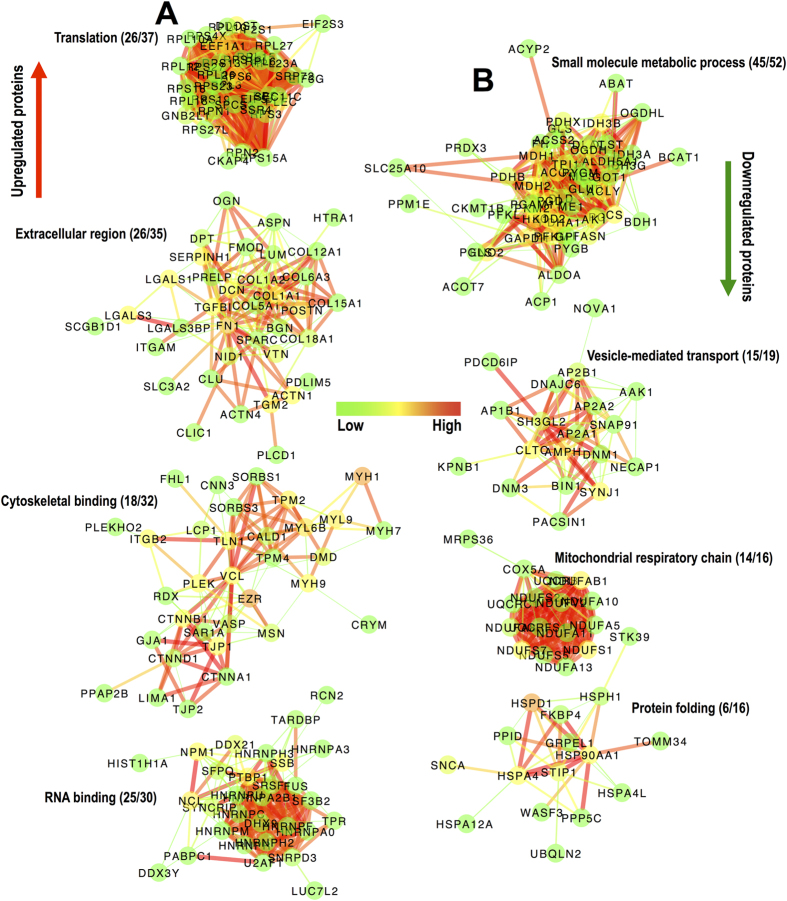
Network analysis of differentially regulated proteins in chronic SCI tissue. (**A,B**) The 8 most populated subnetworks generated by MCL clustering (1.9 inflation value & 0.4 edge weight cut-off; StringDB, v9.1) of upregulated (**A**) and downregulated (**B**) protein networks shown in Supplementary Figure S1. Individual subnetworks were analysed by BiNGO to identify the predominant gene ontology term. Numbers in parenthesis indicate the number of proteins in the predominant gene ontology versus the total number of proteins in each subnetwork. Node color indicates betweeness centrality while edge color indicates interaction score based on the predicted functional links between nodes (green: low values; red: high values).

Network analysis^26^ of upregulated and downregulated proteins (proteins with t-test *P ≤ 0.05* were used) shows tight clustering of identified proteins (Supplementary Figure S1). Based on their network properties (connectivity of nodes), proteins were then clustered using an automated Markov clustering method (MCL^27^; 1.9 inflation value) to generate smaller subnetworks of upregulated (Fig. 2A) and downregulated (Fig. 2B) proteins. Finally, the dominant gene ontology of each MCL-generated subnetwork was determined using BiNGO to identify biologically relevant functional clusters in the proteomics dataset. Fig. 2 shows the 8 most populated protein clusters and highlights their main gene ontology. In the group of upregulated proteins (Fig. 2A) “translation” is the most populated cluster with 26 ribosomal proteins, followed by “extracellular region” containing mainly ECM and matrix-associated proteins, due to extensive matrix remodelling after injury, “cytoskeletal binding”, reflecting cellular reorganization in the injury epicentre and binding to the ECM and “RNA binding”, presumably related to increased translation. In the group of downregulated proteins (Fig. 2B), “small molecule metabolic process” is the most overrepresented category, indicating metabolic paucity in the injured tissue, followed by “vesicle-mediated transport” due to lack of axonal function, “mitochondrial respiratory chain” indicating loss of mitochondria and metabolic depression and finally, “protein folding” suggesting defective protein synthesis.

### Identification of extracellular matrix proteins

**Figure 3:**
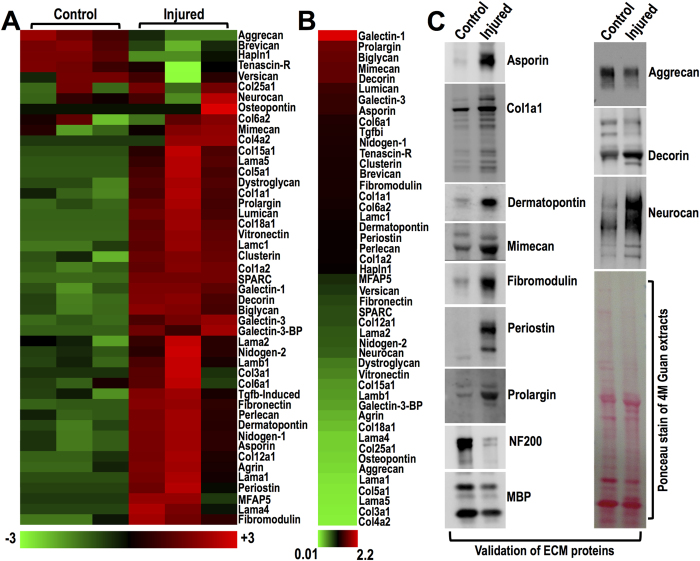
Extracellular matrix proteins identified by proteomics and validated by immunoblotting. (**A**) Differential expression of 47 typical extracellular matrix proteins identified by LC-MS/MS in uninjured control and injured T10 spinal cords. (**B**) Relative matrix protein abundance in the guanidine extracts was calculated as the ratio of protein spectral counts divided by protein molecular mass in kDa. Galectin-1 had the highest spectral count/molecular mass ratio (2.2) while collagen alpha-2(IV) (Col4a2) had the lowest ratio (0.01). (**C**) Different matrix proteins were validated by western-blotting in 4 M guanidine extracts of uninjured control and injured T10 spinal cord specimens, 8 weeks post-contusion. One representative example is shown. Ponceau stain demonstrates comparable loading but distinct protein composition in control versus injured extracts.

To characterize the composition of the pathological insoluble matrix accumulating in spinal lesions, we next focused on ECM proteins. Proteomics identified 47 classic matrix proteins, collagens, glycoproteins, proteoglycans and laminins (Fig. 3A). ECM spectral counts are shown in Supplementary Table S2. Most ECM proteins were upregulated 8 weeks post-injury (Fig. 3A & Supplementary Table S2). Notably, multiple matrix proteins are identified in injured spinal tissue for the first time and their function is unknown. These include the small glycoproteins biglycan, dermatopontin, asporin, fibromodulin, mimecan, prolargin, MFAP5 (microfibrillar associated protein 5) and collagens Col3a1, Col5a1, Col15a1 and Col25a1 amongst others. Apart from measuring differential expression of matrix proteins after SCI, proteomics can also determine their relative abundance in the spinal cord by dividing the number of identified protein spectra of each protein by its molecular mass in kDa (Fig. 3B & Supplementary Table S2). The glycoprotein galectin-1 had the highest ratio of identified spectra to molecular mass, followed by the keratan sulphate proteoglycans prolargin and mimecan (Fig. 3B & Supplementary Table S2). Other small proteoglycans, including the dermatan sulphate decorin, chondroitin sulphate biglycan and keratan sulphate lumican also had high ratios. Basement membrane collagen 6 chain alpha1 (Col6a1) was the highest collagen, followed by the classic component of fibrosis collagen 1 chain alpha1 (Col1a1) (Fig. 3B & Supplementary Table S2). Next, we used western-blotting to validate the identification and upregulation of unusual glycoproteins (asporin, dermatopontin, mimecan, fibromodulin, periostin and prolargin) in a further set of injured animals at 8 weeks post-injury and found an increase in injured extracts compared to controls (Fig. 3C). The increase in Col1a1 was also validated, while NF200 and MBP were decreased as expected at 8 weeks post-injury (Fig. 3C). From the large CSPGs, the decrease in aggrecan and increase in neurocan were confirmed by western blotting (Fig. 3C) in agreement with previous studies^28^. The signature of this relatively insoluble matrix found in guanidine extracts, points towards extensive tissue remodelling and reveals a large number of previously unknown ECM proteins in injured spinal tissue.

### Identification of persistently overexpressed bioactive mediators

**Figure 4:**
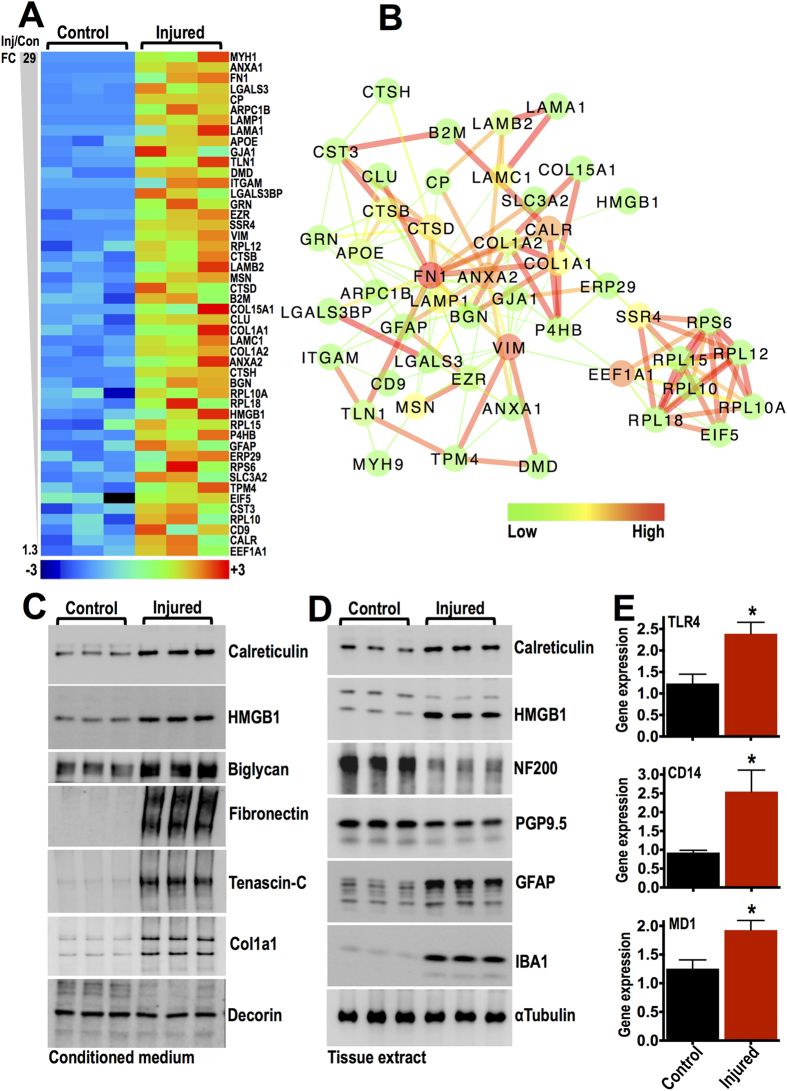
Identification of persistently upregulated bioactive molecules. (**A**) Heat-map displays 48 upregulated proteins found in proteomics analysis of guanidine extracts 8 weeks post-injury. Proteins are listed on the heat-map according to their fold change (FC, Injured/Controls). These molecules were also upregulated at the transcript level 5 weeks post-injury as detected by microarray analysis (see reference 29). (**B**) Network analysis (StringDB, v9.1) of the 48 genes upregulated both at the mRNA and protein level. Node color indicates betweeness centrality while edge color indicates interaction score based on the predicted functional links between nodes (green: low values; red: high values). (**C,D**) Immunoblotting of conditioned medium (**C**) and tissue extracts (**D**) derived from either uninjured control or injured T10 spinal cord explants cultured for 24 hours in plain DMEM culture medium. The endogenous TLR4 ligands HMGB1, biglycan, fibronectin (EDA fragments) and tenascin-C are present in the conditioned medium. Calreticulin and HMGB1 were also upregulated in tissue extracts. (**E**) Relative mRNA expression of TLR4, CD14 and MD1 measured by TaqMan quantitative PCR in either control or injured T10 spinal cord explants, 7 days post-injury. ACTB served as the housekeeping gene. *N* = 3 animals per group; * *P* *≤* *0.05*, two-tailed t-test.

In order to identify molecules with persistent expression at the mRNA and protein levels, we performed an unbiased, large-scale comparison of all differentially regulated transcripts detected 5 weeks after spinal cord injury in a published microarray dataset of rat T8 spinal contusion (E-GEOD-2599, ArrayExpress, EBI; performed by Aimone and colleagues^29^) with all differentially regulated proteins identified in our proteomics analysis 8 weeks after rat T10 spinal contusion. The temporal difference between transcriptomics and proteomics was selected to capture mRNA to protein synthesis. 48 entities were upregulated both at the transcript and protein level after spinal contusion (Fig. 4A, Supplementary Table S3A: transcripts & Supplementary Table S3B: proteins). The interaction network of the 48 upregulated proteins (Fig. 4B) was enriched in extracellular proteins, including various collagens (COL), fibronectin (FN1), biglycan (BGN), laminins (LAMA/B), galectins (LGALS) and others, clustering with typically cellular proteins that can be released into the extracellular space such as calreticulin (CALR), high-mobility group box 1 (HMGB1) and endoplasmic reticulum protein 29 (ERP29), amongst others. MCL clustering and network gene ontology annotations of molecules dysregulated both at the transcript and protein level can be seen in Supplementary Figure S2 and S3.

Interestingly, fibronectin (EDA fragments), biglycan, HMGB1 and calreticulin have been involved in the induction of sterile inflammation after tissue injury and they belong to a group of molecules collectively known as alarmins or danger-associated molecular patterns (DAMPs)^30^. Biglycan, fibronectin and HMGB1 are known ligands of the classic innate immunity receptor TLR4^31^, the best-characterized pattern recognition receptor, while extracellular calreticulin is mainly recognized by the low-density lipoprotein receptor-related protein (Lrp1)^32^, which was also upregulated in our proteomics dataset (Fig. 1D). Although TLR4 was not identified by proteomics, its mRNA was upregulated in the transcriptomics dataset, together with its coreceptors CD14 and MD1 (Supplementary Table S3A; from E-GEOD-2599^29^, ArrayExpress EBI).

### Alarmins in early tissue remodeling

Next we wanted to examine whether this inflammatory signature was upregulated early after spinal cord injury and whether the identified alarmins could be released or secreted from the injured tissue during early pathological remodeling (1 week). Bioactive molecules involved in subacute inflammatory events could be useful targets for early therapeutic interventions in the future. Moreover, proteins released into the extracellular environment shortly after injury could be trapped in the matrix during post-injury tissue remodeling and subsequently become part of the extracellular milieu in chronic lesions. Extracellular alarmins (biglycan, fibronectin) as well as the typically intracellular calreticulin and HMGB1, were released in the conditioned medium of cultured T10 spinal cord explants and were clearly increased in the supernatants of injured explants, 1 week after spinal contusion (Fig. 4C). Tenascin, a matrix glycoprotein upregulated after tissue injury and a known TLR4 ligand^12^, was also found in the conditioned medium of 1 week injured explants (Fig. 4C). Tenascin was surprisingly not detected in our proteomics analysis, but its mRNA was upregulated after injury (Supplementary Table S3A; from E-GEOD-2599^29^, ArrayExpress EBI). Comparatively, Col1a1 was also increased in the injured spinal cord supernatants, while the dermatan sulphate proteoglycan decorin (similar to biglycan) was unchanged, indicating selective release of matrix molecules at this early time point (Fig. 4C). Calreticulin and HMGB1 were also increased in tissue extracts of injured spinal cord explants (Fig. 4D), confirming that they are not just passively released but upregulated *de novo* at 1 week post contusion. In contrast, neuronal markers NF200 and PGP9.5 were decreased in injured spinal cords, while GFAP (astrocytes) and IBA1 (microglia/macrophages) were upregulated as expected (Fig. 4D). Finally, we performed qPCR analysis of intact and 1 week injured explants and found that TLR4, CD14 and MD1 mRNA expression was increased after spinal cord injury (Fig. 4E). This data indicates that tissue-derived alarmins play a role in subacute inflammatory events after spinal cord injury.

### Inflammatory activation by soluble alarmins

**Figure 5:**
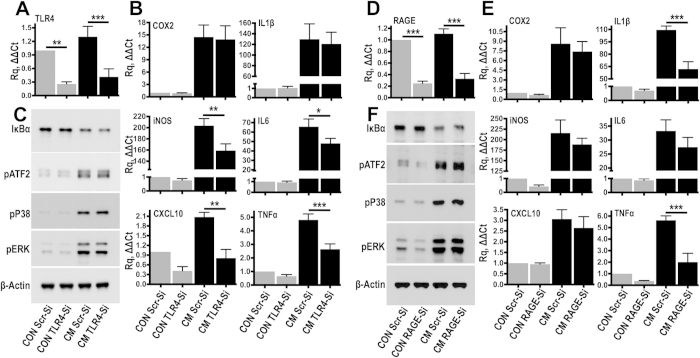
TLR4 and RAGE siRNA knockdown partially suppress inflammatory gene expression. (**A,B**) TLR4 expression was blocked using siRNA in primary fibroblasts (**A**). For comparison, control cells were transfected with target-less scrambled (Scr) siRNA. 48 hours later, Scr or TLR4-transfected fibroblasts were stimulated for 3 hours with conditioned medium (CM) sampled from injured spinal cord explants or kept in plain culture medium (CON). Gene expression of inflammatory genes was measured by TaqMan qPCR (**B**). ACTB served as the housekeeping gene. *N* = 6 independent experiments; *P *≤* 0.05, **P *≤* 0.01, ***P *≤* *0.001*; Anova with Fisher’s LSD multiple comparison test. (**C**) To examine acute signalling activation, scrambled or TLR4 siRNA transfected cells were stimulated with conditioned medium for 25 minutes. Signalling activation was examined by immunoblotting. *N* = 3 independent experiments. (**D,E**) As for TLR4, RAGE expression was blocked using siRNA (**D**) and 48 hours later Scr or RAGE-transfected fibroblasts were stimulated with conditioned medium (CM) and gene expression was measured by TaqMan qPCR (**E**). ACTB served as the housekeeping gene. *N* = 6 independent experiments. ***P *≤* *0.001*; Anova with Fisher’s LSD multiple comparison test. (**F**) Acute signalling activation of RAGE-transfected cells was examined by immunoblotting. *N* = 3 independent experiments.

To determine the role of TLR4 in the recognition of soluble endogenous ligands and inflammatory activation, we silenced the receptor on primary rat fibroblasts using small-interfering RNA (siRNA) (Fig. 5A). Fibroblasts have sentinel immunoregulatory properties and alongside astrocytes and macrophages, play a key role in tissue scarring following spinal cord injury. siRNA-transfected cells were subsequently stimulated with conditioned medium sampled from 1 week injured spinal cord explants, which contains the soluble TLR4 ligands (Fig. 4C). TLR4-siRNA achieved a ~70% reduction in TLR4 mRNA in comparison to fibroblasts transfected with scrambled siRNA (Fig. 5A). Short stimulation (3 hours) of resting cells with injury conditioned medium induced the expression of classic inflammatory markers (COX2, iNOS, IL1β, IL6, TNFα and CXCL10 Fig. 5B). TLR4-siRNA suppressed the expression of iNOS (−23%), IL6 (−28%), TNFα (−45%) and CXCL10 (−62%), while COX2 and IL1β were unaffected (Fig. 5B). This data indicated that soluble factors in the conditioned medium could stimulate inflammatory gene expression and that TLR4 is at least partially involved. TLR4 ligation typically induces MAP kinase and NFκB activation^33^. Stimulation of resting cells with injury conditioned medium (Fig. 5C) resulted in robust phosphorylation of p38, ERK, ATF2 (an AP1 transcription factor downstream of JNK) and degradation of IκBα (activation of NFκΒ), but signalling was unaffected in TLR4 siRNA knock-out cells (Fig. 5C), indicating the involvement of alternative receptors in acute signaling activation.

One of the soluble alarmins upregulated after injury is HMGB1 (Fig. 4A,C,D). HMGB1 is a well-characterized danger signal^34^ that as well as TLR4, can also activate the receptor for advanced glycation end products (RAGE). Binding of HMGB1 to this pattern recognition receptor causes classic inflammatory activation^35^. siRNA inhibition of RAGE (Fig. 5D) in cultured cells suppressed the expression of IL1β (−44%) and TNFα (−67%) following stimulation with 1 week injury conditioned medium (Fig. 5E) but did not affect the other inflammatory markers tested (Fig. 5E) or signalling activation (Fig. 5F). The effect of RAGE knock-down on IL1β and TNFα (the latter downregulated by TLR4-siRNA; Fig. 5B) is interesting given the importance of these cytokines on inflammation.

### Dominant proinflammatory role of the IL1 receptor

**Figure 6:**
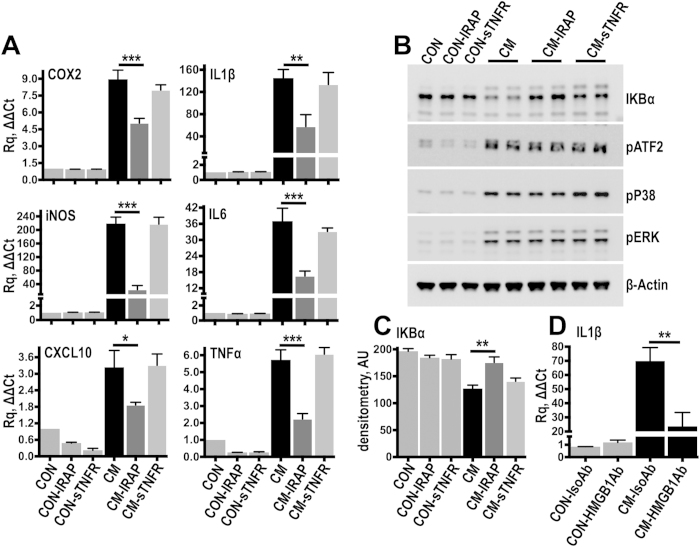
Interleukin 1 is the dominant inflammatory factor in the injury conditioned medium. (**A**) Resting fibroblasts were stimulated for 3 hours with injury conditioned medium (CM) supplemented with 20 ng/ml of either IRAP or sTNFR. Control cells were kept in plain culture medium (CON) and additional controls were incubated with IRAP (CON-IRAP) or sTNFR (CON-sTNFR). Gene expression was measured by TaqMan qPCR. ACTB served as the housekeeping gene. *N* = 4 independent experiments. (**B,C**) To examine acute signalling activation cells were stimulated for 25 minutes with conditioned medium supplemented either with IRAP or sTNFR and signalling activation was examined by immunoblotting (**B**). IκΒα levels were quantified by densitometry (**C**) *N* = 3 independent experiments. (**D**) Resting cells were stimulated with injury conditioned medium which was previously treated for 2 hours with HMGB1 antibodies (HMGB1Ab; 5 μg/ml) to neutralize soluble extracellular HMGB1, or with isotype control antibodies (IsoAb; 5 μg/ml). IL1β expression was measured by TaqMan qPCR. *N* = 6 independent experiments. *P *≤* 0.05, **P *≤* 0.01, ***P *≤* *0.001*; Anova with Fisher’s LSD multiple comparison test.

The partial effect of TLR4 and RAGE knockdown on inflammatory gene expression and the lack of signaling inhibition, prompted us to test alternative activation mechanisms. To this end, we targeted the main proinflammatory cytokines IL1β and TNFα using either IL1 receptor (IL1R) antagonist protein (IRAP) or soluble TNF receptor II (sTNFR) respectively (Fig. 6A). Blocking IL1R with IRAP in cells stimulated with 1 week injury conditioned medium, resulted in a clear inhibition of inflammatory gene expression (Fig. 6A). Moreover, IRAP prevented IκBα degradation (Fig. 6B,C), suggesting inhibition of NFκB activation; MAP kinase phosphorylation was unaffected (Fig. 6B), presumably because the MAP kinases are broadly activated by various molecules including cytokines, growth factors and other stimulating input^36^. This data indicates the dominant involvement of IL1 in the regulation of inflammatory gene expression in this system. TNFRII blockade did not affect gene expression or signalling activation, albeit a small and not significant effect on IκBα degradation (Fig. 6B,C). The involvement of NFκB on inflammatory gene expression was confirmed using TPCA-1^37^, a biochemical inhibitor with high specificity for IκB kinase 2 (IKK2), the main activator of the canonical NFκB pathway^38^. Incubation of cells with TPCA-1 prior to stimulation with injury conditioned medium, led to a substantial reduction of inflammatory transcripts comparable to IL1 receptor blockade (Supplementary Figure S4).

Notably, incubation of injury conditioned medium with neutralizing antibodies against HMGB1 resulted in a substantial inhibition of IL1β mRNA induction (Fig. 6D). Neutralization of the alarmin did not affect COX2, iNOS, CXCL10, IL6 or TNFα expression (Supplementary Figure S5) suggesting a specific effect of soluble HMGB1 on IL1. Taken together, this data suggests that endogenous pattern recognition of danger signals is a complimentary mechanism of inflammatory regulation, which appears to be dominated by IL1R and NFκB activation.

## Discussion

Here we used an innovative methodology to characterize the insoluble extracellular protein fraction in chronic spinal cord lesions using proteomics. Our approach enabled the identification of numerous matrix proteins with unknown function in the injured spinal cord and the characterization of a unique pathological protein signature enriched in extracellular inflammatory mediators.

The biochemical enrichment of insoluble proteins in our extraction method, as well as the suitability of proteomics to detect accumulating matrix proteins, enabled the identification of previously unknown extracellular proteins in spinal cord lesions. We identified molecules associated with fibrotic tissue remodelling and numerous small proteoglycans including the chondroitin sulphate biglycan (TLR4 activator with unknown function in SCI), keratan sulphate family members mimecan and lumican (both found in the cornea) together with prolargin and fibromodulin (abundant in articular cartilage). Although keratan sulphate is known to be upregulated after SCI, the identity and function of core proteins remains elusive with the exception of lumican^39^. Our identifications also included two atypical proteoglycans, asporin and dermatopontin. Their function in SCI is unknown but in other tissues they are both involved in collagen matrix formation and modulation of TGFβ activity, the master regulator of fibrosis. Other ECM glycoproteins with important functionality include osteonectin (SPARC), periostin, perlecan (HSPG2) and galectins 1 and 3. SPARC induces neurite outgrowth by stimulating Schwann cells^40^ and similarly, astrocyte-derived periostin has been shown to promote axonal regeneration^41^. Perlecan is a large heparan sulfate proteoglycan with widespread expression in the basement membrane of different tissues, but its role after spinal cord injury is unknown^42^. The small glycoproteins galectin-1 and −3 have broad functions in many tissues. In SCI their function is currently unclear; galectin-1 is thought to be neuroprotective^43^ and to modulate macrophage behavior in the injury epicentre^44^, while galectin-3 is likely associated with secondary inflammatory events^45^.

Functionally, small proteoglycans and glycoproteins are involved in adhesion of cells in tissues (fibronectin, vitronectin, decorin, biglycan, periostin and lumican) as well as collagen fibril assembly (fibronectin, decorin, biglycan, fibromodulin, dermatopontin and lumican). Increased cellular adhesion is expected in the injured tissue, while the substantial collagen identifications, together with collagen-associated molecules, point towards a fibrosis phenotype in the contused spinal cord and is in agreement with recent work describing the pathological relevance of type I collagen^9^,^21^. Importantly, the guanidine extracts were also enriched in proteins present in cellular organelles (endoplasmic reticulum-ribosomes, mitochondria and lysosomes), hence naturally harder to extract in mildly dissociative conditions (0.08% SDS). Evidently, the decellularization is partial and complete clearance of cellular contents with mild extractants would be difficult to achieve without solubilizing matrix elements, especially in a compressible, matrix-poor tissue like the spinal cord. On the other hand, the decellularization allowed proteomics interrogation of the scarce interstitial matrix and offered insights on the differential regulation of subcellular structures.

An important promise of high-throughput methodologies is to identify novel bioactive mediators and gain mechanistic insights into complex pathological processes. To this end, we performed a comparative analysis of our proteomics findings with a previously published gene expression microarray dataset^29^, in order to identify biological targets that are dysregulated after spinal cord injury both at the transcript and protein levels and which show persistent differential expression in the injury epicentre. Both studies used a moderate severity thoracic contusion in adult female rats. The Aimone study^29^ assessed mRNA changes at 5 weeks post-injury while we examined protein changes 8 weeks post-injury. This comparison identified 48 genes that were upregulated both at the mRNA and protein level after spinal contusion. The shared molecular signature contained a mix of classic extracellular proteins together with cellular proteins that can be released or secreted into the extracellular space. Importantly, although this comparative analysis utilised high-throughput transcriptomics and proteomics data generated in two independent labs with different technical setups, it highlights that persistent dysregulation of proteins involved in extracellular tissue remodelling is a key pathological process after spinal cord injury. These shared upregulated genes might represent important biological targets in spinal cord lesions.

Upon closer inspection of the common transcriptomics and proteomics signature, we also noted the upregulation of the endogenous TLR4 ligands (alarmins) biglycan, fibronectin, tenascin-C and HMGB1. Next, we wanted to investigate whether these alarmins have an inflammatory effect in subacute lesions (1 week post-injury). At this early stage of inflammation and tissue remodelling, therapeutic interventions targeting inflammatory ligands or receptors could be effective in terms of tissue sparing and reducing post-injury inflammatory pathology. Indeed, these alarmins were released from spinal cord explants cultured 1 week after injury, while TLR4 was upregulated at the mRNA level. TLR4 activation by endogenous ligands has been explored in other inflammation and tissue injury paradigms. Biglycan is known to activate TLR4 (and TLR2) in kidney injury^46^ and sepsis^14^. TLR4 activation by fibronectin (EDA) fragments^47^ is proinflammatory in stroke and myocardial infarction^48^ while tenascin is a major TLR4 ligand in rheumatoid arthritis^12^. HMGB1 is a ubiquitous nuclear protein that can be released after acute cell stress, injury or necrosis and acts as a TLR4 danger signal in the extracellular milieu, activating a classic inflammatory response^49^. Recently, HMGB1 release from injured cortical neurons has been linked to microglial activation in the post-ischemic brain^50^. The role of TLR4 and its endogenous ligands in spinal cord injury is not well understood^51^. One key study by Kigerl *et al.*, showed that inactivation of TLR4 in mice caused increased astrogliosis and lesion pathology although it suppressed cytokine mRNA at the lesion site^52^. Importantly, these TLR4 mutant animals (CH3/HeJ) have a global defective TLR4 sensing and this could explain the paradoxical results obtained by affecting the key innate immunity receptor. Nevertheless, given the robust involvement of TLR4 in neuroinflammation^53^ and in an array of different inflammatory settings, more work is needed to clarify its role in SCI, especially with regards to endogenous pattern recognition of ligands generated during tissue remodelling.

In proof-of-concept experiments, downregulation of TLR4 by siRNA, suppressed the expression of classic inflammatory mediators (iNOS, IL6, TNFα and CXCL10) following stimulation of resting fibroblasts with injury conditioned medium derived from spinal cord lesions 1 week post-injury, but failed to alter acute signalling and did not affect COX2 and IL1β expression. The partial effect of TLR4 inhibition demonstrates the complexity of inflammatory activation after tissue injury, involving a multitude of different ligands and receptors. For instance, HMGB1, perhaps the best-characterized alarmin found in the injury conditioned medium, is also known to activate RAGE (another pattern recognition receptor) and has been recently suspected to contribute to spinal cord injury inflammation^54^. When we silenced RAGE, IL1β and TNFα transcripts were decreased. The reduction in IL1β mRNA and the shared (TLR4 and RAGE) inhibitory effect on TNFα led us to investigate the role of these central cytokines. Pharmacological blockade of the IL1 receptor, but not the TNF receptor, had a clear inhibitory effect on all inflammatory transcripts examined *in vitro*. This data is in agreement with recent work demonstrating the importance of IL1 in SCI inflammation^55^. IL1 receptor blockade also suppressed NFκB activation, perhaps the most critical regulator of inflammatory gene expression in numerous injury and inflammation paradigms^56^. Although activation of NFκB after SCI was first reported more than 15 years ago^57^, surprisingly little is known about its role in inflammatory regulation in the injured spinal cord. Importantly, our *in vitro* experiments were performed in primary rat fibroblasts. Fibroblasts are highly suitable as target cells for inflammatory signalling and gene expression experiments. They are key in wound healing, have well-established sentinel immunoregulatory properties^58^ and are excellent target cells to study innate immunity responses and signalling activation by TLRs^59^,^60^ and other classic inflammatory mediators such as IL1 and TNF^61^. Fibroblasts also show very low baseline expression of inflammatory genes and low expression of signalling intermediaries (in contrast to macrophages) but respond avidly to inflammatory stimuli and are well-known to express all classic inflammatory response genes (IL1, TNF, IL6, iNOS, COX2, etc)^58^ as well as proteins of the complement system. Fibroblasts are also relevant in the context of spinal cord injury and alongside reactive astrocytes (gliosis), play a key role in extracellular remodeling and post-injury fibrosis. While the role of meningeal fibroblasts in spinal cord scarring and lesion formation following penetrating injuries (hemi or transection) is well-established^8^,^13^,^20^,^62^, the function of other fibroblast-type cells is recently gaining attention in relation to post-injury fibrosis in the spinal cord, even after contusion where meninges are largely intact (reviewed in detail in^8^,^63^). For instance, perivascular fibroblasts have been shown to drive classic type 1 collagen (Col1a) lesion fibrosis and scarring after spinal contusion in mice^9^,^21^ while fibroblast-type cells (based on expression and accumulation of fibronectin) have also been reported to infiltrate spinal cord crush lesions where they associate with infiltrating leukocytes^64^,^65^. Finally, pericytes, specialized cells with characteristics of fibroblast lineage, were recently shown as the main contributors of post-injury fibrosis after spinal cord dorsal hemisection^66^.

**Figure 7:**
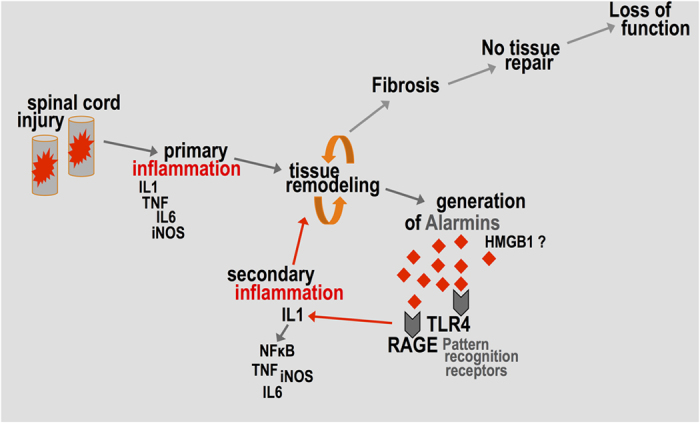
Tissue remodeling and the generation of alarmins after SCI. Schematic summary of the involvement of danger-associated molecular patterns (DAMPs or alarmins) in spinal cord inflammation. After injury, primary inflammation is driven by proinflammatory cytokines and leads to excessive tissue remodelling and fibrosis. Soluble alarmins are generated during pathological tissue remodelling. They activate pattern recognition receptors and contribute to the persistent (secondary) inflammatory activation in the injury epicentre.

Our data suggests that soluble alarmins represent a complimentary mechanism of inflammatory activation, which appears to be dominated by IL1 and NFκB. To this end, neutralization of soluble extracellular HMGB1 inhibited the ability of injury conditioned medium to induce IL1β mRNA expression *in vitro,* indicating the possible role of the alarmin in the regulation of this central cytokine. These findings demonstrate the biological relevance of soluble danger signals; they are released upon injury and pathological tissue remodelling and amplify the inflammatory response via pattern recognition receptors (Fig. 7). We propose that alarmins boost IL1β and TNFα expression, which in turn drive inflammation (Fig. 7). Nevertheless, the clinical applicability of these findings is currently unknown and more work is needed to systematically characterize the potential biomarker, inflammatory and neuronal bioactivity of numerous extracellular targets after spinal cord injury in humans.

In summary, we applied high-throughput proteomics to specifically target the insoluble extracellular proteome following spinal cord injury. Using this approach and unbiased network analysis we have identified a novel pathological signature and soluble bioactive mediators linking tissue remodelling and inflammatory regulation in the injured spinal cord. The involvement of tissue-derived alarmins was predicted and validated *in vitro,* while our findings indicated the importance of the classic IL1-NFκΒ axis in inflammatory activation and identified HMGB1 as a critical endogenous ligand. This work highlights the potential for high-throughput approaches and network analysis to identify novel disease mechanisms and bioactive mediators in spinal cord injury with future drug-targeting potential.

## Methods

### Spinal cord injury model

Anesthetized female adult Sprague-Dawley rats (~200 g; Harlan Laboratories, UK) received a midline 150 kdyne contusion injury at spinal level T10 using an Infinite Horizon impactor device, as previously described in^67^. For proteomics analysis of injured spinal cords, animals were kept for 8 weeks after injury, prior to protein extraction. To generate conditioned medium from injured spinal cords (see below), animals were kept for 1 week post-injury. Control spinal cord tissue was collected from uninjured animals of the same sex, strain and weight. The study has received approval by the institutional Animal Care and Use Committee (King’s College London) and all surgical procedures were performed in accordance with the United Kingdom Animals (Surgical Procedures) Act 1996.

### Protein extraction from spinal cord tissues

Protein extraction from either T10 injury epicentre or uninjured control T10 spinal cord segments (5–6 mm, ~45 mg per explant) was performed in PBS-EDTA perfused female rats. T10 Spinal cord segments were explanted, dissected sagitally and washed 3 times in PBS to reduce contamination of spinal tissue with blood products. To reduce cellular proteins from the tissue (decellularization), spinal cord explants were incubated with 0.08% SDS in double-distilled water, supplemented with a complete proteinase inhibitor cocktail (P8340, Sigma-Aldrich) and 12.5 mM EDTA (metalloproteinase inhibition) for 4 hours at room temperature and mild shaking. Subsequently, 0.08% SDS extracts were collected and SDS-insoluble proteins were extracted by incubating the spinal cord explants in 4 M guanidine suplemmented with proteinase inhibitor cocktail and 12.5 mM EDTA, for 24 hours and vigorous shaking. Proteins were precipitated in 100% ethanol to remove guanidine from lysates and protein pellets were dried before further use either for SDS-PAGE and immunoblotting or shot-gun proteomics (see below). More details about the protein extraction protocol used to enrich relatively insoluble extracellular proteins can be found in^16^,^17^.

### Protein digestion and C18 clean up prior to shot-gun proteomics

Samples were subjected to ultrafiltration for detergent removal, cysteine alkylation and protein digestion^68^. Proteins were digested in 120 μl of 50 mM triethylammonium bicarbonate buffer (pH8.5) using trypsin (Promega) at enzyme to protein ratio of 1:50 Peptide mixtures were desalted using C18 reverse phase cartridges (Fenisterre). Peptides were dried using a vacuum centrifuge and resolubilized in 0.1% formic acid.

### LC-MS/MS analysis and database search

Tryptic peptides were analyzed by reversed-phase liquid chromatography on a Q Exactive mass spectrometer (Thermo) coupled to an Easy-nLC 1000 system (Thermo). Peptides were loaded on a 15 cm-long fused silica frit column (75 μm i.d.; BGB Analytik) and in-house packed with a C18 reverse phase resin (ReproSil-Pur C18-AQ 120A, 1.9μm resin; Dr. Maisch HPLC GmbH, Ammerbuch-Entringen, Germany). The chromatographic separation was performed using an ACN/water solvent system containing 0.1% formic acid, at a flow rate of 300 nl/min. A gradient from 2 to 35% acetonitrile in 120 minutes was used. Mass spectra were acquired in a data-dependent manner, with an automatic switch between MS and MS/MS using a top 12 method. MS spectra were acquired in the Orbitrap analyzer with a mass range of 300–1700 *m*/z and 70,000 resolution at *m*/*z* 200. HCD peptide fragments were obtained using a normalized collision energy of 28 with an AGC target value of 5 × 10^4^ at 35,000 resolution. Dynamic exclusion (±10 ppm tolerance) was used with one repeat count, 30 s exclusion duration. Measurements have been performed using internal lock mass calibration on m/z 371.10124 and 445.12003. Raw spectra were processed with Mascot Distiller 2.4.3.3 (Matrix Science), and subsequent protein identification was performed using Mascot Version 2.4.1 (Matrix Science) as the search engine. Mascot generic files (.mgf) were searched, against a UniProt database containing the forward and reverse sequences of the rat proteome. The following Mascot search settings were used: maximum missed cleavages: 1; peptide mass tolerance: 10 ppm; and fragment ion tolerance: 0.05 Da. Carbamidomethyl (C), was specified as fixed modification, whereas Oxidation (M) was specified as variable modifications. Scaffold (version Scaffold_4.2.1, Proteome Software) was used to validate MS/MS based peptide and protein identifications. Peptide identifications were accepted if they could be established at greater than 95.0% probability by the Peptide Prophet algorithm^69^ with Scaffold delta-mass correction. Protein identifications were accepted if they could be established at greater than 95.0% probability and contained at least 2 identified peptides. For peptide and protein identifications 1% false discovery rate was applied for inclusion. Protein probabilities were assigned by the Protein Prophet algorithm^70^. Proteins that contained similar peptides and could not be differentiated based on MS/MS analysis alone were grouped to satisfy the principles of parsimony. Proteins sharing significant peptide evidence were grouped into clusters.

### Primary rat fibroblasts

Rat lung fibroblasts were extracted from healthy adult male or female rats. Lungs were extracted, washed in sterile PBS, dissected into smaller segments and incubated in plain DMEM supplemented with 1 mg/ml collagenase (Sigma) for 3 h at 37°C. Tissue debris was filtered; cells were collected by centrifugation (1100 RPM/5 min) and plated in DMEM supplemented with 10% foetal calf serum (FCS) and penicillin/streptomycin (1:100). Fibroblasts are able to stick to the plastic culture dish and the remaining floating cells e.g. blood cells and few macrophages are washed-off by gentle tapping and replacing the medium daily for 7 days.

### Stimulation of cells with conditioned medium

Conditioned medium from injured spinal cords was generated 1 week post-injury, by placing injury epicentre explants (T10) in plain DMEM *ex vivo*. Explants were cultured for 24 hours to allow release of bioactive products from injured explants. Conditioned medium was collected and diluted 1:4 with plain DMEM before being applied to resting fibroblasts. For signalling experiments, cells were stimulated with the conditioned medium for 25 minutes (acute activation). For gene expression experiments, stimulation with conditioned medium was stopped after 3 hours to prevent production and release of cytokines by the stimulated cells, as this would influence the inflammatory milieu.

### Western blotting

Spinal cord tissue extracts were denatured and reduced in 4× sample buffer containing 500 mM Tris, pH 6.8, 40% glycerol, 0.2% SDS, 2% β-mercaptoethanol, and 0.02% bromophenol blue and boiled at 98°C for 10 min. 15 μg of protein per sample were loaded and separated on 15-well, Bis-Tris, 4–12% polyacrylamide gradient gels (NuPAGE, Invitrogen). For signalling experiments cells were lysed in 0.2% SDS suplemmented with proteinase inhibitor cocktail, 12.5 mM EDTA and a complete phosphatase inhibitor cocktail (P0044, Sigma). 160 μl of buffer were used in each well (24-well plate) with ~1 million cells. Cell lysates were quantified and mixed with 80 μl sample buffer. 15 μg of protein per sample was loaded in each well as above for immunoblotting. Proteins were then transferred on nitrocellulose membranes. Membranes were stained with Ponceau-Red to visualize successful transfer and protein loading, blocked in 5% fat-free milk powder in PBS and commonly probed for 16 hours at 4°C with primary antibodies (1:500 dilution) as indicated. Membranes were washed 3 times in PBS supplemented with tween-20 (1:1000) and then appropriate horseradish peroxidase conjugated secondary antibodies were applied (DAKO) diluted 1:2000 in 5% fat-free milk, for 1 hour at room temperature. Finally, after 3 washes with PBS and tween-20, membranes were developed with enhanced chemiluminescence (ECL Prime, GE Healthcare) and immunoreactivity was visualized in a UVP BioSpectrum instrument and VisionWorks LS software. Protein levels were quantified using the densitometry function of the ImageJ 64 software.

### RNA extraction and quantitative PCR

Total RNA was extracted from T10 spinal cord tissue explants using Trizol reagent (Sigma). One spinal cord explant was used for each experimental sample. The tissue was homogenised in Trizol and the aqueous (RNA-containing) phase was generated using 1:10 bromo-chloro-propane (BCP) and 30 min centrifugation at 12,000 rpm. The aqueous layer was then mixed 1:1 with 70% ethanol. For the extraction of RNA from cultured fibroblasts, cells were lysed in TRK lysis buffer (Omega Bio-Tek). 250 μl of TRK lysis buffer was used for each well of a 24-well plate (8 × 10^5^–10 × 10^5^ cells). The homogenate was mixed 1:1 with 70% ethanol. Trizol or TRK extracted RNA was purified using the EZNA total RNA kit I (Omega Bio-Tek). 2000 ng of RNA per sample were converted into cDNA using the high capacity RNA-to-cDNA kit (Applied Biosystems). 20 ng of RNA per reaction were quantified using rat prevalidated TaqMan primer/probe mixes (Applied Biosystems), at a concentration of 900 nM for the primer and 200 nM for the probe. Real-time PCR was performed using an automated Roche thermocycler. Fold change in the mRNA expression of target genes was calculated relative to appropriate controls (intact tissue or unstimulated cells) using the ΔΔCt method. GAPDH served as the housekeeping gene. All short amplicon primer/probes were designed, tested and recommended by the provider (Applied Biosystems) as they detect the maximum number of transcripts for the gene of interest, they do not detect homologs, they sequence across an exon-exon junction, they do not hybridize with multiple off-target genes and they do not target the 5’ UTR. The following pre-validated rat primer/probes were used: TLR4 (Rn00569848_m1), RAGE (Rn01525753_g1), TNFα (Rn01525859_g1), COX2 (Rn01483828_m1), IL1β (Rn00580432_m1), iNOS (Rn00561646_m1) and IL6 (Rn01410330_m1), all from Applied Biosystems.

### RNA interference for TLR4 and RAGE

In order to examine the effect of TLR4 and RAGE, expression of the receptors was inhibited using small-interfering RNA (siRNA). First, 70–80% confluent fibroblasts were incubated with 1.5% FCS containing DMEM, supplemented with siRNA transfection reagent (jetPEI, Polyplus) pre-mixed with either scrambled siRNA (negative control sequence that does not target any gene product, 4457287, Ambion), TLR4 (s131043, Ambion) or RAGE (s135582, Ambion) siRNAs. The transfection reagent was used at 1:5000 dilution and siRNA concentration was 20 pM. Cells were incubated with the siRNA mix for 5 h. Subsequently, the culture medium was replaced with fresh DMEM and 1.5% FCS and cells were rested overnight. The transfection was repeated the following day and after 5 h, culture medium (DMEM and 1.5% FCS) was replaced and cells were kept for 36 hours. Finally, prior to stimulation with conditioned medium siRNA transfected cells were serum-starved in plain DMEM for 3–4 h.

### Inhibition of the IL1R, TNFR, IKK2 (NFκΒ) and HMGB1

To test the involvement of IL-1 and TNFα, the IL-1 receptor was blocked using Anakinra (IRAP: IL-1 Receptor Antagonist Protein; SRP6006, Sigma) and TNFα activity was blocked using a soluble form of its receptor, sTNFαRII (310–12, PeproTech). Both inhibitors were used at a concentration of 20 ng/ml. Fibroblasts and conditioned medium generated as above, were pre-incubated with either IRAP or sTNFαRII for 1 hour. Subsequently, fibroblasts were stimulated with the pre-treated conditioned medium, supplemented with either IRAP or sTNFαRII. Positive controls were stimulated with conditioned medium alone and negative controls were kept in plain DMEM. IKK2 was inhibited using 400 nM TPCA-1 (T1452, Sigma, [5-(p-Fluorophenyl)-2-ureido]thiophene-3-carboxamide)^40^. Soluble extracellular HMGB1 was neutralized in the conditioned medium using mouse anti-HMGB1 (clone 3E8; Biolegend). For neutralization, the 1 week injury conditioned medium was incubated for 2 hours with 5 μg/ml of either anti-HMGB1 or isotype control antibodies prior to stimulation of cells.

### Statistical and bioinformatics analysis

Differential protein expression was measured using spectral counting. All assigned spectra for each protein were used. Before statistical analysis, protein spectral counts were normalized using the Scaffold software (version Scaffold_4.2.1, Proteome Software). Normalized spectra were computed by calculating and averaging the number of identified spectra in each sample, then multiplying the number of spectra assigned to each protein by the ratio of the average spectral count and the number of total spectra in that sample. Differential expression was estimated by unpaired, two-tailed t-test of normalized spectra for each identified protein: Control (N = 3) vs. Injured (N = 3). Only proteins identified with at least two unique peptides were included in the analysis and no outliers were removed. Hierarchical clustering and principal component analysis were performed using the GenePattern platform. For hierarchical dendrograms, pairwise similarity in spectral counts between different proteins (rows) was computed using Pearson correlation coefficient. Spectral counts of the entire proteomics dataset (2346 proteins) were used for principal component analysis. The two first principal components (PC1 and PC2) were used to capture the variance of proteomics profiling between the 3 controls and 3 injured spinal cord specimens. For comparison of our proteomics data with high-throughput gene expression analysis, differentially regulated protein identifications were compared with differentially regulated transcripts (N = 3 controls vs. N = 3 injured) from a published microarray dataset of female rat T8 spinal contusion 5 weeks post-injury (E-GEOD-2599, ArrayExpress EBI) performed by Aimone and colleagues^29^. Protein interaction networks were created using the String database (StringDB v9.1^26^) of known and predicted protein-protein interactions and inferring protein associations from co-expression data, according to the standard network instructions provided with a medium stringency threshold of association (0.4). *In vitro* experiments were performed 3–6 times (independent experiments) as indicated in corresponding figure legends. Densitometry and TaqMan qPCR data were analysed by one-way ANOVA and different treatments were compared with Fisher’s least significant difference (LSD) multiple comparison test. GraphPad Prism v6 software was used to carry out statistical tests and obtain *P* values. All data is presented as mean +SEM.

## Additional Information

**How to cite this article**: Didangelos, A. *et al.* High-throughput proteomics reveal alarmins as amplifiers of tissue pathology and inflammation after spinal cord injury. *Sci. Rep.* **6**, 21607; doi: 10.1038/srep21607 (2016).

## Supplementary Material

Supplementary Information
