# Supplementary material for: High-throughput proteomics reveal alarmins as amplifiers of tissue pathology and inflammation after spinal cord injury
Source: Sci Rep. 2016 Feb 22;6:21607. doi: 10.1038/srep21607 (PMC4761922; doi:10.1038/srep21607)
Supplement: Supplementary Information [file srep21607-s1.pdf]

Supplementary Material

High-throughput proteomics reveal alarmins as amplifiers of tissue pathology and inflammation after spinal cord injury

Authors.
Athanasios Didangelos[1]*, Michele Puglia[2], Michaela Iberl[1], Candela Sanchez-Bellot[1], Bernd Roschitzki[2] and Elizabeth J. Bradbury[1]*.

* Corresponding authors
Elizabeth.bradbury@kcl.ac.uk; +44 (0) 207 848 6183
Athanasios.didangelos@kcl.ac.uk; +44 (0) 207 848 6183

Affiliations.
Wolfson Center for Age Related Diseases, Guys Campus, King's College London, United Kingdom[1].

Functional Genomics Center Zurich (FGCZ), ETH Zurich, University of Zurich, Switzerland[2].

Supplementary files in the following order:

- **Supplementary Figure S1**
- **Supplementary Figure S2**
- **Supplementary Figure S3**
- **Supplementary Figure S4**
- **Supplementary Figure S5**
- **Supplementary Table S1** (Full spectrum counting report).
- **Supplementary Table S2**
- **Supplementary Table S3A & S3B**

Supplementary Figure S1

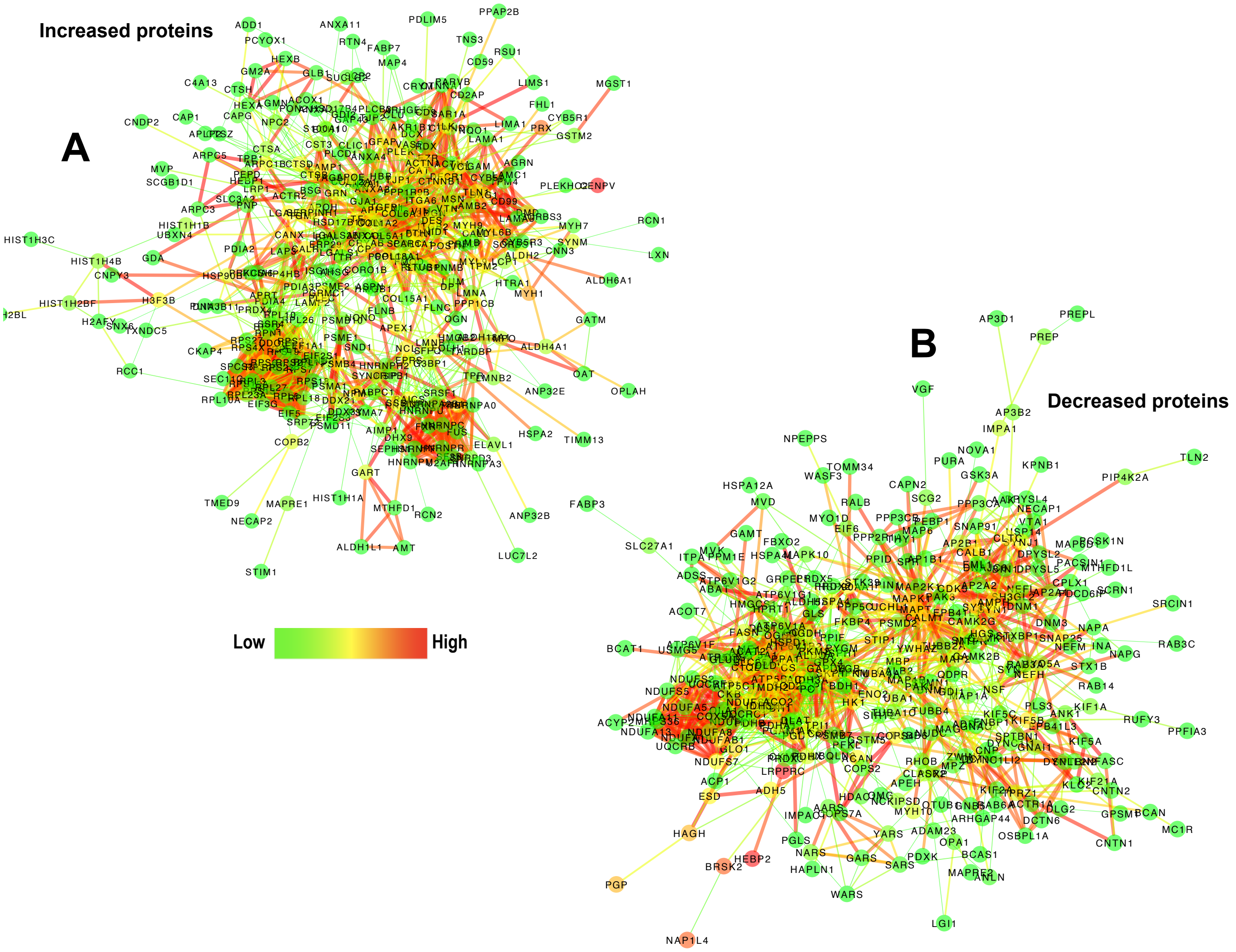

Supplementary Figure S1: Network analysis of proteomics data. Network analysis of differentially regulated proteins (t-test *P*≤0.05) performed using the String database (StringDB, v9.1). **A:** Upregulated proteins. **B:** Downregulated proteins. Proteins form highly interconnected networks. Node color indicates betweeness centrality while edge color indicates interaction score based on the predicted functional links between nodes (green: low values; red: high values).

Supplementary Figure S2

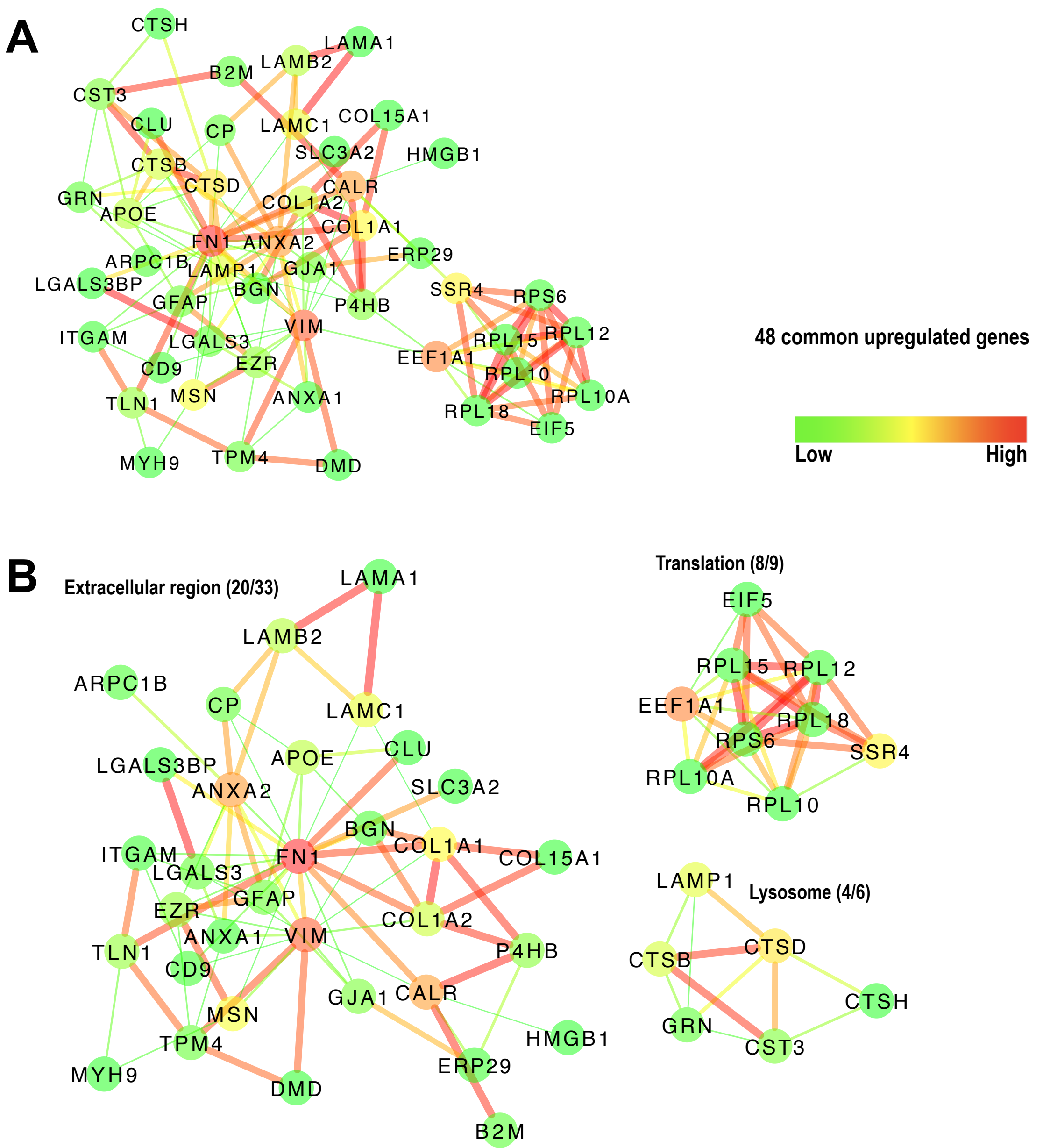

Supplementary Figure S2: Network analysis of persistently upregulated molecules. A: Network analysis (StringDB, v9.1) of the 48 molecules that were upregulated both at the mRNA and protein level. Node color indicates betweeness centrality while edge color indicates interaction score based on the predicted functional links between nodes (green: low values; red: high values). **B**: Subnetworks were generated by MCL clustering (1.9 inflation value & 0.4 edge weight cut-off) of network in **A**. Each subnetwork was analysed by Bingo to identify the predominant gene ontology term as indicated. The number of genes occupying the dominant ontology are shown. Genes downregulated both at the mRNA and protein level are shown in **Supplementary Figure S3**.

Supplementary Figure S3

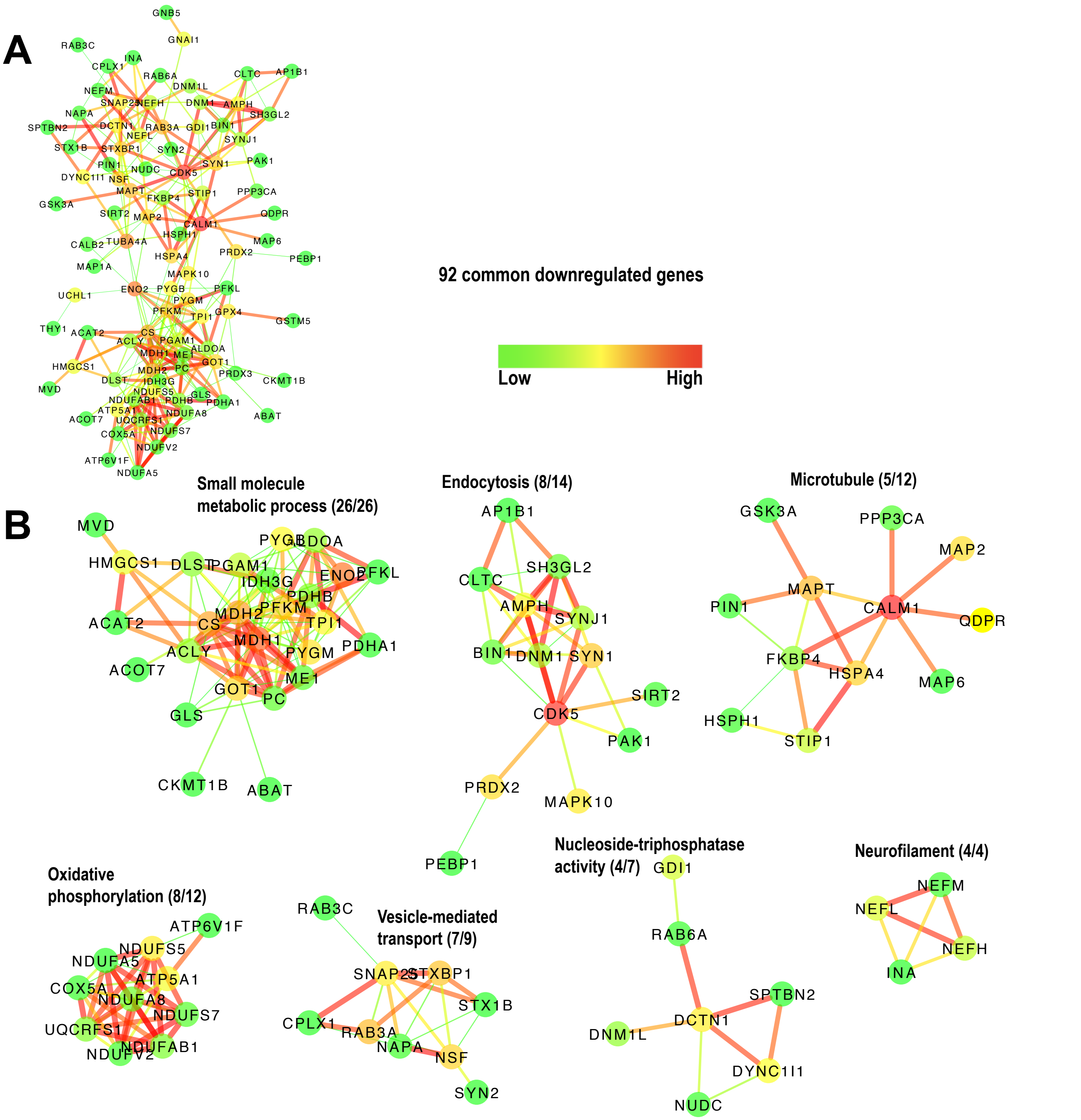

Supplementary Figure S3: Network analysis of persistently downregulated molecules. A: Network analysis (StringDB, v9.1) of 92 genes downregulated both at the mRNA and protein level. Node color indicates betweeness centrality while edge color indicates interaction score based on the predicted functional links between nodes (green: low values; red: high values). **B**: Subnetworks were generated by MCL clustering (1.9 inflation value & 0.4 edge weight cut-off) of network in **A**. Each subnetwork was analysed by Bingo to identify the predominant gene ontology term as indicated.

Supplementary Figure S4

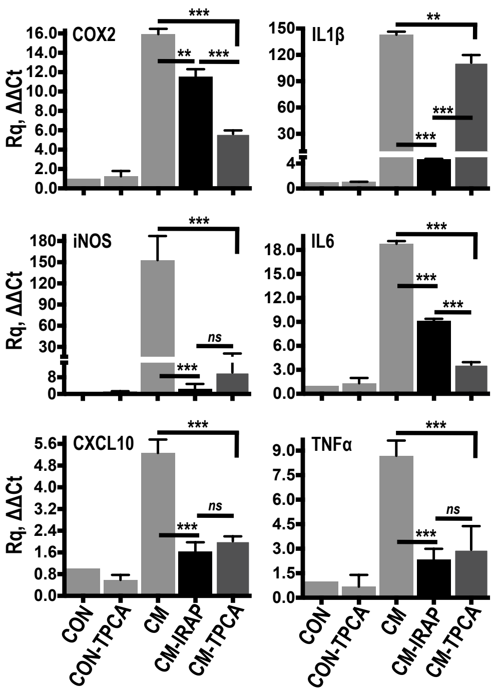

Supplementary Figure S4: IL1 and NFκB regulate inflammatory gene expression. Resting fibroblasts were stimulated for 3 hours with injury conditioned medium (CM) supplemented with either 20ng/ml of IRAP (CM-IRAP) or 400nM IKK2 inhibitor TPCA (CM-TPCA). Control cells were kept in plain culture medium (CON) and additional controls were incubated with 400nM TPCA (CON-TPCA). Gene expression was measured by TaqMan qPCR. ACTB served as the housekeeping gene. n=3; ** p≥0.01, *** p≥0.001, *ns:* not significant; Anova with Fisher's LSD multiple comparison test.

Supplementary Figure S5

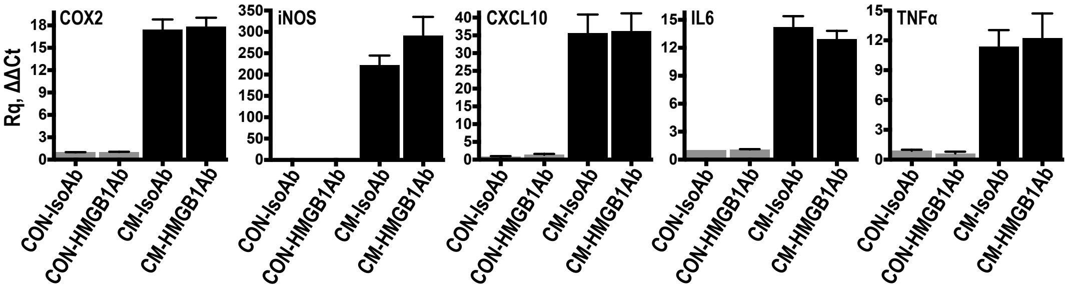

Supplementary Figure S5: Neutralization of soluble extracellular HMGB1 in injury conditioned medium. Resting cells were stimulated with injury conditioned medium which was previously treated either with HMGB1 antibodies (HMGB1Ab; 5µg/ml) to neutralize soluble extracellular HMGB1, or isotype control antibodies (IsoAb; 5µg/ml). Inflammatory gene expression was measured by TaqMan qPCR. *N*=6. No significant differences in COX2, iNOS, CXCL10, IL6 and TNF mRNA expression between isotype and HMGB1 antibody treatment were observed.

Supplementary Table S1

Supplementary Table1: Full spectrum counting report.

Database Set: 1 Database

Database Name: the fgcz_10116_d_20130418 database

Taxonomy: All Entries
Number of Proteins: 57823

Search Engine Set: 1 Search Engine

Search Engine: Mascot

Version: 2.4.1
Samples: All Samples
Fragment Tolerance: 0.050 Da (Monoisotopic)
Parent Tolerance: 10.0 PPM (Monoisotopic)
Fixed Modifications: +57 on C (Carbamidomethyl)
Variable Modifications: +16 on M (Oxidation)
Database: the fgcz_10116_d_20130418 database (unknown version, 57823 entries)
Digestion Enzyme: Trypsin
Max Missed Cleavages: 1

Scaffold: Version: Scaffold_4.2.1

Source: /misc/FGCZ/SCAFFOLD

Protein Grouping Strategy: Experiment-wide grouping with protein cluster analysis
Peptide Thresholds: >95.0%
Protein Thresholds: >95.0% and 2 peptides minimum

Displaying:Quantitative Value (Normalized Total Spectra)
Proteins are sorted according to fold change: INJ/CON

Identified Proteins (2346)	Accession Number	Molecular Weight	8w INJ2	8w INJ3	8w INJ1	CON1	CON3	CON2	ttest	fold change (INJ/CON)
**Protein Myh1 Myh1**	tr|F1LRV9|F1LRV9_RAT	223 kDa	22.339	38.625	25.234	1	1	1	0.005229483	28.73266667
**Uncharacterized protein (Myosin, N-terminal, SH3-like)**	tr|G3V6E1|G3V6E1_RAT	220 kDa	19.293	41.756	22.322	1	1	1	0.018991159	27.79033333
**Filamin alpha Flna**	tr|C0JPT7|C0JPT7_RAT	280 kDa	27.416	35.493	23.293	1	1.9057	1	0.001585089	22.07081957
**Myosin-4 Myh4**	tr|F1LMU0|F1LMU0_RAT	223 kDa	23.354	25.054	17.469	1	1	1	0.000801769	21.959
**Annexin A1 Anxa1**	sp|P07150|ANXA1_RAT	39 kDa	22.339	20.878	19.411	1	1	1	1.93883E-05	20.876
**Fibronectin Fn1**	sp|P04937-4|FINC_RAT (+3)	259 kDa	35.539	38.625	18.44	1	1.9057	2.0419	0.009674443	18.71695367
**Protein RGD1560687 (similar to Ferritin light chain)**	tr|M0R6L9|M0R6L9_RAT	21 kDa	17.262	21.922	15.528	1	1	1	0.000833491	18.23733333
**Periostin, osteoblast specific factor Postn**	tr|D3ZAF5|D3ZAF5_RAT	90 kDa	19.293	29.229	4.8526	1	1	1	0.076590363	17.79153333
**Isoform 2 of Tropomyosin beta chain Tpm2**	sp|P58775-2|TPM2_RAT	33 kDa	14.216	19.834	13.587	1	1	1	0.001697166	15.879
**Tropomyosin beta chain Tpm2**	sp|P58775|TPM2_RAT	33 kDa	12.185	21.922	12.617	1	1	1	0.010115728	15.57466667
**Filamin, beta (Predicted) Flnb**	tr|D4A8D5|D4A8D5_RAT	275 kDa	12.185	21.922	10.676	1	1	1	0.016789588	14.92766667
**Galectin-3 Lgals3**	sp|P08699|LEG3_RAT	27 kDa	13.2	16.703	19.411	1	1.9057	1	0.001149181	12.62616176
**Beta-enolase Eno3**	sp|P15429|ENOB_RAT	47 kDa	1	15.659	14.558	1	1	1	0.116721742	10.40566667
**Protein Lrp1 Lrp1**	tr|G3V928|G3V928_RAT	505 kDa	9.1386	12.527	7	1	1	1	0.006017983	9.5552
**Desmin Des**	tr|Q6P725|Q6P725_RAT (+1)	53 kDa	25.385	42.8	13.587	1	1	7.1467	0.05015532	8.940054883
**Hemoglobin subunit beta-2**	sp|P11517|HBB2_RAT	16 kDa	11.169	9.3952	5.8232	1	1	1	0.007711273	8.7958
**Alpha-2-HS-glycoprotein Ahsg**	sp|P24090|FETUA_RAT	38 kDa	6.0924	7.3073	12.617	1	1	1	0.018621964	8.672233333
**Ceruloplasmin Cp**	tr|G3V7K3|G3V7K3_RAT	121 kDa	26.4	27.142	26.204	3.0063	3.8114	3.0629	4.51511E-07	8.07096735
**Protein Col14a1 Col14a1**	tr|D3ZZT9|D3ZZT9_RAT	193 kDa	3.0462	18.79	1.9411	1	1	1	0.27204517	7.925766667
**Non-muscle caldesmon Cald1**	sp|Q62736|CALD1_RAT (+1)	61 kDa	10.154	8.3513	4.8526	1	1	1	0.012058264	7.785966667
**Tubulointerstitial nephritis antigen-like Tinagl1**	sp|Q9EQT5|TINAL_RAT	53 kDa	8.1232	9.3952	4.8526	1	1	1	0.008824367	7.457
**Cytoskeleton-associated protein 4 (Predicted) Ckap4**	tr|D3ZH41|D3ZH41_RAT	36 kDa	6.0924	9.3952	6.7937	1	1	1	0.003065742	7.4271
**Actin-related protein 2/3 complex subunit 1B Arpc1b**	sp|O88656|ARC1B_RAT	41 kDa	9.1386	7.3073	5.8232	1	1	1	0.002583611	7.423033333
**Uncharacterized protein**	tr|M0R9D5|M0R9D5_RAT	572 kDa	129.97	153.45	81.524	20.042	16.199	13.273	0.007794155	7.370521469
**Integrin, alpha 6, isoform CRA_b Itga6**	tr|G3V991|G3V991_RAT (+1)	122 kDa	11.169	13.571	11.646	1	1.9057	2.0419	0.00019969	7.354272779
**Taste receptor type 1 member 2 Aldh4a1**	tr|F8WFI0|F8WFI0_RAT	62 kDa	7.1078	8.3513	5.8232	1	1	1	0.001124549	7.0941
**Ig gamma-2A chain C region Igg-2a**	sp|P20760|IGG2A_RAT (+1)	35 kDa	7.1078	12.527	1	1	1	1	0.152238246	6.878266667
**Myosin light chain 1/3, skeletal muscle isoform Myl1**	sp|P02600|MYL1_RAT	21 kDa	5.077	12.527	2.9116	1	1	1	0.115467254	6.838533333
**IQ motif containing GTPase activating protein 1 (Predicted), isoform CRA_b Iqgap1**	tr|G3V7Q7|G3V7Q7_RAT	189 kDa	6.0924	11.483	2.9116	1	1	1	0.080243359	6.829
**Lysosome-associated membrane glycoprotein 1 Lamp1**	sp|P14562|LAMP1_RAT	44 kDa	6.0924	7.3073	6.7937	1	1	1	8.33724E-05	6.731133333
**Protein Flnc Flnc**	tr|D3ZHA0|D3ZHA0_RAT	291 kDa	61.94	48.02	61.143	7.0146	10.481	8.1677	0.000468882	6.667225181
**Asporin Aspn**	tr|Q5XIH1|Q5XIH1_RAT	43 kDa	25.385	30.273	9.7053	6.0125	1	3.0629	0.044464376	6.487414892
**Laminin, alpha 1 Lama1**	tr|D4A409|D4A409_RAT	338 kDa	7.1078	9.3952	2.9116	1	1	1	0.044921228	6.471533333
**Nestin Nes**	tr|D3ZWJ2|D3ZWJ2_RAT (+4)	199 kDa	3.0462	10.439	5.8232	1	1	1	0.065260351	6.436133333
**Apolipoprotein E Apoe**	sp|P02650|APOE_RAT	36 kDa	30.462	31.317	29.116	4.0083	1	9.1887	0.000496198	6.40240896
**Isoform 2 of Reticulon-4 Rtn4**	sp|Q9JK11-2|RTN4_RAT	39 kDa	7.1078	5.2195	6.7937	1	1	1	0.000775786	6.373666667
**Protein Lama4 Lama4**	tr|F1LTF8|F1LTF8_RAT	196 kDa	9.1386	7.3073	1.9411	1	1	1	0.076410534	6.129
**CD166 antigen Alcam**	sp|O35112|CD166_RAT	65 kDa	4.0616	8.3513	5.8232	1	1	1	0.015103398	6.0787
**Guanine deaminase Gda**	tr|Q9JKB7|Q9JKB7_RAT	51 kDa	12.185	14.615	8.7347	1	1.9057	3.0629	0.005496669	5.953607211
**Myosin regulatory light chain 2, skeletal muscle isoform Mylpf**	sp|P04466|MLRS_RAT	19 kDa	3.0462	13.571	1	1	1	1	0.279063836	5.8724
**Laminin, alpha 5, isoform CRA_a Lama5**	tr|F1MAN8|F1MAN8_RAT	404 kDa	4.0616	8.3513	4.8526	1	1	1	0.022599303	5.755166667
**Procollagen, type XVIII, alpha 1 Col18a1**	tr|F1LR02|F1LR02_RAT	135 kDa	5.077	7.3073	4.8526	1	1	1	0.003750545	5.745633333
**Transmembrane glycoprotein NMB Gpnmb**	sp|Q6P7C7|GPNMB_RAT	64 kDa	7.1078	5.2195	4.8526	1	1	1	0.002490296	5.726633333
**Isoform 1 of Gamma-adducin Add3**	sp|Q62847-2|ADDG_RAT	75 kDa	1	14.615	1	1	1	1	0.373900966	5.538333333
**Gap junction alpha-1 protein Gja1**	sp|P08050|CXA1_RAT	43 kDa	9.1386	5.2195	12.617	1	1.9057	2.0419	0.027358532	5.452158622
**PDZ and LIM domain protein 5 Pdlim5**	sp|Q62920|PDLI5_RAT	63 kDa	7.1078	8.3513	5.8232	1	1.9057	1	0.001840186	5.449036024
**Integrin beta-1 Itgb1**	sp|P49134|ITB1_RAT	88 kDa	2.0308	8.3513	5.8232	1	1	1	0.074633968	5.401766667
**1-phosphatidylinositol 4,5-bisphosphate phosphodiesterase delta-1 Plcd1**	tr|G3V9D1|G3V9D1_RAT	86 kDa	4.0616	6.2634	5.8232	1	1	1	0.002864824	5.382733333
**Myosin-binding protein C, slow-type Mybpc1**	tr|D4A2S4|D4A2S4_RAT	130 kDa	1	13.571	1	1	1	1	0.373900966	5.190333333
**Complement C3 C3**	tr|M0RBF1|M0RBF1_RAT (+1)	186 kDa	6.0924	8.3513	1	1	1	1	0.129055664	5.1479
**Carboxypeptidase Q Cpq**	sp|Q6IRK9|CBPQ_RAT	52 kDa	4.0616	8.3513	2.9116	1	1	1	0.068066817	5.108166667
**Nucleobindin-2 Nucb2**	sp|Q9JI85|NUCB2_RAT (+1)	50 kDa	5.077	6.2634	3.8821	1	1	1	0.004061218	5.074166667
**Protein Stat1 Stat1**	tr|F1M9D6|F1M9D6_RAT	87 kDa	8.1232	2.0878	4.8526	1	1	1	0.08245465	5.0212
**Calumenin Calu**	tr|G3V6S3|G3V6S3_RAT	37 kDa	6.0924	10.439	2.9116	1	1.9057	1	0.078383696	4.978108918
**Protein Hspg2 Hspg2 Perlecan**	tr|F1M566|F1M566_RAT	231 kDa	37.57	48.02	33.968	6.0125	7.6229	11.231	0.002153006	4.808014027
**Ab2-162 Fam65b**	tr|Q7TP54|Q7TP54_RAT	145 kDa	23.354	27.142	20.381	5.0104	4.7643	5.1048	0.00067754	4.763399308
**Protein Snx6 Snx6**	tr|B5DEY8|B5DEY8_RAT	47 kDa	4.0616	7.3073	2.9116	1	1	1	0.046068714	4.760166667
**Dermatopontin Dpt**	tr|D4A9H2|D4A9H2_RAT	20 kDa	5.077	6.2634	2.9116	1	1	1	0.018740268	4.750666667
**von Willebrand factor A domain-containing protein 1 Vwa1**	sp|Q642A6|VWA1_RAT	45 kDa	5.077	6.2634	2.9116	1	1	1	0.018740268	4.750666667
**Protein Uap1l1 Uap1l1**	tr|B5DEH4|B5DEH4_RAT	56 kDa	5.077	6.2634	2.9116	1	1	1	0.018740268	4.750666667
**Protein Ank2 Ank2**	tr|F1LZM2|F1LZM2_RAT	45 kDa	12.185	1	1	1	1	1	0.373900966	4.728333333
**Protein Ril Ril**	tr|M0R4H5|M0R4H5_RAT	36 kDa	8.1232	3.1317	2.9116	1	1	1	0.093973021	4.722166667
**LIM and senescent cell antigen-like domains 1 isoform D Lims1**	tr|C0KUC5|C0KUC5_RAT	42 kDa	4.0616	5.2195	4.8526	1	1	1	0.000407615	4.711233333
**Protein Rcn1 Rcn1**	tr|D3ZUB0|D3ZUB0_RAT	38 kDa	5.077	4.1756	4.8526	1	1	1	0.000166179	4.701733333
**Polypyrimidine tract-binding protein 1 Ptbp1**	tr|F1LM18|F1LM18_RAT	59 kDa	20.308	19.834	14.558	6.0125	4.7643	1	0.003856217	4.644725222
**Nidogen-2 Nid2**	sp|B5DFC9|NID2_RAT	153 kDa	9.1386	22.966	8.7347	3.0063	3.8114	2.0419	0.086176315	4.609609915
**Protein Krt81 Kb21**	tr|A7M775|A7M775_RAT	53 kDa	1	11.483	1	1	1	1	0.373900966	4.494333333
**Lipoma-preferred partner homolog Lpp**	sp|Q5XI07|LPP_RAT	68 kDa	3.0462	9.3952	1	1	1	1	0.240520158	4.480466667
**Protein Lmnb2 Lmnb2**	tr|D3ZLC1|D3ZLC1_RAT	67 kDa	10.154	13.571	7.7642	3.0063	1	3.0629	0.010978872	4.454422
**Protein Synm Synm**	tr|G3V9G5|G3V9G5_RAT	173 kDa	18.277	14.615	15.528	4.0083	2.8586	4.0839	0.000435846	4.421594769
**Myoglobin Mb**	sp|Q9QZ76|MYG_RAT	17 kDa	4.0616	6.2634	2.9116	1	1	1	0.025582797	4.4122
**Protein Vtn Vtn**	tr|Q3KR94|Q3KR94_RAT	55 kDa	4.0616	5.2195	3.8821	1	1	1	0.001272683	4.387733333
**LOC682908 protein Rcc1**	tr|B1H227|B1H227_RAT	45 kDa	6.0924	9.3952	5.8232	1	2.8586	1	0.01366579	4.386201787
**Proteasome subunit beta type-10 Psmb10**	sp|Q4KM35|PSB10_RAT	29 kDa	5.077	4.1756	3.8821	1	1	1	0.000714722	4.378233333
**Beta-hexosaminidase subunit beta Hexb**	sp|Q6AXR4|HEXB_RAT	62 kDa	3.0462	5.2195	4.8526	1	1	1	0.007378377	4.372766667
**Protein Tln1 Tln1**	tr|G3V852|G3V852_RAT	270 kDa	45.693	66.81	42.703	9.0188	13.34	13.273	0.00668456	4.355828221
**Proteasome (Prosome, macropain) activator subunit 1 Psme1**	tr|Q6P9V7|Q6P9V7_RAT	29 kDa	5.077	3.1317	4.8526	1	1	1	0.005477126	4.353766667
**Protein Dag1 Dag1**	tr|F1M8K0|F1M8K0_RAT	97 kDa	6.0924	9.3952	5.8232	2.0042	1.9057	1	0.010136532	4.340373531

Dystrophin Dmd	tr|F1LN35|F1LN35_RAT	71 kDa	6.0924	5.2195	5.8232	2.0042	1	1	0.000490839	4.279281754
Lamina-associated polypeptide 2, isoform beta Tmpo	sp|Q62733|LAP2_RAT	50 kDa	6.0924	7.3073	3.8821	1	1	2.0419	0.014151952	4.275662436
Lymphocyte cytosolic protein 1 Lcp1	tr|Q5XI38|Q5XI38_RAT	70 kDa	5.077	5.2195	6.7937	1	1	2.0419	0.002601708	4.228258987
Isoform 3 of Electrogenic sodium bicarbonate cotransporter 1 Slc4a4	sp|Q9JI66-3|S4A4_RAT	123 kDa	13.2	10.439	16.499	1	7.6229	1	0.02256964	4.171091875
Myosin-7 Myh7	tr|G3V8B0|G3V8B0_RAT	223 kDa	1	10.439	1	1	1	1	0.373900966	4.146333333
Phosphoglycerate mutase 2 Pgam2	sp|P16290|PGAM2_RAT	29 kDa	4.0616	7.3073	1	1	1	1	0.16150091	4.122966667
Retinal dehydrogenase 2 Aldh1a2	sp|Q63639|AL1A2_RAT	57 kDa	1	8.3513	2.9116	1	1	1	0.233510992	4.087633333
Integrin alpha M Itgam	tr|G3V8L7|G3V8L7_RAT	127 kDa	5.077	5.2195	1.9411	1	1	1	0.045095418	4.0792
Membrane-associated progesterone receptor component 1 Pgrmc1	sp|P70580|PGRC1_RAT	22 kDa	3.0462	6.2634	2.9116	1	1	1	0.048527751	4.073733333
Major vault protein Mvp	sp|Q62667|MVP_RAT	96 kDa	16.246	17.746	14.558	3.0063	3.8114	5.1048	0.000381813	4.072132523
Lupus La protein homolog Ssb	tr|Q66HM7|Q66HM7_RAT (+1)	48 kDa	4.0616	5.2195	2.9116	1	1	1	0.010035955	4.064233333
High mobility group protein B2 Hmgb2	sp|P52925|HMGB2_RAT	24 kDa	4.0616	5.2195	2.9116	1	1	1	0.010035955	4.064233333
SPARC-like protein 1 Sparcl1	sp|P24054|SPRL1_RAT (+1)	71 kDa	6.0924	3.1317	2.9116	1	1	1	0.04117056	4.045233333
Protein RT1-A RT1-A	tr|E9PSX3|E9PSX3_RAT (+1)	41 kDa	4.0616	4.1756	3.8821	1	1	1	3.72287E-06	4.039766667
SPARC Sparc	sp|P16975|SPRC_RAT	34 kDa	4.0616	4.1756	3.8821	1	1	1	3.72287E-06	4.039766667
Erlin-2 Erlin2	sp|B5DEH2|ERLN2_RAT	38 kDa	5.077	3.1317	3.8821	1	1	1	0.005886402	4.030266667
Galectin-3-binding protein Lgals3bp	sp|O70513|LG3BP_RAT	64 kDa	4.0616	3.1317	4.8526	1	1	1	0.003736367	4.0153
Serum paraoxonase/arylesterase 2 Pon2	sp|Q6AXM8|PON2_RAT	40 kDa	5.077	4.1756	6.7937	2.0042	1	1	0.008699966	4.007367264
Prostaglandin reductase 1 Ptgr1	sp|P97584|PTGR1_RAT	36 kDa	3.0462	3.1317	5.8232	1	1	1	0.030193537	4.000366667
Catenin (Cadherin associated protein), alpha 1 Ctnna1	tr|Q5U302|Q5U302_RAT	100 kDa	15.231	18.79	17.469	3.0063	4.7643	5.1048	0.000464506	3.999099057
Electrogenic sodium bicarbonate cotransporter 1 Slc4a4	sp|Q9JI66|S4A4_RAT	121 kDa	12.185	9.3952	16.499	1	7.6229	1	0.034951947	3.957143896
sp|ANXA5_HUMAN|	zz|ZZ_FGCZCont0109|	36 kDa	1	1	9.7053	1	1	1	0.373900966	3.901766667
Fibromodulin Fmod	sp|P50609|FMOD_RAT (+1)	43 kDa	15.231	11.483	7.7642	2.0042	1.9057	5.1048	0.024015993	3.82466416
Sorbin and SH3 domain-containing protein 1 (Fragment) Sorbs1	tr|F1M8Z8|F1M8Z8_RAT	104 kDa	15.231	17.746	15.528	6.0125	5.7171	1	0.00273347	3.810410382
Amyloid beta A4 protein App	sp|P08592|A4_RAT	87 kDa	9.1386	9.3952	7.7642	1	2.8586	3.0629	0.001463038	3.799465434
von Willebrand factor A domain-containing protein 5A Vwa5a	sp|Q75WE7|VWA5A_RAT	91 kDa	6.0924	5.2195	3.8821	2.0042	1	1	0.00675837	3.794515758
Protein kinase C delta-binding protein Prkcdbp	sp|Q9Z1H9|PRDBP_RAT	28 kDa	5.077	5.2195	4.8526	1	1	2.0419	0.000522546	3.748014548
Protein Tpr Tpr	tr|F1MA98|F1MA98_RAT	267 kDa	4.0616	5.2195	1.9411	1	1	1	0.046150587	3.740733333
1-phosphatidylinositol 4,5-bisphosphate phosphodiesterase beta-3 Plcb3	sp|Q99JE6|PLCB3_RAT (+1)	139 kDa	4.0616	5.2195	1.9411	1	1	1	0.046150587	3.740733333
Apolipoprotein H Apoh	tr|Q5I0M1|Q5I0M1_RAT	38 kDa	4.0616	5.2195	1.9411	1	1	1	0.046150587	3.740733333
Protein Hbb-b1 MGC72973	tr|Q62669|Q62669_RAT	16 kDa	5.077	4.1756	1.9411	1	1	1	0.042808725	3.731233333
Protein Sorbs3 Sorbs3	tr|Q5XIL4|Q5XIL4_RAT	82 kDa	5.077	4.1756	1.9411	1	1	1	0.042808725	3.731233333
Protein Vasp Vasp	tr|F7EWC1|F7EWC1_RAT	40 kDa	3.0462	5.2195	2.9116	1	1	1	0.02187411	3.725766667
Putative lysozyme C-2 Lyz2	tr|F1M8E9|F1M8E9_RAT (+1)	17 kDa	4.0616	4.1756	2.9116	1	1	1	0.002541039	3.716266667
Beta-hexosaminidase subunit alpha Hexa	sp|Q641X3|HEXA_RAT	61 kDa	4.0616	4.1756	2.9116	1	1	1	0.002541039	3.716266667
Transmembrane protein 43 Tmem43	sp|Q5XIP9|TMM43_RAT	45 kDa	4.0616	4.1756	2.9116	1	1	1	0.002541039	3.716266667
Elongation factor 1-alpha RGD1566344	tr|D3ZXS6|D3ZXS6_RAT	50 kDa	9.1386	1	1	1	1	1	0.373900966	3.712866667
Isoform C of Amyloid-like protein 2 Aplp2	sp|P15943-3|APLP2_RAT (+6)	81 kDa	5.077	3.1317	2.9116	1	1	1	0.017046547	3.706766667
Integrin-linked protein kinase Ilk	sp|Q99J82|ILK_RAT	51 kDa	6.0924	7.3073	4.8526	1	1.9057	2.0419	0.004733714	3.689121999
Protein S100-A4 S100a4	sp|P05942|S10A4_RAT	12 kDa	2.0308	3.1317	5.8232	1	1	1	0.077395022	3.6619
Macrophage-capping protein Capg	sp|Q6AYC4|CAPG_RAT	39 kDa	8.1232	7.3073	5.8232	1	3.8114	1	0.011152115	3.657242661
Histone H1.5 Hist1h1b	sp|D3ZBN0|H15_RAT	23 kDa	12.185	15.659	11.646	3.0063	4.7643	3.0629	0.002307846	3.64517469
Glutathione S-transferase Mu 2 Gstm2	sp|P08010|GSTM2_RAT	26 kDa	6.0924	12.527	6.7937	3.0063	1	3.0629	0.046552896	3.594904657
Dolichyl-diphosphooligosaccharide--protein glycosyltransferase 48 kDa subunit Ddost	sp|Q641Y0|OST48_RAT	49 kDa	8.1232	6.2634	6.7937	3.0063	1.9057	1	0.003151924	3.582594723
Protein Rrbp1 Rrbp1	tr|F1M5X1|F1M5X1_RAT (+1)	157 kDa	27.416	28.185	24.263	7.0146	8.5757	7.1467	0.000126307	3.512512645
Serine (Or cysteine) proteinase inhibitor, clade H, member 1, isoform CRA_b Serpinh1	tr|Q5RJR9|Q5RJR9_RAT	47 kDa	15.231	20.878	12.617	7.0146	2.8586	4.0839	0.013237073	3.49112638
Dolichyl-diphosphooligosaccharide--protein glycosyltransferase subunit 2 Rpn2	sp|P25235|RPN2_RAT	69 kDa	11.169	15.659	10.676	3.0063	4.7643	3.0629	0.006211765	3.461854433
Protein Lamb1 Lamb1	tr|D3ZQN7|D3ZQN7_RAT	203 kDa	9.1386	15.659	5.8232	3.0063	3.8114	2.0419	0.068782187	3.456228272
LOC681126 protein Krt83	tr|A7M746|A7M746_RAT	55 kDa	1	8.3513	1	1	1	1	0.373900966	3.450433333
Adipocyte enhancer-binding protein 1 Aebp1	sp|A2RUV9|AEBP1_RAT	128 kDa	1	8.3513	1	1	1	1	0.373900966	3.450433333
Protein Kank2 Kank2	tr|D4ACC2|D4ACC2_RAT	90 kDa	2.0308	7.3073	1	1	1	1	0.278725797	3.446033333
Lamin A, isoform CRA_b Lmna	tr|G3V8L3|G3V8L3_RAT	74 kDa	72.094	75.161	47.556	20.042	16.199	20.419	0.006471827	3.438245676
Protein Sf3a1 Sf3a1	tr|D3ZQM0|D3ZQM0_RAT	89 kDa	3.0462	6.2634	1	1	1	1	0.186922148	3.436533333
Ldb3 protein Ldb3	tr|Q5XIG1|Q5XIG1_RAT	31 kDa	3.0462	6.2634	1	1	1	1	0.186922148	3.436533333
Isoform 2 of Fibrinogen beta chain Fgb	sp|P14480-2|FIBB_RAT (+1)	57 kDa	3.0462	6.2634	1	1	1	1	0.186922148	3.436533333
Proteasome subunit beta type-8 Psmb8	sp|P28064|PSB8_RAT	31 kDa	2.0308	6.2634	1.9411	1	1	1	0.166055269	3.411766667
Protein Glipr2 Glipr2	tr|F1M5V2|F1M5V2_RAT	17 kDa	9.1386	8.3513	9.7053	3.0063	1.9057	3.0629	0.00029756	3.410099186
Isoform 2 of Murinoglobulin-1 Mug1	sp|Q03626-2|MUG1_RAT (+2)	165 kDa	3.0462	5.2195	1.9411	1	1	1	0.067154208	3.402266667
Thiomorpholine-carboxylate dehydrogenase Crym	sp|Q9QYU4|CRYM_RAT	34 kDa	4.0616	4.1756	1.9411	1	1	1	0.030125026	3.392766667
Complement C4 C4	sp|P08649|CO4_RAT (+2)	192 kDa	3.0462	4.1756	2.9116	1	1	1	0.00404598	3.3778
Beta-galactosidase Glb1	tr|D3ZUM4|D3ZUM4_RAT	73 kDa	3.0462	4.1756	2.9116	1	1	1	0.00404598	3.3778
Protective protein for beta-galactosidase Ctsa	tr|Q6AYS3|Q6AYS3_RAT	51 kDa	4.0616	3.1317	2.9116	1	1	1	0.002553462	3.3683
Granulins Grn	sp|P23785|GRN_RAT (+2)	63 kDa	4.0616	3.1317	2.9116	1	1	1	0.002553462	3.3683
Isoform 2 of Basigin Bsg	sp|P26453-2|BASI_RAT (+1)	30 kDa	8.1232	8.3513	9.7053	1	4.7643	2.0419	0.007514104	3.353718839
Isoform 2 of Peroxisomal acyl-coenzyme A oxidase 1 Acox1	sp|P07872-2|ACOX1_RAT (+1)	75 kDa	3.0462	3.1317	3.8821	1	1	1	0.000895161	3.353333333
Stromal interaction molecule 1 Stim1	sp|P84903|STIM1_RAT	77 kDa	3.0462	3.1317	3.8821	1	1	1	0.000895161	3.353333333
Protein Cd2ap (Fragment) Cd2ap	tr|F1LRS8|F1LRS8_RAT	70 kDa	4.0616	2.0878	3.8821	1	1	1	0.020483806	3.343833333
Protein Cnpy3 Cnpy3	tr|G3V828|G3V828_RAT	31 kDa	5.077	5.2195	2.9116	2.0042	1	1	0.019959483	3.29856151
Peroxiredoxin-4 Prdx4	sp|Q9Z0V5|PRDX4_RAT	31 kDa	8.1232	7.3073	3.8821	2.0042	2.8586	1	0.033250518	3.29409156
Protein Tns3 Tns3	tr|F1LN91|F1LN91_RAT	156 kDa	6.0924	5.2195	4.8526	2.0042	1.9057	1	0.001530163	3.292225911
Transformer-2 protein homolog beta Tra2b	sp|P62997|TRA2B_RAT	34 kDa	2.0308	7.3073	3.8821	1	1	2.0419	0.125631628	3.270788491
Heterogeneous nuclear ribonucleoprotein H3 (2H9) (Predicted), isoform CRA_c Hnrnph3	tr|D4ABK7|D4ABK7_RAT	37 kDa	5.077	5.2195	2.9116	1	1	2.0419	0.020649425	3.267794849
Heterochromatin protein 1-binding protein 3 Hp1bp3	sp|Q6P747|HP1B3_RAT	61 kDa	11.169	12.527	7.7642	2.0042	5.7171	2.0419	0.018253221	3.222324648
Uncharacterized protein	tr|F1LTJ5|F1LTJ5_RAT	216 kDa	39.601	44.888	31.057	13.027	12.387	11.231	0.002936166	3.153117751
Ezrin Ezr	sp|P31977|EZRI_RAT	69 kDa	25.385	28.185	20.381	5.0104	9.5286	9.1887	0.003467571	3.116652689
Coronin (Fragment) Coro7	tr|D3ZUE2|D3ZUE2_RAT	83 kDa	5.077	4.1756	2.9116	1	1.9057	1	0.016809115	3.114473718
Protein Actn2 Actn2	tr|D3ZCV0|D3ZCV0_RAT	104 kDa	1	7.3073	1	1	1	1	0.373900966	3.102433333
Serine protease inhibitor A3K Serpina3k	sp|P05545|SPA3K_RAT	47 kDa	2.0308	6.2634	1	1	1	1	0.262574952	3.098066667
Non-POU domain-containing octamer-binding protein Nono	sp|Q5FVM4|NONO_RAT	55 kDa	11.169	13.571	8.7347	3.0063	4.7643	3.0629	0.007511065	3.08992477
Plexin B2 Plxnb2	tr|D3ZQ57|D3ZQ57_RAT	206 kDa	3.0462	5.2195	1	1	1	1	0.161605845	3.088566667
Selenium-binding protein 1 Selenbp1	sp|Q8VIF7|SBP1_RAT (+1)	53 kDa	3.0462	5.2195	1	1	1	1	0.161605845	3.088566667
Protein Synpo2 Synpo2	tr|D4A702|D4A702_RAT	136 kDa	3.0462	5.2195	1	1	1	1	0.161605845	3.088566667
Microfibrillar associated protein 5 (Predicted), isoform CRA_a Mfap5	tr|D3ZJB1|D3ZJB1_RAT	19 kDa	4.0616	4.1756	1	1	1	1	0.11624938	3.079066667
Vesicle-trafficking protein SEC22b Sec22b	sp|Q4KM74|SC22B_RAT	25 kDa	2.0308	5.2195	1.9411	1	1	1	0.12812658	3.0638
Tropomyosin 1, alpha, isoform CRA_c Tpm1	tr|F7FK40|F7FK40_RAT	33 kDa	12.185	18.79	12.617	1	1	12.252	0.086054621	3.058658434
Adenylate kinase 3 Ak3	tr|Q6P2A5|Q6P2A5_RAT	25 kDa	5.077	7.3073	8.7347	1	2.8586	3.0629	0.019354713	3.051217222
Catenin (Cadherin associated protein), delta 1 (Predicted), isoform CRA_a Ctnnd1	tr|D3ZZZ9|D3ZZZ9_RAT	104 kDa	7.1078	10.439	9.7053	2.0042	2.8586	4.0839	0.006588592	3.046050499
Protein LOC687575 LOC687575	tr|Q3KR55|Q3KR55_RAT	28 kDa	2.0308	4.1756	2.9116	1	1	1	0.0306049	3.039333333
Histone H1.1 Hist1h1a	sp|D4A3K5|H11_RAT	22 kDa	3.0462	3.1317	2.9116	1	1	1	5.91343E-06	3.029833333
40S ribosomal protein S27-like Rps27l	sp|P24051|RS27L_RAT	9 kDa	3.0462	3.1317	2.9116	1	1	1	5.91343E-06	3.029833333
Microsomal glutathione S-transferase 1 Mgst1	sp|P08011|MGST1_RAT	17 kDa	3.0462	3.1317	2.9116	1	1	1	5.91343E-06	3.029833333
Glutamate carboxypeptidase 2 Folh1	sp|P70627|FOLH1_RAT (+1)	85 kDa	3.0462	3.1317	2.9116	1	1	1	5.91343E-06	3.029833333
UBX domain-containing protein 4 Ubxn4	sp|Q5HZY0|UBXN4_RAT	56 kDa	3.0462	3.1317	2.9116	1	1	1	5.91343E-06	3.029833333
Proteasome activator complex subunit 2 Psme2	sp|Q63798|PSME2_RAT	27 kDa	3.0462	3.1317	2.9116	1	1	1	5.91343E-06	3.029833333
Protein Cenpv Cenpv	tr|D4A9A3|D4A9A3_RAT	27 kDa	3.0462	3.1317	2.9116	1	1	1	5.91343E-06	3.029833333
Tripeptidyl-peptidase 1 Tpp1	sp|Q9EQV6|TPP1_RAT (+1)	61 kDa	3.0462	3.1317	2.9116	1	1	1	5.91343E-06	3.029833333
Translocon-associated protein subunit delta Ssr4	sp|Q07984|SSRD_RAT	19 kDa	3.0462	3.1317	2.9116	1	1	1	5.91343E-06	3.029833333
IST1 homolog Ist1	sp|Q568Z6|IST1_RAT	40 kDa	4.0616	2.0878	2.9116	1	1	1	0.024236279	3.020333333
Legumain Lgmn	sp|Q9R0J8|LGMN_RAT (+1)	49 kDa	4.0616	2.0878	2.9116	1	1	1	0.024236279	3.020333333
Pyridine nucleotide-disulfide oxidoreductase domain-containing protein 2 Pyroxd2	sp|Q68FT3|PYRD2_RAT	63 kDa	2.0308	3.1317	3.8821	1	1	1	0.019984514	3.014866667
Heterogeneous nuclear ribonucleoprotein R Hnrnpr	tr|Q566E4|Q566E4_RAT	71 kDa	8.1232	7.3073	5.8232	3.0063	1	3.0629	0.007777413	3.006521247
Chloride intracellular channel protein 1 Clic1	sp|Q6MG61|CLIC1_RAT	27 kDa	3.0462	2.0878	3.8821	1	1	1	0.018015134	3.005366667
Protein Pgm5 Pgm5	tr|D3ZVR9|D3ZVR9_RAT	62 kDa	2.0308	2.0878	4.8526	1	1	1	0.099382959	2.9904
NADH-cytochrome b5 reductase 1 Cyb5r1	sp|Q5EB81|NB5R1_RAT (+1)	34 kDa	3.0462	4.1756	4.8526	1	1	2.0419	0.013232657	2.987307949
B-cell receptor-associated protein 31 Bcap31	tr|Q6AY58|Q6AY58_RAT	28 kDa	4.0616	1	3.8821	1	1	1	0.116479162	2.981233333
Polymerase I and transcript release factor Ptrf	tr|G3V8L9|G3V8L9_RAT (+1)	44 kDa	16.246	18.79	11.646	6.0125	4.7643	5.1048	0.008431542	2.939376385
Vimentin Vim	sp|P31000|VIME_RAT	54 kDa	153.33	176.42	143.64	51.106	53.36	57.174	0.000462562	2.928668646
Protein Tgfbi Tgfbi	tr|D4A8G5|D4A8G5_RAT	75 kDa	23.354	27.142	12.617	8.0167	9.5286	4.0839	0.040805725	2.917953507
Xaa-Pro dipeptidase Pepd	sp|Q5I0D7|PEPD_RAT	55 kDa	10.154	7.3073	8.7347	3.0063	1.9057	4.0839	0.005187697	2.911993241
Eukaryotic translation initiation factor 2 subunit 3 Eif2s3	sp|P81795|IF2G_RAT	51 kDa	6.0924	8.3513	5.8232	2.0042	1.9057	3.0629	0.007394524	2.906565512
60S ribosomal protein L12 Rpl12	sp|P23358|RL12_RAT	18 kDa	5.077	6.2634	5.8232	1	1.9057	3.0629	0.005665099	2.875649231
Lumican Lum	sp|P51886|LUM_RAT	38 kDa	19.293	24.01	16.499	7.0146	6.67	7.1467	0.004092416	2.870776188
Heterogeneous nuclear ribonucleoprotein F Hnrnpf	sp|Q794E4|HNRPF_RAT	46 kDa	6.0924	8.3513	5.8232	3.0063	1	3.0629	0.013815672	2.86692978
gi|113580|sp|P02770| ALBU_RAT SERUM ALBUMIN PRECURSOR.	zz|ZZ_FGCZCont0020|	69 kDa	50.77	60.547	33.968	16.033	13.34	21.44	0.017859706	2.859209257
Acidic leucine-rich nuclear phosphoprotein 32 family member E Anp32e	sp|Q5XIE0|AN32E_RAT	29 kDa	4.0616	4.1756	2.9116	1	1.9057	1	0.008713917	2.854494713
Protein Tmod3 Tmod3	tr|Q6AXW2|Q6AXW2_RAT	39 kDa	4.0616	4.1756	2.9116	1	1.9057	1	0.008713917	2.854494713
Dolichyl-diphosphooligosaccharide--protein glycosyltransferase subunit 1 Rpn1	tr|Q6P7A7|Q6P7A7_RAT	68 kDa	7.1078	9.3952	5.8232	2.0042	3.8114	2.0419	0.015999196	2.84138721
Vinculin Vcl	sp|P85972|VINC_RAT	117 kDa	30.462	37.581	27.175	10.021	13.34	10.21	0.00321689	2.836317059
Sorbin and SH3 domain-containing protein 1 (Fragment) Sorbs1	tr|F1M865|F1M865_RAT	83 kDa	16.246	17.746	16.499	7.0146	5.7171	5.1048	0.000117405	2.830768368
Lamin-B1 Lmnb1	tr|G3V7U4|G3V7U4_RAT	67 kDa	25.385	28.185	21.352	8.0167	6.67	12.252	0.003543668	2.78120325
NAD(P)H dehydrogenase [quinone] 1 Nqo1	sp|P05982|NQO1_RAT	31 kDa	6.0924	6.2634	6.7937	2.0042	2.8586	2.0419	0.00030793	2.773400727
Alpha-1-antiproteinase Serpina1	sp|P17475|A1AT_RAT	46 kDa	1	6.2634	1	1	1	1	0.373900966	2.754466667

**Protein Krt31 Krt33a**	tr|F6SF42|F6SF42_RAT	46 kDa	1	6.2634	1	1	1	1	0.373900966	2.754466667
**Isoform Gamma-A of Fibrinogen gamma chain Fgg**	sp|P02680-2|FIBG_RAT (+1)	50 kDa	2.0308	5.2195	1	1	1	1	0.240275239	2.7501
**Ig kappa chain C region, A allele**	sp|P01836|KACA_RAT	12 kDa	3.0462	4.1756	1	1	1	1	0.134380626	2.7406
**Uncharacterized protein (Fragment)**	tr|D4A7W8|D4A7W8_RAT (+1)	29 kDa	4.0616	3.1317	1	1	1	1	0.128710215	2.7311
**Protein DEK Dek**	sp|Q6AXS3|DEK_RAT	43 kDa	1	5.2195	1.9411	1	1	1	0.249785096	2.7202
**Lipocalin 7, isoform CRA_a Tinagl1**	tr|Q4V8N0|Q4V8N0_RAT	53 kDa	8.1232	11.483	4.8526	3.0063	1.9057	4.0839	0.062750322	2.718883047
**LDLR chaperone MESD Mesdc2**	sp|Q5U2R7|MESD_RAT	25 kDa	2.0308	4.1756	1.9411	1	1	1	0.078570749	2.715833333
**Protein Sf3b2 Sf3b2**	tr|D3ZMS1|D3ZMS1_RAT	98 kDa	3.0462	3.1317	1.9411	1	1	1	0.011243318	2.706333333
**Protein G3bp1 G3bp1**	tr|D3ZYS7|D3ZYS7_RAT	52 kDa	3.0462	3.1317	1.9411	1	1	1	0.011243318	2.706333333
**Uncharacterized protein (Fragment)**	tr|M0RBW1|M0RBW1_RAT (+1)	109 kDa	3.0462	3.1317	1.9411	1	1	1	0.011243318	2.706333333
**Nucleolar RNA helicase 2 Ddx21**	sp|Q3B8Q1|DDX21_RAT (+1)	86 kDa	3.0462	3.1317	1.9411	1	1	1	0.011243318	2.706333333
**Transmembrane emp24 domain-containing protein 9 Tmed9**	sp|Q5I0E7|TMED9_RAT	27 kDa	3.0462	3.1317	1.9411	1	1	1	0.011243318	2.706333333
**Mitochondrial pyruvate carrier 2 Mpc2**	sp|P38718|MPC2_RAT	14 kDa	2.0308	3.1317	2.9116	1	1	1	0.007339938	2.691366667
**Coatomer subunit beta' Copb2**	sp|O35142|COPB2_RAT	103 kDa	3.0462	2.0878	2.9116	1	1	1	0.00494598	2.681866667
**Serine protease HTRA1 Htra1**	sp|Q9QZK5|HTRA1_RAT	51 kDa	3.0462	2.0878	2.9116	1	1	1	0.00494598	2.681866667
**5-oxoprolinase Oplah**	sp|P97608|OPLA_RAT	138 kDa	3.0462	2.0878	2.9116	1	1	1	0.00494598	2.681866667
**Adipose differentiation related protein Plin2**	tr|Q5U2U5|Q5U2U5_RAT	46 kDa	3.0462	2.0878	2.9116	1	1	1	0.00494598	2.681866667
**DEAH (Asp-Glu-Ala-His) box polypeptide 9 (Predicted) Dhx9**	tr|D4A9D6|D4A9D6_RAT	132 kDa	5.077	7.3073	3.8821	2.0042	1	3.0629	0.043548699	2.681083219
**Extended synaptotagmin-1 Esyt1**	sp|Q9Z1X1|ESYT1_RAT	121 kDa	5.077	5.2195	2.9116	1	1.9057	2.0419	0.027837899	2.669597381
**Protein Hnrnpa0 Hnrnpa0**	tr|F1M3H8|F1M3H8_RAT	30 kDa	5.077	7.3073	5.8232	3.0063	2.8586	1	0.014736271	2.652260047
**Calponin-3 Cnn3**	sp|P37397|CNN3_RAT	36 kDa	11.169	10.439	12.617	5.0104	3.8114	4.0839	0.000642718	2.651928993
**Adaptor protein, phosphotyrosine interaction, PH domain and leucine zipper containing 2 Appl2**	tr|B4F779|B4F779_RAT	74 kDa	3.0462	1	3.8821	1	1	1	0.127431639	2.642766667
**Cathepsin B Ctsb**	sp|P00787|CATB_RAT (+1)	37 kDa	12.185	15.659	13.587	5.0104	6.67	4.0839	0.002464707	2.628153486
**Laminin subunit beta-2 Lamb2**	tr|M0R6K0|M0R6K0_RAT (+1)	197 kDa	25.385	33.405	21.352	12.025	10.481	8.1677	0.011350253	2.612726864
**Protein disulfide-isomerase A4 Pdia4**	sp|P38659|PDIA4_RAT	73 kDa	13.2	14.615	10.676	5.0104	5.7171	4.0839	0.003172427	2.59874151
**Protein Amt Amt**	tr|M0R9I6|M0R9I6_RAT	45 kDa	3.0462	4.1756	2.9116	1	1.9057	1	0.014409945	2.594515708
**Nicotinamide phosphoribosyltransferase Nampt**	sp|Q80Z29|NAMPT_RAT	55 kDa	4.0616	3.1317	5.8232	2.0042	1	2.0419	0.036577324	2.579516855
**Spliceosome RNA helicase Ddx39b Ddx39b**	sp|Q63413|DX39B_RAT	49 kDa	6.0924	8.3513	5.8232	2.0042	2.8586	3.0629	0.008922455	2.557111675
**Phosphoribosylglycinamide formyltransferase, isoform CRA_a Gart**	tr|G3V918|G3V918_RAT	108 kDa	9.1386	7.3073	8.7347	3.0063	3.8114	3.0629	0.001140915	2.548488958
**Heterogeneous nuclear ribonucleoprotein U Hnrnpu**	tr|Q6IMY8|Q6IMY8_RAT	88 kDa	18.277	24.01	15.528	8.0167	6.67	8.1677	0.010170298	2.529709815
**Histone H3.1**	sp|Q6LED0|H31_RAT	15 kDa	1	8.3513	5.8232	4.0083	1	1	0.268212965	2.525589601
**Protein Snrnp70 Snrnp70**	tr|B2RZ74|B2RZ74_RAT	52 kDa	3.0462	5.2195	1.9411	1	1	2.0419	0.115142356	2.525248027
**Adducin 3 (Gamma), isoform CRA_b Add3**	tr|D3ZCH7|D3ZCH7_RAT	75 kDa	16.246	15.659	12.617	5.0104	5.7171	7.1467	0.002319978	2.490852737
**DNA-(apurinic or apyrimidinic site) lyase Apex1**	sp|P43138|APEX1_RAT	36 kDa	3.0462	3.1317	3.8821	1	1	2.0419	0.010117578	2.488928474
**Prolargin Prelp**	sp|Q9EQP5|PRELP_RAT	43 kDa	33.508	43.844	30.086	14.029	16.199	14.293	0.007471745	2.413198266
**DnaJ (Hsp40) homolog, subfamily B, member 4 Dnajb4**	tr|Q5XIP0|Q5XIP0_RAT	38 kDa	3.0462	4.1756	4.8526	3.0063	1	1	0.050470791	2.41184108
**Creatine kinase S-type, mitochondrial Ckmt2**	sp|P09605|KCRS_RAT	47 kDa	1	5.2195	1	1	1	1	0.373900966	2.4065
**Protein Ccar1 Ccar1**	tr|D4A2P1|D4A2P1_RAT	132 kDa	1	5.2195	1	1	1	1	0.373900966	2.4065
**Plasminogen Plg**	sp|Q01177|PLMN_RAT	91 kDa	1	5.2195	1	1	1	1	0.373900966	2.4065
**Carbonic anhydrase 3 Ca3**	sp|P14141|CAH3_RAT	29 kDa	1	5.2195	1	1	1	1	0.373900966	2.4065
**Protein Serpinc1 Serpinc1**	tr|Q5M7T5|Q5M7T5_RAT	52 kDa	1	5.2195	1	1	1	1	0.373900966	2.4065
**Scaffold attachment factor B1 Safb**	sp|O88453|SAFB1_RAT (+1)	105 kDa	2.0308	4.1756	1	1	1	1	0.208226427	2.402133333
**Uncharacterized protein**	tr|E9PU73|E9PU73_RAT	126 kDa	2.0308	4.1756	1	1	1	1	0.208226427	2.402133333
**Structural maintenance of chromosomes protein 3 Smc3**	sp|P97690|SMC3_RAT (+2)	138 kDa	2.0308	4.1756	1	1	1	1	0.208226427	2.402133333
**Serum deprivation-response protein Sdpr**	sp|Q66H98|SDPR_RAT	46 kDa	2.0308	4.1756	1	1	1	1	0.208226427	2.402133333
**Sorting nexin-5 Snx5**	sp|B1H267|SNX5_RAT	47 kDa	10.154	8.3513	7.7642	4.0083	2.8586	4.0839	0.003402998	2.398865836
**Moesin (Fragment) Msn**	tr|F1LP60|F1LP60_RAT	68 kDa	41.631	43.844	37.85	15.031	19.057	17.356	0.000338681	2.397266931
**Transmembrane emp24 domain-containing protein 2 Tmed2**	sp|Q63524|TMED2_RAT	23 kDa	3.0462	3.1317	1	1	1	1	0.116283078	2.392633333
**Signal peptidase complex catalytic subunit SEC11A Sec11a**	sp|P42667|SC11A_RAT (+1)	21 kDa	3.0462	3.1317	1	1	1	1	0.116283078	2.392633333
**Annexin Anxa4**	tr|Q5U362|Q5U362_RAT	36 kDa	7.1078	9.3952	6.7937	4.0083	4.7643	1	0.033135731	2.383879418
**40S ribosomal protein S23 Rps23**	sp|P62268|RS23_RAT	16 kDa	7.1078	8.3513	5.8232	3.0063	2.8586	3.0629	0.004918309	2.383823562
**Hemopexin Hpx**	sp|P20059|HEMO_RAT	51 kDa	4.0616	2.0878	1	1	1	1	0.197567114	2.383133333
**Protein Abi3bp Abi3bp**	tr|F1M0R2|F1M0R2_RAT	129 kDa	4.0616	2.0878	1	1	1	1	0.197567114	2.383133333
**Isoform 2 of Interleukin enhancer-binding factor 3 Ilf3**	sp|Q9JIL3-2|ILF3_RAT (+2)	98 kDa	5.077	2.0878	4.8526	2.0042	1	2.0419	0.08494337	2.381522364
**Protein disulfide-isomerase A6 Pdia6**	sp|Q63081|PDIA6_RAT	48 kDa	13.2	15.659	8.7347	6.0125	5.7171	4.0839	0.026413038	2.37731685
**Isoform 2 of Calpastatin Cast**	sp|P27321-2|ICAL_RAT (+2)	73 kDa	3.0462	5.2195	1	1	1.9057	1	0.227678917	2.372353227
**Putative RNA-binding protein 3 Rbm3**	tr|G3V6P6|G3V6P6_RAT	17 kDa	1	4.1756	1.9411	1	1	1	0.218804268	2.372233333
**GM2 ganglioside activator Gm2a**	tr|Q6IN37|Q6IN37_RAT	21 kDa	2.0308	3.1317	1.9411	1	1	1	0.023305165	2.367866667
**EH domain-containing protein 2 Ehd2**	sp|Q4V8H8|EHD2_RAT	61 kDa	2.0308	3.1317	1.9411	1	1	1	0.023305165	2.367866667
**Protein Sephs1 Sephs1**	tr|D3ZFY0|D3ZFY0_RAT	43 kDa	2.0308	3.1317	1.9411	1	1	1	0.023305165	2.367866667
**Eukaryotic translation initiation factor 3 subunit G Eif3g**	sp|Q5RK09|EIF3G_RAT	36 kDa	2.0308	3.1317	1.9411	1	1	1	0.023305165	2.367866667
**Protein Itgb2 Itgb2**	tr|F7F4S8|F7F4S8_RAT	85 kDa	2.0308	3.1317	1.9411	1	1	1	0.023305165	2.367866667
**Collagen alpha-1(V) chain Col5a1**	tr|G3V763|G3V763_RAT (+1)	162 kDa	2.0308	3.1317	1.9411	1	1	1	0.023305165	2.367866667
**3-hydroxybutyrate dehydrogenase type 2 Bdh2**	sp|D4A1J4|BDH2_RAT	27 kDa	2.0308	3.1317	1.9411	1	1	1	0.023305165	2.367866667
**Epithelial protein lost in neoplasm Lima1**	tr|F1LR10|F1LR10_RAT	84 kDa	2.0308	3.1317	1.9411	1	1	1	0.023305165	2.367866667
**Protein Plin3 Plin3**	tr|M0RA08|M0RA08_RAT	47 kDa	2.0308	3.1317	1.9411	1	1	1	0.023305165	2.367866667
**Cathepsin D Ctsd**	sp|P24268|CATD_RAT (+1)	45 kDa	14.216	12.527	17.469	6.0125	7.6229	5.1048	0.006426062	2.359206412
**Cd99 protein Cd99**	tr|B4F7A5|B4F7A5_RAT	17 kDa	3.0462	2.0878	1.9411	1	1	1	0.017246383	2.358366667
**Protein RGD1309922 RGD1309922**	tr|F1LM55|F1LM55_RAT	103 kDa	3.0462	2.0878	1.9411	1	1	1	0.017246383	2.358366667
**Fragile X mental retardation syndrome-related protein 1 Fxr1**	sp|Q5XI81|FXR1_RAT (+1)	64 kDa	3.0462	2.0878	1.9411	1	1	1	0.017246383	2.358366667
**Protein Fcrls (Fragment) Fcrls**	tr|F1M652|F1M652_RAT	54 kDa	3.0462	2.0878	1.9411	1	1	1	0.017246383	2.358366667
**Pleckstrin Plek**	sp|Q4KM33|PLEK_RAT	40 kDa	3.0462	2.0878	1.9411	1	1	1	0.017246383	2.358366667
**Adaptin ear-binding coat-associated protein 2 Necap2**	sp|Q6P756|NECP2_RAT	28 kDa	3.0462	2.0878	1.9411	1	1	1	0.017246383	2.358366667
**Protein LOC682967 LOC682967**	tr|M0R402|M0R402_RAT	52 kDa	3.0462	2.0878	1.9411	1	1	1	0.017246383	2.358366667
**Protein Myl6b LOC684533**	tr|D3ZHA7|D3ZHA7_RAT	23 kDa	3.0462	2.0878	1.9411	1	1	1	0.017246383	2.358366667
**gi|88041|pir||A31994 keratin 10, type I, epidermal - human gi|623409 (**	zz|ZZ_FGCZCont0158|	57 kDa	1	14.615	15.528	1	1	11.231	0.36183934	2.353790341
**Epididymal secretory protein 1 Npc2**	tr|Q8CHN5|Q8CHN5_RAT	16 kDa	2.0308	2.0878	2.9116	1	1	1	0.009166802	2.3434
**Isoform 2 of Poly(U)-binding-splicing factor PUF60 Puf60**	sp|Q9WV25-2|PUF60_RAT (+1)	59 kDa	2.0308	2.0878	2.9116	1	1	1	0.009166802	2.3434
**Aldose 1-epimerase Galm**	sp|Q66HG4|GALM_RAT	38 kDa	2.0308	2.0878	2.9116	1	1	1	0.009166802	2.3434
**Protein Aldh18a1 Aldh18a1**	tr|D3ZIE9|D3ZIE9_RAT	87 kDa	2.0308	2.0878	2.9116	1	1	1	0.009166802	2.3434
**Lipid phosphate phosphohydrolase 3 Ppap2b**	sp|P97544|LPP3_RAT (+1)	35 kDa	2.0308	2.0878	2.9116	1	1	1	0.009166802	2.3434
**Uncharacterized protein Sdsl**	tr|D3ZHV7|D3ZHV7_RAT	38 kDa	2.0308	2.0878	2.9116	1	1	1	0.009166802	2.3434
**Lysosome-associated membrane glycoprotein 2 Lamp2**	sp|P17046|LAMP2_RAT (+1)	45 kDa	2.0308	2.0878	2.9116	1	1	1	0.009166802	2.3434
**Protein Tmem65 Tmem65**	tr|F1LQZ0|F1LQZ0_RAT	28 kDa	2.0308	2.0878	2.9116	1	1	1	0.009166802	2.3434
**E3 UFM1-protein ligase 1 Ufl1**	sp|B2GV24|UFL1_RAT	90 kDa	2.0308	2.0878	2.9116	1	1	1	0.009166802	2.3434
**Uncharacterized protein**	tr|E9PT22|E9PT22_RAT (+1)	139 kDa	2.0308	2.0878	2.9116	1	1	1	0.009166802	2.3434
**Annexin Anxa11**	tr|Q5XI77|Q5XI77_RAT	54 kDa	7.1078	8.3513	7.7642	3.0063	2.8586	4.0839	0.001104061	2.334281521
**Ornithine aminotransferase, mitochondrial Oat**	sp|P04182|OAT_RAT	48 kDa	9.1386	9.3952	6.7937	4.0083	3.8114	3.0629	0.005348788	2.327339055
**Actin-related protein 2/3 complex subunit 5 Arpc5**	sp|Q4KLF8|ARPC5_RAT	16 kDa	3.0462	3.1317	2.9116	1	1.9057	1	0.004995808	2.327239675
**Uncharacterized protein (Fragment)**	tr|M0R735|M0R735_RAT	17 kDa	2.0308	6.2634	1	2.0042	1	1	0.344069445	2.321112832
**Dual specificity mitogen-activated protein kinase kinase 2 Map2k2**	sp|P36506|MP2K2_RAT	44 kDa	3.0462	1	2.9116	1	1	1	0.116576358	2.319266667
**Protein Pacsin3 Pacsin3**	tr|G3V9N7|G3V9N7_RAT	49 kDa	3.0462	1	2.9116	1	1	1	0.116576358	2.319266667
**Neurabin-2 Ppp1r9b**	sp|O35274|NEB2_RAT	90 kDa	7.1078	5.2195	5.8232	2.0042	3.8114	2.0419	0.013632009	2.309958638
**Isoform III of Cystathionine beta-synthase Cbs**	sp|P32232-2|CBS_RAT (+1)	60 kDa	2.0308	1	3.8821	1	1	1	0.196786239	2.3043
**Hemoglobin subunit beta-1 Hbb**	sp|P02091|HBB1_RAT	16 kDa	15.231	12.527	8.7347	6.0125	4.7643	5.1048	0.02322366	2.297797451
**Protein Rdx Rdx**	tr|E9PT65|E9PT65_RAT	68 kDa	23.354	24.01	22.322	10.021	14.293	6.1258	0.005579129	2.289305449
**Protein Tns1 Tns1**	tr|F1LN42|F1LN42_RAT	204 kDa	4.0616	4.1756	1	1	1	2.0419	0.189403322	2.285360845
**Isoform ZE of Plasma membrane calcium-transporting ATPase 3 Atp2b3**	sp|Q64568-10|AT2B3_RAT (+8)	127 kDa	1	1	4.8526	1	1	1	0.373900966	2.2842
**Isoform XA of Plasma membrane calcium-transporting ATPase 4 Atp2b4**	sp|Q64542-2|AT2B4_RAT (+6)	129 kDa	1	1	4.8526	1	1	1	0.373900966	2.2842
**3-ketoacyl-CoA thiolase A, peroxisomal Acaa1a**	sp|P21775|THIKA_RAT	44 kDa	6.0924	6.2634	5.8232	3.0063	1.9057	3.0629	0.001027556	2.279527016
**Protein Suclg2 Suclg2**	tr|F1LPV8|F1LPV8_RAT	47 kDa	6.0924	6.2634	5.8232	3.0063	1.9057	3.0629	0.001027556	2.279527016
**Eukaryotic translation initiation factor 3 subunit I Eif3i**	sp|B0BNA7|EIF3I_RAT	36 kDa	2.0308	4.1756	2.9116	2.0042	1	1	0.073400372	2.277109036
**40S ribosomal protein S7 Rps7**	sp|P62083|RS7_RAT	22 kDa	11.169	11.483	8.7347	5.0104	5.7171	3.0629	0.007567674	2.275981842
**Protein LOC100909752 (Fragment) LOC100909752**	tr|F1LPD0|F1LPD0_RAT	137 kDa	5.077	9.3952	5.8232	3.0063	2.8586	3.0629	0.04683982	2.273281211
**Heterogeneous nuclear ribonucleoprotein M Hnrnpm**	tr|F1LV13|F1LV13_RAT (+1)	74 kDa	17.262	22.966	13.587	7.0146	8.5757	8.1677	0.022372593	2.265131745
**Nidogen-1 Nid1**	tr|F1LM84|F1LM84_RAT	137 kDa	37.57	42.8	25.234	17.035	13.34	16.335	0.021134679	2.260843502
**RuvB-like 1 Ruvbl1**	sp|P60123|RUVB1_RAT	50 kDa	8.1232	9.3952	4.8526	2.0042	3.8114	4.0839	0.05045986	2.259811102
**Beta-2-microglobulin B2m**	sp|P07151|B2MG_RAT	14 kDa	4.0616	3.1317	3.8821	2.0042	1.9057	1	0.008642066	2.255728223
**60S ribosomal protein L6 Rpl6**	sp|P21533|RL6_RAT (+2)	34 kDa	7.1078	9.3952	5.8232	2.0042	3.8114	4.0839	0.028224735	2.25528562
**Isoform 3 of Agrin Agrn**	sp|P25304-3|AGRIN_RAT (+9)	207 kDa	8.1232	9.3952	4.8526	3.0063	2.8586	4.0839	0.042265995	2.248612898
**Transgelin-2 Tagln2**	sp|Q5XFX0|TAGL2_RAT	22 kDa	15.231	16.703	12.617	6.0125	5.7171	8.1677	0.004460652	2.239047509
**Protein Tgm2 Tgm2**	tr|Q9WVJ6|Q9WVJ6_RAT	77 kDa	19.293	19.834	16.499	8.0167	6.67	10.21	0.00217454	2.234272012
**40S ribosomal protein S9 Rps9**	sp|P29314|RS9_RAT	23 kDa	7.1078	5.2195	2.9116	3.0063	2.8586	1	0.11209211	2.219828402
**Uncharacterized protein**	tr|D3ZZR3|D3ZZR3_RAT (+1)	36 kDa	5.077	4.1756	3.8821	2.0042	1.9057	2.0419	0.002703401	2.206844988
**Cytosolic 10-formyltetrahydrofolate dehydrogenase Aldh1l1**	sp|P28037|AL1L1_RAT	99 kDa	24.37	20.878	24.263	9.0188	13.34	9.1887	0.002246397	2.203375862
**EH-domain containing 4 Ehd4**	tr|Q8R3Z7|Q8R3Z7_RAT	61 kDa	6.0924	5.2195	3.8821	1	2.8586	3.0629	0.039864653	2.195188904
**Isoform 2 of NADH-cytochrome b5 reductase 3 Cyb5r3**	sp|P20070-2|NB5R3_RAT (+2)	35 kDa	11.169	10.439	10.676	2.0042	6.67	6.1258	0.017318434	2.181351351
**Enoyl-CoA delta isomerase 1, mitochondrial Eci1**	sp|P23965|ECI1_RAT (+1)	32 kDa	6.0924	6.2634	4.8526	2.0042	2.8586	3.0629	0.004916459	2.171215161
**Clusterin Clu**	sp|P05371|CLUS_RAT (+1)	51 kDa	12.185	12.527	11.646	6.0125	6.67	4.0839	0.001329917	2.168503674
**Protein Aimp1 Aimp1**	tr|Q4G079|Q4G079_RAT	35 kDa	4.0616	5.2195	5.8232	2.0042	1.9057	3.0629	0.013026336	2.166174277
**Estradiol 17-beta-dehydrogenase 12 Hsd17b12**	sp|Q6P7R8|DHB12_RAT (+1)	35 kDa	3.0462	5.2195	6.7937	1	1.9057	4.0839	0.131194704	2.154543894
**Glycine amidinotransferase, mitochondrial Gatm**	sp|P50442|GATM_RAT	48 kDa	6.0924	4.1756	6.7937	2.0042	2.8586	3.0629	0.022849758	2.152705755

**Eukaryotic initiation factor 4A-III Eif4a3**	sp|Q3B8Q2|IF4A3_RAT	47 kDa	3.0462	7.3073	4.8526	3.0063	1	3.0629	0.126522377	2.151035478
**Creatine kinase M-type Ckm**	sp|P00564|KCRM_RAT	43 kDa	5.077	9.3952	2.9116	2.0042	1	5.1048	0.245068402	2.143766186
**40S ribosomal protein S18 Rps18**	sp|P62271|RS18_RAT (+1)	18 kDa	7.1078	6.2634	7.7642	3.0063	3.8114	3.0629	0.00176539	2.139080623
**Protein Txndc5 Txndc5**	tr|D3ZZC1|D3ZZC1_RAT	46 kDa	10.154	8.3513	6.7937	4.0083	3.8114	4.0839	0.010160549	2.125323432
**Collagen alpha-1(I) chain Col1a1**	sp|P02454|CO1A1_RAT	138 kDa	27.416	39.668	27.175	18.038	14.293	12.252	0.020592628	2.114236368
**Decorin Dcn**	sp|Q01129|PGS2_RAT	40 kDa	29.447	30.273	24.263	15.031	11.434	13.273	0.002358458	2.113417887
**Hypoxia up-regulated protein 1 Hyou1**	tr|F1LN18|F1LN18_RAT	111 kDa	12.185	20.878	8.7347	5.0104	7.6229	7.1467	0.11838634	2.113129424
**Eukaryotic translation initiation factor 3 subunit B Eif3b**	sp|Q4G061|EIF3B_RAT	91 kDa	3.0462	4.1756	1	1	1.9057	1	0.214918447	2.105077195
**Uncharacterized protein Vdac1**	tr|F1M2D3|F1M2D3_RAT	31 kDa	12.185	12.527	11.646	1	1	15.314	0.254729323	2.099919141
**Nucleoside diphosphate kinase Nme3**	tr|G3V816|G3V816_RAT	19 kDa	4.0616	3.1317	1	1	1.9057	1	0.208922837	2.097780167
**Protein Prkcsh Prkcsh**	tr|B1WC34|B1WC34_RAT	59 kDa	12.185	12.527	10.676	5.0104	4.7643	7.1467	0.002882598	2.091316321
**Protein Lrrc47 Lrrc47**	tr|F1LT49|F1LT49_RAT	64 kDa	2.0308	4.1756	1.9411	1	1.9057	1	0.148096633	2.086053716
**Protein Lamc1 Lamc1**	tr|F1MAA7|F1MAA7_RAT	177 kDa	28.431	34.449	30.086	14.029	14.293	16.335	0.001144	2.081778892
**Isoform RC6-IS of Proteasome subunit alpha type-7 Psma7**	sp|P48004-2|PSA7_RAT	28 kDa	9.1386	8.3513	9.7053	7.0146	1	5.1048	0.061210035	2.072899675
**Collagen alpha-2(I) chain Col1a2**	sp|P02466|CO1A2_RAT (+1)	130 kDa	17.262	18.79	17.469	9.0188	7.6229	9.1887	0.000179846	2.072015919
**Protein CDV3 homolog Cdv3**	sp|Q5XIM5|CDV3_RAT	24 kDa	4.0616	2.0878	1.9411	1	1.9057	1	0.135390811	2.071459662
**Phosphoribosyl transferase domain containing 1 Prtfdc1**	tr|B2RYS6|B2RYS6_RAT	26 kDa	2.0308	3.1317	2.9116	1	1.9057	1	0.037136549	2.06726067
**Annexin A2 Anxa2**	sp|Q07936|ANXA2_RAT	39 kDa	25.385	33.405	23.293	12.025	12.387	15.314	0.012248848	2.066228666
**Uncharacterized protein Rsu1**	tr|D4A8F2|D4A8F2_RAT	29 kDa	3.0462	4.1756	2.9116	2.0042	1.9057	1	0.027360522	2.063870955
**Acid ceramidase Asah1**	sp|Q6P7S1|ASAH1_RAT	44 kDa	7.1078	7.3073	3.8821	2.0042	3.8114	3.0629	0.062798902	2.060843611
**Isoform SERCA2A of Sarcoplasmic/endoplasmic reticulum calcium ATPase 2 Atp2a2**	sp|P11507-2|AT2A2_RAT (+3)	110 kDa	6.0924	6.2634	7.7642	2.0042	5.7171	2.0419	0.061695652	2.060799738
**Polypyrimidine tract-binding protein 3 Ptbp3**	sp|Q9Z118|PTBP3_RAT	57 kDa	1	4.1756	1	1	1	1	0.373900966	2.058533333
**Protein Spag9 Spag9**	tr|E9PSJ4|E9PSJ4_RAT	146 kDa	1	4.1756	1	1	1	1	0.373900966	2.058533333
**Protein LOC299282 LOC299282**	tr|F1LM05|F1LM05_RAT	68 kDa	1	4.1756	1	1	1	1	0.373900966	2.058533333
**Glutathione peroxidase 3 Gpx3**	sp|P23764|GPX3_RAT	25 kDa	1	4.1756	1	1	1	1	0.373900966	2.058533333
**Isoform 2 of Troponin T, fast skeletal muscle Tnnt3**	sp|P09739-2|TNNT3_RAT	31 kDa	1	4.1756	1	1	1	1	0.373900966	2.058533333
**Protein Krt31 Krt34**	tr|F7EM03|F7EM03_RAT	44 kDa	1	4.1756	1	1	1	1	0.373900966	2.058533333
**Troponin I, fast skeletal muscle Tnni2**	sp|P27768|TNNI2_RAT (+1)	21 kDa	1	4.1756	1	1	1	1	0.373900966	2.058533333
**Cellular retinoic acid-binding protein 2 Crabp2**	sp|P51673|RABP2_RAT	16 kDa	1	4.1756	1	1	1	1	0.373900966	2.058533333
**Elastin microfibril interfacer 1 (Predicted), isoform CRA_b Emilin1**	tr|D3Z9E1|D3Z9E1_RAT	95 kDa	1	4.1756	1	1	1	1	0.373900966	2.058533333
**Isoform 2 of DNA-binding protein A Csda**	sp|Q62764-2|DBPA_RAT (+1)	31 kDa	1	4.1756	1	1	1	1	0.373900966	2.058533333
**Aldehyde dehydrogenase, dimeric NADP-preferring Aldh3a1**	sp|P11883|AL3A1_RAT	50 kDa	1	4.1756	1	1	1	1	0.373900966	2.058533333
**Histone H2A H2afy2**	tr|B1WC28|B1WC28_RAT	40 kDa	1	4.1756	1	1	1	1	0.373900966	2.058533333
**Protein Tjp2 Tjp2**	tr|Q3ZB99|Q3ZB99_RAT	131 kDa	18.277	14.615	13.587	7.0146	10.481	5.1048	0.019921703	2.056556521
**Phenylalanine--tRNA ligase alpha subunit Farsa**	sp|Q505J8|SYFA_RAT	58 kDa	2.0308	3.1317	1	1	1	1	0.161917289	2.054166667
**Aspartoacylase Aspa**	sp|Q9R1T5|ACY2_RAT	35 kDa	2.0308	3.1317	1	1	1	1	0.161917289	2.054166667
**Eukaryotic translation initiation factor 2, subunit 2 (Beta) Eif2s2**	tr|Q6P685|Q6P685_RAT	38 kDa	2.0308	3.1317	1	1	1	1	0.161917289	2.054166667
**Nuclease-sensitive element-binding protein 1 Ybx1**	sp|P62961|YBOX1_RAT (+2)	36 kDa	2.0308	3.1317	1	1	1	1	0.161917289	2.054166667
**Signal sequence receptor, alpha Ssr1**	tr|Q4V7D1|Q4V7D1_RAT	32 kDa	2.0308	3.1317	1	1	1	1	0.161917289	2.054166667
**Tyrosine-protein kinase CSK Csk**	sp|P32577|CSK_RAT	51 kDa	2.0308	3.1317	1	1	1	1	0.161917289	2.054166667
**Hepatoma-derived growth factor Hdgf**	sp|Q8VHK7|HDGF_RAT (+1)	26 kDa	2.0308	3.1317	1	1	1	1	0.161917289	2.054166667
**Thyroid hormone receptor-associated protein 3 Thrap3**	sp|Q5M7V8|TR150_RAT	108 kDa	2.0308	3.1317	1	1	1	1	0.161917289	2.054166667
**Testin Tes**	sp|Q2LAP6|TES_RAT	48 kDa	2.0308	3.1317	1	1	1	1	0.161917289	2.054166667
**Collagen alpha-1(III) chain Col3a1**	sp|P13941|CO3A1_RAT	139 kDa	2.0308	3.1317	1	1	1	1	0.161917289	2.054166667
**Nucleophosmin Npm1**	sp|P13084|NPM_RAT	33 kDa	9.1386	10.439	8.7347	5.0104	5.7171	3.0629	0.006891064	2.053044147
**Coatomer subunit beta Copb1**	sp|P23514|COPB_RAT	107 kDa	2.0308	2.0878	3.8821	1	1.9057	1	0.114630358	2.048467624
**Protein S100-A10 S100a10**	sp|P05943|S10AA_RAT	11 kDa	5.077	6.2634	4.8526	3.0063	2.8586	2.0419	0.006490195	2.047984014
**Heterogeneous nuclear ribonucleoprotein U-like 1 (Predicted) Hnrnpul1**	tr|D4A962|D4A962_RAT	96 kDa	3.0462	2.0878	1	1	1	1	0.151902325	2.044666667
**Adipocyte plasma membrane-associated protein Apmap**	sp|Q7TP48|APMAP_RAT	42 kDa	3.0462	2.0878	1	1	1	1	0.151902325	2.044666667
**rRNA 2'-O-methyltransferase fibrillarin Fbl**	sp|P22509|FBRL_RAT	34 kDa	3.0462	2.0878	1	1	1	1	0.151902325	2.044666667
**Complement C1q subcomponent subunit B C1qb**	sp|P31721|C1QB_RAT (+1)	27 kDa	3.0462	2.0878	1	1	1	1	0.151902325	2.044666667
**LIM and cysteine-rich domains 1 Lmcd1**	tr|Q6AYF2|Q6AYF2_RAT	41 kDa	3.0462	2.0878	1	1	1	1	0.151902325	2.044666667
**Cell division cycle 5-like protein Cdc5l**	sp|O08837|CDC5L_RAT	92 kDa	3.0462	2.0878	1	1	1	1	0.151902325	2.044666667
**1-phosphatidylinositol 4,5-bisphosphate phosphodiesterase delta-1 Plcd1**	sp|P10688|PLCD1_RAT	86 kDa	4.0616	6.2634	5.8232	3.0063	2.8586	2.0419	0.020297275	2.042318005
**40S ribosomal protein S13 Rps13**	sp|P62278|RS13_RAT	17 kDa	11.169	9.3952	9.7053	4.0083	5.7171	5.1048	0.002257006	2.041071597
**Biglycan Bgn**	sp|P47853|PGS1_RAT	42 kDa	32.493	31.317	25.234	16.033	13.34	14.293	0.003158633	2.039206705
**Glycerol-3-phosphate dehydrogenase, mitochondrial Gpd2**	sp|P35571|GPDM_RAT	81 kDa	6.0924	9.3952	8.7347	5.0104	3.8114	3.0629	0.023736695	2.038107819
**Protein LOC100362339 LOC100362339**	tr|D4A6G6|D4A6G6_RAT	16 kDa	9.1386	8.3513	6.7937	3.0063	3.8114	5.1048	0.011043561	2.036787586
**Small nuclear ribonucleoprotein-associated protein Snrpb**	tr|B0BN51|B0BN51_RAT	24 kDa	2.0308	4.1756	1.9411	2.0042	1	1	0.160723845	2.034738525
**Protein Srsf1 Srsf1**	tr|D4A9L2|D4A9L2_RAT	28 kDa	7.1078	8.3513	6.7937	4.0083	2.8586	4.0839	0.003687628	2.032070716
**Alpha glucosidase 2 alpha neutral subunit (Predicted) Ganab**	tr|D3ZAN3|D3ZAN3_RAT	91 kDa	5.077	5.2195	7.7642	1	3.8114	4.0839	0.081106846	2.03036435
**Heat shock 27kDa protein 1 Hspb1**	tr|G3V913|G3V913_RAT	23 kDa	28.431	27.142	25.234	11.023	11.434	17.356	0.003694466	2.029663678
**Eukaryotic translation initiation factor 1A Eif1a**	sp|Q6VV72|IF1A_RAT	17 kDa	1	3.1317	1.9411	1	1	1	0.172112744	2.024266667
**Alpha-2-macroglobulin receptor-associated protein Lrpap1**	sp|Q99068|AMRP_RAT	42 kDa	1	3.1317	1.9411	1	1	1	0.172112744	2.024266667
**Isoform 2 of Sequestosome-1 Sqstm1**	sp|O08623-2|SQSTM_RAT (+1)	45 kDa	1	3.1317	1.9411	1	1	1	0.172112744	2.024266667
**Parkinson disease 7 domain containing 1 (Predicted), isoform CRA_a LOC100911365**	tr|D3ZVI9|D3ZVI9_RAT	23 kDa	1	3.1317	1.9411	1	1	1	0.172112744	2.024266667
**N-acylethanolamine-hydrolyzing acid amidase Naaa**	sp|Q5KTC7|NAAA_RAT	40 kDa	1	3.1317	1.9411	1	1	1	0.172112744	2.024266667
**Protein Tjp1 Tjp1**	tr|F1M4A0|F1M4A0_RAT	194 kDa	19.293	25.054	17.469	12.025	12.387	6.1258	0.027005563	2.024245361
**Lethal(2) giant larvae protein homolog 1 Llgl1**	tr|G3V6I1|G3V6I1_RAT	113 kDa	11.169	7.3073	13.587	4.0083	5.7171	6.1258	0.049555891	2.022767992
**Coatomer subunit delta Arcn1**	sp|Q66H80|COPD_RAT	57 kDa	3.0462	5.2195	5.8232	2.0042	1.9057	3.0629	0.061623764	2.020551285
**sp|KCRM_HUMAN|**	zz|ZZ_FGCZCont0110|	43 kDa	4.0616	1	1	1	1	1	0.373900966	2.020533333
**40S ribosomal protein S15a Rps15a**	sp|P62246|RS15A_RAT	15 kDa	2.0308	2.0878	1.9411	1	1	1	1.82175E-05	2.0199
**ELAV (Embryonic lethal, abnormal vision, Drosophila)-like 1 (Hu antigen R) Elavl1**	tr|B5DF91|B5DF91_RAT	36 kDa	2.0308	2.0878	1.9411	1	1	1	1.82175E-05	2.0199
**Neuronal migration protein doublecortin Dcx**	tr|G3V997|G3V997_RAT	41 kDa	2.0308	2.0878	1.9411	1	1	1	1.82175E-05	2.0199
**Pro-cathepsin H Ctsh**	sp|P00786|CATH_RAT	37 kDa	2.0308	2.0878	1.9411	1	1	1	1.82175E-05	2.0199
**Protein Hyi Hyi**	tr|F1LZJ4|F1LZJ4_RAT	31 kDa	2.0308	2.0878	1.9411	1	1	1	1.82175E-05	2.0199
**Protein Plekho2 Plekho2**	tr|D3Z9K4|D3Z9K4_RAT	54 kDa	2.0308	2.0878	1.9411	1	1	1	1.82175E-05	2.0199
**Cathepsin Z Ctsz**	sp|Q9R1T3|CATZ_RAT	34 kDa	2.0308	2.0878	1.9411	1	1	1	1.82175E-05	2.0199
**Signal peptidase complex catalytic subunit SEC11C Sec11c**	sp|Q9WTR7|SC11C_RAT (+1)	22 kDa	2.0308	2.0878	1.9411	1	1	1	1.82175E-05	2.0199
**Interferon, alpha-inducible protein (Clone IFI-15K) (Predicted) Isg15**	tr|D4A3X3|D4A3X3_RAT	18 kDa	2.0308	2.0878	1.9411	1	1	1	1.82175E-05	2.0199
**Transthyretin Ttr**	sp|P02767|TTHY_RAT	16 kDa	2.0308	2.0878	1.9411	1	1	1	1.82175E-05	2.0199
**DnaJ homolog subfamily B member 11 Dnajb11**	sp|Q6TUG0|DJB11_RAT	40 kDa	2.0308	2.0878	1.9411	1	1	1	1.82175E-05	2.0199
**Protein Parvb Parvb**	tr|D3ZKG5|D3ZKG5_RAT	42 kDa	2.0308	2.0878	1.9411	1	1	1	1.82175E-05	2.0199
**Signal peptidase complex subunit 3 Spcs3**	tr|D3ZF12|D3ZF12_RAT	20 kDa	2.0308	2.0878	1.9411	1	1	1	1.82175E-05	2.0199
**Protein Hebp1 Hebp1**	tr|F7EVC6|F7EVC6_RAT	15 kDa	2.0308	2.0878	1.9411	1	1	1	1.82175E-05	2.0199
**Mesencephalic astrocyte-derived neurotrophic factor Manf**	sp|P0C5H9|MANF_RAT	20 kDa	4.0616	4.1756	1.9411	2.0042	1	2.0419	0.100070185	2.017062682
**LUC7-like 2 (S. cerevisiae) Luc7l2**	tr|B2RYP6|B2RYP6_RAT	47 kDa	2.0308	3.1317	2.9116	2.0042	1	1	0.045978132	2.016407772
**DAZ associated protein 1 Dazap1**	tr|Q4KLZ3|Q4KLZ3_RAT	43 kDa	4.0616	5.2195	2.9116	3.0063	1	2.0419	0.081147611	2.015922093
**Mitochondrial import inner membrane translocase subunit Tim13 Timm13**	sp|P62076|TIM13_RAT	10 kDa	3.0462	2.0878	2.9116	2.0042	1	1	0.03998418	2.009290245
**60S ribosomal protein L27 Rpl27**	sp|P61354|RL27_RAT	16 kDa	4.0616	4.1756	3.8821	3.0063	1	2.0419	0.025914412	2.00378625
**Actin related protein 2/3 complex, subunit 3 Arpc3**	tr|B2GV73|B2GV73_RAT	21 kDa	4.0616	3.1317	2.9116	2.0042	1	2.0419	0.026349566	2.002516795
**Protein Dtna-ps1 Dtna-ps1**	tr|D3ZD04|D3ZD04_RAT (+5)	78 kDa	3.0462	1	1.9411	1	1	1	0.167472088	1.995766667
**Osteoglycin (Predicted) Ogn**	tr|D3ZVB7|D3ZVB7_RAT	34 kDa	25.385	27.142	18.44	11.023	13.34	11.231	0.012894547	1.993791088
**Protein RT1-CE2 (Fragment) RT1-CE2**	tr|D3ZQG9|D3ZQG9_RAT	41 kDa	2.0308	1	2.9116	1	1	1	0.150465622	1.9808
**Heat shock protein beta-8 Hspb8**	sp|Q9EPX0|HSPB8_RAT	22 kDa	2.0308	1	2.9116	1	1	1	0.150465622	1.9808
**Atlastin-1 Atl1**	sp|Q6PST4|ATLA1_RAT	63 kDa	2.0308	1	2.9116	1	1	1	0.150465622	1.9808
**Isoform 2 of Latrophilin-3 Lphn3**	sp|Q9Z173-2|LPHN3_RAT (+6)	163 kDa	2.0308	1	2.9116	1	1	1	0.150465622	1.9808
**Microtubule-actin cross-linking factor 1 Macf1**	sp|D3ZHV2|MACF1_RAT	620 kDa	2.0308	1	2.9116	1	1	1	0.150465622	1.9808
**PRA1 family protein 3 Arl6ip5**	sp|Q9ES40|PRAF3_RAT	22 kDa	2.0308	2.0878	3.8821	1	1	2.0419	0.132510999	1.979440362
**Far upstream element-binding protein 1 Fubp1**	sp|Q32PX7|FUBP1_RAT	67 kDa	3.0462	2.0878	4.8526	1	1	3.0629	0.197453294	1.972505876
**Myl9 protein Myl9**	tr|B0BMS8|B0BMS8_RAT	20 kDa	7.1078	7.3073	6.7937	1	5.7171	4.0839	0.067198635	1.963595963
**Protein Slc12a2 Slc12a2**	tr|E9PTX9|E9PTX9_RAT	130 kDa	4.0616	6.2634	6.7937	2.0042	5.7171	1	0.167156538	1.962861041
**Protein Ipo5 Ipo5**	tr|D4A781|D4A781_RAT	124 kDa	1	1	3.8821	1	1	1	0.373900966	1.9607
**A-kinase anchor protein 2 Akap2**	sp|Q5U301|AKAP2_RAT (+1)	96 kDa	1	1	3.8821	1	1	1	0.373900966	1.9607
**Endoplasmin Hsp90b1**	sp|Q66HD0|ENPL_RAT	93 kDa	34.524	35.493	31.057	19.04	15.246	17.356	0.000689375	1.957205375
**Heterogeneous nuclear ribonucleoprotein Q Syncrip**	sp|Q7TP47|HNRPQ_RAT	60 kDa	6.0924	9.3952	7.7642	5.0104	3.8114	3.0629	0.026894471	1.956448207
**CArG-binding factor A Hnrnpab**	tr|Q9QX80|Q9QX80_RAT (+1)	31 kDa	4.0616	6.2634	4.8526	3.0063	3.8114	1	0.080692834	1.941440577
**Protein Lama2 (Fragment) Lama2**	tr|F1M614|F1M614_RAT	281 kDa	17.262	28.185	15.528	13.027	11.434	7.1467	0.086778944	1.929118538
**Isoform 1 of Core histone macro-H2A.1 H2afy**	sp|Q02874-2|H2AY_RAT (+1)	39 kDa	11.169	12.527	8.7347	6.0125	5.7171	5.1048	0.010370575	1.926454165
**Eukaryotic translation initiation factor 2 subunit 1 Eif2s1**	sp|P68101|IF2A_RAT	36 kDa	6.0924	5.2195	5.8232	4.0083	2.8586	2.0419	0.011873512	1.923390356
**LIM and SH3 domain protein 1 Lasp1**	sp|Q99MZ8|LASP1_RAT	30 kDa	11.169	11.483	9.7053	6.0125	5.7171	5.1048	0.001055746	1.922094046
**Protein Sfpq (Fragment) Sfpq**	tr|F1LQW3|F1LQW3_RAT	66 kDa	12.185	16.703	14.558	8.0167	8.5757	6.1258	0.009999067	1.912387425
**Protein Tom1 Tom1**	tr|Q5XI21|Q5XI21_RAT	54 kDa	5.077	5.2195	1	3.0063	1.9057	1	0.297568179	1.910774696
**Pre-mRNA-processing factor 19 Prpf19**	sp|Q9JMJ4|PRP19_RAT	55 kDa	5.077	5.2195	2.9116	4.0083	1.9057	1	0.145439867	1.910341336
**Amine oxidase [flavin-containing] B Maob**	sp|P19643|AOFB_RAT (+1)	58 kDa	4.0616	4.1756	6.7937	1	3.8114	3.0629	0.123534256	1.90885539
**Protein LOC687711 LOC687711**	tr|M0R907|M0R907_RAT	14 kDa	4.0616	5.2195	3.8821	2.0042	2.8586	2.0419	0.014320726	1.906411575
**Protein Sar1a Sar1a**	tr|Q6AY18|Q6AY18_RAT	22 kDa	4.0616	5.2195	3.8821	2.0042	2.8586	2.0419	0.014320726	1.906411575
**60S ribosomal protein L8 Rpl8**	sp|P62919|RL8_RAT	28 kDa	9.1386	8.3513	4.8526	2.0042	4.7643	5.1048	0.100857498	1.881743071
**Ferritin Fth1**	tr|Q66HI5|Q66HI5_RAT	21 kDa	22.339	27.142	21.352	13.027	12.387	12.252	0.003593905	1.880555408
**Acidic leucine-rich nuclear phosphoprotein 32 family member B Anp32b**	sp|Q9EST6|AN32B_RAT	31 kDa	4.0616	4.1756	2.9116	2.0042	1.9057	2.0419	0.012951772	1.873181222
**Prenylcysteine oxidase Pcyox1**	sp|Q99ML5|PCYOX_RAT	56 kDa	7.1078	7.3073	7.7642	3.0063	4.7643	4.0839	0.003269576	1.870960395
**60S ribosomal protein L10a Rpl10a**	sp|P62907|RL10A_RAT	25 kDa	6.0924	7.3073	6.7937	4.0083	4.7643	2.0419	0.024129501	1.8672523

**Na(+)/H(+) exchange regulatory cofactor NHE-RF1 Slc9a3r1**	sp|Q9JJ19|NHRF1_RAT	39 kDa	10.154	7.3073	6.7937	4.0083	2.8586	6.1258	0.057015831	1.866817521
**Protein LOC100910660 Srsf3**	tr|Q0ZFS8|Q0ZFS8_RAT	19 kDa	3.0462	4.1756	3.8821	1	1.9057	3.0629	0.067152945	1.86038602
**Transmembrane emp24 domain-containing protein 10 Tmed10**	sp|Q63584|TMEDA_RAT	25 kDa	3.0462	6.2634	1.9411	3.0063	1	2.0419	0.289048248	1.860173275
**Delta(3,5)-Delta(2,4)-dienoyl-CoA isomerase, mitochondrial Ech1**	sp|Q62651|ECH1_RAT	36 kDa	16.246	14.615	9.7053	8.0167	5.7171	8.1677	0.042584578	1.852215602
**26S proteasome non-ATPase regulatory subunit 11 Psmd11**	sp|F1LMZ8|PSD11_RAT	47 kDa	5.077	7.3073	7.7642	3.0063	3.8114	4.0839	0.025826424	1.848214941
**Heterogeneous nuclear ribonucleoprotein C LOC100911576**	tr|G3V9R8|G3V9R8_RAT	33 kDa	9.1386	12.527	7.7642	6.0125	3.8114	6.1258	0.048681195	1.845163232
**Proteasome (Prosome, macropain) 26S subunit, non-ATPase, 7 (Predicted) Psmd7**	tr|D4AEH3|D4AEH3_RAT	36 kDa	3.0462	3.1317	6.7937	3.0063	1	3.0629	0.235166741	1.834945963
**Peripheral plasma membrane protein CASK Cask**	sp|Q62915|CSKP_RAT	103 kDa	4.0616	3.1317	3.8821	3.0063	1	2.0419	0.060309164	1.831189445
**Catenin beta-1 Ctnnb1**	sp|Q9WU82|CTNB1_RAT	85 kDa	9.1386	9.3952	6.7937	5.0104	4.7643	4.0839	0.011876893	1.827565555
**Methylcrotonoyl-CoA carboxylase beta chain, mitochondrial Mccc2**	sp|Q5XIT9|MCCB_RAT	62 kDa	4.0616	3.1317	1.9411	3.0063	1	1	0.20409989	1.824581028
**Retinoid-inducible serine carboxypeptidase Scpep1**	sp|Q920A6|RISC_RAT	51 kDa	2.0308	4.1756	2.9116	3.0063	1	1	0.207957401	1.821305156
**60S ribosomal protein L18 Rpl18**	sp|P12001|RL18_RAT	22 kDa	10.154	7.3073	7.7642	5.0104	4.7643	4.0839	0.014906449	1.820205504
**Eukaryotic translation initiation factor 3 subunit H Eif3h**	sp|Q6P9U8|EIF3H_RAT	40 kDa	2.0308	3.1317	1.9411	1	1.9057	1	0.094057411	1.818777684
**Cytochrome c oxidase subunit 7A2, mitochondrial Cox7a2**	sp|P35171|CX7A2_RAT	9 kDa	3.0462	3.1317	2.9116	3.0063	1	1	0.112735643	1.815612328
**60S ribosomal protein L23a Rpl23a**	sp|P62752|RL23A_RAT (+2)	18 kDa	8.1232	7.3073	7.7642	4.0083	5.7171	3.0629	0.012918711	1.813743813
**Protein LOC100910272 LOC100910272**	tr|D4A7R0|D4A7R0_RAT	75 kDa	3.0462	2.0878	1.9411	1	1.9057	1	0.083052704	1.811480656
**Protein LOC681825 LOC681825**	tr|M0R919|M0R919_RAT	20 kDa	3.0462	2.0878	1.9411	1	1.9057	1	0.083052704	1.811480656
**Chloride intracellular channel 4, isoform CRA_b Clic4**	tr|G3V8C4|G3V8C4_RAT	29 kDa	12.185	9.3952	10.676	7.0146	5.7171	5.1048	0.008114559	1.808437754
**Protein Snrpd2 Snrpd2**	tr|B5DES0|B5DES0_RAT	14 kDa	1	5.2195	1	2.0042	1	1	0.499669273	1.802981869
**Protein RGD1564698 RGD1564698**	tr|F1LT36|F1LT36_RAT	19 kDa	9.1386	6.2634	7.7642	4.0083	4.7643	4.0839	0.016458474	1.801905651
**GMP synthase [glutamine-hydrolyzing] Gmps**	sp|Q4V7C6|GUAA_RAT	77 kDa	2.0308	2.0878	2.9116	1	1.9057	1	0.066032583	1.799984638
**COP9 (Constitutive photomorphogenic) homolog, subunit 6 (Arabidopsis thaliana) (Predicted), isoform CRA_a Cops6**	tr|D3ZI16|D3ZI16_RAT	38 kDa	2.0308	2.0878	2.9116	1	1.9057	1	0.066032583	1.799984638
**Calnexin Canx**	sp|P35565|CALX_RAT	67 kDa	8.1232	10.439	9.7053	3.0063	7.6229	5.1048	0.049486793	1.796587009
**High density lipoprotein binding protein Hdlbp**	tr|Q3KRF2|Q3KRF2_RAT	142 kDa	11.169	16.703	9.7053	6.0125	5.7171	9.1887	0.081978249	1.796384027
**Cold-inducible RNA-binding protein Cirbp**	sp|P60825|CIRBP_RAT	19 kDa	3.0462	3.1317	1	2.0042	1	1	0.242957436	1.792592778
**Aspartate--tRNA ligase, cytoplasmic Dars**	sp|P15178|SYDC_RAT	57 kDa	4.0616	6.2634	7.7642	4.0083	1	5.1048	0.178513934	1.788689917
**Isoamyl acetate-hydrolyzing esterase 1 homolog Iah1**	sp|Q711G3|IAH1_RAT	28 kDa	3.0462	1	2.9116	1	1.9057	1	0.233987048	1.781447628
**60S ribosomal protein L17 Rpl17**	sp|P24049|RL17_RAT	21 kDa	4.0616	6.2634	3.8821	2.0042	1.9057	4.0839	0.118361849	1.77726488
**PC4 and SFRS1 interacting protein 1 SBP75**	tr|F1SW39|F1SW39_RAT	60 kDa	4.0616	5.2195	2.9116	3.0063	2.8586	1	0.12810185	1.776092878
**Uncharacterized protein Fam98b**	tr|D4AE02|D4AE02_RAT	46 kDa	2.0308	3.1317	1.9411	2.0042	1	1	0.111994861	1.774037261
**High mobility group protein B1 Hmgb1**	sp|P63159|HMGB1_RAT (+2)	25 kDa	11.169	13.571	8.7347	6.0125	5.7171	7.1467	0.029182578	1.7733719
**Fusion, derived from t(12;16) malignant liposarcoma (Human) Fus**	tr|Q5PQK2|Q5PQK2_RAT	53 kDa	10.154	9.3952	6.7937	5.0104	4.7643	5.1048	0.02017536	1.770415673
**Cysteine-rich protein 2 Crip2**	sp|P36201|CRIP2_RAT	23 kDa	7.1078	6.2634	5.8232	2.0042	4.7643	4.0839	0.038035382	1.768677896
**Isoform Short of Cytochrome b5 Cyb5a**	sp|P00173-2|CYB5_RAT (+2)	11 kDa	6.0924	7.3073	5.8232	4.0083	3.8114	3.0629	0.006740801	1.766388547
**Transgelin Tagln**	sp|P31232|TAGL_RAT	23 kDa	9.1386	4.1756	5.8232	2.0042	4.7643	4.0839	0.175354944	1.763425602
**Protein Stub1 Stub1**	tr|D4A4T0|D4A4T0_RAT	35 kDa	4.0616	4.1756	5.8232	3.0063	1.9057	3.0629	0.041055842	1.763081669
**Peroxisomal multifunctional enzyme type 2 Hsd17b4**	sp|P97852|DHB4_RAT	79 kDa	19.293	20.878	19.411	12.025	9.5286	12.252	0.001049089	1.762489055
**Protein Ruvbl2 Ruvbl2**	tr|G3V8T5|G3V8T5_RAT	51 kDa	2.0308	3.1317	1.9411	1	1	2.0419	0.119546755	1.757490289
**Multifunctional protein ADE2 Paics**	sp|P51583|PUR6_RAT	47 kDa	8.1232	11.483	11.646	6.0125	6.67	5.1048	0.022046867	1.756995159
**Electron transfer flavoprotein-ubiquinone oxidoreductase, mitochondrial Etfdh**	sp|Q6UPE1|ETFD_RAT (+1)	68 kDa	3.0462	5.2195	3.8821	1	2.8586	3.0629	0.128630495	1.755081991
**Nucleolin Ncl**	sp|P13383|NUCL_RAT (+1)	77 kDa	9.1386	11.483	10.676	7.0146	5.7171	5.1048	0.007236811	1.754694026
**Fatty acid-binding protein, brain Fabp7**	sp|P55051|FABP7_RAT	15 kDa	19.293	13.571	17.469	9.0188	10.481	9.1887	0.014568302	1.754466075
**40S ribosomal protein S14 Rps14**	sp|P13471|RS14_RAT (+1)	16 kDa	5.077	4.1756	4.8526	4.0083	1	3.0629	0.096234607	1.747596392
**Non-specific lipid-transfer protein Scp2**	sp|P11915|NLTP_RAT (+1)	59 kDa	13.2	8.3513	9.7053	7.0146	4.7643	6.1258	0.048483542	1.745720397
**60S ribosomal protein L15 Rpl15**	sp|P61314|RL15_RAT	24 kDa	6.0924	6.2634	6.7937	3.0063	2.8586	5.1048	0.022573719	1.74567217
**Eukaryotic translation initiation factor 3 subunit A Eif3a**	sp|Q1JU68|EIF3A_RAT	163 kDa	1	3.1317	2.9116	1	1	2.0419	0.258808891	1.742571563
**60S ribosomal protein L26 Rpl26**	tr|G3V6I9|G3V6I9_RAT	17 kDa	5.077	6.2634	5.8232	4.0083	3.8114	2.0419	0.027138421	1.740447798
**Proliferation-associated 2G4 Pa2g4**	tr|Q6AYD3|Q6AYD3_RAT	44 kDa	7.1078	6.2634	3.8821	3.0063	2.8586	4.0839	0.079298075	1.734209151
**Histone H4 Hist1h4b**	sp|P62804|H4_RAT	11 kDa	29.447	29.229	20.381	15.031	15.246	15.314	0.02022733	1.734048387
**Protein Sec23a Sec23a**	tr|B5DFC3|B5DFC3_RAT	86 kDa	2.0308	5.2195	2.9116	2.0042	2.8586	1	0.259641217	1.733284437
**Protein Setd7 Setd7**	tr|D4ADE5|D4ADE5_RAT	41 kDa	4.0616	4.1756	1.9411	1	2.8586	2.0419	0.189842373	1.724989408
**Protein disulfide-isomerase P4hb**	sp|P04785|PDIA1_RAT	57 kDa	24.37	29.229	26.204	15.031	16.199	15.314	0.001612226	1.714571158
**40S ribosomal protein S27 Rps27**	sp|Q71TY3|RS27_RAT	9 kDa	1	3.1317	1	1	1	1	0.373900966	1.710566667
**Protein RT1-CE4 RT1-CE4**	tr|D4A0E0|D4A0E0_RAT (+1)	40 kDa	1	3.1317	1	1	1	1	0.373900966	1.710566667
**Uncharacterized protein (Fragment)**	tr|E9PT39|E9PT39_RAT	28 kDa	1	3.1317	1	1	1	1	0.373900966	1.710566667
**Microtubule-associated protein 1S Map1s**	sp|P0C5W1|MAP1S_RAT	103 kDa	1	3.1317	1	1	1	1	0.373900966	1.710566667
**Protein Col4a1 Col4a1**	tr|F1MA59|F1MA59_RAT	161 kDa	1	3.1317	1	1	1	1	0.373900966	1.710566667
**T-kininogen 2**	sp|P08932|KNT2_RAT	48 kDa	1	3.1317	1	1	1	1	0.373900966	1.710566667
**Fermt3 protein Fermt3**	tr|B2GVB9|B2GVB9_RAT	76 kDa	1	3.1317	1	1	1	1	0.373900966	1.710566667
**Protein Rcn3 Rcn3**	tr|I6L9G5|I6L9G5_RAT	38 kDa	1	3.1317	1	1	1	1	0.373900966	1.710566667
**Protein Hook3 (Fragment) Hook3**	tr|F1MA65|F1MA65_RAT (+1)	78 kDa	1	3.1317	1	1	1	1	0.373900966	1.710566667
**Methylcrotonoyl-CoA carboxylase subunit alpha, mitochondrial Mccc1**	sp|Q5I0C3|MCCA_RAT (+1)	79 kDa	1	3.1317	1	1	1	1	0.373900966	1.710566667
**Paxillin Pxn**	sp|Q66H76|PAXI_RAT (+1)	64 kDa	1	3.1317	1	1	1	1	0.373900966	1.710566667
**Protein Itih4 Itih4**	tr|D3ZFC6|D3ZFC6_RAT (+1)	103 kDa	1	3.1317	1	1	1	1	0.373900966	1.710566667
**Lymphocyte specific 1, isoform CRA_a Lsp1**	tr|Q4QQV6|Q4QQV6_RAT	37 kDa	1	3.1317	1	1	1	1	0.373900966	1.710566667
**Protein Rprd1b Rprd1b**	tr|B5DEK0|B5DEK0_RAT	37 kDa	1	3.1317	1	1	1	1	0.373900966	1.710566667
**Protein Spg20 Spg20**	tr|E9PT90|E9PT90_RAT	65 kDa	1	3.1317	1	1	1	1	0.373900966	1.710566667
**Ig gamma-2B chain C region Igh-1a**	sp|P20761|IGG2B_RAT (+1)	36 kDa	1	3.1317	1	1	1	1	0.373900966	1.710566667
**Protein S100-A11 S100a11**	sp|Q6B345|S10AB_RAT	11 kDa	1	3.1317	1	1	1	1	0.373900966	1.710566667
**Nuclear ubiquitous casein and cyclin-dependent kinase substrate 1 Nucks1**	sp|Q9EPJ0|NUCKS_RAT	27 kDa	1	3.1317	1	1	1	1	0.373900966	1.710566667
**H/ACA ribonucleoprotein complex subunit 4 Dkc1**	sp|P40615|DKC1_RAT	57 kDa	1	3.1317	1	1	1	1	0.373900966	1.710566667
**Prolyl 4-hydroxylase subunit alpha-1 P4ha1**	sp|P54001|P4HA1_RAT	61 kDa	1	3.1317	1	1	1	1	0.373900966	1.710566667
**Myotilin (Predicted) Myot**	tr|D3ZTC5|D3ZTC5_RAT (+2)	55 kDa	1	3.1317	1	1	1	1	0.373900966	1.710566667
**Calsequestrin-1 Casq1**	sp|P19633|CASQ1_RAT	46 kDa	1	3.1317	1	1	1	1	0.373900966	1.710566667
**Olfactomedin-like protein 3 Olfml3**	sp|B0BNI5|OLFL3_RAT (+1)	46 kDa	1	3.1317	1	1	1	1	0.373900966	1.710566667
**Protein Numa1 (Fragment) Numa1**	tr|F1LW91|F1LW91_RAT	233 kDa	1	3.1317	1	1	1	1	0.373900966	1.710566667
**Poly (ADP-ribose) polymerase family, member 3 Parp3**	tr|Q5U2U3|Q5U2U3_RAT	59 kDa	1	3.1317	1	1	1	1	0.373900966	1.710566667
**Emd protein Emd**	tr|Q6PCU4|Q6PCU4_RAT	30 kDa	1	3.1317	1	1	1	1	0.373900966	1.710566667
**Protein S100a16 S100a16**	tr|B0BMX3|B0BMX3_RAT	14 kDa	5.077	3.1317	3.8821	3.0063	1	3.0629	0.131144447	1.71034912
**Protein Pkn3 Pkn3**	tr|D3ZC07|D3ZC07_RAT	107 kDa	4.0616	4.1756	1.9411	2.0042	1.9057	2.0419	0.124954011	1.710121308
**60S ribosomal protein L35a Rpl35a**	sp|P04646|RL35A_RAT (+1)	13 kDa	2.0308	2.0878	1	1	1	1	0.116404357	1.7062
**Charged multivesicular body protein 3 Chmp3**	sp|Q8CGS4|CHMP3_RAT	25 kDa	2.0308	2.0878	1	1	1	1	0.116404357	1.7062
**Isoform 2 of Protein phosphatase 1 regulatory subunit 12A Ppp1r12a**	sp|Q10728-2|MYPT1_RAT (+5)	109 kDa	2.0308	2.0878	1	1	1	1	0.116404357	1.7062
**Transcription intermediary factor 1-beta Trim28**	sp|O08629|TIF1B_RAT	89 kDa	2.0308	2.0878	1	1	1	1	0.116404357	1.7062
**Cleavage and polyadenylation specificity factor subunit 5 Nudt21**	sp|Q4KM65|CPSF5_RAT	26 kDa	2.0308	2.0878	1	1	1	1	0.116404357	1.7062
**Protein Smarcc2 Smarcc2**	tr|D3ZPF5|D3ZPF5_RAT (+1)	132 kDa	2.0308	2.0878	1	1	1	1	0.116404357	1.7062
**Medium-chain specific acyl-CoA dehydrogenase, mitochondrial Acadm**	sp|P08503|ACADM_RAT (+1)	47 kDa	2.0308	2.0878	1	1	1	1	0.116404357	1.7062
**Glutathione S-transferase alpha-4 Gsta4**	sp|P14942|GSTA4_RAT	26 kDa	2.0308	2.0878	1	1	1	1	0.116404357	1.7062
**Protein U2af2 LOC690372**	tr|F2Z3T9|F2Z3T9_RAT	54 kDa	2.0308	2.0878	1	1	1	1	0.116404357	1.7062
**Proteasomal ubiquitin receptor ADRM1 Adrm1**	sp|Q9JMB5|ADRM1_RAT	42 kDa	2.0308	2.0878	1	1	1	1	0.116404357	1.7062
**Protein Snx9 (Fragment) Snx9**	tr|M0R9L3|M0R9L3_RAT	66 kDa	2.0308	2.0878	1	1	1	1	0.116404357	1.7062
**Proteasome subunit beta type Psmb9**	tr|Q6MGA6|Q6MGA6_RAT	23 kDa	2.0308	2.0878	1	1	1	1	0.116404357	1.7062
**Choline dehydrogenase, mitochondrial Chdh**	sp|Q6UPE0|CHDH_RAT	66 kDa	2.0308	2.0878	1	1	1	1	0.116404357	1.7062
**Uncharacterized protein (Fragment)**	tr|M0R433|M0R433_RAT	70 kDa	2.0308	2.0878	1	1	1	1	0.116404357	1.7062
**Interferon-induced protein 35 Ifi35**	tr|Q5M849|Q5M849_RAT	32 kDa	2.0308	2.0878	1	1	1	1	0.116404357	1.7062
**KDEL motif-containing protein 2 Kdelc2**	sp|Q566E5|KDEL2_RAT	59 kDa	2.0308	2.0878	1	1	1	1	0.116404357	1.7062
**Peptidyl-prolyl cis-trans isomerase FKBP9 Fkbp9**	sp|Q66H94|FKBP9_RAT	63 kDa	2.0308	2.0878	1	1	1	1	0.116404357	1.7062
**Protein Specc1 Specc1**	tr|Q4KLM7|Q4KLM7_RAT	87 kDa	2.0308	2.0878	1	1	1	1	0.116404357	1.7062
**Glucosamine (N-acetyl)-6-sulfatase Gns**	tr|Q32KJ5|Q32KJ5_RAT	61 kDa	2.0308	2.0878	1	1	1	1	0.116404357	1.7062
**Protein Tmed4 Tmed4**	tr|G3V6N2|G3V6N2_RAT	20 kDa	2.0308	2.0878	1	1	1	1	0.116404357	1.7062
**Lactadherin Mfge8**	sp|P70490|MFGM_RAT (+1)	47 kDa	2.0308	2.0878	1	1	1	1	0.116404357	1.7062
**Protein Sf3a3 Sf3a3**	tr|Q4KLI7|Q4KLI7_RAT	59 kDa	2.0308	2.0878	1	1	1	1	0.116404357	1.7062
**Protein Snrpa1 Snrpa1**	tr|D3ZZR5|D3ZZR5_RAT	28 kDa	2.0308	2.0878	1	1	1	1	0.116404357	1.7062
**Inosine-5'-monophosphate dehydrogenase 2 Impdh2**	sp|E9PU28|IMDH2_RAT	56 kDa	2.0308	2.0878	1	1	1	1	0.116404357	1.7062
**Myosin, heavy polypeptide 9, non-muscle LOC100911597**	tr|G3V6P7|G3V6P7_RAT	226 kDa	25.385	31.317	28.145	15.031	14.293	20.419	0.010557561	1.705707336
**Catalase Cat**	sp|P04762|CATA_RAT	60 kDa	20.308	15.659	23.293	10.021	10.481	14.293	0.035013783	1.703118264
**LRRGT00050 Eprs**	tr|Q6TXE9|Q6TXE9_RAT	167 kDa	9.1386	9.3952	11.646	4.0083	7.6229	6.1258	0.034687462	1.699600158
**Aldehyde dehydrogenase, mitochondrial Aldh2**	tr|F1LN88|F1LN88_RAT	57 kDa	20.308	20.878	19.411	11.023	12.387	12.252	0.00016666	1.699203634
**Annexin A3 Anxa3**	sp|P14669|ANXA3_RAT (+1)	36 kDa	18.277	17.746	21.352	11.023	8.5757	14.293	0.01738087	1.69289236
**Heat shock protein 75 kDa, mitochondrial Trap1**	sp|Q5XHZ0|TRAP1_RAT	80 kDa	3.0462	7.3073	4.8526	3.0063	1.9057	4.0839	0.209508718	1.690336709
**Ras-related protein Rab-8A Rab8a**	sp|P35280|RAB8A_RAT	24 kDa	3.0462	1	1	1	1	1	0.373900966	1.682066667
**Ig gamma-1 chain C region**	sp|P20759|IGHG1_RAT (+1)	36 kDa	3.0462	1	1	1	1	1	0.373900966	1.682066667
**Nicotinamide nucleotide transhydrogenase Nnt**	tr|Q5BJZ3|Q5BJZ3_RAT	114 kDa	3.0462	1	1	1	1	1	0.373900966	1.682066667
**Prothymosin alpha Ptma**	sp|P06302|PTMA_RAT	12 kDa	3.0462	1	1	1	1	1	0.373900966	1.682066667
**Ewsr1 protein Ewsr1**	tr|B1WC50|B1WC50_RAT (+1)	68 kDa	3.0462	1	1	1	1	1	0.373900966	1.682066667
**Protein Slc25a24 Slc25a24**	tr|B1WC67|B1WC67_RAT	53 kDa	3.0462	1	1	1	1	1	0.373900966	1.682066667
**Sorbin and SH3 domain-containing protein 2 Sorbs2**	sp|O35413|SRBS2_RAT (+1)	134 kDa	3.0462	1	1	1	1	1	0.373900966	1.682066667
**Coiled-coil domain-containing protein 47 Ccdc47**	sp|Q5U2X6|CCD47_RAT	56 kDa	3.0462	1	1	1	1	1	0.373900966	1.682066667
**Peptidyl-prolyl cis-trans isomerase (Fragment) Fkbp15**	tr|D4AE06|D4AE06_RAT	131 kDa	3.0462	1	1	1	1	1	0.373900966	1.682066667
**Dipeptidyl peptidase 1 Ctsc**	sp|P80067|CATC_RAT	52 kDa	3.0462	1	1	1	1	1	0.373900966	1.682066667
**Putative N-acetylglucosamine-6-phosphate deacetylase Amdhd2**	sp|Q5BJY6|NAGA_RAT	44 kDa	3.0462	1	1	1	1	1	0.373900966	1.682066667

**Myosin light chain 3 Myl3**	sp|P16409|MYL3_RAT	22 kDa	3.0462	1	1	1	1	1	0.373900966	1.682066667
**Histone H3 Hist1h3c**	tr|D3ZJ08|D3ZJ08_RAT	15 kDa	9.1386	10.439	8.7347	5.0104	5.7171	6.1258	0.003292351	1.679926187
**Histone H2B Hist3h2ba**	tr|D3ZNZ9|D3ZNZ9_RAT (+1)	14 kDa	22.339	29.229	23.293	15.031	15.246	14.293	0.009710556	1.679627552
**Sulfated glycoprotein 1 Psap**	sp|P10960|SAP_RAT	61 kDa	18.277	17.746	12.617	12.025	5.7171	11.231	0.070857701	1.67879861
**Protein Eci3 Eci3**	tr|Q5M884|Q5M884_RAT	34 kDa	1	2.0878	1.9411	1	1	1	0.118191861	1.6763
**Prostaglandin-H2 D-isomerase Ptgds**	sp|P22057|PTGDS_RAT	21 kDa	1	2.0878	1.9411	1	1	1	0.118191861	1.6763
**Protein Erh (Fragment) Erh**	tr|F1M4T3|F1M4T3_RAT	14 kDa	1	2.0878	1.9411	1	1	1	0.118191861	1.6763
**Myosin phosphatase Rho-interacting protein Mprip**	tr|G3V9F3|G3V9F3_RAT	117 kDa	1	2.0878	1.9411	1	1	1	0.118191861	1.6763
**Basal cell adhesion molecule Bcam**	sp|Q9ESS6|BCAM_RAT	68 kDa	1	2.0878	1.9411	1	1	1	0.118191861	1.6763
**LSM8 homolog, U6 small nuclear RNA associated (S. cerevisiae) Naa38**	tr|B2RZB6|B2RZB6_RAT	10 kDa	1	2.0878	1.9411	1	1	1	0.118191861	1.6763
**Syntaxin-7 Stx7**	sp|O70257|STX7_RAT	30 kDa	1	2.0878	1.9411	1	1	1	0.118191861	1.6763
**Protein Col4a2 Col4a2**	tr|F1M6Q3|F1M6Q3_RAT	166 kDa	1	2.0878	1.9411	1	1	1	0.118191861	1.6763
**Tyrosine-protein phosphatase non-receptor type substrate 1 Sirpa**	sp|P97710|SHPS1_RAT	56 kDa	1	2.0878	1.9411	1	1	1	0.118191861	1.6763
**Isoform 4 of CUGBP Elav-like family member 2 Celf2**	sp|Q792H5-4|CELF2_RAT (+3)	57 kDa	1	2.0878	1.9411	1	1	1	0.118191861	1.6763
**Uncharacterized protein**	tr|D4A567|D4A567_RAT	68 kDa	1	2.0878	1.9411	1	1	1	0.118191861	1.6763
**Protein RGD1565486 RGD1565486**	tr|D3Z8R4|D3Z8R4_RAT	56 kDa	1	2.0878	1.9411	1	1	1	0.118191861	1.6763
**Nola2 protein Nhp2**	tr|B1WC56|B1WC56_RAT	17 kDa	1	2.0878	1.9411	1	1	1	0.118191861	1.6763
**Histone deacetylase (Fragment) Hdac1l**	tr|D3ZVU7|D3ZVU7_RAT (+1)	56 kDa	1	2.0878	1.9411	1	1	1	0.118191861	1.6763
**Cbx5 protein Cbx5**	tr|B2RYU7|B2RYU7_RAT	22 kDa	1	2.0878	1.9411	1	1	1	0.118191861	1.6763
**Rho guanine nucleotide exchange factor 7 Arhgef7**	sp|O55043|ARHG7_RAT (+1)	73 kDa	5.077	4.1756	3.8821	3.0063	2.8586	2.0419	0.020435161	1.661190368
**Phosphate carrier protein, mitochondrial Slc25a3**	tr|G3V741|G3V741_RAT	40 kDa	5.077	4.1756	8.7347	3.0063	4.7643	3.0629	0.189105737	1.66034061
**2-oxoisovalerate dehydrogenase subunit alpha, mitochondrial (Fragment) Bckdha**	sp|P11960|ODBA_RAT (+1)	50 kDa	2.0308	1	1.9411	1	1	1	0.116938926	1.6573
**Protein Snrpd1 Snrpd1**	tr|B2RZB7|B2RZB7_RAT	13 kDa	2.0308	1	1.9411	1	1	1	0.116938926	1.6573
**ADP-sugar pyrophosphatase Nudt5**	sp|Q6AY63|NUDT5_RAT	24 kDa	2.0308	1	1.9411	1	1	1	0.116938926	1.6573
**Synaptosomal-associated protein 29 Snap29**	sp|Q9Z2P6|SNP29_RAT	29 kDa	2.0308	1	1.9411	1	1	1	0.116938926	1.6573
**Acyl-coenzyme A thioesterase 8 Acot8**	sp|Q8VHK0|ACOT8_RAT (+1)	36 kDa	2.0308	1	1.9411	1	1	1	0.116938926	1.6573
**Serine/threonine-protein kinase WNK4 Wnk4**	tr|D3Z8M2|D3Z8M2_RAT	173 kDa	2.0308	1	1.9411	1	1	1	0.116938926	1.6573
**Ragulator complex protein LAMTOR1 Lamtor1**	sp|Q6P791|LTOR1_RAT	18 kDa	2.0308	1	1.9411	1	1	1	0.116938926	1.6573
**Protein Akr1b8 Akr1b8**	tr|G3V786|G3V786_RAT	36 kDa	2.0308	1	1.9411	1	1	1	0.116938926	1.6573
**Guanine nucleotide-binding protein subunit gamma Gng10**	tr|Q3KRE3|Q3KRE3_RAT	7 kDa	2.0308	1	1.9411	1	1	1	0.116938926	1.6573
**40S ribosomal protein S3 Rps3**	sp|P62909|RS3_RAT	27 kDa	14.216	13.571	11.646	8.0167	7.6229	8.1677	0.002726518	1.656340702
**Protein Cmpk2 Cmpk2**	tr|D3ZC63|D3ZC63_RAT	47 kDa	8.1232	4.1756	5.8232	4.0083	2.8586	4.0839	0.119733759	1.654856266
**Acidic leucine-rich nuclear phosphoprotein 32 family member A Anp32a**	sp|P49911|AN32A_RAT (+2)	29 kDa	3.0462	3.1317	1.9411	2.0042	1.9057	1	0.098743991	1.653597833
**Protein Sf1 Sf1**	tr|F1LM37|F1LM37_RAT (+1)	60 kDa	3.0462	3.1317	1.9411	2.0042	1.9057	1	0.098743991	1.653597833
**Four and a half LIM domains protein 1 Fhl1**	sp|Q9WUH4|FHL1_RAT (+1)	32 kDa	14.216	16.703	11.646	9.0188	8.5757	8.1677	0.019380577	1.652226906
**Alpha-actinin-1 Actn1**	sp|Q9Z1P2|ACTN1_RAT	103 kDa	15.231	20.878	19.411	11.023	10.481	12.252	0.01489628	1.644744638
**Basic transcription factor 3 Btf3**	tr|Q5U3Y8|Q5U3Y8_RAT	18 kDa	2.0308	3.1317	2.9116	2.0042	1.9057	1	0.085412753	1.644453044
**Paraspeckle component 1 Pspc1**	sp|Q4KLH4|PSPC1_RAT	59 kDa	2.0308	3.1317	2.9116	2.0042	1.9057	1	0.085412753	1.644453044
**Protein Tardbp Tardbp**	tr|I6L9G6|I6L9G6_RAT	32 kDa	6.0924	8.3513	6.7937	4.0083	3.8114	5.1048	0.023687189	1.643189292
**Collagen alpha-1(XII) chain (Fragment) Col12a1**	tr|F1LQC3|F1LQC3_RAT	210 kDa	16.246	17.746	11.646	9.0188	8.5757	10.21	0.03516959	1.641388984
**Coronin Coro1b**	tr|G3V940|G3V940_RAT	54 kDa	8.1232	9.3952	6.7937	4.0083	5.7171	5.1048	0.024820865	1.63936427
**Ras-related protein Rab-4A Rab4a**	sp|P05714|RAB4A_RAT (+1)	24 kDa	1	1	2.9116	1	1	1	0.373900966	1.6372
**Isoform GLAST-1A of Excitatory amino acid transporter 1 Slc1a3**	sp|P24942-2|EAA1_RAT (+2)	54 kDa	1	1	2.9116	1	1	1	0.373900966	1.6372
**Osteopontin Spp1**	sp|P08721|OSTP_RAT	35 kDa	1	1	2.9116	1	1	1	0.373900966	1.6372
**Carboxymethylenebutenolidase homolog Cmbl**	sp|Q7TP52|CMBL_RAT	28 kDa	1	1	2.9116	1	1	1	0.373900966	1.6372
**Glutaredoxin-1 Glrx**	sp|Q9ESH6|GLRX1_RAT	12 kDa	1	1	2.9116	1	1	1	0.373900966	1.6372
**Argininosuccinate lyase Asl**	sp|P20673|ARLY_RAT	52 kDa	1	1	2.9116	1	1	1	0.373900966	1.6372
**Isoform II of V-type proton ATPase 116 kDa subunit a isoform 1 Atp6v0a1**	sp|P25286-2|VPP1_RAT (+2)	96 kDa	1	1	2.9116	1	1	1	0.373900966	1.6372
**ER membrane protein complex subunit 3 Emc3**	sp|Q5U2V8|EMC3_RAT	30 kDa	1	1	2.9116	1	1	1	0.373900966	1.6372
**Isoform 1 of CD44 antigen Cd44**	sp|P26051-2|CD44_RAT (+4)	40 kDa	1	1	2.9116	1	1	1	0.373900966	1.6372
**Ab2-417 Tf**	tr|Q7TMC7|Q7TMC7_RAT	107 kDa	36.554	42.8	33.968	19.04	25.727	24.503	0.011620322	1.635946297
**Far upstream element-binding protein 2 Khsrp**	sp|Q99PF5|FUBP2_RAT (+1)	74 kDa	7.1078	6.2634	5.8232	2.0042	6.67	3.0629	0.164241537	1.635361375
**Protein LOC100362069 Rpl28**	tr|Q642E2|Q642E2_RAT	16 kDa	4.0616	2.0878	1.9411	1	1.9057	2.0419	0.239034076	1.635237287
**Isoform 2 of 40S ribosomal protein S24 Rps24**	sp|P62850-2|RS24_RAT (+3)	15 kDa	2.0308	3.1317	2.9116	1	1.9057	2.0419	0.090438177	1.631922548
**Acyl-coenzyme A thioesterase 2, mitochondrial Acot2**	sp|O55171|ACOT2_RAT (+1)	50 kDa	2.0308	3.1317	2.9116	1	1.9057	2.0419	0.090438177	1.631922548
**Septin-9 (Fragment) Sept9**	tr|F1LN75|F1LN75_RAT	64 kDa	14.216	14.615	16.499	10.021	8.5757	9.1887	0.002035849	1.631432335
**Serotransferrin Tf**	sp|P12346|TRFE_RAT	76 kDa	37.57	43.844	32.998	19.04	26.68	24.503	0.019150176	1.629266764
**Cold shock domain-containing protein E1 Csde1**	sp|P18395|CSDE1_RAT	89 kDa	3.0462	2.0878	2.9116	1	1.9057	2.0419	0.08036643	1.62616218
**Heme oxygenase 2 Hmox2**	sp|P23711|HMOX2_RAT	36 kDa	3.0462	2.0878	2.9116	1	1.9057	2.0419	0.08036643	1.62616218
**ADP-ribosylation factor-like protein 8B Arl8b**	sp|Q66HA6|ARL8B_RAT	22 kDa	8.1232	7.3073	8.7347	1	5.7171	8.1677	0.22246372	1.623481673
**Purine nucleoside phosphorylase Pnp**	sp|P85973|PNPH_RAT (+1)	32 kDa	24.37	20.878	22.322	13.027	13.34	15.314	0.002243706	1.621122334
**LOC683667 protein Sri**	tr|B0BNJ1|B0BNJ1_RAT	22 kDa	6.0924	5.2195	4.8526	4.0083	1.9057	4.0839	0.062637826	1.616789526
**Protein disulfide-isomerase A3 Pdia3**	sp|P11598|PDIA3_RAT	57 kDa	40.616	40.712	40.762	25.052	21.916	28.587	0.00129406	1.615908941
**2,4-dienoyl-CoA reductase, mitochondrial Decr1**	sp|Q64591|DECR_RAT (+1)	36 kDa	10.154	8.3513	8.7347	6.0125	4.7643	6.1258	0.007939583	1.611586383
**Proteasome subunit alpha type Psma7**	tr|F1LSQ6|F1LSQ6_RAT	28 kDa	9.1386	8.3513	9.7053	7.0146	4.7643	5.1048	0.012833866	1.610736983
**Glial fibrillary acidic protein Gfap**	sp|P47819|GFAP_RAT	50 kDa	249.79	210.87	252.34	140.29	141.98	171.52	0.006804233	1.571211353
**Isoform Glt-1A of Excitatory amino acid transporter 2 Slc1a2**	sp|P31596-2|EAA2_RAT (+1)	62 kDa	15.231	10.439	15.528	6.0125	10.481	9.1887	0.070980324	1.604146062
**Reticulocalbin-2 Rcn2**	sp|Q62703|RCN2_RAT	37 kDa	14.216	14.615	15.528	10.021	9.5286	8.1677	0.001206704	1.600408409
**Ras-related protein Rab-11B Rab11b**	sp|O35509|RB11B_RAT	24 kDa	7.1078	8.3513	6.7937	6.0125	3.8114	4.0839	0.029644651	1.600023009
**Endoplasmic reticulum resident protein 29 Erp29**	sp|P52555|ERP29_RAT	29 kDa	9.1386	9.3952	6.7937	6.0125	4.7643	5.1048	0.025610174	1.594770048
**C-1-tetrahydrofolate synthase, cytoplasmic Mthfd1**	tr|G3V6S5|G3V6S5_RAT	101 kDa	18.277	13.571	15.528	10.021	9.5286	10.21	0.013101723	1.591956881
**Staphylococcal nuclease domain-containing protein 1 Snd1**	sp|Q66X93|SND1_RAT	102 kDa	16.246	18.79	13.587	9.0188	10.481	11.231	0.021880356	1.582223697
**Annexin A5 Anxa5**	sp|P14668|ANXA5_RAT	36 kDa	23.354	21.922	17.469	15.031	11.434	13.273	0.020224628	1.578967235
**sp|K1C10_HUMAN|**	zz|ZZ_FGCZCont0160|	60 kDa	21.323	16.703	18.44	13.027	9.5286	13.273	0.019105713	1.576003528
**26S protease regulatory subunit 7 Psmc2**	tr|G3V7L6|G3V7L6_RAT	49 kDa	13.2	9.3952	6.7937	5.0104	8.5757	5.1048	0.180220374	1.572364092
**40S ribosomal protein S6 Rps6**	sp|P62755|RS6_RAT	29 kDa	8.1232	6.2634	5.8232	4.0083	4.7643	4.0839	0.030178009	1.571951931
**Acyl-Coenzyme A dehydrogenase, very long chain Acadvl**	tr|Q5M9H2|Q5M9H2_RAT	71 kDa	11.169	11.483	6.7937	6.0125	6.67	6.1258	0.080956549	1.565569456
**Heterogeneous nuclear ribonucleoproteins A2/B1 (Fragment) Hnrnpa2b1**	tr|F1LNF1|F1LNF1_RAT	37 kDa	29.447	29.229	28.145	20.042	18.104	17.356	0.00031005	1.564285972
**4F2 cell-surface antigen heavy chain Slc3a2**	sp|Q794F9|4F2_RAT	58 kDa	14.216	11.483	14.558	8.0167	8.5757	9.1887	0.009420798	1.561492721
**Tropomyosin alpha-4 chain Tpm4**	sp|P09495|TPM4_RAT	29 kDa	19.293	21.922	17.469	14.029	12.387	11.231	0.010072359	1.558796186
**Acyl-protein thioesterase 1 Lypla1**	sp|P70470|LYPA1_RAT	25 kDa	1	3.1317	1.9411	1	1.9057	1	0.352174588	1.554855724
**Guanine nucleotide-binding protein subunit beta-2-like 1 Gnb2l1**	sp|P63245|GBLP_RAT	35 kDa	14.216	12.527	11.646	9.0188	8.5757	7.1467	0.008469213	1.551622395
**NADH dehydrogenase (Ubiquinone) flavoprotein 3-like, isoform CRA_a Ndufv3**	tr|G3V644|G3V644_RAT	49 kDa	10.154	10.439	8.7347	10.021	3.8114	5.1048	0.152525673	1.548681959
**60S ribosomal protein L13 Rpl13**	sp|P41123|RL13_RAT (+1)	24 kDa	5.077	8.3513	4.8526	4.0083	4.7643	3.0629	0.156427518	1.544581978
**Nucleolar protein 3 Nol3**	sp|Q62881|NOL3_RAT (+1)	25 kDa	3.0462	5.2195	3.8821	1	3.8114	3.0629	0.247250941	1.542714908
**Protein Snta1 Snta1**	tr|B5DFL0|B5DFL0_RAT	53 kDa	2.0308	3.1317	3.8821	2.0042	2.8586	1	0.235312191	1.542709968
**Protein Hnrpll Hnrpll**	tr|D4A3E1|D4A3E1_RAT	64 kDa	1	4.1756	1	2.0042	1	1	0.549994601	1.542280605
**Heterogeneous nuclear ribonucleoprotein H1, isoform CRA_b Hnrph1**	tr|G3V9Q3|G3V9Q3_RAT	49 kDa	12.185	12.527	10.676	8.0167	4.7643	10.21	0.069823593	1.539210996
**Neurocan core protein Ncan**	sp|P55067|NCAN_RAT (+1)	136 kDa	9.1386	2.0878	14.558	3.0063	7.6229	6.1258	0.478859556	1.538907789
**Enoyl-CoA delta isomerase 2, mitochondrial Eci2**	sp|Q5XIC0|ECI2_RAT	43 kDa	6.0924	8.3513	6.7937	4.0083	5.7171	4.0839	0.046531091	1.537905614
**60S ribosomal protein L34 Rpl34**	tr|B2RZD4|B2RZD4_RAT (+2)	13 kDa	3.0462	3.1317	2.9116	3.0063	1.9057	1	0.143731899	1.537466171
**Eukaryotic translation initiation factor 5 Eif5**	sp|Q07205|IF5_RAT	49 kDa	5.077	5.2195	4.8526	4.0083	3.8114	2.0419	0.04988568	1.536170601
**Isoform 2 of Catechol O-methyltransferase Comt**	sp|P22734-2|COMT_RAT (+1)	25 kDa	5.077	4.1756	2.9116	2.0042	2.8586	3.0629	0.116254034	1.534779262
**Protein Snx12 Snx12**	tr|D4A719|D4A719_RAT	20 kDa	2.0308	4.1756	2.9116	2.0042	1.9057	2.0419	0.165903238	1.531973521
**60S ribosomal protein L7 Rpl7**	tr|B0K031|B0K031_RAT	30 kDa	7.1078	8.3513	5.8232	3.0063	4.7643	6.1258	0.101282148	1.531497366
**Sorting nexin-1 Snx1**	sp|Q99N27|SNX1_RAT	59 kDa	8.1232	5.2195	4.8526	5.0104	3.8114	3.0629	0.14916199	1.530985216
**Protein RGD1309586 RGD1309586**	tr|D3ZN21|D3ZN21_RAT	73 kDa	7.1078	8.3513	8.7347	4.0083	5.7171	6.1258	0.026815541	1.526307157
**Dipeptidyl peptidase 2 Dpp7**	sp|Q9EPB1|DPP2_RAT	55 kDa	5.077	4.1756	2.9116	3.0063	1.9057	3.0629	0.129205661	1.525310662
**Protein Ktn1 Ktn1**	tr|D4A4Z9|D4A4Z9_RAT (+2)	152 kDa	2.0308	6.2634	6.7937	2.0042	3.8114	4.0839	0.351705639	1.524107278
**Nitrilase 1, isoform CRA_a Nit1**	tr|F7ESM5|F7ESM5_RAT	32 kDa	2.0308	2.0878	1.9411	2.0042	1	1	0.112155136	1.513335997
**60S ribosomal protein L3 Rpl3**	sp|P21531|RL3_RAT	46 kDa	10.154	11.483	12.617	7.0146	8.5757	7.1467	0.011563552	1.506531205
**Isoform 2 of Septin-11 Sept11**	sp|B3GNI6-2|SEP11_RAT (+2)	50 kDa	21.323	18.79	16.499	12.025	13.34	12.252	0.012029273	1.504957865
**Septin-9 Sept9**	sp|Q9QZR6|SEPT9_RAT	64 kDa	16.246	14.615	18.44	12.025	9.5286	11.231	0.014401836	1.503785314
**Cytosolic non-specific dipeptidase Cndp2**	sp|Q6Q0N1|CNDP2_RAT	53 kDa	18.277	17.746	17.469	11.023	13.34	11.231	0.001548208	1.502837557
**Eukaryotic translation initiation factor 1A, Y-linked Eif1ax**	tr|B5DF60|B5DF60_RAT	16 kDa	1	3.1317	1.9411	1	1	2.0419	0.393031907	1.502461714
**Alpha-crystallin B chain Cryab**	sp|P23928|CRYAB_RAT	20 kDa	13.2	10.439	13.587	8.0167	7.6229	9.1887	0.019655507	1.49933745
**MARCKS-related protein Marcksl1**	sp|Q9EPH2|MRP_RAT	20 kDa	2.0308	2.0878	1.9411	1	1	2.0419	0.126952237	1.499220664
**Enthoprotin Clint1**	tr|Q6DGF2|Q6DGF2_RAT	52 kDa	2.0308	2.0878	1.9411	1	1	2.0419	0.126952237	1.499220664
**Protein Tial1 Tial1**	tr|Q5BJN3|Q5BJN3_RAT	43 kDa	2.0308	2.0878	1.9411	1	1	2.0419	0.126952237	1.499220664
**Centaurin, gamma 2 (Predicted) Agap1**	tr|G3V9U1|G3V9U1_RAT	74 kDa	1	2.0878	2.9116	2.0042	1	1	0.362023617	1.498276809
**60S ribosomal protein L24 Rpl24**	sp|P83732|RL24_RAT	18 kDa	6.0924	8.3513	4.8526	3.0063	3.8114	6.1258	0.201471614	1.490810059
**LETM1 and EF-hand domain-containing protein 1, mitochondrial Letm1**	sp|Q5XIN6|LETM1_RAT	83 kDa	3.0462	2.0878	3.8821	3.0063	1	2.0419	0.272095993	1.490707979
**Actin-binding LIM protein 2 Ablim2**	sp|Q6KC51|ABLM2_RAT	68 kDa	13.2	5.2195	9.7053	8.0167	4.7643	6.1258	0.285541572	1.487549453
**ARF GTPase-activating protein GIT1 Git1**	sp|Q9Z272|GIT1_RAT	85 kDa	3.0462	4.1756	2.9116	2.0042	3.8114	1	0.293346448	1.486795
**Histone H2B Hist1h2bo**	tr|D3ZNH4|D3ZNH4_RAT (+5)	15 kDa	23.354	30.273	24.263	16.033	19.057	17.356	0.022284985	1.485146627
**Protein LOC100910272 Srp72**	tr|B0BND9|B0BND9_RAT	25 kDa	1	2.0878	2.9116	1	1	2.0419	0.374562915	1.484301937
**Protein Ckap5 Ckap5**	tr|F1M0X7|F1M0X7_RAT (+1)	219 kDa	1	2.0878	2.9116	1	1	2.0419	0.374562915	1.484301937
**Plectin (Fragment) Plec**	tr|F1MAL6|F1MAL6_RAT (+2)	518 kDa	127.94	138.84	140.73	89.186	95.286	91.887	0.000555737	1.474567501
**Protein Srsf7 Srsf7**	tr|D4A720|D4A720_RAT	27 kDa	2.0308	4.1756	3.8821	1	3.8114	2.0419	0.366613598	1.472064553
**ATPase family AAA domain-containing protein 3 Atad3**	sp|Q3KRE0|ATAD3_RAT	67 kDa	3.0462	1	4.8526	3.0063	1	2.0419	0.490999229	1.471313779
**40S ribosomal protein S16 Rps16**	sp|P62250|RS16_RAT	16 kDa	5.077	4.1756	3.8821	3.0063	2.8586	3.0629	0.018368276	1.471213513
**Tropomodulin 1 Tmod1**	tr|Q6IMZ5|Q6IMZ5_RAT (+1)	40 kDa	2.0308	1	2.9116	1	1	2.0419	0.386587058	1.470199659

Ubiquitin-fold modifier-conjugating enzyme 1 Ufc1	sp|Q6BBI8|UFC1_RAT	19 kDa	2.0308	1	2.9116	1	1	2.0419	0.386587058	1.470199659
Protein Trove2 Trove2	tr|D3ZRN5|D3ZRN5_RAT	60 kDa	3.0462	2.0878	4.8526	2.0042	3.8114	1	0.411815503	1.465256177
Transaldolase Taldo1	sp|Q9EQS0|TALDO_RAT	37 kDa	9.1386	11.483	12.617	8.0167	8.5757	6.1258	0.050138359	1.463082463
Proteasome subunit beta type Psmb4	tr|G3V8U9|G3V8U9_RAT	29 kDa	6.0924	7.3073	6.7937	4.0083	5.7171	4.0839	0.032047816	1.462304389
Beta-arrestin-1 Arrb1	sp|P29066|ARRB1_RAT	47 kDa	3.0462	5.2195	4.8526	4.0083	1.9057	3.0629	0.202255429	1.461339661
Adenine phosphoribosyltransferase Aprt	sp|P36972|APT_RAT	20 kDa	8.1232	6.2634	8.7347	5.0104	5.7171	5.1048	0.035073469	1.460387941
Protein Gpd1l Gpd1l	tr|D3ZAP9|D3ZAP9_RAT	38 kDa	3.0462	4.1756	2.9116	2.0042	1.9057	3.0629	0.125748772	1.453275585
Cystatin-C Cst3	sp|P14841|CYTC_RAT	15 kDa	7.1078	6.2634	6.7937	4.0083	4.7643	5.1048	0.006750947	1.453074783
60S ribosomal protein L5 Rpl5	sp|P09895|RL5_RAT	34 kDa	6.0924	6.2634	4.8526	3.0063	4.7643	4.0839	0.058024291	1.4516344
DEAD (Asp-Glu-Ala-Asp) box polypeptide 5 Ddx5	tr|Q6AYI1|Q6AYI1_RAT	69 kDa	13.2	11.483	9.7053	10.021	7.6229	6.1258	0.080122222	1.446728398
Aldose reductase Akr1b1	sp|P07943|ALDR_RAT	36 kDa	12.185	11.483	10.676	7.0146	8.5757	8.1677	0.005257107	1.445576227
Uncharacterized protein	tr|F1LZX9|F1LZX9_RAT	115 kDa	4.0616	2.0878	1	1	1.9057	2.0419	0.484549318	1.44502385
Myosin regulatory light chain RLC-A Rlc-a	sp|P13832|MRLCA_RAT	20 kDa	7.1078	8.3513	5.8232	4.0083	5.7171	5.1048	0.071880387	1.435064935
Sodium/potassium-transporting ATPase subunit beta-2 Atp1b2	sp|P13638|AT1B2_RAT (+1)	33 kDa	7.1078	6.2634	10.676	4.0083	6.67	6.1258	0.200589434	1.431031712
60S ribosomal protein L13a Rpl13a	tr|Q5RK10|Q5RK10_RAT	23 kDa	4.0616	5.2195	1.9411	2.0042	3.8114	2.0419	0.37711221	1.428215081
DEAD/H (Asp-Glu-Ala-Asp/His) box polypeptide 3, X-linked Ddx3x	tr|D4ADE8|D4ADE8_RAT	34 kDa	6.0924	7.3073	7.7642	5.0104	3.8114	6.1258	0.067864876	1.415872782
60S ribosomal protein L10 Rpl10	sp|Q6PDV7|RL10_RAT	25 kDa	9.1386	7.3073	8.7347	6.0125	6.67	5.1048	0.026392738	1.415650492
CD9 antigen Cd9	sp|P40241|CD9_RAT	25 kDa	5.077	6.2634	6.7937	4.0083	4.7643	4.0839	0.035091112	1.410500525
40S ribosomal protein S2 Rps2	sp|P27952|RS2_RAT (+1)	31 kDa	11.169	11.483	6.7937	8.0167	5.7171	7.1467	0.159649759	1.410200905
Methionine aminopeptidase 2 Metap2	sp|P38062|AMPM2_RAT	53 kDa	5.077	3.1317	2.9116	3.0063	2.8586	2.0419	0.226710331	1.40642232
40S ribosomal protein S26 Rps26	sp|P62856|RS26_RAT	13 kDa	3.0462	2.0878	1.9411	2.0042	1	2.0419	0.23666393	1.402092705
G1 to S phase transition 1 Gspt1	tr|Q6AYD5|Q6AYD5_RAT	69 kDa	3.0462	2.0878	1.9411	2.0042	1	2.0419	0.23666393	1.402092705
Canopy 2 homolog (Zebrafish) Cnpy2	tr|A0JN30|A0JN30_RAT	21 kDa	3.0462	2.0878	1.9411	2.0042	1	2.0419	0.23666393	1.402092705
Cellular nucleic acid-binding protein Cnbp	sp|P62634|CNBP_RAT	19 kDa	3.0462	2.0878	1.9411	2.0042	1	2.0419	0.23666393	1.402092705
Uncharacterized protein	tr|D3ZEI4|D3ZEI4_RAT	47 kDa	6.0924	8.3513	7.7642	4.0083	5.7171	6.1258	0.086615534	1.401023266
Histone H3.3 H3f3b	sp|P84245|H33_RAT (+2)	15 kDa	7.1078	8.3513	6.7937	5.0104	4.7643	6.1258	0.028805361	1.39950316
Calreticulin Calr	sp|P18418|CALR_RAT	48 kDa	25.385	20.878	24.263	15.031	18.104	17.356	0.015209576	1.396803391
Alpha-actinin-4 Actn4	sp|Q9QXQ0|ACTN4_RAT	105 kDa	22.339	29.229	23.293	16.033	17.151	20.419	0.048511827	1.396582281
Thioredoxin Txn	sp|P11232|THIO_RAT	12 kDa	7.1078	7.3073	7.7642	6.0125	4.7643	5.1048	0.007508812	1.396540651
Inositol-3-phosphate synthase 1 Isyna1	sp|Q6AYK3|INO1_RAT	61 kDa	3.0462	4.1756	3.8821	3.0063	1.9057	3.0629	0.108445461	1.392356017
Leukocyte elastase inhibitor A Serpinb1a	sp|Q4G075|ILEUA_RAT	43 kDa	2.0308	4.1756	1.9411	2.0042	2.8586	1	0.448162693	1.389694344
CD81 antigen Cd81	tr|Q6P9V1|Q6P9V1_RAT	26 kDa	6.0924	4.1756	4.8526	4.0083	3.8114	3.0629	0.08867269	1.389428997
Vesicle-associated membrane protein-associated protein A Vapa	sp|Q9Z270|VAPA_RAT	28 kDa	8.1232	8.3513	9.7053	7.0146	5.7171	6.1258	0.017454459	1.388296434
Proteasome subunit alpha type-1 Psma1	sp|P18420|PSA1_RAT	30 kDa	8.1232	8.3513	6.7937	5.0104	6.67	5.1048	0.040695242	1.38623311
Protein Zyx Zyx	tr|D4A7U1|D4A7U1_RAT	60 kDa	4.0616	5.2195	2.9116	2.0042	4.7643	2.0419	0.375217578	1.383898574
Histone H1.4 Hist1h1e	sp|P15865|H14_RAT	22 kDa	14.216	15.659	12.617	10.021	10.481	10.21	0.011521228	1.383563428
CD59 glycoprotein Cd59	sp|P27274|CD59_RAT	14 kDa	5.077	5.2195	4.8526	4.0083	2.8586	4.0839	0.027063591	1.383378383
Protein Susd2 Susd2	tr|D3ZEV8|D3ZEV8_RAT	90 kDa	3.0462	3.1317	4.8526	3.0063	1.9057	3.0629	0.218611631	1.38315214
Profilin-1 Pfn1	sp|P62963|PROF1_RAT	15 kDa	12.185	17.746	12.617	10.021	9.5286	11.231	0.102132872	1.38229924
Glutathione peroxidase 1 Gpx1	sp|P04041|GPX1_RAT	22 kDa	10.154	9.3952	7.7642	9.0188	5.7171	5.1048	0.150975991	1.376634897
60S ribosomal protein L30 Rpl30	sp|P62890|RL30_RAT	13 kDa	4.0616	5.2195	2.9116	3.0063	3.8114	2.0419	0.25647255	1.376213373
gi|125105|sp|P13647| K2C5_HUMAN KERATIN, TYPE II CYTOSKELETAL 5 (CYTOK	zz|ZZ_FGCZCont0251|	62 kDa	6.0924	1	3.8821	6.0125	1	1	0.680608971	1.369672387
Peroxisomal trans-2-enoyl-CoA reductase Pecr	sp|Q9WVK3|PECR_RAT	32 kDa	2.0308	4.1756	1.9411	2.0042	1.9057	2.0419	0.373648812	1.368913606
60S ribosomal protein L27a Rpl27a	sp|P18445|RL27A_RAT (+1)	17 kDa	2.0308	3.1317	2.9116	1	2.8586	2.0419	0.317110023	1.368375561
Eukaryotic translation initiation factor 3 subunit J Eif3j	sp|A0JPM9|EIF3J_RAT	29 kDa	5.077	6.2634	4.8526	4.0083	4.7643	3.0629	0.092104157	1.368172025
Adenylyl cyclase-associated protein 2 Cap2	sp|P52481|CAP2_RAT	53 kDa	4.0616	1	5.8232	3.0063	1.9057	3.0629	0.542423146	1.364882318
Alpha-adducin Add1	sp|Q63028|ADDA_RAT	80 kDa	24.37	26.098	25.234	18.038	19.057	18.377	0.000316774	1.364688491
Sorting nexin-3 Snx3	sp|Q5U211|SNX3_RAT	19 kDa	3.0462	3.1317	1.9411	2.0042	1.9057	2.0419	0.134261779	1.364125139
Cytochrome c oxidase subunit 4 isoform 1, mitochondrial Cox4i1	sp|P10888|COX41_RAT	20 kDa	10.154	11.483	10.676	10.021	8.5757	5.1048	0.129927184	1.363331435
Protein kinase C delta type Prkcd	sp|P09215|KPCD_RAT	78 kDa	1	2.0878	1	1	1	1	0.373900966	1.3626
Protein Tenc1 Tenc1	tr|D4ADU3|D4ADU3_RAT (+1)	152 kDa	1	2.0878	1	1	1	1	0.373900966	1.3626
Protein Etl4 (Fragment) Etl4	tr|F1M6H0|F1M6H0_RAT (+2)	206 kDa	1	2.0878	1	1	1	1	0.373900966	1.3626
Allograft inflammatory factor 1 Aif1	sp|P55009|AIF1_RAT	17 kDa	1	2.0878	1	1	1	1	0.373900966	1.3626
60S ribosomal protein L18a Rpl18a	sp|P62718|RL18A_RAT	21 kDa	1	2.0878	1	1	1	1	0.373900966	1.3626
Misshapen-like kinase 1 Mink1	sp|F1LP90|MINK1_RAT (+1)	150 kDa	1	2.0878	1	1	1	1	0.373900966	1.3626
Kinesin 13B Kif13b	tr|Q70AM4|Q70AM4_RAT	197 kDa	1	2.0878	1	1	1	1	0.373900966	1.3626
Methionine adenosyltransferase 2 subunit beta Mat2b	sp|Q5U2R0|MAT2B_RAT	37 kDa	1	2.0878	1	1	1	1	0.373900966	1.3626
Mpp6 protein Mpp6	tr|B5DFE0|B5DFE0_RAT	61 kDa	1	2.0878	1	1	1	1	0.373900966	1.3626
Protein Sphk2 Sphk2	tr|Q6AYB2|Q6AYB2_RAT	66 kDa	1	2.0878	1	1	1	1	0.373900966	1.3626
Cytosolic Fe-S cluster assembly factor NUBP2 Nubp2	sp|Q68FS1|NUBP2_RAT	29 kDa	1	2.0878	1	1	1	1	0.373900966	1.3626
Parathymosin Ptms	sp|P04550|PTMS_RAT	12 kDa	1	2.0878	1	1	1	1	0.373900966	1.3626
40S ribosomal protein S21 Rps21	sp|P05765|RS21_RAT (+1)	9 kDa	1	2.0878	1	1	1	1	0.373900966	1.3626
Aa2-277 Mrpl49	tr|Q7TP77|Q7TP77_RAT	19 kDa	1	2.0878	1	1	1	1	0.373900966	1.3626
Protein Pnn Pnn	tr|D3ZAY8|D3ZAY8_RAT	83 kDa	1	2.0878	1	1	1	1	0.373900966	1.3626
Protein Zfp207 Zfp207	tr|Q498C9|Q498C9_RAT	50 kDa	1	2.0878	1	1	1	1	0.373900966	1.3626
N-acetylgalactosamine-6-sulfatase Galns	sp|Q32KJ6|GALNS_RAT	58 kDa	1	2.0878	1	1	1	1	0.373900966	1.3626
m7GpppX diphosphatase Dcps	sp|Q8K4F7|DCPS_RAT (+1)	39 kDa	1	2.0878	1	1	1	1	0.373900966	1.3626
Dipeptidyl peptidase 4 Dpp4	tr|F1M7X5|F1M7X5_RAT (+1)	84 kDa	1	2.0878	1	1	1	1	0.373900966	1.3626
E3 ubiquitin-protein ligase NEDD4 Nedd4	sp|Q62940|NEDD4_RAT	102 kDa	1	2.0878	1	1	1	1	0.373900966	1.3626
Aminopeptidase N Anpep	sp|P15684|AMPN_RAT (+1)	109 kDa	1	2.0878	1	1	1	1	0.373900966	1.3626
Isoform 2 of Fragile X mental retardation protein 1 homolog Fmr1	sp|Q80WE1-2|FMR1_RAT (+1)	66 kDa	1	2.0878	1	1	1	1	0.373900966	1.3626
Protein Chchd2 Chchd2	tr|M0R785|M0R785_RAT	16 kDa	1	2.0878	1	1	1	1	0.373900966	1.3626
Protein Trim25 Trim25	tr|D4A9N5|D4A9N5_RAT	45 kDa	1	2.0878	1	1	1	1	0.373900966	1.3626
Vesicle transport through interaction with t-SNAREs homolog 1B Vti1b	sp|P58200|VTI1B_RAT (+1)	27 kDa	1	2.0878	1	1	1	1	0.373900966	1.3626
Pitrilysin metallepetidase 1 (Predicted) Pitrm1	tr|D3ZUF9|D3ZUF9_RAT	109 kDa	1	2.0878	1	1	1	1	0.373900966	1.3626
Protein Fam120a Fam120a	tr|D4AB03|D4AB03_RAT	122 kDa	1	2.0878	1	1	1	1	0.373900966	1.3626
Neutrophil cytosolic factor 1 Ncf1	tr|F1M707|F1M707_RAT	45 kDa	1	2.0878	1	1	1	1	0.373900966	1.3626
Isoform 2 of General transcription factor II-I Gtf2i	sp|Q5U2Y1-2|GTF2I_RAT (+1)	103 kDa	1	2.0878	1	1	1	1	0.373900966	1.3626
F-box only protein 6 Fbxo6	sp|Q923V4|FBX6_RAT	33 kDa	1	2.0878	1	1	1	1	0.373900966	1.3626
Isoform UBF2 of Nucleolar transcription factor 1 Ubtf	sp|P25977-2|UBF1_RAT (+1)	85 kDa	1	2.0878	1	1	1	1	0.373900966	1.3626
Isoform 2 of Protein LYRIC Mtdh	sp|Q9Z1W6-2|LYRIC_RAT (+4)	62 kDa	1	2.0878	1	1	1	1	0.373900966	1.3626
Actin-like 6A Actl6a	tr|Q4KM87|Q4KM87_RAT	47 kDa	1	2.0878	1	1	1	1	0.373900966	1.3626
Protein Ranbp3 Ranbp3	tr|M0R5Q3|M0R5Q3_RAT (+2)	52 kDa	1	2.0878	1	1	1	1	0.373900966	1.3626
Membrane-associated progesterone receptor component 2 Pgrmc2	sp|Q5XIU9|PGRC2_RAT	23 kDa	1	2.0878	1	1	1	1	0.373900966	1.3626
Protein Msi2 (Fragment) Msi2	tr|F1LWE6|F1LWE6_RAT	34 kDa	1	2.0878	1	1	1	1	0.373900966	1.3626
Protein Ntpcr Ntpcr	tr|D4A478|D4A478_RAT	21 kDa	1	2.0878	1	1	1	1	0.373900966	1.3626
Protein Sec61b Sec61b	tr|B2RZD1|B2RZD1_RAT	10 kDa	1	2.0878	1	1	1	1	0.373900966	1.3626
Isoform 2 of Hepatoma-derived growth factor-related protein 2 Hdgfrp2	sp|Q925G1-2|HDGR2_RAT (+1)	74 kDa	1	2.0878	1	1	1	1	0.373900966	1.3626
Sulfotransferase 1A1 Sult1a1	sp|P17988|ST1A1_RAT	34 kDa	1	2.0878	1	1	1	1	0.373900966	1.3626
3-alpha-hydroxysteroid dehydrogenase Akr1c9	sp|P23457|DIDH_RAT	37 kDa	1	2.0878	1	1	1	1	0.373900966	1.3626
Isoform 2 of Haptoglobin Hp	sp|P06866-2|HPT_RAT (+1)	42 kDa	1	2.0878	1	1	1	1	0.373900966	1.3626
Mitochondrial fission factor Mff	sp|Q4KM98|MFF_RAT	25 kDa	1	2.0878	1	1	1	1	0.373900966	1.3626
PTB domain-containing engulfment adapter protein 1 Gulp1	sp|Q5PQS4|GULP1_RAT (+2)	34 kDa	1	2.0878	1	1	1	1	0.373900966	1.3626
Ragulator complex protein LAMTOR3 Lamtor3	sp|Q5U204|LTOR3_RAT	14 kDa	1	2.0878	1	1	1	1	0.373900966	1.3626
Cysteine sulfinic acid decarboxylase Csad	sp|Q64611|CSAD_RAT	55 kDa	1	2.0878	1	1	1	1	0.373900966	1.3626
H/ACA ribonucleoprotein complex subunit 1 Gar1	sp|Q6AYA1|GAR1_RAT	23 kDa	1	2.0878	1	1	1	1	0.373900966	1.3626
Protein Tnks1bp1 Tnks1bp1	tr|D3ZF26|D3ZF26_RAT	180 kDa	1	2.0878	1	1	1	1	0.373900966	1.3626
Protein arginine N-methyltransferase 1 Prmt1	sp|Q63009|ANM1_RAT	41 kDa	1	2.0878	1	1	1	1	0.373900966	1.3626
Protein Xrcc6 Xrcc6	tr|E9PT85|E9PT85_RAT (+1)	70 kDa	1	2.0878	1	1	1	1	0.373900966	1.3626
Methionine aminopeptidase Metap1	tr|D3ZE72|D3ZE72_RAT	43 kDa	1	2.0878	1	1	1	1	0.373900966	1.3626
Protein Vps37b Vps37b	tr|D3ZU64|D3ZU64_RAT	31 kDa	1	2.0878	1	1	1	1	0.373900966	1.3626
Myomesin 2 Myom2	tr|G3V7K1|G3V7K1_RAT	165 kDa	1	2.0878	1	1	1	1	0.373900966	1.3626
Cleavage and polyadenylation specific factor 6, 68kDa (Predicted), isoform CRA_b Cpsf6	tr|D3ZPL1|D3ZPL1_RAT	59 kDa	1	2.0878	1	1	1	1	0.373900966	1.3626
Growth arrest-specific protein 7 Gas7	sp|O55148|GAS7_RAT (+2)	48 kDa	1	2.0878	1	1	1	1	0.373900966	1.3626
Deoxyribonuclease-2-alpha Dnase2	sp|Q9QZK8|DNS2A_RAT	38 kDa	1	2.0878	1	1	1	1	0.373900966	1.3626
Transmembrane emp24 domain-containing protein 5 Tmed5	sp|Q6AXN3|TMED5_RAT	26 kDa	1	2.0878	1	1	1	1	0.373900966	1.3626
Golgi reassembly-stacking protein 2 Gorasp2	sp|Q9R064|GORS2_RAT (+1)	47 kDa	1	2.0878	1	1	1	1	0.373900966	1.3626
Choline-phosphate cytidylyltransferase A Pcyt1a	sp|P19836|PCY1A_RAT	42 kDa	1	2.0878	1	1	1	1	0.373900966	1.3626
Isoform 2 of Alpha-1B-glycoprotein A1bg	sp|Q9EPH1-2|A1BG_RAT (+1)	52 kDa	1	2.0878	1	1	1	1	0.373900966	1.3626
Protein Myoz1 Myoz1	tr|D4A7U8|D4A7U8_RAT	31 kDa	1	2.0878	1	1	1	1	0.373900966	1.3626
Poly(A) binding protein, nuclear 1, isoform CRA_a Pabpn1	tr|G3V7Z8|G3V7Z8_RAT	32 kDa	1	2.0878	1	1	1	1	0.373900966	1.3626
Protein Arap1 Arap1	tr|D4ABQ7|D4ABQ7_RAT (+1)	162 kDa	1	2.0878	1	1	1	1	0.373900966	1.3626
Phospholipase B-like 1 Plbd1	sp|Q5U2V4|PLBL1_RAT	63 kDa	1	2.0878	1	1	1	1	0.373900966	1.3626
GATA zinc finger domain containing 2B Gatad2b	tr|Q4V8E1|Q4V8E1_RAT	65 kDa	1	2.0878	1	1	1	1	0.373900966	1.3626
Protein H1fx H1fx	tr|D3ZIX4|D3ZIX4_RAT	20 kDa	1	2.0878	1	1	1	1	0.373900966	1.3626
Uncharacterized protein	tr|M0R8A9|M0R8A9_RAT (+1)	44 kDa	1	2.0878	1	1	1	1	0.373900966	1.3626
Heat shock protein beta-7 (Fragment) Hspb7	sp|Q9QUK5|HSPB7_RAT (+1)	10 kDa	1	2.0878	1	1	1	1	0.373900966	1.3626
Uncharacterized protein	tr|M0R7E6|M0R7E6_RAT	100 kDa	1	2.0878	1	1	1	1	0.373900966	1.3626
DnaJ homolog subfamily C member 10 Dnajc10	sp|Q498R3|DJC10_RAT	91 kDa	1	2.0878	1	1	1	1	0.373900966	1.3626
PDZ and LIM domain protein 3 Pdlim3	sp|Q66HS7|PDLI3_RAT (+1)	39 kDa	1	2.0878	1	1	1	1	0.373900966	1.3626
Serine/threonine-protein kinase DCLK1 Dclk1	sp|O08875|DCLK1_RAT	48 kDa	1	2.0878	1	1	1	1	0.373900966	1.3626
Protein RGD1310507 RGD1310507	tr|E9PST1|E9PST1_RAT	51 kDa	1	2.0878	1	1	1	1	0.373900966	1.3626

**Protein LOC684828 LOC684828**	tr|M0R7B4|M0R7B4_RAT	23 kDa	13.2	14.615	12.617	10.021	10.481	9.1887	0.007034203	1.361773215
**Isoform 2 of Actin-binding LIM protein 2 Ablim2**	sp|Q6KC51-2|ABLM2_RAT	64 kDa	12.185	5.2195	9.7053	8.0167	4.7643	7.1467	0.3488698	1.360407874
**D-amino-acid oxidase Dao**	sp|O35078|OXDA_RAT	39 kDa	2.0308	3.1317	2.9116	2.0042	1.9057	2.0419	0.105047503	1.356581202
**Latexin Lxn**	sp|Q64361|LXN_RAT	26 kDa	7.1078	6.2634	6.7937	5.0104	4.7643	5.1048	0.002713611	1.355213549
**Heterogeneous nuclear ribonucleoprotein H2 Hnrnph2**	sp|Q6AY09|HNRH2_RAT	49 kDa	9.1386	10.439	8.7347	7.0146	5.7171	8.1677	0.047616977	1.354694393
**Microtubule-associated protein RP/EB family member 1 Mapre1**	sp|Q66HR2|MARE1_RAT	30 kDa	5.077	5.2195	5.8232	4.0083	3.8114	4.0839	0.004421274	1.354186969
**Proteasome (Prosome, macropain) 26S subunit, non-ATPase, 3 Psmd3**	tr|Q5U2S7|Q5U2S7_RAT	61 kDa	4.0616	5.2195	6.7937	2.0042	4.7643	5.1048	0.329056101	1.353861184
**Microtubule-associated protein 4 Map4**	sp|Q5M7W5|MAP4_RAT	110 kDa	35.539	33.405	32.998	23.048	25.727	26.545	0.002532716	1.353451938
**60S ribosomal protein L19 Rpl19**	sp|P84100|RL19_RAT	23 kDa	3.0462	2.0878	2.9116	2.0042	1.9057	2.0419	0.082147639	1.351792735
**RCG24191 Sfxn1**	tr|Q6AYS2|Q6AYS2_RAT	36 kDa	5.077	1	4.8526	3.0063	1	4.0839	0.586483415	1.350967838
**Chromobox homolog 3 (HP1 gamma homolog, Drosophila) Cbx3**	tr|Q5RJK5|Q5RJK5_RAT	21 kDa	4.0616	4.1756	3.8821	4.0083	1.9057	3.0629	0.163190968	1.350054028
**FERM, RhoGEF (Arhgef) and pleckstrin domain protein 1 (Chondrocyte-derived) (Predicted) Farp1**	tr|F1LYQ8|F1LYQ8_RAT	119 kDa	3.0462	2.0878	2.9116	1	1.9057	3.0629	0.358497838	1.347987803
**Protein Myo6 Myo6**	tr|D4A5I9|D4A5I9_RAT	146 kDa	11.169	7.3073	13.587	8.0167	7.6229	8.1677	0.20824552	1.34678439
**Elongation factor 1-alpha 1 Eef1a1**	sp|P62630|EF1A1_RAT	50 kDa	43.662	38.625	41.733	29.061	30.491	32.671	0.004192963	1.344783839
**ATP synthase protein 8 Mt-atp8**	sp|P11608|ATP8_RAT	8 kDa	2.0308	1	1	1	1	1	0.373900966	1.3436
**Inter-alpha trypsin inhibitor, heavy chain 1 (Predicted), isoform CRA_a Itih1**	tr|B2RYM3|B2RYM3_RAT	101 kDa	2.0308	1	1	1	1	1	0.373900966	1.3436
**Isoform T1 of BDNF/NT-3 growth factors receptor Ntrk2**	sp|Q63604-2|NTRK2_RAT (+1)	53 kDa	2.0308	1	1	1	1	1	0.373900966	1.3436
**Fibrinogen alpha chain Fga**	tr|F7EUB6|F7EUB6_RAT (+1)	60 kDa	2.0308	1	1	1	1	1	0.373900966	1.3436
**Protein Srrm2 Srrm2**	tr|F1LRJ2|F1LRJ2_RAT	296 kDa	2.0308	1	1	1	1	1	0.373900966	1.3436
**E3 ubiquitin-protein ligase TRIM9 Trim9**	sp|Q91ZY8|TRIM9_RAT	79 kDa	2.0308	1	1	1	1	1	0.373900966	1.3436
**Chromatin modifying protein 2A Chmp2a**	tr|B2RZB5|B2RZB5_RAT	25 kDa	2.0308	1	1	1	1	1	0.373900966	1.3436
**Thioredoxin domain-containing protein 12 Txndc12**	sp|Q498E0|TXD12_RAT	19 kDa	2.0308	1	1	1	1	1	0.373900966	1.3436
**NHP2-like protein 1 Nhp2l1**	sp|P55770|NH2L1_RAT	14 kDa	2.0308	1	1	1	1	1	0.373900966	1.3436
**Prefoldin subunit 2 Pfdn2**	sp|B0BN18|PFD2_RAT	17 kDa	2.0308	1	1	1	1	1	0.373900966	1.3436
**40S ribosomal protein S29 Rps29**	sp|P62275|RS29_RAT	7 kDa	2.0308	1	1	1	1	1	0.373900966	1.3436
**Dystrobrevin beta Dtnb**	sp|P84060|DTNB_RAT	74 kDa	2.0308	1	1	1	1	1	0.373900966	1.3436
**Pleiotrophin Ptn**	sp|P63090|PTN_RAT	19 kDa	2.0308	1	1	1	1	1	0.373900966	1.3436
**Protein Rab23 Rab23**	tr|D3ZRM5|D3ZRM5_RAT	27 kDa	2.0308	1	1	1	1	1	0.373900966	1.3436
**Protein Lamtor2 Lamtor2**	tr|D4A465|D4A465_RAT	13 kDa	2.0308	1	1	1	1	1	0.373900966	1.3436
**Transcription elongation factor A protein 1 Tcea1**	sp|Q4KLL0|TCEA1_RAT	34 kDa	2.0308	1	1	1	1	1	0.373900966	1.3436
**Ester hydrolase C11orf54 homolog**	sp|Q5U2Q3|CK054_RAT	35 kDa	2.0308	1	1	1	1	1	0.373900966	1.3436
**Protein Snx18 Snx18**	tr|D3ZZ38|D3ZZ38_RAT	68 kDa	2.0308	1	1	1	1	1	0.373900966	1.3436
**Short/branched chain specific acyl-CoA dehydrogenase, mitochondrial Acadsb**	sp|P70584|ACDSB_RAT	48 kDa	2.0308	1	1	1	1	1	0.373900966	1.3436
**Dynein light chain Tctex-type 1 Dynlt1**	sp|Q9Z336|DYLT1_RAT	12 kDa	2.0308	1	1	1	1	1	0.373900966	1.3436
**Glutathione peroxidase Gpx7**	tr|D3ZQI1|D3ZQI1_RAT	21 kDa	2.0308	1	1	1	1	1	0.373900966	1.3436
**FACT complex subunit SSRP1 Ssrp1**	sp|Q04931|SSRP1_RAT	81 kDa	2.0308	1	1	1	1	1	0.373900966	1.3436
**Nuclear pore glycoprotein p62 Nup62**	sp|P17955|NUP62_RAT	53 kDa	2.0308	1	1	1	1	1	0.373900966	1.3436
**Complement C1q subcomponent subunit A C1qa**	sp|P31720|C1QA_RAT	26 kDa	2.0308	1	1	1	1	1	0.373900966	1.3436
**PYD and CARD domain containing Pycard**	tr|G3V8L1|G3V8L1_RAT	22 kDa	2.0308	1	1	1	1	1	0.373900966	1.3436
**Tenomodulin Tnmd**	sp|Q9ESC2|TNMD_RAT	37 kDa	2.0308	1	1	1	1	1	0.373900966	1.3436
**Disabled homolog 2 Dab2**	sp|O88797|DAB2_RAT (+2)	82 kDa	2.0308	1	1	1	1	1	0.373900966	1.3436
**Cell cycle control protein 50A Tmem30a**	sp|Q6AY41|CC50A_RAT	37 kDa	2.0308	1	1	1	1	1	0.373900966	1.3436
**Probable Xaa-Pro aminopeptidase 3 Xpnpep3**	sp|B5DEQ3|XPP3_RAT (+1)	56 kDa	2.0308	1	1	1	1	1	0.373900966	1.3436
**Protein Lamtor4 Lamtor4**	tr|D4AD29|D4AD29_RAT	11 kDa	2.0308	1	1	1	1	1	0.373900966	1.3436
**Pro-MCH Pmch**	sp|P14200|MCH_RAT (+1)	18 kDa	2.0308	1	1	1	1	1	0.373900966	1.3436
**Protein LOC690976 LOC690976**	tr|D3ZRX9|D3ZRX9_RAT	24 kDa	2.0308	1	1	1	1	1	0.373900966	1.3436
**Actin-related protein 2 Actr2**	sp|Q5M7U6|ARP2_RAT	45 kDa	9.1386	10.439	9.7053	6.0125	7.6229	8.1677	0.029082741	1.343061308
**cAMP-dependent protein kinase type I-alpha regulatory subunit Prkar1a**	sp|P09456|KAP0_RAT	43 kDa	19.293	11.483	14.558	11.023	10.481	12.252	0.173148009	1.342990876
**Protein LOC100360604 LOC100364176**	tr|D3ZPN7|D3ZPN7_RAT (+1)	19 kDa	3.0462	3.1317	1.9411	3.0063	1	2.0419	0.37664956	1.342382858
**Lysosomal alpha-glucosidase Gaa**	sp|Q6P7A9|LYAG_RAT	106 kDa	3.0462	3.1317	1.9411	3.0063	1	2.0419	0.37664956	1.342382858
**Protein Snx2 Snx2**	tr|B2RYP4|B2RYP4_RAT	59 kDa	6.0924	6.2634	4.8526	5.0104	4.7643	3.0629	0.126460046	1.340468623
**Isoform Short of Aquaporin-4 Aqp4**	sp|P47863-2|AQP4_RAT (+1)	29 kDa	6.0924	5.2195	5.8232	3.0063	5.7171	4.0839	0.156878485	1.337916657
**Isoform 3 of Heterogeneous nuclear ribonucleoprotein D0 Hnrnpd**	sp|Q9JJ54-3|HNRPD_RAT (+3)	33 kDa	9.1386	12.527	8.7347	10.021	7.6229	5.1048	0.242235269	1.336353286
**Serine/threonine-protein phosphatase PP1-alpha catalytic subunit Ppp1ca**	sp|P62138|PP1A_RAT	38 kDa	16.246	15.659	14.558	13.027	9.5286	12.252	0.029402454	1.334852159
**60S acidic ribosomal protein P0 Rplp0**	sp|P19945|RLA0_RAT	34 kDa	8.1232	10.439	7.7642	5.0104	7.6229	7.1467	0.133350707	1.330960566
**RCG55853, isoform CRA_b rCG_55853**	tr|F1LVX3|F1LVX3_RAT	31 kDa	11.169	9.3952	13.587	9.0188	9.5286	7.1467	0.117009666	1.329145602
**Calcium-regulated heat stable protein 1 Carhsp1**	sp|Q9WU49|CHSP1_RAT	16 kDa	3.0462	3.1317	2.9116	1	3.8114	2.0419	0.416357116	1.326295361
**GTP-binding protein SAR1b Sar1b**	sp|Q5HZY2|SAR1B_RAT	22 kDa	3.0462	2.0878	3.8821	2.0042	3.8114	1	0.492572849	1.322862257
**Protein Eps15 Eps15**	tr|E9PSY8|E9PSY8_RAT	87 kDa	4.0616	4.1756	4.8526	2.0042	3.8114	4.0839	0.202143814	1.322268801
**Protein Appl1 (Fragment) Appl1**	tr|D3ZWA8|D3ZWA8_RAT	77 kDa	5.077	4.1756	3.8821	3.0063	2.8586	4.0839	0.114438458	1.320229575
**Actin-related protein 3 Actr3**	sp|Q4V7C7|ARP3_RAT	47 kDa	10.154	10.439	9.7053	10.021	4.7643	8.1677	0.190261725	1.320014813
**Myosin light polypeptide 6 Myl6**	sp|Q64119|MYL6_RAT	17 kDa	12.185	15.659	8.7347	9.0188	9.5286	9.1887	0.215387869	1.318811945
**Glutaryl-Coenzyme A dehydrogenase (Predicted) Gcdh**	tr|D3ZT90|D3ZT90_RAT	50 kDa	6.0924	7.3073	4.8526	4.0083	4.7643	5.1048	0.134587681	1.315253578
**40S ribosomal protein S4, X isoform Rps4x**	sp|P62703|RS4X_RAT	30 kDa	13.2	13.571	13.587	11.023	10.481	9.1887	0.004484617	1.314905499
**Nucleobindin-1 Nucb1**	sp|Q63083|NUCB1_RAT	54 kDa	4.0616	8.3513	5.8232	4.0083	4.7643	5.1048	0.321852117	1.314086212
**Isoform L-type of Pyruvate kinase isozymes R/L Pklr**	sp|P12928-2|KPYR_RAT (+1)	59 kDa	1	1	1.9411	1	1	1	0.373900966	1.3137
**ATPase inhibitor, mitochondrial Atpif1**	sp|Q03344|ATIF1_RAT	12 kDa	1	1	1.9411	1	1	1	0.373900966	1.3137
**Peptidyl-tRNA hydrolase 2 Ptrh2**	tr|Q5XI86|Q5XI86_RAT	20 kDa	1	1	1.9411	1	1	1	0.373900966	1.3137
**Protein Cisd2 Cisd2**	tr|D4AAE9|D4AAE9_RAT	15 kDa	1	1	1.9411	1	1	1	0.373900966	1.3137
**Adenosine kinase Adk**	sp|Q64640|ADK_RAT	40 kDa	1	1	1.9411	1	1	1	0.373900966	1.3137
**Cell adhesion molecule 1 Cadm1**	tr|Q6AYP5|Q6AYP5_RAT	52 kDa	1	1	1.9411	1	1	1	0.373900966	1.3137
**Protein Srsf4 Srsf4**	tr|G3V798|G3V798_RAT	56 kDa	1	1	1.9411	1	1	1	0.373900966	1.3137
**Methyltransferase like 7A Mettl7a**	tr|Q3KRE2|Q3KRE2_RAT	28 kDa	1	1	1.9411	1	1	1	0.373900966	1.3137
**Pcyox1l protein Pcyox1l**	tr|B5DEI0|B5DEI0_RAT	55 kDa	1	1	1.9411	1	1	1	0.373900966	1.3137
**Arginase-1 Arg1**	sp|P07824|ARGI1_RAT	35 kDa	1	1	1.9411	1	1	1	0.373900966	1.3137
**Activated RNA polymerase II transcriptional coactivator p15 Sub1**	sp|Q63396|TCP4_RAT	14 kDa	1	1	1.9411	1	1	1	0.373900966	1.3137
**Protein Arhgdib Arhgdib**	tr|Q5M860|Q5M860_RAT	23 kDa	1	1	1.9411	1	1	1	0.373900966	1.3137
**Matrix Gla protein Mgp**	sp|P08494|MGP_RAT (+1)	12 kDa	1	1	1.9411	1	1	1	0.373900966	1.3137
**Deubiquitinating protein VCIP135 Vcpip1**	sp|Q8CF97|VCIP1_RAT	135 kDa	1	1	1.9411	1	1	1	0.373900966	1.3137
**Cathepsin L1 Ctsl1**	sp|P07154|CATL1_RAT	38 kDa	1	1	1.9411	1	1	1	0.373900966	1.3137
**Inositol-trisphosphate 3-kinase B Itpkb**	sp|P42335|IP3KB_RAT	102 kDa	1	1	1.9411	1	1	1	0.373900966	1.3137
**Prothrombin F2**	sp|P18292|THRB_RAT (+1)	70 kDa	1	1	1.9411	1	1	1	0.373900966	1.3137
**Protein Sec23ip Sec23ip**	tr|G3V8Q8|G3V8Q8_RAT	111 kDa	1	1	1.9411	1	1	1	0.373900966	1.3137
**Coiled-coil domain-containing protein 22 Ccdc22**	sp|P86182|CCD22_RAT	71 kDa	1	1	1.9411	1	1	1	0.373900966	1.3137
**ADP-ribosyl cyclase 1 Cd38**	sp|Q64244|CD38_RAT	34 kDa	1	1	1.9411	1	1	1	0.373900966	1.3137
**N-acetylneuraminate lyase Npl**	sp|Q66H59|NPL_RAT	35 kDa	1	1	1.9411	1	1	1	0.373900966	1.3137
**Protein Dcaf7 Dcaf7**	tr|B2RZ68|B2RZ68_RAT	39 kDa	1	1	1.9411	1	1	1	0.373900966	1.3137
**Complement C1q subcomponent subunit C C1qc**	sp|P31722|C1QC_RAT	26 kDa	1	1	1.9411	1	1	1	0.373900966	1.3137
**Protein LOC100365676 LOC100365676**	tr|D4ABI7|D4ABI7_RAT	43 kDa	1	1	1.9411	1	1	1	0.373900966	1.3137
**Renin receptor Atp6ap2**	sp|Q6AXS4|RENR_RAT	39 kDa	1	1	1.9411	1	1	1	0.373900966	1.3137
**Nicotinate phosphoribosyltransferase Naprt1**	sp|Q6XQN1|PNCB_RAT (+1)	59 kDa	1	1	1.9411	1	1	1	0.373900966	1.3137
**Serine/arginine-rich splicing factor 5 Srsf5**	sp|Q09167|SRSF5_RAT	31 kDa	1	1	1.9411	1	1	1	0.373900966	1.3137
**Sortilin Sort1**	sp|O54861|SORT_RAT	91 kDa	1	1	1.9411	1	1	1	0.373900966	1.3137
**25-hydroxycholesterol 7-alpha-hydroxylase (Fragment) Cyp7b1**	sp|Q63688|CP7B1_RAT (+1)	48 kDa	1	1	1.9411	1	1	1	0.373900966	1.3137
**Sodium- and chloride-dependent GABA transporter 1 Slc6a1**	sp|P23978|SC6A1_RAT	67 kDa	1	1	1.9411	1	1	1	0.373900966	1.3137
**Mitochondrial import receptor subunit TOM40 homolog Tomm40**	sp|Q75Q40|TOM40_RAT (+1)	38 kDa	1	1	1.9411	1	1	1	0.373900966	1.3137
**Interleukin enhancer-binding factor 2 Ilf2**	sp|Q7TP98|ILF2_RAT	51 kDa	1	1	1.9411	1	1	1	0.373900966	1.3137
**Ubiquitin fusion degradation protein 1 homolog Ufd1l**	sp|Q9ES53|UFD1_RAT	34 kDa	1	1	1.9411	1	1	1	0.373900966	1.3137
**Protein Spcs2 Spcs2**	tr|D3ZD11|D3ZD11_RAT	25 kDa	1	1	1.9411	1	1	1	0.373900966	1.3137
**Activity and neurotransmitter-induced early gene protein 4 Dclk1**	tr|Q9WVP7|Q9WVP7_RAT	6 kDa	1	1	1.9411	1	1	1	0.373900966	1.3137
**3'(2'),5'-bisphosphate nucleotidase 1 Bpnt1**	sp|Q9Z1N4|BPNT1_RAT	33 kDa	2.0308	4.1756	2.9116	3.0063	1.9057	2.0419	0.368492704	1.311206661
**Phospholipase A-2-activating protein Plaa**	sp|P54319|PLAP_RAT	87 kDa	2.0308	2.0878	1	1	1.9057	1	0.433527894	1.310546125
**Acylpyruvase FAHD1, mitochondrial Fahd1**	sp|Q6AYQ8|FAHD1_RAT	24 kDa	2.0308	2.0878	1	1	1.9057	1	0.433527894	1.310546125
**Galectin-1 Lgals1**	sp|P11762|LEG1_RAT	15 kDa	18.277	18.79	17.469	14.029	13.34	14.293	0.000852314	1.309010609
**Barrier-to-autointegration factor Banf1**	sp|Q9R1T1|BAF_RAT	10 kDa	3.0462	3.1317	2.9116	3.0063	1.9057	2.0419	0.11340126	1.307108241
**Isoform 2 of Cytosol aminopeptidase Lap3**	sp|Q68FS4-2|AMPL_RAT (+1)	53 kDa	31.477	27.142	32.027	24.05	22.869	22.461	0.011830643	1.306514846
**Glycogenin-1 Gyg1**	sp|O08730|GLYG_RAT (+1)	37 kDa	3.0462	1	3.8821	1	1	4.0839	0.669691041	1.303160801
**Heat shock-related 70 kDa protein 2 Hspa2**	sp|P14659|HSP72_RAT	70 kDa	42.647	37.581	35.909	30.063	28.586	30.629	0.013342085	1.300846793
**Protein Col6a1 Col6a1**	tr|D3ZUL3|D3ZUL3_RAT	109 kDa	24.37	30.273	17.469	18.038	17.151	20.419	0.22388948	1.296791829
**Transcription factor A, mitochondrial Tfam**	sp|Q91ZW1|TFAM_RAT	28 kDa	3.0462	1	1	1	1.9057	1	0.63710951	1.292009115
**Heterogeneous nuclear ribonucleoprotein A3 Hnrnpa3**	sp|Q6URK4|ROA3_RAT	40 kDa	19.293	18.79	19.411	14.029	16.199	14.293	0.003667464	1.29139058
**Transcription elongation factor B polypeptide 1 Tceb1**	sp|P83941|ELOC_RAT	12 kDa	1	2.0878	1.9411	1	1.9057	1	0.457074332	1.287579691
**Protein Serpinb9 Serpinb9**	tr|Q6AYF8|Q6AYF8_RAT	42 kDa	1	2.0878	1.9411	1	1.9057	1	0.457074332	1.287579691
**OTU domain containing 6B (Predicted), isoform CRA_b Otud6b**	tr|D3ZGY2|D3ZGY2_RAT	37 kDa	1	2.0878	1.9411	1	1.9057	1	0.457074332	1.287579691
**Redox-regulatory protein FAM213A Fam213a**	sp|Q6AXX6|F213A_RAT	26 kDa	7.1078	6.2634	5.8232	6.0125	3.8114	5.1048	0.127649793	1.285738209
**Cytochrome c oxidase subunit 2 Mtco2**	sp|P00406|COX2_RAT	26 kDa	5.077	6.2634	7.7642	6.0125	4.7643	4.0839	0.214965185	1.285578741
**Glutathione S-transferase Mu 1 Gstm1**	sp|P04905|GSTM1_RAT	26 kDa	15.231	11.483	16.499	11.023	11.434	11.231	0.103425026	1.282741629
**Isoform 2 of Drebrin-like protein Dbnl**	sp|Q9JHL4-2|DBNL_RAT (+2)	48 kDa	7.1078	9.3952	7.7642	7.0146	4.7643	7.1467	0.158795641	1.282242043
**Neuromodulin Gap43**	sp|P07936|NEUM_RAT	24 kDa	12.185	12.527	14.558	9.0188	11.434	10.21	0.04779704	1.280704958
**Sodium/potassium-transporting ATPase subunit alpha-1 Atp1a1**	sp|P06685|AT1A1_RAT	113 kDa	34.524	25.054	46.585	24.05	33.35	25.524	0.32233719	1.280244561
**Tropomyosin alpha-1 chain Tpm1**	sp|P04692|TPM1_RAT	33 kDa	16.246	24.01	15.528	17.035	14.293	12.252	0.252985832	1.280036714
**40S ribosomal protein S15 Rps15**	sp|P62845|RS15_RAT	17 kDa	3.0462	3.1317	3.8821	4.0083	2.8586	1	0.469419267	1.27877563

**Uncharacterized protein (Fragment)**	tr|D3ZZM1|D3ZZM1_RAT (+1)	58 kDa	2.0308	2.0878	1	2.0042	1	1	0.487957403	1.278307777
**Eukaryotic translation initiation factor 1 Eif1**	tr|B0K008|B0K008_RAT	13 kDa	2.0308	2.0878	1	2.0042	1	1	0.487957403	1.278307777
**Kallikrein 6, isoform CRA_a Klk6**	tr|O54854|O54854_RAT	28 kDa	2.0308	2.0878	1	2.0042	1	1	0.487957403	1.278307777
**Methylmalonate-semialdehyde dehydrogenase [acylating], mitochondrial Aldh6a1**	sp|Q02253|MMSA_RAT (+1)	58 kDa	14.216	15.659	14.558	11.023	10.481	13.273	0.028426086	1.277654772
**Putative 60S ribosomal protein L37a Rpl37a-ps1**	sp|P61515|RL37P_RAT	10 kDa	4.0616	2.0878	3.8821	2.0042	3.8114	2.0419	0.45051377	1.276678333
**Peptidyl-prolyl cis-trans isomerase FKBP1A Fkbp1a**	sp|Q62658|FKB1A_RAT	12 kDa	4.0616	4.1756	1.9411	3.0063	1.9057	3.0629	0.420219051	1.276291866
**ATP-dependent (S)-NAD(P)H-hydrate dehydratase Carkd**	sp|D4AAT7|NNRD_RAT (+1)	37 kDa	3.0462	5.2195	6.7937	5.0104	4.7643	2.0419	0.495714435	1.2744275
**Long-chain-fatty-acid--CoA ligase ACSBG1 Acsbg1**	sp|Q924N5|ACBG1_RAT	81 kDa	9.1386	3.1317	11.646	8.0167	6.67	4.0839	0.570257481	1.274136149
**Protein PRRC1 Prrc1**	sp|Q3T1I4|PRRC1_RAT (+1)	46 kDa	2.0308	1	1.9411	1	1.9057	1	0.471096034	1.272985636
**Protein Copa Copa**	tr|G3V6T1|G3V6T1_RAT	138 kDa	2.0308	1	1.9411	1	1.9057	1	0.471096034	1.272985636
**60S ribosomal protein L36 LOC100361060**	tr|D3ZZ95|D3ZZ95_RAT	12 kDa	2.0308	1	1.9411	1	1.9057	1	0.471096034	1.272985636
**Protein Rabl6 Rabl6**	tr|D3ZKQ4|D3ZKQ4_RAT	80 kDa	1	4.1756	1	1	2.8586	1	0.738486153	1.271065739
**Ribonuclease UK114 Hrsp12**	sp|P52759|UK114_RAT	14 kDa	7.1078	3.1317	4.8526	6.0125	2.8586	3.0629	0.53113602	1.264630468
**Glutathione reductase Gsr**	tr|F1LRE1|F1LRE1_RAT	45 kDa	4.0616	4.1756	2.9116	1	4.7643	3.0629	0.541463762	1.263005256
**3-ketoacyl-CoA thiolase, mitochondrial Acaa2**	tr|G3V9U2|G3V9U2_RAT	42 kDa	15.231	14.615	13.587	11.023	14.293	9.1887	0.130520656	1.258756054
**Ndufa4 protein Ndufa4**	tr|B2RZD6|B2RZD6_RAT	9 kDa	4.0616	4.1756	2.9116	3.0063	3.8114	2.0419	0.306576009	1.258386383
**Quinone oxidoreductase Cryz**	sp|Q6AYT0|QOR_RAT	35 kDa	9.1386	4.1756	3.8821	4.0083	7.6229	2.0419	0.645140603	1.257673827
**Isoform 2 of Large proline-rich protein BAG6 Bag6**	sp|Q6MG49-2|BAG6_RAT	115 kDa	1	1	2.9116	1	1.9057	1	0.659210785	1.257546663
**Histone H2A.J H2afj**	sp|A9UMV8|H2AJ_RAT (+10)	14 kDa	9.1386	10.439	7.7642	8.0167	6.67	7.1467	0.101610516	1.252292359
**Histone H2A type 1**	sp|P02262|H2A1_RAT (+2)	14 kDa	9.1386	10.439	7.7642	8.0167	6.67	7.1467	0.101610516	1.252292359
**Glia maturation factor beta Gmfb**	sp|Q63228|GMFB_RAT	17 kDa	4.0616	3.1317	3.8821	3.0063	3.8114	2.0419	0.275647759	1.250101585
**Isoform 2 of Echinoderm microtubule-associated protein-like 2 Eml2**	sp|Q6P6T4-2|EMAL2_RAT (+1)	69 kDa	7.1078	4.1756	4.8526	4.0083	3.8114	5.1048	0.33319019	1.248481566
**SH3 domain-binding glutamic acid-rich-like protein Sh3bgrl**	tr|B5DFD8|B5DFD8_RAT	13 kDa	4.0616	1	1	1	2.8586	1	0.753837476	1.24760219
**Tumor protein D54 Tpd52l2**	sp|Q6PCT3|TPD54_RAT	24 kDa	5.077	2.0878	3.8821	3.0063	3.8114	2.0419	0.509552591	1.246884735
**Vesicle-associated membrane protein 2 Vamp2**	sp|P63045|VAMP2_RAT	13 kDa	3.0462	3.1317	3.8821	4.0083	1	3.0629	0.514050825	1.246406978
**Coronin-1A Coro1a**	sp|Q91ZN1|COR1A_RAT	51 kDa	7.1078	7.3073	7.7642	8.0167	5.7171	4.0839	0.277334976	1.244790293
**Vacuolar protein sorting-associated protein 28 homolog Vps28**	sp|B5DEN9|VPS28_RAT	26 kDa	1	2.0878	1.9411	1	1	2.0419	0.536021726	1.244192088
**Protein Gna13 (Fragment) Gna13**	tr|F1LNG7|F1LNG7_RAT	36 kDa	1	2.0878	1.9411	1	1	2.0419	0.536021726	1.244192088
**Isoform 2 of Mitochondrial fission 1 protein Fis1**	sp|P84817-2|FIS1_RAT (+2)	20 kDa	5.077	7.3073	4.8526	5.0104	4.7643	4.0839	0.246886104	1.243769212
**Endophilin-B1 Sh3glb1**	sp|Q6AYE2|SHLB1_RAT	41 kDa	6.0924	6.2634	8.7347	6.0125	3.8114	7.1467	0.35004516	1.242766903
**Palmitoyl-protein thioesterase 1 Ppt1**	sp|P45479|PPT1_RAT	34 kDa	9.1386	8.3513	5.8232	5.0104	7.6229	6.1258	0.292773513	1.242762179
**NADH dehydrogenase [ubiquinone] 1 alpha subcomplex subunit 2 Ndufa2**	tr|D3ZS58|D3ZS58_RAT	11 kDa	3.0462	2.0878	1	1	1.9057	2.0419	0.589681812	1.239793031
**sp|K22E_HUMAN|**	zz|ZZ_FGCZCont0146|	66 kDa	21.323	13.571	9.7053	13.027	6.67	16.335	0.555080909	1.237769205
**Ferrochelatase Fech**	tr|D3ZBM3|D3ZBM3_RAT	48 kDa	4.0616	1	1	2.0042	1.9057	1	0.737753106	1.234566895
**Protein Vps26b Vps26b**	tr|B1WBS4|B1WBS4_RAT	39 kDa	2.0308	2.0878	1.9411	2.0042	1.9057	1	0.300303453	1.234179922
**Prostaglandin E synthase 2 (Predicted), isoform CRA_b Ptges2**	tr|D4AE56|D4AE56_RAT	43 kDa	2.0308	2.0878	1.9411	2.0042	1.9057	1	0.300303453	1.234179922
**Heat shock 70 kDa protein 1A/1B Hspa1a**	sp|Q07439|HSP71_RAT (+1)	70 kDa	10.154	9.3952	8.7347	8.0167	5.7171	9.1887	0.179244352	1.233892464
**Proteasome subunit alpha type-2 Psma2**	sp|P17220|PSA2_RAT	26 kDa	11.169	9.3952	8.7347	7.0146	8.5757	8.1677	0.099386302	1.233222493
**KH domain-containing, RNA-binding, signal transduction-associated protein 1 Khdrbs1**	sp|Q91V33|KHDR1_RAT	48 kDa	3.0462	6.2634	2.9116	4.0083	2.8586	3.0629	0.5433532	1.230759935
**Protein Arl8a Arl8a**	tr|D3ZPP2|D3ZPP2_RAT	21 kDa	7.1078	8.3513	7.7642	6.0125	5.7171	7.1467	0.062233845	1.230288775
**Protein phosphatase 1A Ppm1a**	sp|P20650|PPM1A_RAT	42 kDa	2.0308	1	1.9411	1	1	2.0419	0.552663804	1.230089809
**40S ribosomal protein S3a Rps3a**	sp|P49242|RS3A_RAT	30 kDa	10.154	10.439	8.7347	8.0167	6.67	9.1887	0.113142671	1.22836476
**COMM domain-containing protein 3 Commd3**	sp|Q6P9U3|COMD3_RAT	22 kDa	1	1	2.9116	2.0042	1	1	0.695912062	1.226612057
**Histone H1.0 H1f0**	sp|P43278|H10_RAT	21 kDa	7.1078	7.3073	3.8821	6.0125	3.8114	5.1048	0.43009486	1.225639205
**Bcl2-associated athanogene 3 Bag3**	tr|Q5U2U8|Q5U2U8_RAT	61 kDa	14.216	9.3952	7.7642	10.021	10.481	5.1048	0.499171228	1.225276099
**Periaxin Prx**	tr|G3V8D2|G3V8D2_RAT	146 kDa	38.585	44.888	43.674	32.067	39.067	32.671	0.058100631	1.224863928
**Protein Hnrnpl Hnrnpl**	tr|F2Z3R2|F2Z3R2_RAT (+1)	64 kDa	8.1232	14.615	7.7642	11.023	5.7171	8.1677	0.528189936	1.22461237
**Serine/threonine-protein phosphatase PP1-beta catalytic subunit Ppp1cb**	sp|P62142|PP1B_RAT	37 kDa	14.216	12.527	14.558	13.027	9.5286	11.231	0.102928306	1.2224077
**Protein Col6a3 Col6a3**	tr|D4A111|D4A111_RAT	306 kDa	82.248	87.688	81.524	72.15	69.559	64.321	0.007366053	1.220501869
**Cytoplasmic aconitate hydratase Aco1**	tr|G3V6S2|G3V6S2_RAT	98 kDa	6.0924	11.483	7.7642	7.0146	7.6229	6.1258	0.407892839	1.220403308
**Polyadenylate-binding protein 1 Pabpc1**	sp|Q9EPH8|PABP1_RAT	71 kDa	19.293	18.79	17.469	16.033	15.246	14.293	0.010904408	1.218994119
**Bifunctional purine biosynthesis protein PURH Atic**	sp|O35567|PUR9_RAT	64 kDa	22.339	13.571	20.381	15.031	20.01	11.231	0.415134607	1.216524032
**F-actin-capping protein subunit beta Capzb**	sp|Q5XI32|CAPZB_RAT	31 kDa	10.154	10.439	11.646	9.0188	11.434	6.1258	0.303939357	1.212968328
**Nucleoside diphosphate kinase A Nme1**	sp|Q05982|NDKA_RAT	17 kDa	11.169	13.571	18.44	14.029	12.387	9.1887	0.381123676	1.212761237
**WD repeat-containing protein 1 Wdr1**	sp|Q5RKI0|WDR1_RAT	66 kDa	18.277	15.659	16.499	11.023	14.293	16.335	0.165590927	1.210895297
**Protein LOC684558 LOC684558**	tr|F1LY19|F1LY19_RAT	124 kDa	2.0308	1	2.9116	2.0042	1.9057	1	0.618302405	1.210289415
**Ribosome maturation protein SBDS Sbds**	sp|Q5RK30|SBDS_RAT	29 kDa	6.0924	3.1317	3.8821	3.0063	4.7643	3.0629	0.514080855	1.209784465
**Leucine-rich repeat-containing protein 59 Lrrc59**	sp|Q5RJR8|LRC59_RAT	35 kDa	4.0616	2.0878	1	3.0063	1.9057	1	0.718867843	1.209303112
**Ribose-phosphate pyrophosphokinase 2 Prps2**	sp|P09330|PRPS2_RAT	35 kDa	1	7.3073	4.8526	3.0063	3.8114	4.0839	0.706969748	1.207153078
**Protein S100-A1 S100a1**	sp|P35467|S10A1_RAT	11 kDa	4.0616	4.1756	4.8526	4.0083	3.8114	3.0629	0.124427744	1.202819179
**Grancalcin (Predicted) Gca**	tr|D3ZYI0|D3ZYI0_RAT	25 kDa	2.0308	2.0878	1.9411	2.0042	1	2.0419	0.381438786	1.200867997
**Lon protease homolog, mitochondrial Lonp1**	sp|Q924S5|LONM_RAT	106 kDa	6.0924	11.483	13.587	9.0188	5.7171	11.231	0.562660412	1.200081642
**Periaxin Prx**	sp|Q63425|PRAX_RAT	146 kDa	44.678	49.064	48.526	36.075	43.831	38.797	0.041785597	1.198520678
**Claudin 11 Cldn11**	tr|Q6IRG7|Q6IRG7_RAT	22 kDa	3.0462	4.1756	1	3.0063	2.8586	1	0.709795849	1.19765765
**3-hydroxyacyl-CoA dehydrogenase type-2 Hsd17b10**	sp|O70351|HCD2_RAT (+1)	27 kDa	14.216	13.571	12.617	12.025	10.481	11.231	0.026004906	1.19761686
**Antisecretory factor Psmd4**	tr|O88321|O88321_RAT	41 kDa	7.1078	5.2195	6.7937	8.0167	2.8586	5.1048	0.549393435	1.19655071
**Small glutamine-rich tetratricopeptide repeat-containing protein alpha Sgta**	sp|O70593|SGTA_RAT	34 kDa	5.077	3.1317	4.8526	5.0104	2.8586	3.0629	0.483788736	1.194787731
**Isoform 2 of Heterogeneous nuclear ribonucleoprotein D0 Hnrnpd**	sp|Q9JJ54-2|HNRPD_RAT (+2)	36 kDa	9.1386	10.439	8.7347	10.021	8.5757	5.1048	0.376634567	1.194536211
**DnaJ (Hsp40) homolog, subfamily A, member 2 Dnaja2**	tr|Q5M9H7|Q5M9H7_RAT	46 kDa	7.1078	7.3073	6.7937	4.0083	7.6229	6.1258	0.338415482	1.194390944
**Protein Col6a3 Col6a3**	tr|D4A115|D4A115_RAT	240 kDa	80.217	86.644	81.524	72.15	70.511	65.342	0.009017858	1.194141431
**Protein LOC687295 LOC687295**	tr|D3ZJX5|D3ZJX5_RAT	40 kDa	2.0308	3.1317	1.9411	1	1.9057	3.0629	0.621960246	1.190161847
**Adenylyl cyclase-associated protein 1 Cap1**	sp|Q08163|CAP1_RAT	52 kDa	23.354	24.01	22.322	20.042	18.104	20.419	0.013007845	1.189891573
**Arf-GAP domain and FG repeat-containing protein 1 Agfg1**	sp|Q4KLH5|AGFG1_RAT (+1)	58 kDa	3.0462	2.0878	1.9411	2.0042	1.9057	2.0419	0.343600984	1.18873282
**Dynein light chain roadblock-type 1 Dynlrb1**	sp|P62628|DLRB1_RAT	11 kDa	3.0462	2.0878	1.9411	2.0042	1.9057	2.0419	0.343600984	1.18873282
**Membrane-bound carbonic anhydrase 14 Car14**	tr|A2IBE0|A2IBE0_RAT	38 kDa	4.0616	5.2195	4.8526	4.0083	3.8114	4.0839	0.101704926	1.187346685
**Uncharacterized protein**	tr|E9PT29|E9PT29_RAT	47 kDa	8.1232	7.3073	5.8232	7.0146	4.7643	6.1258	0.300182019	1.187045859
**Protein S100a13 S100a13**	tr|D3ZTB5|D3ZTB5_RAT	11 kDa	2.0308	1	2.9116	3.0063	1	1	0.737235977	1.1869844
**Endophilin-A2 Sh3gl1**	sp|O35964|SH3G1_RAT	41 kDa	5.077	5.2195	3.8821	5.0104	2.8586	4.0839	0.38070862	1.186205858
**Tumor susceptibility gene 101 protein Tsg101**	sp|Q6IRE4|TS101_RAT (+1)	44 kDa	3.0462	2.0878	1.9411	1	1.9057	3.0629	0.621423632	1.185386858
**Apoptosis-inducing factor 1, mitochondrial Aifm1**	sp|Q9JM53|AIFM1_RAT	67 kDa	7.1078	11.483	4.8526	5.0104	7.6229	7.1467	0.593055173	1.18520728
**Protein NEWGENE_1309751 Tfg**	tr|Q6AYR1|Q6AYR1_RAT	43 kDa	3.0462	4.1756	1	3.0063	1.9057	2.0419	0.691951902	1.18232934
**Protein LOC100360057 (Fragment) Rpl22**	tr|F7FLF2|F7FLF2_RAT	15 kDa	2.0308	2.0878	2.9116	2.0042	1.9057	2.0419	0.279274781	1.181188884
**Neurolysin, mitochondrial Nln**	sp|P42676|NEUL_RAT	80 kDa	2.0308	2.0878	2.9116	2.0042	1.9057	2.0419	0.279274781	1.181188884
**Protein Vps35 Vps35**	tr|G3V8A5|G3V8A5_RAT	92 kDa	2.0308	2.0878	2.9116	1	1.9057	3.0629	0.620959333	1.177864156
**Tropomyosin alpha-3 chain Tpm3**	sp|Q63610|TPM3_RAT	29 kDa	20.308	21.922	18.44	22.046	16.199	13.273	0.332294957	1.177646648
**F-actin-capping protein subunit alpha-2 Capza2**	sp|Q3T1K5|CAZA2_RAT	33 kDa	11.169	8.3513	9.7053	8.0167	7.6229	9.1887	0.193824959	1.17710838
**Flotillin-1 Flot1**	sp|Q9Z1E1|FLOT1_RAT	47 kDa	9.1386	10.439	9.7053	9.0188	6.67	9.1887	0.17644916	1.17708371
**Annexin Anxa7**	tr|Q6IRJ7|Q6IRJ7_RAT	50 kDa	3.0462	5.2195	1	1	3.8114	3.0629	0.769673551	1.176701421
**Protein Epb4.1l2 Epb4.1l2**	tr|D3ZM69|D3ZM69_RAT	110 kDa	28.431	35.493	27.175	28.058	22.869	26.545	0.206100315	1.175895808
**Eukaryotic translation initiation factor 5A-1 Eif5a**	sp|Q3T1J1|IF5A1_RAT	17 kDa	7.1078	7.3073	7.7642	6.0125	5.7171	7.1467	0.082230525	1.174981326
**Histone H2A.Z H2afz**	sp|P0C0S7|H2AZ_RAT (+1)	14 kDa	8.1232	8.3513	6.7937	7.0146	6.67	6.1258	0.104291717	1.174544684
**Omega-amidase NIT2 Nit2**	sp|Q497B0|NIT2_RAT	31 kDa	6.0924	4.1756	4.8526	3.0063	4.7643	5.1048	0.432755795	1.174379048
**40S ribosomal protein S11 Rps11**	sp|P62282|RS11_RAT	18 kDa	5.077	5.2195	4.8526	5.0104	3.8114	4.0839	0.119221875	1.173830168
**Mitochondrial inner membrane protein (Fragment) Immt**	sp|Q3KR86|IMMT_RAT	67 kDa	23.354	28.185	19.411	23.048	19.057	18.377	0.298921333	1.173076287
**Aflatoxin B1 aldehyde reductase member 2 Akr7a2**	sp|Q8CG45|ARK72_RAT	41 kDa	4.0616	6.2634	4.8526	3.0063	3.8114	6.1258	0.547667248	1.17260401
**Sideroflexin-3 Sfxn3**	sp|Q9JHY2|SFXN3_RAT	35 kDa	4.0616	6.2634	4.8526	3.0063	3.8114	6.1258	0.547667248	1.17260401
**Glutathione S-transferase kappa 1 Gstk1**	sp|P24473|GSTK1_RAT	25 kDa	4.0616	5.2195	5.8232	5.0104	3.8114	4.0839	0.310393823	1.170358834
**Peptidyl-prolyl cis-trans isomerase Fkbp3**	tr|G3V6L9|G3V6L9_RAT	25 kDa	2.0308	3.1317	2.9116	2.0042	2.8586	2.0419	0.422635423	1.169362898
**Heterogeneous nuclear ribonucleoprotein A1 Hnrnpa1**	sp|P04256|ROA1_RAT (+1)	34 kDa	17.262	17.746	18.44	16.033	13.34	16.335	0.063263761	1.169335784
**Isoform RTN1-S of Reticulon-1 Rtn1**	sp|Q64548-2|RTN1_RAT	24 kDa	3.0462	1	2.9116	2.0042	1.9057	2.0419	0.639130468	1.169024497
**Protein LOC312502 (Fragment) LOC312502**	tr|D3ZJ63|D3ZJ63_RAT	70 kDa	2.0308	3.1317	2.9116	4.0083	1.9057	1	0.705506397	1.167789991
**60S ribosomal protein L14 Rpl14**	sp|Q63507|RL14_RAT (+1)	23 kDa	3.0462	3.1317	1.9411	3.0063	1.9057	2.0419	0.494097763	1.167546269
**Protein quaking Qki**	sp|Q91XU1|QKI_RAT	38 kDa	6.0924	7.3073	9.7053	8.0167	6.67	5.1048	0.460582368	1.167420357
**Protein Ugp2 Ugp2**	tr|Q4V8I9|Q4V8I9_RAT	57 kDa	10.154	9.3952	11.646	7.0146	9.5286	10.21	0.276220211	1.166036212
**26S protease regulatory subunit 8 Psmc5**	sp|P62198|PRS8_RAT	46 kDa	10.154	11.483	10.676	10.021	9.5286	8.1677	0.086012082	1.165806193
**Isoform 2 of Choline transporter-like protein 1 Slc44a1**	sp|Q8VII6-2|CTL1_RAT (+3)	73 kDa	2.0308	1	4.8526	1	4.7643	1	0.837343905	1.1654421
**Nucleoside diphosphate kinase B Nme2**	sp|P19804|NDKB_RAT	17 kDa	17.262	17.746	20.381	17.035	16.199	14.293	0.106817331	1.16542176
**Protein Psmc6 LOC100365869**	tr|G3V6W6|G3V6W6_RAT	46 kDa	8.1232	9.3952	6.7937	8.0167	5.7171	7.1467	0.319309083	1.164344724
**Fatty aldehyde dehydrogenase Aldh3a2**	sp|P30839|AL3A2_RAT (+1)	54 kDa	5.077	5.2195	5.8232	5.0104	4.7643	4.0839	0.103910688	1.163155008
**Proteasome (Prosome, macropain) 26S subunit, non-ATPase, 14 Psmd14**	tr|Q4V8E2|Q4V8E2_RAT	35 kDa	3.0462	3.1317	2.9116	3.0063	3.8114	1	0.639681908	1.162682119
**78 kDa glucose-regulated protein Hspa5**	sp|P06761|GRP78_RAT	72 kDa	35.539	35.493	29.116	29.061	28.586	28.587	0.096017317	1.161351671
**Calcineurin B homologous protein 1 Chp1**	sp|P61023|CHP1_RAT	22 kDa	4.0616	3.1317	1.9411	4.0083	2.8586	1	0.713074672	1.161118102
**Septin-7 Sept7**	tr|D4A0F5|D4A0F5_RAT (+1)	51 kDa	34.524	28.185	27.175	23.048	24.774	29.608	0.241737451	1.160842051
**Methyl-CpG-binding protein 2 Mecp2**	sp|Q00566|MECP2_RAT	53 kDa	4.0616	7.3073	4.8526	5.0104	2.8586	6.1258	0.616490973	1.159109098
**Eukaryotic translation initiation factor 4E Eif4e**	sp|P63074|IF4E_RAT	25 kDa	2.0308	3.1317	2.9116	2.0042	1.9057	3.0629	0.503805679	1.15794229
**Sodium/potassium-transporting ATPase subunit beta-3 Atp1b3**	sp|Q63377|AT1B3_RAT	32 kDa	2.0308	3.1317	2.9116	2.0042	1.9057	3.0629	0.503805679	1.15794229
**Ribosomal protein S5, isoform CRA_b Rps5**	tr|B0BN81|B0BN81_RAT	23 kDa	4.0616	6.2634	6.7937	6.0125	5.7171	3.0629	0.57060453	1.157255366
**Transforming protein RhoA Rhoa**	sp|P61589|RHOA_RAT	22 kDa	13.2	12.527	9.7053	11.023	11.434	8.1677	0.340842078	1.156984395
**EH domain-containing protein 1 Ehd1**	sp|Q641Z6|EHD1_RAT	61 kDa	10.154	9.3952	7.7642	7.0146	10.481	6.1258	0.459173423	1.156298949
**Fascin Fscn1**	sp|P85845|FSCN1_RAT	54 kDa	21.323	20.878	24.263	22.046	15.246	20.419	0.274956825	1.151669526
**Peptidyl-prolyl cis-trans isomerase B Ppib**	sp|P24368|PPIB_RAT	24 kDa	10.154	12.527	11.646	11.023	8.5757	10.21	0.206041965	1.151576553
**Isoform 2 of Tropomyosin alpha-3 chain Tpm3**	sp|Q63610-2|TPM3_RAT	29 kDa	20.308	20.878	20.381	22.046	17.151	14.293	0.30138209	1.151000187

**Protein Rhoc Rhoc**	tr|B2RYP0|B2RYP0_RAT	22 kDa	1	8.3513	1	7.0146	1	1	0.894885017	1.148281676
**60S ribosomal protein L7a Rpl7a**	sp|P62425|RL7A_RAT (+1)	30 kDa	11.169	12.527	12.617	12.025	11.434	8.1677	0.291822152	1.148175434
**Histone H2A Hist2h2ab**	tr|D3ZWE0|D3ZWE0_RAT	14 kDa	3.0462	4.1756	2.9116	1	4.7643	3.0629	0.726410542	1.147974443
**Rab GDP dissociation inhibitor beta Gdi2**	sp|P50399|GDIB_RAT	51 kDa	31.477	31.317	28.145	25.052	26.68	27.566	0.041577371	1.146800676
**Methylmalonyl CoA epimerase (Predicted), isoform CRA_d Mcee**	tr|D4A197|D4A197_RAT	19 kDa	4.0616	1	2.9116	3.0063	1.9057	2.0419	0.740678664	1.146579617
**Protein LOC100911677 LOC100911677**	tr|M0R9Q1|M0R9Q1_RAT (+1)	69 kDa	3.0462	4.1756	1.9411	2.0042	1.9057	4.0839	0.705403024	1.146250844
**Malic enzyme Me2**	tr|D3ZJH9|D3ZJH9_RAT	65 kDa	6.0924	7.3073	4.8526	7.0146	3.8114	5.1048	0.544351338	1.145724006
**Eukaryotic translation initiation factor 4A1 Eif4a1**	tr|Q6P3V8|Q6P3V8_RAT	46 kDa	7.1078	11.483	8.7347	9.0188	6.67	8.1677	0.469737008	1.145411104
**Protein LOC100364427 LOC100364427**	tr|M0R9I8|M0R9I8_RAT (+1)	15 kDa	4.0616	7.3073	6.7937	7.0146	4.7643	4.0839	0.598192179	1.14498071
**Protein Epb4.1l2 Epb4.1l2**	tr|D3ZAY7|D3ZAY7_RAT	88 kDa	29.447	34.449	29.116	29.061	24.774	27.566	0.143949554	1.142639525
**Coiled-coil-helix-coiled-coil-helix domain containing 3 (Predicted), isoform CRA_a Chchd3**	tr|D3ZUX5|D3ZUX5_RAT	26 kDa	7.1078	7.3073	5.8232	6.0125	7.6229	4.0839	0.496466004	1.142161372
**60S ribosomal protein L4 Rpl4**	tr|Q6P3V9|Q6P3V9_RAT	47 kDa	12.185	12.527	12.617	12.025	10.481	10.21	0.057018684	1.141001345
**Uncharacterized protein**	tr|E9PTD2|E9PTD2_RAT	65 kDa	3.0462	3.1317	2.9116	3.0063	1.9057	3.0629	0.385897519	1.139763508
**Ubiquitin-40S ribosomal protein S27a Rps27a**	sp|P62982|RS27A_RAT	18 kDa	9.1386	8.3513	10.676	8.0167	8.5757	8.1677	0.181513294	1.137551948
**Stomatin-like protein 2, mitochondrial Stoml2**	sp|Q4FZT0|STML2_RAT	38 kDa	11.169	10.439	8.7347	10.021	9.5286	7.1467	0.34776206	1.136588216
**Protein FAM98A Fam98a**	sp|Q5FWT1|FA98A_RAT	55 kDa	4.0616	2.0878	2.9116	3.0063	1.9057	3.0629	0.625198188	1.136189795
**Isoform 2 of Protein transport protein Sec31A Sec31a**	sp|Q9Z2Q1-2|SC31A_RAT (+2)	117 kDa	2.0308	3.1317	3.8821	3.0063	1.9057	3.0629	0.615848017	1.134133343
**Protein LOC100362751 LOC498555**	tr|D4A4D5|D4A4D5_RAT	12 kDa	7.1078	7.3073	5.8232	6.0125	5.7171	6.1258	0.173816	1.133455425
**3-mercaptopyruvate sulfurtransferase Mpst**	sp|P97532|THTM_RAT	33 kDa	3.0462	3.1317	3.8821	2.0042	3.8114	3.0629	0.539452503	1.133074281
**Protein kinase C alpha type Prkca**	tr|F1LS98|F1LS98_RAT	77 kDa	4.0616	3.1317	2.9116	3.0063	2.8586	3.0629	0.334218235	1.131846592
**60S ribosomal protein L23 Rpl23**	sp|P62832|RL23_RAT	15 kDa	3.0462	6.2634	1.9411	3.0063	2.8586	4.0839	0.764418877	1.130860003
**3-ketoacyl-CoA thiolase, mitochondrial Acaa2**	sp|P13437|THIM_RAT	42 kDa	14.216	15.659	13.587	12.025	15.246	11.231	0.294683164	1.128824477
**Protein Sept6 Sept6**	tr|B5DFG5|B5DFG5_RAT	49 kDa	16.246	13.571	11.646	12.025	11.434	13.273	0.334886691	1.128797779
**Mammalian ependymin-related protein 1 Epdr1**	sp|Q5XII0|EPDR1_RAT	26 kDa	7.1078	6.2634	6.7937	5.0104	5.7171	7.1467	0.321155831	1.128156785
**cAMP-dependent protein kinase type I-beta regulatory subunit Prkar1b**	sp|P81377|KAP1_RAT (+1)	43 kDa	5.077	1	2.9116	3.0063	1.9057	3.0629	0.798166571	1.127111312
**Sodium/potassium-transporting ATPase subunit alpha-2 Atp1a2**	sp|P06686|AT1A2_RAT	112 kDa	31.477	24.01	45.615	23.048	38.114	28.587	0.649379524	1.126497231
**Coactosin-like protein Cotl1**	sp|B0BNA5|COTL1_RAT	16 kDa	7.1078	7.3073	6.7937	8.0167	5.7171	5.1048	0.429072965	1.125816143
**BWK4 Erp44**	tr|Q5VLR5|Q5VLR5_RAT	47 kDa	4.0616	5.2195	2.9116	3.0063	4.7643	3.0629	0.634260636	1.125462685
**Acetyl-Coenzyme A dehydrogenase, short chain, isoform CRA_a Acads**	tr|Q6IMX3|Q6IMX3_RAT	45 kDa	8.1232	4.1756	7.7642	7.0146	5.7171	5.1048	0.619324612	1.124828302
**Protein Eea1 (Fragment) Eea1**	tr|F1LUA1|F1LUA1_RAT	161 kDa	8.1232	7.3073	7.7642	5.0104	9.5286	6.1258	0.573956766	1.122425574
**Cysteine and glycine-rich protein 1 Csrp1**	sp|P47875|CSRP1_RAT	21 kDa	14.216	14.615	14.558	12.025	12.387	14.293	0.094168586	1.121017956
**Nuclear transport factor 2 Nutf2**	sp|P61972|NTF2_RAT	14 kDa	4.0616	3.1317	3.8821	3.0063	3.8114	3.0629	0.359651881	1.120923831
**N(G),N(G)-dimethylarginine dimethylaminohydrolase 2 Ddah2**	sp|Q6MG60|DDAH2_RAT	30 kDa	9.1386	7.3073	6.7937	6.0125	7.6229	7.1467	0.393315003	1.118250802
**Protein Myef2 Myef2**	tr|D4AEI5|D4AEI5_RAT	63 kDa	3.0462	4.1756	3.8821	4.0083	2.8586	3.0629	0.469002171	1.118240045
**Synaptic vesicle membrane protein VAT-1 homolog Vat1**	sp|Q3MIE4|VAT1_RAT	43 kDa	18.277	15.659	13.587	11.023	16.199	15.314	0.472245837	1.117241866
**Matrin-3 Matr3**	sp|P43244|MATR3_RAT	94 kDa	7.1078	10.439	7.7642	8.0167	8.5757	6.1258	0.530419877	1.11412876
**Acetyl-CoA acetyltransferase, mitochondrial Acat1**	sp|P17764|THIL_RAT	45 kDa	13.2	17.746	9.7053	11.023	14.293	11.231	0.620917084	1.112301967
**Disks large homolog 1 Dlg1**	tr|F1LNM0|F1LNM0_RAT	101 kDa	5.077	3.1317	3.8821	4.0083	3.8114	3.0629	0.560645859	1.111021263
**Myosin regulatory light chain 12B Myl12b**	sp|P18666|ML12B_RAT	20 kDa	9.1386	8.3513	7.7642	7.0146	8.5757	7.1467	0.259435766	1.110705018
**Protein Fmnl2 Fmnl2**	tr|M0R9R1|M0R9R1_RAT	118 kDa	11.169	7.3073	7.7642	7.0146	9.5286	7.1467	0.593235797	1.107666136
**Osteoclast-stimulating factor 1 Ostf1**	sp|Q6P686|OSTF1_RAT	24 kDa	3.0462	5.2195	3.8821	4.0083	1.9057	5.1048	0.756254279	1.102461248
**Isoform 2 of Gelsolin Gsn**	sp|Q68FP1-2|GELS_RAT	81 kDa	28.431	27.142	25.234	21.044	25.727	26.545	0.269392149	1.10217415
**Aspartyl aminopeptidase Dnpep**	tr|Q4V8H5|Q4V8H5_RAT	53 kDa	11.169	6.2634	8.7347	7.0146	8.5757	8.1677	0.618812691	1.101401633
**Hydroxysteroid dehydrogenase-like protein 2 Hsdl2**	sp|Q4V8F9|HSDL2_RAT	58 kDa	4.0616	5.2195	3.8821	5.0104	2.8586	4.0839	0.619615938	1.101255762
**Poly(RC) binding protein 2 Pcbp2**	tr|Q6AYU5|Q6AYU5_RAT	39 kDa	6.0924	10.439	8.7347	10.021	4.7643	8.1677	0.718428724	1.100775498
**Proteasome subunit alpha type-4 Psma4**	sp|P21670|PSA4_RAT	29 kDa	4.0616	5.2195	4.8526	4.0083	4.7643	4.0839	0.36577144	1.099342745
**Actin related protein 2/3 complex, subunit 4 (Predicted), isoform CRA_a Arpc4**	tr|B2RZ72|B2RZ72_RAT	20 kDa	4.0616	4.1756	4.8526	5.0104	2.8586	4.0839	0.602018661	1.095114993
**Isoform 2 of Mitogen-activated protein kinase 3 Mapk3**	sp|P21708-2|MK03_RAT (+1)	46 kDa	13.2	14.615	14.558	12.025	12.387	14.293	0.220056814	1.094768118
**Protein Dnaja3 Dnaja3**	tr|G3V6I5|G3V6I5_RAT	52 kDa	4.0616	6.2634	4.8526	4.0083	4.7643	5.1048	0.58010475	1.093691902
**RNA-binding motif protein, X chromosome retrogene-like Rbmxrtl**	sp|P84586|RMXRL_RAT	42 kDa	5.077	9.3952	2.9116	5.0104	4.7643	6.1258	0.812453157	1.093286375
**F-actin-capping protein subunit alpha-1 Capza1**	sp|B2GUZ5|CAZA1_RAT	33 kDa	10.154	10.439	10.676	8.0167	11.434	9.1887	0.436055256	1.091817566
**Enoyl-CoA hydratase, mitochondrial Echs1**	sp|P14604|ECHM_RAT	32 kDa	11.169	10.439	9.7053	8.0167	10.481	10.21	0.383062328	1.090763105
**Twinfilin-1 Twf1**	sp|Q5RJR2|TWF1_RAT	40 kDa	6.0924	3.1317	4.8526	4.0083	3.8114	5.1048	0.706124891	1.089148516
**ATP synthase subunit b, mitochondrial Atp5f1**	sp|P19511|AT5F1_RAT	29 kDa	9.1386	9.3952	10.676	10.021	7.6229	9.1887	0.403333002	1.088593726
**EH domain-containing protein 3 Ehd3**	sp|Q8R491|EHD3_RAT	61 kDa	5.077	5.2195	5.8232	5.0104	5.7171	4.0839	0.453098316	1.08833061
**Protein Ranbp1 RGD1565297**	tr|D4A2G9|D4A2G9_RAT	24 kDa	4.0616	7.3073	5.8232	5.0104	5.7171	5.1048	0.66267294	1.085887711
**Glutathione S-transferase alpha-3 Gsta3**	sp|P04904|GSTA3_RAT	25 kDa	11.169	10.439	11.646	9.0188	11.434	10.21	0.330565933	1.084506307
**Adenosylhomocysteinase Ahcyl2**	tr|D3ZWL6|D3ZWL6_RAT	66 kDa	20.308	14.615	13.587	16.033	12.387	16.335	0.635677069	1.08390124
**Peripherin Prph**	sp|P21807|PERI_RAT	54 kDa	42.647	38.625	38.821	37.077	38.114	35.734	0.107757968	1.082650439
**Vesicle-associated membrane protein-associated protein B Vapb**	sp|Q9Z269|VAPB_RAT	27 kDa	6.0924	6.2634	5.8232	4.0083	6.67	6.1258	0.606851896	1.081819318
**Isoform 3 of Dynamin-2 Dnm2**	sp|P39052-3|DYN2_RAT (+1)	98 kDa	9.1386	14.615	13.587	12.025	13.34	9.1887	0.678202791	1.08065417
**Coronin Coro1c**	tr|G3V624|G3V624_RAT	53 kDa	7.1078	6.2634	5.8232	5.0104	6.67	6.1258	0.494948181	1.077961609
**Nucleosome assembly protein 1-like 1 Nap1l1**	tr|G3V6H9|G3V6H9_RAT	45 kDa	7.1078	6.2634	5.8232	7.0146	4.7643	6.1258	0.599602978	1.072031366
**Long-chain specific acyl-CoA dehydrogenase, mitochondrial Acadl**	sp|P15650|ACADL_RAT	48 kDa	12.185	11.483	14.558	11.023	12.387	12.252	0.451915124	1.071897258
**Biliverdin reductase B (Flavin reductase (NADPH)) Blvrb**	tr|B5DF65|B5DF65_RAT	22 kDa	7.1078	5.2195	7.7642	5.0104	7.6229	6.1258	0.700561134	1.071026862
**Myristoylated alanine-rich C-kinase substrate Marcks**	tr|F1LMW7|F1LMW7_RAT (+1)	30 kDa	10.154	9.3952	6.7937	7.0146	10.481	7.1467	0.728727512	1.069011415
**Ras-related protein Rap-1b Rap1b**	sp|Q62636|RAP1B_RAT	21 kDa	15.231	16.703	16.499	16.033	18.104	11.231	0.650098136	1.067558632
**Actin-related protein 2/3 complex subunit 2 Arpc2**	sp|P85970|ARPC2_RAT	34 kDa	8.1232	5.2195	8.7347	4.0083	9.5286	7.1467	0.821746368	1.067386722
**Protein Psat1 Psat1**	tr|E9PSV5|E9PSV5_RAT	40 kDa	12.185	11.483	15.528	11.023	11.434	14.293	0.640615763	1.066557823
**Peroxiredoxin-1 Prdx1**	sp|Q63716|PRDX1_RAT	22 kDa	23.354	24.01	22.322	23.048	20.963	21.44	0.15219685	1.064704894
**Procollagen, type VI, alpha 2, isoform CRA_a Col6a2**	tr|F1LNH3|F1LNH3_RAT	110 kDa	14.216	17.746	18.44	16.033	18.104	13.273	0.630055967	1.063109049
**Actin, alpha skeletal muscle Acta1**	sp|P68136|ACTS_RAT	42 kDa	51.786	59.503	51.438	51.106	52.407	50.027	0.323310778	1.059834571
**26S protease regulatory subunit 6B Psmc4**	sp|Q63570|PRS6B_RAT	47 kDa	9.1386	9.3952	9.7053	7.0146	10.481	9.1887	0.639577411	1.058266471
**Protein Pkp4 Pkp4**	tr|F1M2K6|F1M2K6_RAT (+1)	131 kDa	11.169	9.3952	8.7347	7.0146	10.481	10.21	0.709921701	1.057508229
**Prohibitin-2 Phb2**	sp|Q5XIH7|PHB2_RAT	33 kDa	16.246	24.01	18.44	19.04	17.151	19.398	0.689961364	1.055892353
**Myelin proteolipid protein Plp1**	sp|P60203|MYPR_RAT	30 kDa	20.308	20.878	22.322	16.033	24.774	19.398	0.695378448	1.054862553
**Actin, aortic smooth muscle Acta2**	sp|P62738|ACTA_RAT	42 kDa	47.724	52.195	51.438	47.098	48.596	47.985	0.151966061	1.053438568
**Fumarylacetoacetase Fah**	sp|P25093|FAAA_RAT	46 kDa	8.1232	8.3513	10.676	7.0146	8.5757	10.21	0.733239428	1.052332725
**Guanine nucleotide-binding protein subunit gamma Gng12**	tr|G3V6P8|G3V6P8_RAT	8 kDa	2.0308	3.1317	1	2.0042	2.8586	1	0.908564535	1.051118919
**Adenosylhomocysteinase Ahcy**	sp|P10760|SAHH_RAT	48 kDa	12.185	12.527	9.7053	12.025	9.5286	11.231	0.661894975	1.049800821
**14-3-3 protein epsilon Ywhae**	sp|P62260|1433E_RAT	29 kDa	32.493	29.229	32.998	29.061	27.633	33.692	0.543243365	1.047949904
**Pre-B-cell leukemia transcription factor-interacting protein 1 Pbxip1**	sp|A2VD12|PBIP1_RAT	80 kDa	1	2.0878	1	1	1.9057	1	0.903844968	1.046624165
**Protein Ubfd1 Ubfd1**	tr|G3V8E4|G3V8E4_RAT	34 kDa	1	2.0878	1	1	1.9057	1	0.903844968	1.046624165
**Eukaryotic translation initiation factor 1B Eif1b**	tr|B5DFN1|B5DFN1_RAT	13 kDa	2.0308	2.0878	1	2.0042	1.9057	1	0.890994835	1.042505957
**Proteasome (Prosome, macropain) 26S subunit, non-ATPase, 12 Psmd12**	tr|Q5XIC6|Q5XIC6_RAT	53 kDa	2.0308	2.0878	1	2.0042	1.9057	1	0.890994835	1.042505957
**NADH dehydrogenase (Ubiquinone) 1 beta subcomplex, 11 (Predicted) Ndufb11**	tr|D4A7L4|D4A7L4_RAT	18 kDa	2.0308	2.0878	1	2.0042	1.9057	1	0.890994835	1.042505957
**NADH dehydrogenase (Ubiquinone) 1 beta subcomplex, 7 (Predicted) Ndufb7**	tr|D3ZLT1|D3ZLT1_RAT	17 kDa	3.0462	2.0878	1.9411	2.0042	3.8114	1	0.927456965	1.038074418
**Enolase**	tr|F1LTP6|F1LTP6_RAT	41 kDa	26.4	1	23.293	24.05	23.821	1	0.958861174	1.037281824
**Fatty acid-binding protein, epidermal Fabp5**	sp|P55053|FABP5_RAT	15 kDa	16.246	14.615	17.469	16.033	15.246	15.314	0.539546195	1.037280278
**Trifunctional enzyme subunit alpha, mitochondrial Hadha**	sp|Q64428|ECHA_RAT	83 kDa	30.462	34.449	27.175	29.061	31.444	28.587	0.684353381	1.033605711
**Fermitin family homolog 2 (Drosophila) Fermt2**	tr|Q5XI19|Q5XI19_RAT	78 kDa	2.0308	6.2634	3.8821	3.0063	5.7171	3.0629	0.935815755	1.033089265
**SPRY domain-containing protein 4 Spryd4**	sp|Q4FZT8|SPRY4_RAT	23 kDa	2.0308	1	1	1	1.9057	1	0.931740881	1.03203011
**Protein Thoc4 Thoc4**	tr|D3ZXH7|D3ZXH7_RAT	20 kDa	2.0308	1	1	1	1.9057	1	0.931740881	1.03203011
**Serine/threonine-protein kinase WNK1 Wnk1**	tr|D3ZX61|D3ZX61_RAT (+3)	279 kDa	2.0308	1	1	1	1.9057	1	0.931740881	1.03203011
**NADPH--cytochrome P450 reductase Por**	sp|P00388|NCPR_RAT	77 kDa	3.0462	4.1756	3.8821	3.0063	5.7171	2.0419	0.926601597	1.031452909
**Alpha-enolase Eno1**	sp|P04764|ENOA_RAT (+1)	47 kDa	58.893	53.239	60.173	56.117	60.03	51.048	0.638988365	1.030563115
**ADP-ribosylation factor 4 Arf4**	sp|P61751|ARF4_RAT	20 kDa	1	2.0878	4.8526	1	5.7171	1	0.971323273	1.02893574
**Protein phosphatase 1 regulatory subunit 14A Ppp1r14a**	sp|Q99MC0|PP14A_RAT	17 kDa	4.0616	3.1317	1.9411	2.0042	3.8114	3.0629	0.920920137	1.028822436
**Spectrin alpha chain, non-erythrocytic 1 Sptan1**	sp|P16086|SPTN1_RAT	285 kDa	205.11	191.03	183.43	191.4	178.18	195	0.573126895	1.02655071
**Isoform 2 of Plasminogen activator inhibitor 1 RNA-binding protein Serbp1**	sp|Q6AXS5-2|PAIRB_RAT (+1)	43 kDa	3.0462	3.1317	2.9116	3.0063	3.8114	2.0419	0.889015865	1.025949253
**GTPase activating protein (SH3 domain) binding protein 2 G3bp2**	tr|Q6AY21|Q6AY21_RAT	51 kDa	2.0308	2.0878	1.9411	3.0063	1.9057	1	0.936611216	1.024983085
**S-adenosylmethionine synthase isoform type-2 Mat2a**	sp|P18298|METK2_RAT (+1)	44 kDa	3.0462	4.1756	3.8821	3.0063	4.7643	3.0629	0.899289584	1.024959616
**60S ribosomal protein L29 Rpl29**	sp|P25886|RL29_RAT (+2)	17 kDa	1	2.0878	1.9411	2.0042	1.9057	1	0.936417788	1.024236746
**Transcriptional activator protein Pur-beta Purb**	sp|Q68A21|PURB_RAT	33 kDa	4.0616	3.1317	2.9116	6.0125	2.8586	1	0.961180592	1.023685304
**Toll-interacting protein Tollip**	sp|A2RUW1|TOLIP_RAT	30 kDa	4.0616	5.2195	4.8526	4.0083	5.7171	4.0839	0.876657061	1.023491415
**Proteasome subunit beta type Psmb5**	tr|G3V7Q6|G3V7Q6_RAT	29 kDa	3.0462	6.2634	6.7937	5.0104	6.67	4.0839	0.939284815	1.021504285
**sp|TRYP_PIG|**	zz|ZZ_FGCZCont0179|	24 kDa	7.1078	6.2634	6.7937	7.0146	7.6229	5.1048	0.868394456	1.021405814
**Acyl-CoA thioesterase 9 Acot9**	tr|Q5U2X8|Q5U2X8_RAT	50 kDa	2.0308	3.1317	3.8821	3.0063	3.8114	2.0419	0.93776308	1.020881304
**Eukaryotic translation initiation factor 3 subunit D Eif3d**	sp|Q6AYK8|EIF3D_RAT	64 kDa	1	2.0878	1	2.0042	1	1	0.957676069	1.020878078
**Nuclear protein localization protein 4 homolog Nploc4**	sp|Q9ES54|NPL4_RAT (+1)	68 kDa	1	2.0878	1	2.0042	1	1	0.957676069	1.020878078
**Isoform 2 of A-kinase anchor protein 12 Akap12**	sp|Q5QD51-2|AKA12_RAT (+2)	173 kDa	18.277	18.79	14.558	18.038	16.199	16.335	0.8216847	1.020821799
**Serine/arginine-rich splicing factor 2 Srsf2**	sp|Q6PDU1|SRSF2_RAT	25 kDa	1	3.1317	1.9411	2.0042	1.9057	2.0419	0.951103631	1.020329984
**40S ribosomal protein SA Rpsa**	sp|P38983|RSSA_RAT	33 kDa	6.0924	7.3073	6.7937	8.0167	6.67	5.1048	0.890332706	1.020306697
**C-terminal-binding protein 1 Ctbp1**	sp|Q9Z2F5|CTBP1_RAT (+1)	47 kDa	5.077	4.1756	3.8821	3.0063	4.7643	5.1048	0.912996483	1.02013918
**Isoform Non-brain of Clathrin light chain B Cltb**	sp|P08082-2|CLCB_RAT	23 kDa	6.0924	5.2195	4.8526	4.0083	5.7171	6.1258	0.89536698	1.019765065
**Electron transfer flavoprotein subunit alpha, mitochondrial Etfa**	sp|P13803|ETFA_RAT	35 kDa	12.185	11.483	11.646	11.023	12.387	11.231	0.660910187	1.019427846
**Isoform 2 of Tyrosine-protein phosphatase non-receptor type 11 Ptpn11**	sp|P41499-2|PTN11_RAT (+1)	68 kDa	8.1232	8.3513	7.7642	6.0125	8.5757	9.1887	0.88369898	1.019422212
**Cytochrome b5 type B Cyb5b**	sp|P04166|CYB5B_RAT	16 kDa	2.0308	3.1317	1	3.0063	1	2.0419	0.966207055	1.018898185
**60S ribosomal protein L35 Rpl35**	sp|P17078|RL35_RAT	15 kDa	2.0308	3.1317	2.9116	2.0042	2.8586	3.0629	0.920774183	1.018723898
**4-trimethylaminobutyraldehyde dehydrogenase Aldh9a1**	sp|Q9JLJ3|AL9A1_RAT	54 kDa	11.169	11.483	8.7347	12.025	8.5757	10.21	0.891519534	1.018694804
**Serine hydroxymethyltransferase Shmt2**	tr|Q5U3Z7|Q5U3Z7_RAT	56 kDa	4.0616	3.1317	5.8232	7.0146	4.7643	1	0.969135253	1.01859315

**iRT_Protein_with_AAAAK_spacers concatenated Biognosys Retention Time Kit peptides with AAAAK spacers**	zz|ZZ_FGCZCont0260|	18 kDa	9.1386	12.527	10.676	12.025	9.5286	10.21	0.883179398	1.01819693
**Isoform 2 of Reticulon-3 Rtn3**	sp|Q6RJR6-2|RTN3_RAT	25 kDa	4.0616	4.1756	4.8526	4.0083	4.7643	4.0839	0.832507024	1.018146463
**ArsA arsenite transporter, ATP-binding, homolog 1 (Bacterial) Asna1**	tr|G3V9T7|G3V9T7_RAT	39 kDa	3.0462	3.1317	2.9116	3.0063	2.8586	3.0629	0.574935293	1.018111965
**Protein Limch1 Limch1**	tr|F1M392|F1M392_RAT	122 kDa	4.0616	3.1317	2.9116	4.0083	2.8586	3.0629	0.912629944	1.017633789
**Voltage-dependent anion-selective channel protein 2 Vdac2**	sp|P81155|VDAC2_RAT	32 kDa	16.246	15.659	17.469	17.035	16.199	15.314	0.724774044	1.017014089
**Protein Ddx19a Ddx19b**	tr|F7DP80|F7DP80_RAT	54 kDa	1	3.1317	1	2.0042	1	2.0419	0.972858175	1.016963596
**Glucose-6-phosphate 1-dehydrogenase G6pdx**	sp|P05370|G6PD_RAT	59 kDa	19.293	18.79	18.44	18.038	17.151	20.419	0.776997076	1.016454467
**Bifunctional protein NCOAT Mgea5**	sp|Q8VIJ5|NCOAT_RAT	103 kDa	1	2.0878	1.9411	1	1.9057	2.0419	0.956993725	1.01643221
**Hemoglobin subunit alpha-1/2 Hba1**	sp|P01946|HBA_RAT	15 kDa	11.169	6.2634	6.7937	9.0188	6.67	8.1677	0.945682332	1.015492633
**S-methyl-5'-thioadenosine phosphorylase Mtap**	tr|Q7TP15|Q7TP15_RAT	43 kDa	2.0308	2.0878	1.9411	1	1.9057	3.0629	0.961967329	1.015263211
**Interferon-inducible double stranded RNA-dependent protein kinase activator A Prkra**	sp|Q4V8C7|PRKRA_RAT (+1)	34 kDa	3.0462	2.0878	1.9411	2.0042	1.9057	3.0629	0.949625834	1.014671294
**Destrin Dstn**	sp|Q7M0E3|DEST_RAT	19 kDa	13.2	10.439	14.558	14.029	12.387	11.231	0.906039898	1.014609398
**GTP-binding protein Rheb Rheb**	sp|Q62639|RHEB_RAT	20 kDa	2.0308	2.0878	1	2.0042	1	2.0419	0.963125557	1.014367531
**CDGSH iron-sulfur domain-containing protein 1 Cisd1**	sp|B0K020|CISD1_RAT	12 kDa	7.1078	7.3073	8.7347	6.0125	7.6229	9.1887	0.922648748	1.014270004
**Alcohol dehydrogenase [NADP(+)] Akr1a1**	sp|P51635|AK1A1_RAT	37 kDa	9.1386	10.439	8.7347	10.021	6.67	11.231	0.933179155	1.013978225
**NADH dehydrogenase (Ubiquinone) 1 beta subcomplex, 5 (Predicted), isoform CRA_b Ndufb5**	tr|D4A565|D4A565_RAT	22 kDa	3.0462	4.1756	4.8526	3.0063	3.8114	5.1048	0.952980039	1.012740616
**Protein Ccdc6 (Fragment) Ccdc6**	tr|D4AEK9|D4AEK9_RAT	43 kDa	2.0308	1	1.9411	2.0042	1.9057	1	0.96625594	1.012627548
**Peroxisomal membrane protein PEX14 Pex14**	sp|Q642G4|PEX14_RAT	41 kDa	2.0308	1	1.9411	2.0042	1.9057	1	0.96625594	1.012627548
**Glutathione S-transferase omega-1 Gsto1**	sp|Q9Z339|GSTO1_RAT (+1)	28 kDa	6.0924	4.1756	6.7937	5.0104	5.7171	6.1258	0.938616547	1.012365531
**gi|1346343|sp|P04264| K2C1_HUMAN KERATIN, TYPE II CYTOSKELETAL 1 (CYTO**	zz|ZZ_FGCZCont0025| (+1)	66 kDa	26.4	22.966	25.234	39.081	15.246	19.398	0.970529058	1.01186843
**Protein LOC684270 LOC684270**	tr|M0RAK2|M0RAK2_RAT	22 kDa	1	2.0878	1	1	1	2.0419	0.977150028	1.011356045
**Heat shock protein HSP 90-beta Hsp90ab1**	sp|P34058|HS90B_RAT	83 kDa	41.631	45.932	46.585	43.09	43.831	45.943	0.821166461	1.009664017
**DNA damage-binding protein 1 Ddb1**	tr|G3V8T4|G3V8T4_RAT	127 kDa	1	1	1.9411	1	1.9057	1	0.979675801	1.009063676
**SUMO-activating enzyme subunit 1 Sae1**	sp|Q6AXQ0|SAE1_RAT	39 kDa	1	1	1.9411	1	1.9057	1	0.979675801	1.009063676
**Protein Aspscr1 Aspscr1**	tr|F1LR71|F1LR71_RAT	51 kDa	1	1	1.9411	1	1.9057	1	0.979675801	1.009063676
**Vacuolar-sorting protein SNF8 Snf8**	sp|Q5RK19|SNF8_RAT	29 kDa	1	1	1.9411	1	1.9057	1	0.979675801	1.009063676
**Protein Chmp4bl1 Chmp4bl1**	tr|D4A9Z8|D4A9Z8_RAT	25 kDa	5.077	1	3.8821	6.0125	2.8586	1	0.988412964	1.008914913
**60S ribosomal protein L31 Rpl31**	sp|P62902|RL31_RAT	14 kDa	2.0308	2.0878	2.9116	2.0042	1.9057	3.0629	0.969289576	1.008231987
**26S proteasome non-ATPase regulatory subunit 9 Psmd9**	sp|Q9WTV5|PSMD9_RAT (+1)	25 kDa	2.0308	2.0878	2.9116	2.0042	1.9057	3.0629	0.969289576	1.008231987
**Arylsulfatase B Arsb**	sp|P50430|ARSB_RAT	59 kDa	2.0308	1	1	2.0042	1	1	0.986138013	1.006643025
**STAM-binding protein Stambp**	sp|Q8R424|STABP_RAT	49 kDa	2.0308	1	1	2.0042	1	1	0.986138013	1.006643025
**Trifunctional enzyme subunit beta, mitochondrial Hadhb**	sp|Q60587|ECHB_RAT	51 kDa	19.293	21.922	20.381	21.044	22.869	17.356	0.954412737	1.00533712
**Exportin-1 Xpo1**	sp|Q80U96|XPO1_RAT	123 kDa	2.0308	1	1.9411	1	1.9057	2.0419	0.986917159	1.004911472
**Protein pelota homolog Pelo**	sp|Q5XIP1|PELO_RAT	43 kDa	1	2.0878	1.9411	3.0063	1	1	0.992472762	1.004514312
**Glutamine synthetase Glul**	sp|P09606|GLNA_RAT	42 kDa	22.339	25.054	26.204	26.054	24.774	22.461	0.950530363	1.004202541
**Protein Dctn3 Dctn3**	tr|D4A1B8|D4A1B8_RAT	21 kDa	4.0616	3.1317	3.8821	3.0063	1.9057	6.1258	0.99274451	1.003406476
**Maleylacetoacetate isomerase Gstz1**	sp|P57113|MAAI_RAT	24 kDa	2.0308	1	4.8526	2.0042	3.8114	2.0419	0.995006634	1.003296214
**Charged multivesicular body protein 5 Chmp5**	sp|Q4QQV8|CHMP5_RAT	25 kDa	1	1	3.8821	2.0042	2.8586	1	0.995616264	1.003291942
**Prohibitin Phb**	sp|P67779|PHB_RAT	30 kDa	16.246	18.79	13.587	14.029	18.104	16.335	0.979715172	1.003197986
**Protein Hnrnpul2 Hnrnpul2**	tr|D4ABT8|D4ABT8_RAT	85 kDa	3.0462	1	1.9411	1	1.9057	3.0629	0.994436342	1.003133063
**Isoform 2 of Polypyrimidine tract-binding protein 2 Ptbp2**	sp|Q66H20-2|PTBP2_RAT (+1)	58 kDa	2.0308	2.0878	1.9411	3.0063	1	2.0419	0.995050695	1.001901392
**Ras-related protein Rap-1A Rap1a**	sp|P62836|RAP1A_RAT	21 kDa	17.262	16.703	19.411	19.04	20.01	14.293	0.995768577	1.000618638
**Farnesyltransferase, CAAX box, alpha Fnta**	tr|Q5RKJ4|Q5RKJ4_RAT	44 kDa	3.0462	1	3.8821	1	2.8586	4.0839	0.997136151	0.99821215
**T-complex protein 1 subunit delta Cct4**	sp|Q7TPB1|TCPD_RAT	58 kDa	17.262	16.703	19.411	18.038	18.104	17.356	0.964524936	0.997719541
**Oxysterol-binding protein Osbp**	tr|D4A9D8|D4A9D8_RAT	78 kDa	2.0308	1	1	1	1	2.0419	0.99431996	0.997253767
**Pyrroline-5-carboxylate reductase 3 Pycrl**	sp|Q5PQJ6|P5CR3_RAT	29 kDa	2.0308	1	1	1	1	2.0419	0.99431996	0.997253767
**Protein LOC100911431 LOC100911431**	tr|D3ZU13|D3ZU13_RAT (+1)	176 kDa	2.0308	1	1	1	1	2.0419	0.99431996	0.997253767
**Acyl-Coenzyme A binding domain containing 3 Acbd3**	tr|G3V6E4|G3V6E4_RAT	60 kDa	1	2.0878	1.9411	2.0042	1	2.0419	0.99108343	0.996591427
**ADP/ATP translocase 2 Slc25a5**	sp|Q09073|ADT2_RAT	33 kDa	18.277	13.571	17.469	16.033	17.151	16.335	0.966138252	0.995920758
**Actin, cytoplasmic 1 Actb**	sp|P60711|ACTB_RAT (+1)	42 kDa	82.248	85.6	87.347	84.175	91.474	80.656	0.921336656	0.995669222
**Transcription elongation factor B polypeptide 2 Tceb2**	sp|P62870|ELOB_RAT	13 kDa	2.0308	1	2.9116	1	1.9057	3.0629	0.991947027	0.995610361
**ATPase, H+ transporting, lysosomal 38kDa, V0 subunit d1 Atp6v0d1**	tr|Q5M7T6|Q5M7T6_RAT	40 kDa	2.0308	2.0878	6.7937	3.0063	2.8586	5.1048	0.991738523	0.994767405
**ATP synthase subunit delta, mitochondrial Atp5d**	tr|G3V7Y3|G3V7Y3_RAT	18 kDa	1	2.0878	1.9411	1	1	3.0629	0.988924853	0.993284481
**Glutamate--cysteine ligase regulatory subunit Gclm**	sp|P48508|GSH0_RAT	31 kDa	2.0308	1	1.9411	3.0063	1	1	0.988466346	0.993128658
**Glutathione S-transferase P Gstp1**	sp|P04906|GSTP1_RAT	23 kDa	14.216	11.483	13.587	13.027	14.293	12.252	0.929905332	0.992772668
**Lysine--tRNA ligase Kars**	tr|Q5XIM7|Q5XIM7_RAT	72 kDa	1	1	2.9116	1	1.9057	2.0419	0.98743432	0.992723745
**Sorting and assembly machinery component 50 homolog Samm50**	sp|Q6AXV4|SAM50_RAT	52 kDa	2.0308	1	5.8232	3.0063	2.8586	3.0629	0.987430093	0.991733686
**Tripartite motif-containing protein 2 Trim2**	sp|D3ZQG6|TRIM2_RAT	81 kDa	10.154	11.483	10.676	13.027	11.434	8.1677	0.946783542	0.990324469
**Rho GDP-dissociation inhibitor 1 Arhgdia**	sp|Q5XI73|GDIR1_RAT	23 kDa	12.185	9.3952	13.587	13.027	13.34	9.1887	0.946583611	0.989073482
**60S ribosomal protein L32 Rpl32**	sp|P62912|RL32_RAT	16 kDa	2.0308	2.0878	3.8821	3.0063	1	4.0839	0.979457029	0.988937233
**Succinate dehydrogenase [ubiquinone] iron-sulfur subunit, mitochondrial Sdhb**	sp|P21913|DHSB_RAT	32 kDa	11.169	9.3952	8.7347	10.021	9.5286	10.21	0.848682004	0.984519281
**Unconventional myosin-Ic Myo1c**	sp|Q63355|MYO1C_RAT	120 kDa	1	1	1.9411	2.0042	1	1	0.965628337	0.984241546
**NADPH:adrenodoxin oxidoreductase, mitochondrial Fdxr**	sp|P56522|ADRO_RAT	54 kDa	1	1	1.9411	2.0042	1	1	0.965628337	0.984241546
**ATP-binding cassette, sub-family E (OABP), member 1 Abce1**	tr|D3ZD23|D3ZD23_RAT	67 kDa	1	1	1.9411	2.0042	1	1	0.965628337	0.984241546
**40S ribosomal protein S20 Rps20**	sp|P60868|RS20_RAT (+1)	13 kDa	3.0462	1	2.9116	3.0063	1	3.0629	0.970601398	0.984241498
**Glycerol-3-phosphate dehydrogenase [NAD(+)], cytoplasmic Gpd1**	sp|O35077|GPDA_RAT	38 kDa	21.323	15.659	18.44	20.042	19.057	17.356	0.858663207	0.981702241
**ADP/ATP translocase 1 Slc25a4**	sp|Q05962|ADT1_RAT (+1)	33 kDa	19.293	16.703	18.44	18.038	18.104	19.398	0.697713516	0.980122434
**Elongation factor 2 Eef2**	sp|P05197|EF2_RAT	95 kDa	21.323	25.054	27.175	24.05	27.633	23.482	0.814684563	0.978540544
**Ras-related C3 botulinum toxin substrate 1 Rac1**	sp|Q6RUV5|RAC1_RAT	21 kDa	7.1078	7.3073	7.7642	7.0146	8.5757	7.1467	0.746305786	0.975471698
**Isoform Short of Disks large homolog 3 Dlg3**	sp|Q62936-2|DLG3_RAT (+1)	92 kDa	1	1	1.9411	1	1	2.0419	0.946211711	0.975061234
**Glycolipid transfer protein GLTP**	sp|B0BNM9|GLTP_RAT	24 kDa	1	1	1.9411	1	1	2.0419	0.946211711	0.975061234
**Protein Tsta3 Tsta3**	tr|G3V762|G3V762_RAT	36 kDa	1	1	1.9411	1	1	2.0419	0.946211711	0.975061234
**GMP reductase Gmpr**	tr|F1LRV6|F1LRV6_RAT	37 kDa	1	1	1.9411	1	1	2.0419	0.946211711	0.975061234
**Galactosylceramidase Galc**	tr|G3V6H1|G3V6H1_RAT	75 kDa	1	1	1.9411	1	1	2.0419	0.946211711	0.975061234
**Catenin delta-2 (Fragment) Ctnnd2**	tr|F1M787|F1M787_RAT	93 kDa	1	1	1.9411	1	1	2.0419	0.946211711	0.975061234
**Bifunctional ATP-dependent dihydroxyacetone kinase/FAD-AMP lyase (cyclizing) Dak**	sp|Q4KLZ6|DHAK_RAT	59 kDa	1	1	1.9411	1	1	2.0419	0.946211711	0.975061234
**WD repeat-containing protein 61 Wdr61**	sp|Q4V7A0|WDR61_RAT	34 kDa	1	1	1.9411	1	1	2.0419	0.946211711	0.975061234
**Protein Ddx6 Ddx6**	tr|D3ZD73|D3ZD73_RAT	54 kDa	1	1	1.9411	1	1	2.0419	0.946211711	0.975061234
**Isocitrate dehydrogenase [NADP], mitochondrial Idh2**	sp|P56574|IDHP_RAT	51 kDa	20.308	21.922	20.381	25.052	21.916	17.356	0.815799898	0.973369193
**Heterogeneous nuclear ribonucleoprotein K Hnrnpk**	sp|P61980|HNRPK_RAT (+1)	51 kDa	21.323	20.878	18.44	20.042	21.916	20.419	0.615187705	0.972169229
**26S protease regulatory subunit 4 Psmc1**	sp|P62193|PRS4_RAT	49 kDa	5.077	9.3952	8.7347	8.0167	6.67	9.1887	0.891064264	0.972000469
**40S ribosomal protein S8 Rps8**	sp|P62243|RS8_RAT	24 kDa	5.077	7.3073	6.7937	6.0125	7.6229	6.1258	0.830490484	0.970487622
**Protein Ahcyl1 Ahcyl1**	tr|D4A5X8|D4A5X8_RAT	53 kDa	22.339	19.834	20.381	24.05	20.01	20.419	0.689343297	0.970145319
**Ras-related protein Rab-1A Rab1A**	sp|Q6NYB7|RAB1A_RAT	23 kDa	6.0924	7.3073	7.7642	5.0104	7.6229	9.1887	0.875780834	0.969842361
**Isoform Delta 7 of Calcium/calmodulin-dependent protein kinase type II subunit delta Camk2d**	sp|P15791-7|KCC2D_RAT (+7)	56 kDa	7.1078	6.2634	11.646	9.0188	7.6229	9.1887	0.883972166	0.968517716
**Brain acid soluble protein 1 Basp1**	sp|Q05175|BASP1_RAT	22 kDa	12.185	14.615	14.558	16.033	12.387	14.293	0.749937108	0.968276637
**Kinesin-1 heavy chain Kif5b**	sp|Q2PQA9|KINH_RAT	110 kDa	15.231	10.439	16.499	11.023	16.199	16.335	0.8644329	0.96813371
**Isoform 2 of Elongation factor 1-delta Eef1d**	sp|Q68FR9-2|EF1D_RAT	72 kDa	8.1232	6.2634	4.8526	7.0146	4.7643	8.1677	0.872333077	0.964535309
**DnaJ homolog subfamily A member 1 Dnaja1**	sp|P63036|DNJA1_RAT	45 kDa	5.077	6.2634	6.7937	6.0125	6.67	6.1258	0.702017924	0.964154123
**Protein Cct7 Cct7**	tr|D4AC23|D4AC23_RAT	60 kDa	15.231	19.834	21.352	21.044	18.104	19.398	0.743963702	0.963635432
**Electron transfer flavoprotein subunit beta Etfb**	sp|Q68FU3|ETFB_RAT	28 kDa	13.2	10.439	9.7053	12.025	12.387	10.21	0.75219028	0.963095719
**Elongation factor 1-gamma Eef1g**	sp|Q68FR6|EF1G_RAT	50 kDa	8.1232	12.527	12.617	9.0188	14.293	11.231	0.851528293	0.963071899
**Proteasome subunit alpha type Psma5**	tr|Q6P9V6|Q6P9V6_RAT	26 kDa	6.0924	7.3073	5.8232	9.0188	3.8114	7.1467	0.882048369	0.962256406
**Succinyl-CoA:3-ketoacid coenzyme A transferase 1, mitochondrial Oxct1**	sp|B2GV06|SCOT1_RAT	56 kDa	12.185	9.3952	10.676	11.023	12.387	10.21	0.68071826	0.95943486
**Isoform 2B of GTPase KRas Kras**	sp|P08644-2|RASK_RAT	21 kDa	7.1078	5.2195	5.8232	7.0146	4.7643	7.1467	0.799661761	0.959044892
**Heat shock cognate 71 kDa protein Hspa8**	sp|P63018|HSP7C_RAT	71 kDa	67.017	60.547	67.937	63.131	68.606	72.488	0.461498827	0.957282409
**Protein lin-7 homolog C Lin7c**	sp|Q792I0|LIN7C_RAT	22 kDa	4.0616	6.2634	3.8821	6.0125	4.7643	4.0839	0.830095072	0.956018223
**Propionyl-CoA carboxylase alpha chain, mitochondrial Pcca**	tr|D4A882|D4A882_RAT (+1)	71 kDa	6.0924	5.2195	5.8232	5.0104	4.7643	8.1677	0.822820226	0.955006019
**Isoform 2 of Dihydropyrimidinase-related protein 3 Dpysl3**	sp|Q62952-2|DPYL3_RAT	74 kDa	36.554	33.405	35.909	35.073	37.161	38.797	0.299061893	0.953499473
**Transketolase Tkt**	sp|P50137|TKT_RAT	68 kDa	37.57	34.449	35.909	37.077	34.303	41.86	0.498914078	0.953090781
**Peptidyl-prolyl cis-trans isomerase Fkbp2**	tr|D3ZZR9|D3ZZR9_RAT	15 kDa	6.0924	4.1756	5.8232	7.0146	4.7643	5.1048	0.788623216	0.953061237
**Transketolase Tkt**	tr|G3V826|G3V826_RAT	71 kDa	37.57	34.449	34.939	37.077	33.35	41.86	0.538890465	0.952541256
**Thiosulfate sulfurtransferase Tst**	sp|P24329|THTR_RAT	33 kDa	9.1386	9.3952	9.7053	12.025	10.481	7.1467	0.761304463	0.952328119
**Ras-related protein Rab-21 Rab21**	sp|Q6AXT5|RAB21_RAT	24 kDa	5.077	4.1756	4.8526	4.0083	5.7171	5.1048	0.692661893	0.951113269
**Uncharacterized protein (Fragment)**	tr|F1M9N9|F1M9N9_RAT	427 kDa	51.786	42.8	45.615	51.106	45.737	51.048	0.467391751	0.948002245
**Transitional endoplasmic reticulum ATPase Vcp**	sp|P46462|TERA_RAT	89 kDa	44.678	43.844	45.615	45.094	47.643	49.006	0.113558588	0.946339502
**D-dopachrome decarboxylase Ddt**	sp|P80254|DOPD_RAT	13 kDa	8.1232	8.3513	9.7053	12.025	9.5286	6.1258	0.792717365	0.945822525
**Stress-70 protein, mitochondrial Hspa9**	tr|F1M953|F1M953_RAT (+1)	74 kDa	29.447	29.229	24.263	27.056	32.397	28.587	0.504430213	0.942060427
**Thioredoxin-like protein 1 Txnl1**	sp|Q920J4|TXNL1_RAT	32 kDa	7.1078	9.3952	8.7347	10.021	7.6229	9.1887	0.615539227	0.940561109
**V-type proton ATPase subunit C 1 Atp6v1c1**	sp|Q5FVI6|VATC1_RAT	44 kDa	4.0616	3.1317	4.8526	3.0063	5.7171	4.0839	0.798817365	0.94054953
**ATP synthase subunit O, mitochondrial Atp5o**	sp|Q06647|ATPO_RAT	23 kDa	21.323	20.878	19.411	22.046	20.01	23.482	0.322749636	0.940095822
**Uncharacterized protein**	tr|F1LM42|F1LM42_RAT	434 kDa	49.755	40.712	44.644	49.102	45.737	49.006	0.363543054	0.939281866
**Protein Rab5c Rab5c**	tr|B0BNK1|B0BNK1_RAT	23 kDa	8.1232	8.3513	5.8232	9.0188	7.6229	7.1467	0.639983309	0.937335004
**Voltage-dependent anion-selective channel protein 3 Vdac3**	sp|Q9R1Z0|VDAC3_RAT	31 kDa	8.1232	6.2634	9.7053	8.0167	9.5286	8.1677	0.650363531	0.93695407
**Peroxiredoxin-6 Prdx6**	sp|O35244|PRDX6_RAT	25 kDa	16.246	14.615	18.44	16.033	16.199	20.419	0.571304155	0.936373478
**Serine/threonine-protein phosphatase 2A 55 kDa regulatory subunit B alpha isoform Ppp2r2a**	sp|P36876|2ABA_RAT	52 kDa	8.1232	8.3513	5.8232	7.0146	6.67	10.21	0.720542426	0.933169001
**Cytoplasmic dynein 1 light intermediate chain 1 Dync1li1**	sp|Q9QXU8|DC1L1_RAT (+1)	57 kDa	8.1232	6.2634	7.7642	8.0167	8.5757	7.1467	0.494552089	0.933093504
**Glutathione S-transferase Yb-3 Gstm3**	sp|P08009|GSTM4_RAT	26 kDa	12.185	15.659	13.587	13.027	18.104	13.273	0.635788966	0.933046572
**Transgelin-3 Tagln3**	sp|P37805|TAGL3_RAT	23 kDa	23.354	14.615	20.381	18.038	20.01	24.503	0.684270624	0.932838804
**Cofilin-1 Cfl1**	sp|P45592|COF1_RAT	19 kDa	21.323	16.703	17.469	20.042	19.057	20.419	0.417884215	0.932407003
**ATP synthase subunit f, mitochondrial Atp5j2**	sp|D3ZAF6|ATPK_RAT	10 kDa	3.0462	4.1756	2.9116	4.0083	3.8114	3.0629	0.639455425	0.931156158

Calpain small subunit 1 Capns1	sp|Q64537|CPNS1_RAT (+1)	29 kDa	7.1078	8.3513	5.8232	7.0146	6.67	9.1887	0.64744966	0.930442918
Hsc70-interacting protein St13	sp|P50503|F10A1_RAT	41 kDa	11.169	10.439	10.676	11.023	10.481	13.273	0.399158338	0.928314691
Glyoxalase domain-containing protein 4 Glod4	sp|Q5I0D1|GLOD4_RAT	33 kDa	7.1078	6.2634	8.7347	7.0146	7.6229	9.1887	0.587037753	0.92779797
14-3-3 protein eta Ywhah	sp|P68511|1433F_RAT	28 kDa	20.308	15.659	18.44	19.04	17.151	22.461	0.529695665	0.927623951
Isocitrate dehydrogenase [NADP] cytoplasmic Idh1	sp|P41562|IDHC_RAT	47 kDa	11.169	8.3513	10.676	12.025	11.434	9.1887	0.541326415	0.924913547
Cell division control protein 42 homolog Cdc42	sp|Q8CFN2|CDC42_RAT	21 kDa	10.154	9.3952	11.646	10.021	10.481	13.273	0.517243487	0.923618061
Tropomyosin 1, alpha Tpm1	tr|Q6AZ25|Q6AZ25_RAT	29 kDa	11.169	15.659	12.617	18.038	11.434	13.273	0.66684653	0.922797988
UMP-CMP kinase Cmpk1	sp|Q4KM73|KCY_RAT	22 kDa	6.0924	8.3513	4.8526	6.0125	5.7171	9.1887	0.738596578	0.922460238
Mitochondrial 2-oxoglutarate/malate carrier protein Slc25a11	tr|G3V6H5|G3V6H5_RAT	34 kDa	6.0924	6.2634	7.7642	9.0188	6.67	6.1258	0.614032857	0.922318081
UBX domain-containing protein 1 Ubxn1	sp|Q499N6|UBXN1_RAT	34 kDa	5.077	2.0878	2.9116	4.0083	2.8586	4.0839	0.780000759	0.920151952
Coiled-coil-helix-coiled-coil-helix domain-containing protein 6, mitochondrial Chchd6	sp|D4A7N1|CHCH6_RAT	29 kDa	5.077	1	4.8526	5.0104	3.8114	3.0629	0.835809422	0.919636171
tRNA-splicing ligase RtcB homolog	sp|Q6AYT3|RTCB_RAT	55 kDa	11.169	8.3513	9.7053	9.0188	9.5286	13.273	0.610727299	0.918454828
3-hydroxyisobutyryl-CoA hydrolase, mitochondrial Hibch	sp|Q5XIE6|HIBCH_RAT	43 kDa	3.0462	2.0878	5.8232	5.0104	2.8586	4.0839	0.808463829	0.91669804
Protein Mapk8ip3 Mapk8ip3	tr|E9PSK7|E9PSK7_RAT	146 kDa	3.0462	3.1317	2.9116	1	3.8114	5.1048	0.831472299	0.916631371
Voltage-dependent anion-selective channel protein 1 Vdac1	sp|Q9Z2L0|VDAC1_RAT	31 kDa	31.477	31.317	29.116	29.061	30.491	40.839	0.496965672	0.915520316
Uncharacterized protein	tr|D4A8C2|D4A8C2_RAT (+1)	131 kDa	11.169	6.2634	12.617	12.025	8.5757	12.252	0.700558058	0.914670636
14-3-3 protein theta Ywhaq	sp|P68255|1433T_RAT	28 kDa	17.262	12.527	16.499	15.031	16.199	19.398	0.502283802	0.914276685
Adenylate kinase isoenzyme 4, mitochondrial Ak4	sp|Q9WUS0|KAD4_RAT	25 kDa	2.0308	2.0878	4.8526	3.0063	4.7643	2.0419	0.830249829	0.91426242
Sodium/potassium-transporting ATPase subunit alpha-3 Atp1a3	sp|P06687|AT1A3_RAT	112 kDa	43.662	36.537	51.438	44.092	56.219	43.901	0.51838819	0.912801986
Guanine nucleotide-binding protein G(I)/G(S)/G(T) subunit beta-1 Gnb1	sp|P54311|GBB1_RAT	37 kDa	24.37	21.922	25.234	24.05	25.727	28.587	0.240414251	0.912740544
Caprin-1 Caprin1	sp|Q5M9G3|CAPR1_RAT	78 kDa	4.0616	2.0878	2.9116	4.0083	2.8586	3.0629	0.689168445	0.912505791
Protein Vps4b Vps4b	tr|Q4KLL7|Q4KLL7_RAT	49 kDa	3.0462	2.0878	2.9116	4.0083	3.8114	1	0.812173878	0.912230575
Protein Dusp3 Dusp3	tr|G3V9L3|G3V9L3_RAT	21 kDa	6.0924	5.2195	5.8232	7.0146	6.67	5.1048	0.438687219	0.911955677
Protein DJ-1 Park7	sp|O88767|PARK7_RAT	20 kDa	12.185	11.483	11.646	14.029	11.434	13.273	0.226682604	0.911658406
Biliverdin reductase A Blvra	tr|Q6AZ33|Q6AZ33_RAT	34 kDa	3.0462	1	6.7937	4.0083	3.8114	4.0839	0.844823327	0.910640479
Ribose-phosphate pyrophosphokinase 1 Prps1	sp|P60892|PRPS1_RAT	35 kDa	5.077	6.2634	4.8526	6.0125	6.67	5.1048	0.446713099	0.910368634
Protein Map7d1 Map7d1	tr|D4A644|D4A644_RAT	90 kDa	3.0462	3.1317	1.9411	3.0063	2.8586	3.0629	0.525622368	0.909406573
Cadherin-2 Cdh2	sp|Q9Z1Y3|CADH2_RAT (+1)	100 kDa	4.0616	1	3.8821	4.0083	3.8114	2.0419	0.807025382	0.906921798
Elongation factor Tu, mitochondrial Tufm	sp|P85834|EFTU_RAT	50 kDa	18.277	19.834	20.381	23.048	18.104	23.482	0.327378838	0.904972615
28 kDa heat- and acid-stable phosphoprotein Pdap1	sp|Q62785|HAP28_RAT	21 kDa	2.0308	3.1317	2.9116	3.0063	2.8586	3.0629	0.451927092	0.904377338
Proteasome subunit beta type-2 Psmb2	sp|P40307|PSB2_RAT	23 kDa	2.0308	2.0878	4.8526	4.0083	2.8586	3.0629	0.764480496	0.903462305
Fructose-bisphosphate aldolase C Aldoc	sp|P09117|ALDOC_RAT	39 kDa	59.909	53.239	63.084	73.152	62.889	59.216	0.279984309	0.902564313
Coronin (Fragment) Coro2b	tr|F1LMV9|F1LMV9_RAT	55 kDa	4.0616	2.0878	1.9411	3.0063	1.9057	4.0839	0.761536407	0.89935415
Protein Ndufb10 Ndufb10	tr|D4A0T0|D4A0T0_RAT	21 kDa	10.154	10.439	10.676	11.023	10.481	13.273	0.249182788	0.899128735
Isoform M2 of Pyruvate kinase isozymes M1/M2 Pkm	sp|P11980-2|KPYM_RAT	58 kDa	87.325	73.073	81.524	87.182	94.333	87.803	0.125596382	0.898276387
D-3-phosphoglycerate dehydrogenase Phgdh	sp|O08651|SERA_RAT	56 kDa	17.262	14.615	20.381	21.044	20.01	17.356	0.362253475	0.894675569
Sorbitol dehydrogenase Sord	sp|P27867|DHSO_RAT	38 kDa	7.1078	2.0878	4.8526	4.0083	7.6229	4.0839	0.782127196	0.893930042
Protein S100-B S100b	sp|P04631|S100B_RAT	11 kDa	16.246	13.571	14.558	18.038	14.293	17.356	0.272127168	0.893090748
Protein Myh14 Myh14	tr|F1LNF0|F1LNF0_RAT	229 kDa	14.216	10.439	11.646	12.025	13.34	15.314	0.376334958	0.892376902
Septin-2 Sept2	sp|Q91Y81|SEPT2_RAT	42 kDa	16.246	13.571	12.617	18.038	15.246	14.293	0.334270263	0.891901549
Protein NDRG1 Ndrg1	sp|Q6JE36|NDRG1_RAT	43 kDa	12.185	12.527	9.7053	14.029	13.34	11.231	0.318350599	0.891639896
Protein Ubxn6 Ubxn6	tr|M0RAE4|M0RAE4_RAT	45 kDa	2.0308	3.1317	1.9411	3.0063	1.9057	3.0629	0.617344019	0.890744712
Guanine nucleotide-binding protein G(i) subunit alpha-2 Gnai2	sp|P04897|GNAI2_RAT	41 kDa	14.216	8.3513	14.558	13.027	13.34	15.314	0.516705298	0.890700799
Eukaryotic peptide chain release factor subunit 1 Etf1	sp|Q5U2Q7|ERF1_RAT	49 kDa	2.0308	2.0878	1.9411	2.0042	3.8114	1	0.774929872	0.88909267
Isoform 2B of Cytoplasmic dynein 1 intermediate chain 2 Dync1i2	sp|Q62871-2|DC1I2_RAT (+1)	70 kDa	7.1078	10.439	7.7642	9.0188	13.34	6.1258	0.673433688	0.888585411
Pleckstrin homology domain-containing family B member 1 Plekhb1	sp|Q9WU68|PKHB1_RAT	25 kDa	3.0462	1	3.8821	3.0063	2.8586	3.0629	0.717650511	0.888046327
NADH dehydrogenase (Ubiquinone) Fe-S protein 8 (Predicted), isoform CRA_a Ndufs8	tr|B0BNE6|B0BNE6_RAT	24 kDa	9.1386	9.3952	8.7347	12.025	9.5286	9.1887	0.274213974	0.887002599
Isoform 2 of Cell division control protein 42 homolog Cdc42	sp|Q8CFN2-2|CDC42_RAT	21 kDa	7.1078	6.2634	7.7642	6.0125	7.6229	10.21	0.524623431	0.886351246
ADP-ribosylation factor 3 Arf3	sp|P61206|ARF3_RAT (+1)	21 kDa	14.216	8.3513	12.617	13.027	12.387	14.293	0.458100571	0.886098169
T-complex protein 1 subunit gamma Cct3	sp|Q6P502|TCPG_RAT	61 kDa	21.323	16.703	15.528	22.046	18.104	20.419	0.329157254	0.884181677
Phosphoglycerate kinase 1 Pgk1	sp|P16617|PGK1_RAT	45 kDa	36.554	35.493	35.909	39.081	38.114	44.922	0.09299647	0.88403744
Vacuolar protein sorting-associated protein 29 Vps29	sp|B2RZ78|VPS29_RAT	20 kDa	4.0616	5.2195	3.8821	4.0083	4.7643	6.1258	0.482514339	0.883531117
Fumarylacetoacetate hydrolase domain-containing protein 2 Fahd2	sp|B2RYW9|FAHD2_RAT	35 kDa	10.154	8.3513	9.7053	14.029	6.67	11.231	0.605048665	0.883513937
Guanine nucleotide-binding protein G(I)/G(S)/G(T) subunit beta-2 Gnb2	sp|P54313|GBB2_RAT	37 kDa	24.37	21.922	26.204	28.058	27.633	26.545	0.069669165	0.881560387
Diphosphoinositol polyphosphate phosphohydrolase 1 Nudt3	sp|Q566C7|NUDT3_RAT	19 kDa	2.0308	2.0878	2.9116	3.0063	1.9057	3.0629	0.541243717	0.881540834
Dynactin subunit 2 Dctn2	sp|Q6AYH5|DCTN2_RAT	44 kDa	14.216	15.659	16.499	15.031	16.199	21.44	0.370154394	0.880463262
Protein Ank3 Ank3	tr|F1LNM3|F1LNM3_RAT (+1)	282 kDa	11.169	5.2195	10.676	12.025	9.5286	9.1887	0.591656623	0.880366791
T-complex protein 1 subunit beta Cct2	sp|Q5XIM9|TCPB_RAT	57 kDa	29.447	27.142	27.175	29.061	31.444	34.713	0.102180967	0.879707618
Uncharacterized protein	tr|D3ZFD0|D3ZFD0_RAT	233 kDa	5.077	4.1756	2.9116	3.0063	5.7171	5.1048	0.619942839	0.879666189
Isoaspartyl peptidase/L-asparaginase Asrgl1	sp|Q8VI04|ASGL1_RAT	34 kDa	7.1078	5.2195	7.7642	8.0167	6.67	8.1677	0.363596218	0.879108618
Ubiquilin-1 Ubqln1	sp|Q9JJP9|UBQL1_RAT	62 kDa	2.0308	1	2.9116	1	4.7643	1	0.851359241	0.878494449
60S ribosomal protein L11 Rpl11	sp|P62914|RL11_RAT	20 kDa	2.0308	3.1317	1.9411	3.0063	1	4.0839	0.754371941	0.878049986
Protein Ppp2r4 Ppp2r4	tr|B2RYQ2|B2RYQ2_RAT	37 kDa	3.0462	5.2195	3.8821	6.0125	4.7643	3.0629	0.624021928	0.87775024
GTP-binding nuclear protein Ran Ran	sp|P62828|RAN_RAT	24 kDa	8.1232	9.3952	7.7642	9.0188	8.5757	11.231	0.285521552	0.877091464
Protein LOC100360501 LOC100360501	tr|E2RUH2|E2RUH2_RAT	50 kDa	11.169	12.527	13.587	16.033	15.246	11.231	0.348824742	0.877040696
Cytoplasmic FMR1 interacting protein 1 (Predicted) Cyfip1	tr|D4A8H8|D4A8H8_RAT	145 kDa	2.0308	2.0878	1.9411	1	2.8586	3.0629	0.684785561	0.875489417
Hydroxyacyl-coenzyme A dehydrogenase, mitochondrial Hadh	sp|Q9WVK7|HCDH_RAT	34 kDa	9.1386	5.2195	10.676	10.021	11.434	7.1467	0.594104566	0.875266156
Isoform 2 of Plasma membrane calcium-transporting ATPase 2 Atp2b2	sp|P11506-14|AT2B2_RAT (+13)	133 kDa	4.0616	1	8.7347	4.0083	6.67	5.1048	0.794420807	0.874118519
Proteasome subunit alpha type-3 Psma3	sp|P18422|PSA3_RAT (+1)	28 kDa	2.0308	2.0878	1	2.0042	2.8586	1	0.719285233	0.873064065
Peflin Pef1	sp|Q641Z8|PEF1_RAT	30 kDa	2.0308	2.0878	1	2.0042	2.8586	1	0.719285233	0.873064065
Succinyl-CoA ligase [ADP/GDP-forming] subunit alpha, mitochondrial Suclg1	sp|P13086|SUCA_RAT	36 kDa	15.231	13.571	14.558	17.035	14.293	18.377	0.177734352	0.872346846
Protein Kbtbd11 Kbtbd11	tr|D4A5J1|D4A5J1_RAT	68 kDa	3.0462	1	1.9411	3.0063	2.8586	1	0.755048797	0.872161284
Fructose-bisphosphate aldolase A Aldoa	sp|P05065|ALDOA_RAT	39 kDa	51.786	52.195	58.232	62.129	64.794	59.216	0.038854584	0.87146165
Isoform 2 of Serine/threonine-protein kinase DCLK2 Dclk2	sp|Q5MPA9-2|DCLK2_RAT (+3)	78 kDa	2.0308	2.0878	1.9411	3.0063	1.9057	2.0419	0.441235383	0.871410288
NADH dehydrogenase [ubiquinone] 1 subunit C2 Ndufc2	tr|Q5PQZ9|Q5PQZ9_RAT	14 kDa	4.0616	4.1756	3.8821	5.0104	3.8114	5.1048	0.229280282	0.87022676
CAP-Gly domain-containing linker protein 2 Clip2	tr|G3V949|G3V949_RAT	116 kDa	10.154	10.439	8.7347	10.021	11.434	12.252	0.156515285	0.870077432
Clathrin light chain B Cltb	sp|P08082|CLCB_RAT	25 kDa	6.0924	5.2195	5.8232	5.0104	7.6229	7.1467	0.355056904	0.866284125
Retinal dehydrogenase 1 Aldh1a1	sp|P51647|AL1A1_RAT	54 kDa	3.0462	1	1.9411	1	2.8586	3.0629	0.742237798	0.865029257
Heat shock protein HSP 90-alpha Hsp90aa1	sp|P82995|HS90A_RAT	85 kDa	45.693	46.976	49.497	55.115	52.407	57.174	0.013382539	0.863202506
Cell cycle exit and neuronal differentiation protein 1 Cend1	sp|Q5FVI4|CEND_RAT	15 kDa	7.1078	6.2634	10.676	8.0167	7.6229	12.252	0.557699778	0.862166387
ADP-ribosylation factor 2 Arf2	sp|P84082|ARF2_RAT	21 kDa	9.1386	5.2195	8.7347	8.0167	8.5757	10.21	0.429345526	0.861594484
Poly(RC) binding protein 3 Pcbp3	tr|Q6AY48|Q6AY48_RAT	34 kDa	5.077	6.2634	5.8232	8.0167	4.7643	7.1467	0.422407648	0.861293576
10 kDa heat shock protein, mitochondrial Hspe1	sp|P26772|CH10_RAT	11 kDa	3.0462	4.1756	2.9116	4.0083	5.7171	2.0419	0.656372035	0.861149117
Protein phosphatase 1 regulatory subunit 7 Ppp1r7	sp|Q5HZV9|PP1R7_RAT	41 kDa	9.1386	6.2634	6.7937	8.0167	8.5757	9.1887	0.275079142	0.86092913
Protein phosphatase 1F Ppm1f	sp|Q9WVR7|PPM1F_RAT	49 kDa	6.0924	4.1756	5.8232	5.0104	8.5757	5.1048	0.546536935	0.860910925
Guanine nucleotide-binding protein subunit beta-4 Gnb4	sp|O35353|GBB4_RAT	37 kDa	15.231	13.571	15.528	19.04	16.199	16.335	0.094727024	0.85954163
Cdc42 effector protein 1 Cdc42ep1	sp|A1A5P0|BORG5_RAT	41 kDa	4.0616	4.1756	2.9116	5.0104	2.8586	5.1048	0.507719171	0.859331884
Heat shock 70 kDa protein 4 Hspa4	sp|O88600|HSP74_RAT	94 kDa	26.4	28.185	26.204	30.063	32.397	31.65	0.008914743	0.858452874
Guanine nucleotide-binding protein G(o) subunit alpha Gnao1	tr|D4ABT0|D4ABT0_RAT (+1)	40 kDa	18.277	12.527	19.411	16.033	20.01	22.461	0.385256024	0.85831738
Creatine kinase B-type Ckb	sp|P07335|KCRB_RAT	43 kDa	73.109	68.898	74.73	83.173	90.521	79.635	0.028711422	0.855555424
Isoform 2 of Voltage-dependent anion-selective channel protein 3 Vdac3	sp|Q9R1Z0-2|VDAC3_RAT	31 kDa	7.1078	5.2195	9.7053	8.0167	8.5757	9.1887	0.405045669	0.85460279
Kinesin light chain 4 Klc4	sp|Q5PQM2|KLC4_RAT	69 kDa	1	3.1317	1	4.0083	1	1	0.823753816	0.854101826
Dihydrolipoamide branched chain transacylase E2 Dbt	tr|B2GV15|B2GV15_RAT	53 kDa	2.0308	5.2195	2.9116	4.0083	3.8114	4.0839	0.575754932	0.853682919
Succinate dehydrogenase [ubiquinone] flavoprotein subunit, mitochondrial Sdha	sp|Q920L2|DHSA_RAT	72 kDa	16.246	21.922	12.617	23.048	18.104	18.377	0.406825941	0.853113608
Protein LOC100911440 Slc25a22	tr|Q5FVG4|Q5FVG4_RAT	25 kDa	1	2.0878	1.9411	3.0063	1.9057	1	0.684308113	0.850625846
Protein Pmp2 Pmp2	tr|D3ZFG5|D3ZFG5_RAT	15 kDa	6.0924	6.2634	8.7347	8.0167	7.6229	9.1887	0.270263651	0.84945405
Heterogeneous nuclear ribonucleoprotein D-like Hnrpdl	sp|Q3SWU3|HNRDL_RAT	35 kDa	1	3.1317	1	3.0063	1	2.0419	0.755697607	0.848467313
Protein RGD1562502 Pbdc1	tr|G3V6C3|G3V6C3_RAT	22 kDa	2.0308	1	1.9411	2.0042	2.8586	1	0.662037884	0.848041891
Peptidyl-prolyl cis-trans isomerase A Ppia	sp|P10111|PPIA_RAT	18 kDa	29.447	29.229	27.175	34.071	30.491	36.755	0.057633921	0.847350395
Serine/threonine-protein kinase PAK 2 Pak2	sp|Q64303|PAK2_RAT	58 kDa	8.1232	8.3513	7.7642	10.021	10.481	8.1677	0.11213658	0.845446586
Protein LOC100912917 Pfas	tr|D4AB17|D4AB17_RAT	137 kDa	1	1	2.9116	1	3.8114	1	0.804343666	0.845166397
Peptidyl-prolyl cis-trans isomerase FKBP8 Fkbp8	sp|Q3B7U9|FKBP8_RAT	44 kDa	1	1	2.9116	1	3.8114	1	0.804343666	0.845166397
Leucine-rich repeat-containing protein 57 Lrrc57	sp|Q5FVI3|LRC57_RAT	27 kDa	1	2.0878	1.9411	2.0042	1.9057	2.0419	0.420728619	0.844937666
ADP-ribosylation factor 6 Arf6	sp|P62332|ARF6_RAT	20 kDa	1	2.0878	1.9411	2.0042	1.9057	2.0419	0.420728619	0.844937666
Isoform 2 of Adenylate kinase 2, mitochondrial Ak2	sp|P29410-2|KAD2_RAT (+1)	26 kDa	1	2.0878	1.9411	2.0042	1.9057	2.0419	0.420728619	0.844937666
Protein Sptbn1 Sptbn1	tr|G3V6S0|G3V6S0_RAT	273 kDa	91.386	90.82	95.111	111.23	101.96	115.37	0.015100367	0.844037619
T-complex protein 1 subunit epsilon Cct5	sp|Q68FQ0|TCPE_RAT	60 kDa	15.231	17.746	16.499	20.042	17.151	21.44	0.104196994	0.84382515
Pyruvate kinase isozymes M1/M2 Pkm	sp|P11980|KPYM_RAT	58 kDa	84.278	70.986	79.583	93.194	94.333	90.866	0.022600493	0.843580837
Cytochrome b-c1 complex subunit 2, mitochondrial Uqcrc2	sp|P32551|QCR2_RAT	48 kDa	16.246	16.703	15.528	23.048	18.104	16.335	0.214524442	0.843268913
Cytochrome c oxidase subunit VIIa polypeptide 2 like (Predicted), isoform CRA_e Cox7a2l	tr|D3ZYX8|D3ZYX8_RAT	13 kDa	4.0616	2.0878	3.8821	3.0063	3.8114	5.1048	0.512452651	0.841392325
Proline synthetase co-transcribed (Predicted) Prosc	tr|D3ZCA0|D3ZCA0_RAT	30 kDa	5.077	5.2195	3.8821	5.0104	5.7171	6.1258	0.170792294	0.841295177
Protein Tppp Tppp	tr|D3ZQL7|D3ZQL7_RAT	24 kDa	12.185	7.3073	9.7053	12.025	10.481	12.252	0.288060627	0.840025318
Prefoldin 5 (Predicted), isoform CRA_a Pfdn5	tr|B5DFN4|B5DFN4_RAT	17 kDa	1	2.0878	1.9411	4.0083	1	1	0.773276986	0.836992161
Dihydropteridine reductase Qdpr	sp|P11348|DHPR_RAT	26 kDa	14.216	12.527	15.528	17.035	17.151	16.335	0.038479045	0.83670157
Bleomycin hydrolase Blmh	sp|P70645|BLMH_RAT (+1)	52 kDa	4.0616	3.1317	1.9411	2.0042	3.8114	5.1048	0.613542314	0.836452877
ARP1 actin-related protein 1 homolog B (Yeast) Actr1b	tr|B2RYJ7|B2RYJ7_RAT	42 kDa	9.1386	9.3952	8.7347	12.025	11.434	9.1887	0.112739072	0.835234948
Single-stranded DNA-binding protein, mitochondrial Ssbp1	sp|P28042|SSBP_RAT (+1)	17 kDa	3.0462	3.1317	2.9116	4.0083	3.8114	3.0629	0.112730367	0.835232389
Protein LOC100912106 Pdcd5	tr|D4ADF5|D4ADF5_RAT	14 kDa	3.0462	3.1317	2.9116	4.0083	3.8114	3.0629	0.112730367	0.835232389
Dynein light chain 1, cytoplasmic Dynll1	sp|P63170|DYL1_RAT	10 kDa	4.0616	4.1756	5.8232	6.0125	5.7171	5.1048	0.215370259	0.835218362
Carbonyl reductase [NADPH] 1 Cbr1	sp|P47727|CBR1_RAT	31 kDa	6.0924	7.3073	9.7053	8.0167	10.481	9.1887	0.298092384	0.834525254
Eukaryotic translation elongation factor 1 beta 2 Eef1b2	tr|B5DEN5|B5DEN5_RAT	25 kDa	5.077	4.1756	4.8526	6.0125	4.7643	6.1258	0.143541096	0.834498834
Neural cell adhesion molecule 1 Ncam1	sp|P13596|NCAM1_RAT (+1)	95 kDa	17.262	14.615	24.263	20.042	24.774	22.461	0.308670217	0.834460514

Isovaleryl-CoA dehydrogenase, mitochondrial Ivd	sp|P12007|IVD_RAT	46 kDa	4.0616	4.1756	5.8232	5.0104	5.7171	6.1258	0.228742874	0.834281713
Adapter molecule crk Crk	sp|Q63768|CRK_RAT	34 kDa	6.0924	5.2195	6.7937	7.0146	8.5757	6.1258	0.229092935	0.833740865
Macrophage migration inhibitory factor Mif	sp|P30904|MIF_RAT (+1)	12 kDa	1	2.0878	1	2.0042	1.9057	1	0.601041466	0.832562781
cAMP-dependent protein kinase catalytic subunit beta Prkacb	sp|P68182|KAPCB_RAT	41 kDa	1	2.0878	1	2.0042	1.9057	1	0.601041466	0.832562781
Sideroflexin-5 Sfxn5	sp|Q8CFD0|SFXN5_RAT	37 kDa	1	1	2.9116	1	2.8586	2.0419	0.712785107	0.832404034
Glutathione S-transferase theta-2 Gstt2	sp|P30713|GSTT2_RAT	27 kDa	1	2.0878	1.9411	3.0063	1	2.0419	0.639780192	0.83147052
GTPase HRas Hras1	sp|P20171|RASH_RAT (+1)	21 kDa	4.0616	5.2195	3.8821	4.0083	5.7171	6.1258	0.310436226	0.830422933
Enolase-phosphatase E1 Enoph1	sp|Q5PPH0|ENOPH_RAT	29 kDa	2.0308	3.1317	3.8821	3.0063	3.8114	4.0839	0.379708864	0.829658032
Protein Clip1 Clip1	tr|F1MAH8|F1MAH8_RAT	148 kDa	2.0308	1	1	1	2.8586	1	0.716757307	0.829621702
Protein Kif3a Kif3a	tr|F1LQZ3|F1LQZ3_RAT	80 kDa	2.0308	1	1	1	2.8586	1	0.716757307	0.829621702
NADH dehydrogenase (Ubiquinone) 1 beta subcomplex, 9 Ndufb9	tr|B2RYW3|B2RYW3_RAT	22 kDa	3.0462	5.2195	4.8526	5.0104	5.7171	5.1048	0.270026109	0.828578286
3-hydroxyisobutyrate dehydrogenase, mitochondrial Hibadh	sp|P29266|3HIDH_RAT	35 kDa	13.2	10.439	11.646	12.025	14.293	16.335	0.172179794	0.827257168
Dead end homolog 1 (Zebrafish) Hars	tr|Q4QQV4|Q4QQV4_RAT	57 kDa	8.1232	5.2195	8.7347	9.0188	9.5286	8.1677	0.251648007	0.826401548
Protein Opalin Opalin	tr|Q56A26|Q56A26_RAT	16 kDa	1	2.0878	1	1	1.9057	2.0419	0.588741223	0.826218773
60S ribosomal protein L9 Rpl9	sp|P17077|RL9_RAT	22 kDa	6.0924	6.2634	4.8526	7.0146	6.67	7.1467	0.060828787	0.826083826
Hsp90 co-chaperone Cdc37 Cdc37	sp|Q63692|CDC37_RAT	45 kDa	5.077	4.1756	3.8821	5.0104	4.7643	6.1258	0.170211737	0.826055784
Estradiol 17-beta-dehydrogenase 11 Hsd17b11	sp|Q6AYS8|DHB11_RAT	33 kDa	1	1	2.9116	2.0042	1.9057	2.0419	0.615940152	0.825229342
Myc box-dependent-interacting protein 1 Bin1	tr|D4ABS7|D4ABS7_RAT	49 kDa	20.308	19.834	20.381	26.054	23.821	23.482	0.006576174	0.825047371
Versican core protein (Fragments) Vcan	sp|Q9ERB4|CSPG2_RAT	300 kDa	19.293	13.571	19.411	18.038	22.869	22.461	0.208920121	0.824943189
Protein Sucla2 Sucla2	tr|F1LM47|F1LM47_RAT	50 kDa	6.0924	9.3952	10.676	10.021	10.481	11.231	0.258388636	0.824491854
Cofilin 2, muscle (Predicted), isoform CRA_b Cfl2	tr|M0RC65|M0RC65_RAT	19 kDa	8.1232	7.3073	10.676	10.021	10.481	11.231	0.155739919	0.822692465
L-lactate dehydrogenase A chain Ldha	sp|P04642|LDHA_RAT	36 kDa	8.1232	9.3952	11.646	10.021	15.246	10.21	0.351491404	0.822065
Protein Apoo Apoo	tr|M0R7G4|M0R7G4_RAT (+1)	23 kDa	2.0308	1	1.9411	3.0063	1	2.0419	0.618960396	0.822046229
EF-hand domain-containing protein D2 Efhd2	sp|Q4FZY0|EFHD2_RAT	27 kDa	5.077	6.2634	5.8232	8.0167	5.7171	7.1467	0.175914617	0.821991811
Translationally-controlled tumor protein Tpt1	sp|P63029|TCTP_RAT	19 kDa	8.1232	6.2634	5.8232	9.0188	10.481	5.1048	0.450310359	0.821382993
Protein NDRG2 Ndrg2	sp|Q8VBU2|NDRG2_RAT	41 kDa	10.154	9.3952	14.558	13.027	13.34	15.314	0.225082229	0.818291308
ATP-dependent RNA helicase DDX1 Ddx1	sp|Q641Y8|DDX1_RAT	82 kDa	8.1232	10.439	9.7053	11.023	13.34	10.21	0.144285837	0.817617794
ATP synthase subunit alpha, mitochondrial Atp5a1	sp|P15999|ATPA_RAT	60 kDa	56.863	55.327	57.261	65.136	68.606	73.509	0.007275819	0.81761246
Aminoacyl tRNA synthase complex-interacting multifunctional protein 2 Aimp2	sp|Q32PX2|AIMP2_RAT	35 kDa	1	1	2.9116	4.0083	1	1	0.773672343	0.817469168
Alanyl-tRNA editing protein Aarsd1 Aarsd1	sp|Q5XI97|AASD1_RAT	45 kDa	1	2.0878	1	3.0063	1	1	0.70792678	0.816531171
Aspartate aminotransferase, mitochondrial Got2	sp|P00507|AATM_RAT	47 kDa	31.477	30.273	29.116	45.094	29.539	36.755	0.206802422	0.815761123
Protein SEC13 homolog Sec13	sp|Q5XFW8|SEC13_RAT	36 kDa	2.0308	1	1	1	1.9057	2.0419	0.554467088	0.814698035
Protein Dip2b (Fragment) Dip2b	tr|F1M8R8|F1M8R8_RAT	165 kDa	2.0308	1	1	1	1.9057	2.0419	0.554467088	0.814698035
Apolipoprotein D Apod	tr|M0R4S2|M0R4S2_RAT	22 kDa	4.0616	4.1756	3.8821	5.0104	4.7643	5.1048	0.002269426	0.814496455
sp|K1C9_HUMAN|	zz|ZZ_FGCZCont0099|	62 kDa	17.262	9.3952	16.499	32.067	6.67	14.293	0.69941276	0.813807279
Calmodulin Calm1	sp|P62161|CALM_RAT	17 kDa	13.2	13.571	13.587	15.031	16.199	18.377	0.035574416	0.813554539
Isoform Cytoplasmic of Cysteine desulfurase, mitochondrial Nfs1	sp|Q99P39-2|NFS1_RAT (+2)	44 kDa	3.0462	4.1756	4.8526	3.0063	5.7171	6.1258	0.452176315	0.813134714
Platelet-activating factor acetylhydrolase IB subunit alpha Pafah1b1	sp|P63004|LIS1_RAT	47 kDa	14.216	16.703	12.617	18.038	17.151	18.377	0.054759156	0.812754359
Dihydropyrimidinase-related protein 1 Crmp1	sp|Q62950|DPYL1_RAT	62 kDa	22.339	13.571	20.381	22.046	24.774	22.461	0.195539655	0.812502706
Protein LOC100911774 Tbcb	tr|Q1RP74|Q1RP74_RAT	27 kDa	6.0924	4.1756	5.8232	7.0146	6.67	6.1258	0.130287986	0.812260227
Proteasome subunit beta type Psmb1	tr|Q6PDW4|Q6PDW4_RAT	26 kDa	7.1078	9.3952	6.7937	11.023	9.5286	8.1677	0.195035048	0.811186206
Oligoribonuclease, mitochondrial Rexo2	sp|Q5U1X1|ORN_RAT	27 kDa	1	1	1.9411	1	2.8586	1	0.682393144	0.811159593
Golgi phosphoprotein 3 Golph3	sp|Q9ERE4|GOLP3_RAT (+1)	34 kDa	1	1	1.9411	1	2.8586	1	0.682393144	0.811159593
Protein LOC100359642 LOC100359642	tr|M0RCH6|M0RCH6_RAT	22 kDa	4.0616	4.1756	3.8821	5.0104	3.8114	6.1258	0.234266538	0.810785678
Elongation factor 1-alpha 2 Eef1a2	sp|P62632|EF1A2_RAT	50 kDa	22.339	20.878	20.381	27.056	22.869	28.587	0.051327507	0.810041777
Dihydropyrimidinase-related protein 2 Dpysl2	sp|P47942|DPYL2_RAT	62 kDa	73.109	61.59	76.672	83.173	91.474	86.782	0.031611938	0.808521625
Protein Ube2l3 Ube2l3	tr|B2RZA9|B2RZA9_RAT	18 kDa	8.1232	4.1756	7.7642	8.0167	7.6229	9.1887	0.303084645	0.808069824
Hypoxanthine-guanine phosphoribosyltransferase Hprt1	sp|P27605|HPRT_RAT	24 kDa	6.0924	7.3073	5.8232	8.0167	7.6229	8.1677	0.034384706	0.807437215
Protein RGD1565368 RGD1565368	tr|M0R660|M0R660_RAT	36 kDa	52.801	50.108	55.32	61.127	68.606	66.363	0.009223628	0.806895602
PDZ domain-containing protein GIPC1 Gipc1	sp|Q9Z254|GIPC1_RAT	36 kDa	6.0924	4.1756	4.8526	9.0188	6.67	3.0629	0.542561344	0.80635889
Isoform 2 of Myelin-associated oligodendrocyte basic protein Mobp	sp|Q63327-2|MOBP_RAT (+1)	19 kDa	12.185	8.3513	9.7053	14.029	14.293	9.1887	0.293040272	0.806212627
NADH dehydrogenase [ubiquinone] 1 alpha subcomplex subunit 6 Ndufa6	tr|D4A3V2|D4A3V2_RAT	15 kDa	3.0462	4.1756	4.8526	7.0146	2.8586	5.1048	0.501486459	0.806142342
Putative hydrolase RBBP9 Rbbp9	sp|O88350|RBBP9_RAT	21 kDa	2.0308	1	1	3.0063	1	1	0.687690392	0.805145517
Malate dehydrogenase, cytoplasmic Mdh1	sp|O88989|MDHC_RAT	36 kDa	12.185	13.571	12.617	16.033	14.293	17.356	0.033650646	0.804769095
Thimet oligopeptidase Thop1	sp|P24155|THOP1_RAT	78 kDa	4.0616	4.1756	3.8821	5.0104	1.9057	8.1677	0.614069761	0.803464644
Dihydrolipoyllysine-residue acetyltransferase component of pyruvate dehydrogenase complex, mitochondrial Dlat	sp|P08461|ODP2_RAT	67 kDa	19.293	17.746	19.411	23.048	24.774	22.461	0.006271301	0.803181424
Peroxiredoxin-2 Prdx2	sp|P35704|PRDX2_RAT	22 kDa	11.169	10.439	12.617	14.029	14.293	14.293	0.012360223	0.803120967
26S protease regulatory subunit 6A Psmc3	sp|Q63569|PRS6A_RAT (+1)	49 kDa	7.1078	7.3073	8.7347	9.0188	8.5757	11.231	0.122319198	0.803101421
ATP synthase subunit epsilon, mitochondrial Atp5e	sp|P29418|ATP5E_RAT	6 kDa	1	1	1.9411	2.0042	1.9057	1	0.510729739	0.802684372
Type I inositol 3,4-bisphosphate 4-phosphatase Inpp4a	tr|D3ZAN1|D3ZAN1_RAT (+1)	104 kDa	1	1	1.9411	2.0042	1.9057	1	0.510729739	0.802684372
Hippocalcin-like protein 1 Hpcal1	sp|P62749|HPCL1_RAT	22 kDa	1	1	1.9411	2.0042	1.9057	1	0.510729739	0.802684372
Cytochrome b-c1 complex subunit Rieske, mitochondrial Uqcrfs1	sp|P20788|UCRI_RAT	29 kDa	10.154	9.3952	10.676	13.027	11.434	13.273	0.021790561	0.801007049
Serine/threonine-protein phosphatase 2A 55 kDa regulatory subunit B delta isoform Ppp2r2d	sp|P56932|2ABD_RAT	52 kDa	4.0616	3.1317	3.8821	5.0104	4.7643	4.0839	0.079830499	0.799171633
Protein Arhgap23 Arhgap23	tr|F1M2D4|F1M2D4_RAT	161 kDa	2.0308	1	1	2.0042	1	2.0419	0.523106836	0.798795109
Active BCR-related gene (Predicted) Abr	tr|D4A6K9|D4A6K9_RAT	98 kDa	1	1	1.9411	1	1.9057	2.0419	0.500150618	0.796568033
Annexin A6 Anxa6	sp|P48037|ANXA6_RAT	76 kDa	33.508	29.229	27.175	33.069	40.02	39.818	0.060101181	0.796336808
Isoform AMPH2-2 of Myc box-dependent-interacting protein 1 Bin1	sp|O08839-2|BIN1_RAT (+1)	61 kDa	21.323	19.834	20.381	27.056	24.774	25.524	0.002746435	0.795537399
Protein LOC685778 LOC685778	tr|D4A5G8|D4A5G8_RAT	44 kDa	14.216	13.571	15.528	20.042	18.104	16.335	0.037581596	0.795047815
Annexin (Fragment) Anxa6	tr|D4ABR6|D4ABR6_RAT	76 kDa	30.462	29.229	25.234	31.065	38.114	37.776	0.057799877	0.794025525
Proteasome (Prosome, macropain) 26S subunit, non-ATPase, 5 (Predicted), isoform CRA_a Psmd5	tr|G3V8G2|G3V8G2_RAT	56 kDa	2.0308	3.1317	1.9411	2.0042	2.8586	4.0839	0.438462865	0.793991081
Dihydrolipoamide S-succinyltransferase (E2 component of 2-oxo-glutarate complex), isoform CRA_a Dlst	tr|G3V6P2|G3V6P2_RAT	49 kDa	9.1386	11.483	11.646	14.029	13.34	13.273	0.029864327	0.793947148
RAB10, member RAS oncogene family Rab10	tr|Q5RKJ9|Q5RKJ9_RAT	23 kDa	5.077	4.1756	4.8526	6.0125	6.67	5.1048	0.080920115	0.792992753
NSFL1 cofactor p47 Nsfl1c	sp|O35987|NSF1C_RAT	41 kDa	8.1232	6.2634	6.7937	11.023	8.5757	7.1467	0.21485536	0.791923097
14-3-3 protein gamma Ywhag	sp|P61983|1433G_RAT	28 kDa	23.354	17.746	23.293	27.056	24.774	29.608	0.070931621	0.790699673
LRRGT00192 RGD1304704	tr|Q6QI16|Q6QI16_RAT	34 kDa	5.077	6.2634	5.8232	8.0167	7.6229	6.1258	0.08455997	0.788572689
Cytochrome c oxidase subunit 5B, mitochondrial Cox5b	sp|P12075|COX5B_RAT	14 kDa	10.154	8.3513	9.7053	13.027	9.5286	13.273	0.127801971	0.787376565
Neural cell adhesion molecule L1 L1cam	tr|D3ZPC4|D3ZPC4_RAT (+2)	140 kDa	1	1	1.9411	3.0063	1	1	0.655856583	0.787228093
Epsin-2 Epn2	tr|F1LQ45|F1LQ45_RAT	62 kDa	2.0308	2.0878	2.9116	5.0104	1.9057	2.0419	0.574375463	0.784795713
NADH dehydrogenase [ubiquinone] flavoprotein 2, mitochondrial Ndufv2	sp|P19234|NDUV2_RAT	27 kDa	8.1232	9.3952	9.7053	12.025	11.434	11.231	0.009929282	0.784770827
Glyceraldehyde-3-phosphate dehydrogenase Gapdh	sp|P04797|G3P_RAT	36 kDa	74.124	64.722	80.554	85.177	101.96	92.908	0.038966418	0.783445518
Protein LOC100361891 LOC100361891	tr|M0RC99|M0RC99_RAT	24 kDa	5.077	4.1756	3.8821	6.0125	6.67	4.0839	0.229862713	0.783394169
ATP synthase subunit g, mitochondrial Atp5l	sp|Q6PDU7|ATP5L_RAT	11 kDa	6.0924	5.2195	5.8232	9.0188	5.7171	7.1467	0.185246705	0.783046804
Protein LOC100909464 LOC100909464	tr|F1MAA3|F1MAA3_RAT	69 kDa	1	1	1.9411	2.0042	1	2.0419	0.471274312	0.781019005
Chaperonin containing Tcp1, subunit 6A (Zeta 1) Cct6a	tr|Q3MHS9|Q3MHS9_RAT	58 kDa	17.262	13.571	16.499	22.046	17.151	21.44	0.080703632	0.780579514
Glutamate dehydrogenase 1, mitochondrial Glud1	sp|P10860|DHE3_RAT	61 kDa	54.832	48.02	49.497	63.131	65.747	66.363	0.003375577	0.780312537
Proteasome subunit alpha type-6 Psma6	sp|P60901|PSA6_RAT	27 kDa	5.077	6.2634	4.8526	7.0146	7.6229	6.1258	0.068975626	0.779885664
Septin-5 Sept5	sp|Q9JJM9|SEPT5_RAT (+1)	43 kDa	12.185	10.439	10.676	16.033	12.387	14.293	0.057297506	0.779622129
Septin 8 (Predicted) Sept8	tr|G3V9Z6|G3V9Z6_RAT	50 kDa	18.277	15.659	15.528	22.046	20.01	21.44	0.012331612	0.779009701
Aspartate aminotransferase, cytoplasmic Got1	sp|P13221|AATC_RAT	46 kDa	20.308	17.746	21.352	27.056	25.727	23.482	0.019797519	0.778941848
Cullin-3 Cul3	sp|B5DF89|CUL3_RAT	89 kDa	1	1	1.9411	1	1	3.0629	0.646721651	0.778427384
Uncharacterized protein (Fragment)	tr|D3ZC00|D3ZC00_RAT	46 kDa	1	1	1.9411	1	1	3.0629	0.646721651	0.778427384
Mitogen-activated protein kinase 1 Mapk1	sp|P63086|MK01_RAT	41 kDa	8.1232	9.3952	8.7347	11.023	10.481	12.252	0.017411251	0.777731366
Aminoacylase-1A Acy1a	sp|Q6AYS7|ACY1A_RAT	46 kDa	8.1232	9.3952	7.7642	11.023	12.387	9.1887	0.080992808	0.775570805
60 kDa heat shock protein, mitochondrial Hspd1	sp|P63039|CH60_RAT	61 kDa	50.77	48.02	46.585	59.123	60.983	67.384	0.007297039	0.775374687
Tubulin beta-5 chain Tubb5	sp|P69897|TBB5_RAT	50 kDa	113.73	92.908	107.73	137.29	133.4	134.77	0.00848708	0.775336655
Calcium/calmodulin-dependent protein kinase type II subunit gamma Camk2g	sp|P11730|KCC2G_RAT	59 kDa	15.231	13.571	12.617	18.038	17.151	18.377	0.008761205	0.773233021
Reticulon-4 Rtn4	sp|Q9JK11|RTN4_RAT (+1)	126 kDa	17.262	12.527	21.352	20.042	24.774	21.44	0.158493864	0.771869717
Paralemmin-1 Palm	sp|Q920Q0|PALM_RAT	42 kDa	7.1078	6.2634	5.8232	8.0167	6.67	10.21	0.15856316	0.770961613
Rab GTPase-binding effector protein 1 Rabep1	sp|O35550|RABE1_RAT	99 kDa	3.0462	3.1317	2.9116	5.0104	4.7643	2.0419	0.394300173	0.769214495
Protein Stxbp3 Stxbp3	tr|Q99PV2|Q99PV2_RAT	68 kDa	4.0616	6.2634	1.9411	6.0125	3.8114	6.1258	0.446980757	0.769048948
Sodium/potassium-transporting ATPase subunit beta-1 Atp1b1	sp|P07340|AT1B1_RAT	35 kDa	12.185	11.483	9.7053	14.029	17.151	12.252	0.105848538	0.768403481
Protein lin-7 homolog A Lin7a	sp|Q9Z250|LIN7A_RAT (+1)	26 kDa	1	1	1	1	1.9057	1	0.373900966	0.76810815
Tyrosine-protein kinase Fyn Fyn	sp|Q62844|FYN_RAT	61 kDa	1	1	1	1	1.9057	1	0.373900966	0.76810815
Protein Uap1 Uap1	tr|D3ZF39|D3ZF39_RAT	58 kDa	1	1	1	1	1.9057	1	0.373900966	0.76810815
Platelet-activating factor acetylhydrolase IB subunit gamma Pafah1b3	sp|O35263|PA1B3_RAT	26 kDa	1	1	1	1	1.9057	1	0.373900966	0.76810815
UPF0696 protein C11orf68 homolog Bles03	sp|Q566Q8|CK068_RAT	27 kDa	1	1	1	1	1.9057	1	0.373900966	0.76810815
Protein Mdp1 Mdp1	tr|D4A4U3|D4A4U3_RAT	19 kDa	1	1	1	1	1.9057	1	0.373900966	0.76810815
Mitochondrial pyruvate carrier 1 Mpc1	sp|P63031|MPC1_RAT	12 kDa	1	1	1	1	1.9057	1	0.373900966	0.76810815
Cullin-5 Cul5	sp|Q9JJ31|CUL5_RAT	91 kDa	1	1	1	1	1.9057	1	0.373900966	0.76810815
26S proteasome non-ATPase regulatory subunit 1 Psmd1	sp|O88761|PSMD1_RAT (+1)	106 kDa	1	1	1	1	1.9057	1	0.373900966	0.76810815
MOB-like protein phocein Mob4	sp|Q9QYW3|PHOCN_RAT	26 kDa	1	1	1	1	1.9057	1	0.373900966	0.76810815
Unconventional myosin-Ie Myo1e	sp|Q63356|MYO1E_RAT (+1)	127 kDa	1	1	1	1	1.9057	1	0.373900966	0.76810815
Suppressor of G2 allele of SKP1 homolog Sugt1	sp|B0BN85|SUGT1_RAT	38 kDa	1	1	1	1	1.9057	1	0.373900966	0.76810815
Protein LOC679974 LOC679974	tr|M0RBL8|M0RBL8_RAT (+1)	22 kDa	1	1	1	1	1.9057	1	0.373900966	0.76810815
Abl interactor 1 Abi1	sp|Q9QZM5|ABI1_RAT	52 kDa	1	1	1	1	1.9057	1	0.373900966	0.76810815
Protein Rtn4ip1 Rtn4ip1	tr|B2GUZ6|B2GUZ6_RAT	44 kDa	1	1	1	1	1.9057	1	0.373900966	0.76810815
Muscleblind-like protein 2 Mbnl2	sp|F2Z3T4|MBNL2_RAT (+2)	40 kDa	1	1	1	1	1.9057	1	0.373900966	0.76810815
Protein Txndc17 Txndc17	tr|B0K010|B0K010_RAT	14 kDa	1	1	1	1	1.9057	1	0.373900966	0.76810815
Isoform 2 of Brain-specific angiogenesis inhibitor 1-associated protein 2 Baiap2	sp|Q6GMN2-2|BAIP2_RAT (+3)	58 kDa	1	1	1	1	1.9057	1	0.373900966	0.76810815
Tubulin-specific chaperone E Tbce	sp|Q5FVQ9|TBCE_RAT	59 kDa	1	1	1	1	1.9057	1	0.373900966	0.76810815
Phospholipase A2, group VII (Platelet-activating factor acetylhydrolase, plasma) Pla2g7	tr|Q5M7T7|Q5M7T7_RAT	49 kDa	1	1	1	1	1.9057	1	0.373900966	0.76810815

**Exocyst complex component 8 Exoc8**	sp|O54924|EXOC8_RAT	81 kDa	1	1	1	1	1.9057	1	0.373900966	0.76810815
**Isoform 2 of ERO1-like protein alpha Ero1l**	sp|Q8R4A1-2|ERO1A_RAT (+2)	42 kDa	1	1	1	1	1.9057	1	0.373900966	0.76810815
**NADH dehydrogenase [ubiquinone] 1 alpha subcomplex assembly factor 4 Ndufaf4**	sp|Q9NQR8|NDUF4_RAT	20 kDa	1	1	1	1	1.9057	1	0.373900966	0.76810815
**CD82 antigen Cd82**	sp|O70352|CD82_RAT (+1)	29 kDa	1	1	1	1	1.9057	1	0.373900966	0.76810815
**Coproporphyrinogen-III oxidase, mitochondrial Cpox**	sp|Q3B7D0|HEM6_RAT	49 kDa	1	1	1	1	1.9057	1	0.373900966	0.76810815
**Protein phosphatase methylesterase 1 Ppme1**	sp|Q4FZT2|PPME1_RAT	42 kDa	1	1	1	1	1.9057	1	0.373900966	0.76810815
**Isoform 2 of Homer protein homolog 1 Homer1**	sp|Q9Z214-2|HOME1_RAT (+1)	40 kDa	1	1	1	1	1.9057	1	0.373900966	0.76810815
**EF hand calcium binding protein 2 Necab2**	tr|F1LQY6|F1LQY6_RAT	39 kDa	1	1	1	1	1.9057	1	0.373900966	0.76810815
**Ly6/neurotoxin 1 (Predicted), isoform CRA_a Lynx1**	tr|D4A6F2|D4A6F2_RAT	13 kDa	1	1	1	1	1.9057	1	0.373900966	0.76810815
**Cytidine monophosphate N-acetylneuraminic acid synthetase Cmas**	tr|Q5M963|Q5M963_RAT	48 kDa	1	1	1	1	1.9057	1	0.373900966	0.76810815
**Engulfment and cell motility 1, ced-12 homolog (C. elegans) (Predicted), isoform CRA_a Elmo1**	tr|D3ZY46|D3ZY46_RAT (+1)	84 kDa	1	1	1	1	1.9057	1	0.373900966	0.76810815
**Ganglioside-induced differentiation-associated-protein 1 (Predicted) Gdap1**	tr|D4A5X7|D4A5X7_RAT	41 kDa	1	1	1	1	1.9057	1	0.373900966	0.76810815
**Beta-adrenergic receptor kinase 1 Adrbk1**	sp|P26817|ARBK1_RAT (+1)	80 kDa	1	1	1	1	1.9057	1	0.373900966	0.76810815
**Protein rogdi homolog Rogdi**	sp|Q4V7D2|ROGDI_RAT	32 kDa	1	1	1	1	1.9057	1	0.373900966	0.76810815
**Serine/threonine-protein kinase Nek7 Nek7**	sp|D3ZBE5|NEK7_RAT	35 kDa	1	1	1	1	1.9057	1	0.373900966	0.76810815
**Isoform 2 of Membrane-associated guanylate kinase, WW and PDZ domain-containing protein 1 Magi1**	sp|Q4L1J4-2|MAGI1_RAT (+3)	111 kDa	1	1	1	1	1.9057	1	0.373900966	0.76810815
**Protein NDRG3 Ndrg3**	sp|Q6AYR2|NDRG3_RAT	42 kDa	1	1	1	1	1.9057	1	0.373900966	0.76810815
**Protein Mylk Mylk**	tr|D3ZFU9|D3ZFU9_RAT	215 kDa	1	1	1	1	1.9057	1	0.373900966	0.76810815
**Isoform 2 of Arf-GAP with SH3 domain, ANK repeat and PH domain-containing protein 1 Asap1**	sp|Q1AAU6-2|ASAP1_RAT (+2)	121 kDa	1	1	1	1	1.9057	1	0.373900966	0.76810815
**Geranylgeranyl transferase type-2 subunit alpha Rabggta**	sp|Q08602|PGTA_RAT	65 kDa	1	1	1	1	1.9057	1	0.373900966	0.76810815
**Eukaryotic translation initiation factor 4 gamma, 3 (Predicted), isoform CRA_a Eif4g3**	tr|D4A554|D4A554_RAT	175 kDa	1	1	1	1	1.9057	1	0.373900966	0.76810815
**Ras-related protein Rab-31 Rab31**	sp|Q6GQP4|RAB31_RAT	21 kDa	1	1	1	1	1.9057	1	0.373900966	0.76810815
**Isoform Short of 14-3-3 protein beta/alpha Ywhab**	sp|P35213-2|1433B_RAT (+1)	28 kDa	18.277	11.483	17.469	19.04	20.01	22.461	0.115215231	0.767813887
**Protein Rab5b Rab5b**	tr|A1L1J8|A1L1J8_RAT	24 kDa	4.0616	5.2195	2.9116	6.0125	4.7643	5.1048	0.182485549	0.767724914
**Serine/threonine-protein kinase 24 (Fragment) Stk24**	tr|H9KVF3|H9KVF3_RAT (+1)	47 kDa	2.0308	2.0878	1.9411	3.0063	2.8586	2.0419	0.111888226	0.766390955
**Naca protein Naca**	tr|B2RYX0|B2RYX0_RAT	23 kDa	2.0308	2.0878	1.9411	3.0063	2.8586	2.0419	0.111888226	0.766390955
**Prostaglandin reductase 2 Ptgr2**	sp|Q5BK81|PTGR2_RAT	38 kDa	3.0462	1	1.9411	3.0063	3.8114	1	0.583334198	0.76586464
**Protein Ablim1 Ablim1**	tr|F1LWK7|F1LWK7_RAT	46 kDa	2.0308	4.1756	2.9116	3.0063	3.8114	5.1048	0.34424842	0.764772489
**Cytochrome c oxidase subunit 6B1 LOC681754**	tr|D3ZD09|D3ZD09_RAT	10 kDa	7.1078	6.2634	4.8526	8.0167	6.67	9.1887	0.127202135	0.763287735
**Malignant T-cell-amplified sequence 1 Mcts1**	sp|Q4G009|MCTS1_RAT	21 kDa	2.0308	5.2195	4.8526	7.0146	4.7643	4.0839	0.402981341	0.76297375
**Protein Tubb6 Tubb6**	tr|Q4QQV0|Q4QQV0_RAT	50 kDa	59.909	50.108	59.202	73.152	75.276	73.509	0.005500473	0.762464123
**Programmed cell death 6-interacting protein Pdcd6ip**	sp|Q9QZA2|PDC6I_RAT	97 kDa	10.154	12.527	9.7053	14.029	15.246	13.273	0.031838789	0.761170913
**ATP synthase subunit gamma, mitochondrial Atp5c1**	sp|P35435|ATPG_RAT (+1)	30 kDa	10.154	10.439	12.617	14.029	14.293	15.314	0.016262475	0.761068842
**Vacuolar protein sorting-associated protein 4A Vps4a**	sp|Q793F9|VPS4A_RAT	49 kDa	3.0462	3.1317	2.9116	5.0104	2.8586	4.0839	0.202277774	0.760443072
**Tubulin beta-2A chain Tubb2a**	sp|P85108|TBB2A_RAT	50 kDa	119.82	98.127	109.67	144.3	142.93	143.96	0.005337018	0.759797305
**Versican core protein (Fragment) Vcan**	tr|D3Z9N6|D3Z9N6_RAT	163 kDa	15.231	9.3952	14.558	15.031	17.151	19.398	0.137967249	0.75967817
**Serine-threonine kinase receptor-associated protein Strap**	sp|Q5XIG8|STRAP_RAT	38 kDa	4.0616	3.1317	4.8526	6.0125	4.7643	5.1048	0.108740044	0.758481513
**Astrocytic phosphoprotein PEA-15 Pea15**	sp|Q5U318|PEA15_RAT	15 kDa	9.1386	7.3073	10.676	13.027	10.481	12.252	0.0794913	0.758442394
**Tubulin beta-2B chain Tubb2b**	sp|Q3KRE8|TBB2B_RAT	50 kDa	118.8	98.127	109.67	143.3	143.88	143.96	0.004332884	0.757519599
**Neural cell adhesion molecule 1 (Fragment) Ncam1**	tr|F1LUV9|F1LUV9_RAT	92 kDa	16.246	13.571	23.293	21.044	27.633	21.44	0.190422316	0.757448265
**Protein Rtcd1 Rtcd1**	tr|Q68FS8|Q68FS8_RAT	39 kDa	3.0462	2.0878	3.8821	4.0083	3.8114	4.0839	0.140509187	0.757426325
**Isoform 2 of Dynactin subunit 4 Dctn4**	sp|Q9QUR2-2|DCTN4_RAT (+1)	52 kDa	5.077	2.0878	4.8526	6.0125	4.7643	5.1048	0.279597046	0.756686984
**Heat shock 70 kDa protein 4L Hspa4l**	tr|B4F772|B4F772_RAT	94 kDa	19.293	15.659	21.352	25.052	22.869	26.545	0.037584877	0.756103457
**Ras-related protein Rab-18 Rab18**	sp|Q5EB77|RAB18_RAT	23 kDa	1	2.0878	2.9116	1	2.8586	4.0839	0.571978172	0.755354108
**14-3-3 protein zeta/delta Ywhaz**	sp|P63102|1433Z_RAT	28 kDa	17.262	16.703	18.44	23.048	22.869	23.482	0.000478073	0.755126155
**Isoform 2 of Protein NDRG2 Ndrg2**	sp|Q8VBU2-2|NDRG2_RAT	39 kDa	10.154	10.439	14.558	15.031	15.246	16.335	0.061132821	0.754119111
**Acyl-CoA synthetase family member 2, mitochondrial Acsf2**	sp|Q499N5|ACSF2_RAT	68 kDa	5.077	5.2195	3.8821	5.0104	6.67	7.1467	0.115843388	0.753095272
**Protein Pabpc1l2b (Fragment) Pabpc1l2b**	tr|F1M8Y9|F1M8Y9_RAT	23 kDa	1	1	1	2.0042	1	1	0.373900966	0.749213326
**Mitochondrial glutamate carrier 2 Slc25a18**	sp|Q505J6|GHC2_RAT	34 kDa	1	1	1	2.0042	1	1	0.373900966	0.749213326
**Protein Ndufa3 Ndufa3**	tr|F1M8L6|F1M8L6_RAT (+1)	9 kDa	1	1	1	2.0042	1	1	0.373900966	0.749213326
**Protein Larp1 (Fragment) Larp1**	tr|F1M062|F1M062_RAT	109 kDa	1	1	1	2.0042	1	1	0.373900966	0.749213326
**40S ribosomal protein S25 Rps25**	sp|P62853|RS25_RAT (+1)	14 kDa	1	1	1	2.0042	1	1	0.373900966	0.749213326
**Protein LOC100366237 LOC100366237**	tr|M0R5N4|M0R5N4_RAT	15 kDa	1	1	1	2.0042	1	1	0.373900966	0.749213326
**Phosphofurin acidic cluster sorting protein 1 Pacs1**	sp|O88588|PACS1_RAT (+1)	105 kDa	1	1	1	2.0042	1	1	0.373900966	0.749213326
**Casein kinase 2, alpha prime polypeptide Csnk2a2**	tr|B4F7A9|B4F7A9_RAT	41 kDa	1	1	1	2.0042	1	1	0.373900966	0.749213326
**Protein Scrn3 Scrn3**	tr|G3V8F4|G3V8F4_RAT	34 kDa	1	1	1	2.0042	1	1	0.373900966	0.749213326
**Importin subunit alpha Kpna3**	tr|Q56R18|Q56R18_RAT	58 kDa	1	1	1	2.0042	1	1	0.373900966	0.749213326
**Trafficking protein particle complex subunit 3 Trappc3**	sp|Q5U1Z2|TPPC3_RAT	20 kDa	1	1	1	2.0042	1	1	0.373900966	0.749213326
**UPF0587 protein C1orf123 homolog**	sp|Q498R7|CA123_RAT	18 kDa	1	1	1	2.0042	1	1	0.373900966	0.749213326
**Coatomer subunit gamma-1 Copg1**	sp|Q4AEF8|COPG1_RAT	98 kDa	1	1	1	2.0042	1	1	0.373900966	0.749213326
**Alkyldihydroxyacetonephosphate synthase, peroxisomal Agps**	sp|Q9EQR2|ADAS_RAT	72 kDa	1	1	1	2.0042	1	1	0.373900966	0.749213326
**DnaJ (Hsp40) homolog, subfamily B, member 1 (Predicted), isoform CRA_a Dnajb1**	tr|D3ZUU5|D3ZUU5_RAT	42 kDa	1	1	1	2.0042	1	1	0.373900966	0.749213326
**Mitochondrial import inner membrane translocase subunit TIM44 Timm44**	sp|O35094|TIM44_RAT (+1)	51 kDa	1	1	1	2.0042	1	1	0.373900966	0.749213326
**Striatin Strn**	sp|P70483|STRN_RAT (+1)	86 kDa	1	1	1	2.0042	1	1	0.373900966	0.749213326
**Protein Chmp2b Chmp2b**	tr|F1M8B7|F1M8B7_RAT	24 kDa	1	1	1	2.0042	1	1	0.373900966	0.749213326
**Parvalbumin alpha Pvalb**	sp|P02625|PRVA_RAT	12 kDa	1	1	1	2.0042	1	1	0.373900966	0.749213326
**Guanine nucleotide-binding protein subunit alpha-11 Gna11**	sp|Q9JID2|GNA11_RAT (+1)	42 kDa	1	1	1	2.0042	1	1	0.373900966	0.749213326
**Protein Atxn2 (Fragment) Atxn2**	tr|F1LXI6|F1LXI6_RAT (+1)	108 kDa	1	1	1	2.0042	1	1	0.373900966	0.749213326
**Protein LOC100364487 Srm**	tr|Q99MI5|Q99MI5_RAT	34 kDa	1	1	1	2.0042	1	1	0.373900966	0.749213326
**NmrA-like family domain-containing protein 1 Nmral1**	tr|D3ZDB9|D3ZDB9_RAT	34 kDa	1	1	1	2.0042	1	1	0.373900966	0.749213326
**AP-1 complex subunit mu-1 Ap1m1**	sp|Q32Q06|AP1M1_RAT	49 kDa	1	1	1	2.0042	1	1	0.373900966	0.749213326
**Protein Psd3 (Fragment) Psd3**	tr|D3ZI98|D3ZI98_RAT (+6)	109 kDa	1	1	1	2.0042	1	1	0.373900966	0.749213326
**Receptor-type tyrosine-protein phosphatase-like N Ptprn**	sp|Q63259|PTPRN_RAT	106 kDa	1	1	1	2.0042	1	1	0.373900966	0.749213326
**Protein Hdac6 Hdac6**	tr|D3ZVD8|D3ZVD8_RAT	126 kDa	1	1	1	2.0042	1	1	0.373900966	0.749213326
**Mitochondrial-processing peptidase subunit beta Pmpcb**	sp|Q03346|MPPB_RAT	54 kDa	1	1	1	2.0042	1	1	0.373900966	0.749213326
**Protein Dbndd2 Dbndd2**	tr|Q331S7|Q331S7_RAT	17 kDa	1	1	1	2.0042	1	1	0.373900966	0.749213326
**Immunoglobulin-binding protein 1 Igbp1**	sp|O08836|IGBP1_RAT	39 kDa	1	1	1	2.0042	1	1	0.373900966	0.749213326
**Uncharacterized protein**	tr|D4AB12|D4AB12_RAT	35 kDa	1	1	1	2.0042	1	1	0.373900966	0.749213326
**Copper transport protein ATOX1 Atox1**	sp|Q9WUC4|ATOX1_RAT	7 kDa	1	1	1	2.0042	1	1	0.373900966	0.749213326
**Protein Brk1 Brk1**	tr|D3ZUP5|D3ZUP5_RAT	9 kDa	1	1	1	2.0042	1	1	0.373900966	0.749213326
**ATG4 autophagy related 4 homolog B (S. cerevisiae) Atg4b**	tr|Q4KM36|Q4KM36_RAT	25 kDa	1	1	1	2.0042	1	1	0.373900966	0.749213326
**Protein Dip2c Dip2c**	tr|D3ZZB0|D3ZZB0_RAT	177 kDa	1	1	1	2.0042	1	1	0.373900966	0.749213326
**Protein Mut Mut**	tr|D3ZKG1|D3ZKG1_RAT	83 kDa	1	1	1	2.0042	1	1	0.373900966	0.749213326
**Uncharacterized protein**	tr|D3ZET9|D3ZET9_RAT	126 kDa	1	1	1	2.0042	1	1	0.373900966	0.749213326
**Protein Tubb4a Tubb4**	tr|B4F7C2|B4F7C2_RAT	50 kDa	111.69	93.952	110.64	142.3	141.02	138.85	0.003764201	0.749181609
**Histidine triad nucleotide binding protein 2 (Predicted), isoform CRA_a Hint2**	tr|D4AB01|D4AB01_RAT	17 kDa	4.0616	3.1317	3.8821	5.0104	5.7171	4.0839	0.087071726	0.747761859
**S-formylglutathione hydrolase Esd**	sp|B0BNE5|ESTD_RAT	31 kDa	5.077	4.1756	4.8526	6.0125	5.7171	7.1467	0.036234833	0.747243898
**Tripartite motif protein 3, isoform CRA_a Trim3**	tr|G3V8D6|G3V8D6_RAT	81 kDa	7.1078	7.3073	4.8526	9.0188	7.6229	9.1887	0.078403917	0.745931151
**Protein Atp6v1a Atp6v1a**	tr|D4A133|D4A133_RAT	68 kDa	32.493	30.273	33.968	44.092	43.831	41.86	0.001014773	0.745351857
**NADH dehydrogenase [ubiquinone] 1 alpha subcomplex subunit 9, mitochondrial Ndufa9**	sp|Q5BK63|NDUA9_RAT	43 kDa	9.1386	8.3513	7.7642	10.021	8.5757	15.314	0.238866317	0.744723642
**Profilin-2 Pfn2**	sp|Q9EPC6|PROF2_RAT (+1)	15 kDa	7.1078	5.2195	6.7937	9.0188	9.5286	7.1467	0.078086419	0.744178625
**6-phosphofructokinase type C Pfkp**	sp|P47860|K6PP_RAT	86 kDa	15.231	16.703	26.204	27.056	27.633	23.482	0.143416137	0.743728493
**Carboxypeptidase E Cpe**	sp|P15087|CBPE_RAT	53 kDa	4.0616	2.0878	1.9411	4.0083	3.8114	3.0629	0.277953855	0.743434473
**Malic enzyme (Fragment) Me1**	tr|F1LQQ1|F1LQQ1_RAT	61 kDa	11.169	12.527	14.558	17.035	18.104	16.335	0.016587101	0.74317131
**ATP synthase subunit beta, mitochondrial Atp5b**	sp|P10719|ATPB_RAT (+1)	56 kDa	78.186	75.161	77.642	110.23	103.86	96.992	0.002461841	0.742534123
**Rho-associated protein kinase 2 Rock2**	sp|Q62868|ROCK2_RAT (+1)	160 kDa	1	1	1	1	1	2.0419	0.373900966	0.742225191
**Phenylalanyl-tRNA synthetase, beta subunit Farsb**	tr|Q68FT7|Q68FT7_RAT	66 kDa	1	1	1	1	1	2.0419	0.373900966	0.742225191
**Hspc159 protein Lgalsl**	tr|B4F7A3|B4F7A3_RAT	19 kDa	1	1	1	1	1	2.0419	0.373900966	0.742225191
**Protein Aplp1 (Fragment) Aplp1**	tr|F1LRS5|F1LRS5_RAT	68 kDa	1	1	1	1	1	2.0419	0.373900966	0.742225191
**Dynein light chain Tctex-type 3 Dynlt3**	tr|Q5XI90|Q5XI90_RAT	13 kDa	1	1	1	1	1	2.0419	0.373900966	0.742225191
**ADP-dependent glucokinase, isoform CRA_a Adpgk**	tr|G3V784|G3V784_RAT	54 kDa	1	1	1	1	1	2.0419	0.373900966	0.742225191
**5'-AMP-activated protein kinase catalytic subunit alpha-1 Prkaa1**	sp|P54645|AAPK1_RAT	64 kDa	1	1	1	1	1	2.0419	0.373900966	0.742225191
**SEC14-like protein 2 Sec14l2**	sp|Q99MS0|S14L2_RAT (+1)	46 kDa	1	1	1	1	1	2.0419	0.373900966	0.742225191
**Lgi2 protein Lgi2**	tr|B5DF14|B5DF14_RAT	62 kDa	1	1	1	1	1	2.0419	0.373900966	0.742225191
**Protein Diras2 Diras2**	tr|D3ZHX3|D3ZHX3_RAT	22 kDa	1	1	1	1	1	2.0419	0.373900966	0.742225191
**Glycogen [starch] synthase, muscle Gys1**	sp|A2RRU1|GYS1_RAT	84 kDa	1	1	1	1	1	2.0419	0.373900966	0.742225191
**Protein Syap1 Syap1**	tr|Q6AYB6|Q6AYB6_RAT	37 kDa	1	1	1	1	1	2.0419	0.373900966	0.742225191
**Protein Dip2a Dip2a**	tr|F1LZ43|F1LZ43_RAT	170 kDa	1	1	1	1	1	2.0419	0.373900966	0.742225191
**Junctional adhesion molecule C Jam3**	sp|Q68FQ2|JAM3_RAT	35 kDa	1	1	1	1	1	2.0419	0.373900966	0.742225191
**Pyrroline-5-carboxylate reductase Pycr1**	tr|D3ZXI0|D3ZXI0_RAT	32 kDa	1	1	1	1	1	2.0419	0.373900966	0.742225191
**Protein Srp68 Srp68**	tr|B2RYI2|B2RYI2_RAT	70 kDa	1	1	1	1	1	2.0419	0.373900966	0.742225191
**RGD1562230 protein Ctnna3**	tr|B2RYN9|B2RYN9_RAT	17 kDa	1	1	1	1	1	2.0419	0.373900966	0.742225191
**Protein bassoon Bsn**	tr|G3V984|G3V984_RAT	418 kDa	1	1	1	1	1	2.0419	0.373900966	0.742225191
**Huntingtin interacting protein 2 (Predicted), isoform CRA_a Ube2k**	tr|D3ZXS8|D3ZXS8_RAT	22 kDa	1	1	1	1	1	2.0419	0.373900966	0.742225191
**Uncharacterized protein (Fragment) LOC100364190**	tr|F1LTJ9|F1LTJ9_RAT	49 kDa	1	1	1	1	1	2.0419	0.373900966	0.742225191
**Guanine nucleotide-binding protein G(z) subunit alpha Gnaz**	sp|P19627|GNAZ_RAT	41 kDa	1	1	1	1	1	2.0419	0.373900966	0.742225191
**Tubulin gamma-1 chain Tubg1**	sp|P83888|TBG1_RAT	51 kDa	1	1	1	1	1	2.0419	0.373900966	0.742225191
**Protein Vps51 Vps51**	tr|F1M4I4|F1M4I4_RAT	86 kDa	1	1	1	1	1	2.0419	0.373900966	0.742225191
**Family with sequence similarity 126, member B Fam126b**	tr|Q4V7D4|Q4V7D4_RAT	51 kDa	1	1	1	1	1	2.0419	0.373900966	0.742225191
**Protein Armc6 Armc6**	tr|F7F1L0|F7F1L0_RAT	30 kDa	1	1	1	1	1	2.0419	0.373900966	0.742225191
**Protein Nacad Nacad**	tr|F1M6E5|F1M6E5_RAT	148 kDa	1	1	1	1	1	2.0419	0.373900966	0.742225191

**Isoform 2 of Thioredoxin reductase 2, mitochondrial Txnrd2**	sp|Q9Z0J5-2|TRXR2_RAT (+2)	53 kDa	1	1	1	1	1	2.0419	0.373900966	0.742225191
**Vacuolar protein sorting-associated protein 45 Vps45**	sp|O08700|VPS45_RAT	65 kDa	1	3.1317	1	4.0083	1.9057	1	0.62969473	0.742218687
**Ras-related protein Ral-A Rala**	sp|P63322|RALA_RAT	24 kDa	9.1386	6.2634	12.617	12.025	11.434	14.293	0.185771563	0.742185844
**60S acidic ribosomal protein P1 Rplp1**	sp|P19944|RLA1_RAT	11 kDa	4.0616	2.0878	1.9411	3.0063	3.8114	4.0839	0.283125182	0.742138769
**Endophilin-A1 Sh3gl2**	sp|O35179|SH3G2_RAT (+1)	40 kDa	10.154	8.3513	8.7347	13.027	11.434	12.252	0.011579304	0.741971509
**cAMP-dependent protein kinase type II-alpha regulatory subunit Prkar2a**	sp|P12368|KAP2_RAT (+1)	46 kDa	6.0924	7.3073	5.8232	10.021	5.7171	10.21	0.218337284	0.740821101
**Protein Sept4 Sept4**	tr|E9PST0|E9PST0_RAT	38 kDa	10.154	7.3073	9.7053	14.029	11.434	11.231	0.065577368	0.740355371
**OCIA domain-containing protein 1 Ociad1**	sp|Q5XIG4|OCAD1_RAT	28 kDa	2.0308	2.0878	1	4.0083	1.9057	1	0.566261232	0.74032398
**Myelin-oligodendrocyte glycoprotein Mog**	sp|Q63345|MOG_RAT (+1)	28 kDa	9.1386	6.2634	11.646	10.021	13.34	13.273	0.168270626	0.738330513
**Malate dehydrogenase, mitochondrial Mdh2**	sp|P04636|MDHM_RAT	36 kDa	24.37	24.01	26.204	35.073	33.35	32.671	0.000858237	0.737768809
**Actin-related protein 2/3 complex subunit 1A Arpc1a**	sp|Q99PD4|ARC1A_RAT	42 kDa	8.1232	2.0878	5.8232	8.0167	7.6229	6.1258	0.360237714	0.736682992
**Protein Stam Stam**	tr|B5DF55|B5DF55_RAT	60 kDa	2.0308	1	2.9116	4.0083	1	3.0629	0.534718099	0.736247398
**T-complex protein 1 subunit alpha Tcp1**	sp|P28480|TCPA_RAT	60 kDa	15.231	16.703	18.44	27.056	20.963	20.419	0.060398043	0.73605307
**Phosphatidylinositol transfer protein alpha isoform Pitpna**	sp|P16446|PIPNA_RAT	32 kDa	4.0616	4.1756	4.8526	4.0083	6.67	7.1467	0.192138275	0.734350631
**Ubiquitin thioesterase OTUB1 Otub1**	sp|B2RYG6|OTUB1_RAT	31 kDa	4.0616	4.1756	4.8526	6.0125	5.7171	6.1258	0.004477434	0.733100351
**Reticulon-1 Rtn1**	sp|Q64548|RTN1_RAT	83 kDa	2.0308	3.1317	8.7347	5.0104	4.7643	9.1887	0.540038013	0.732843267
**Amine oxidase [flavin-containing] A Maoa**	sp|P21396|AOFA_RAT (+1)	60 kDa	2.0308	2.0878	1	1	1.9057	4.0839	0.559516315	0.732316585
**40S ribosomal protein S17 Rps17**	sp|P04644|RS17_RAT (+1)	16 kDa	3.0462	3.1317	3.8821	4.0083	6.67	3.0629	0.331748225	0.732104911
**gi|125080|sp|P02533| K1CN_HUMAN KERATIN, TYPE I CYTOSKELETAL 14 (CYTOK**	zz|ZZ_FGCZCont0187|	52 kDa	7.1078	1	4.8526	12.025	4.7643	1	0.685537211	0.728550308
**Thioredoxin reductase 1, cytoplasmic Txnrd1**	sp|O89049|TRXR1_RAT	55 kDa	6.0924	4.1756	7.7642	8.0167	8.5757	8.1677	0.099575018	0.728276542
**Superoxide dismutase [Cu-Zn] Sod1**	sp|P07632|SODC_RAT	16 kDa	4.0616	6.2634	4.8526	10.021	5.7171	5.1048	0.322740811	0.728190415
**Programmed cell death 6 (Predicted), isoform CRA_a Pdcd6**	tr|G3V7W1|G3V7W1_RAT	22 kDa	4.0616	3.1317	2.9116	4.0083	4.7643	5.1048	0.058388367	0.72815513
**D-tyrosyl-tRNA(Tyr) deacylase Dtd1**	tr|B0K014|B0K014_RAT	23 kDa	2.0308	4.1756	3.8821	5.0104	4.7643	4.0839	0.158636654	0.727959534
**Ethylmalonic encephalopathy 1 Ethe1**	tr|B0BNJ4|B0BNJ4_RAT	28 kDa	2.0308	3.1317	1.9411	2.0042	5.7171	2.0419	0.529563389	0.727589315
**Triosephosphate isomerase Tpi1**	sp|P48500|TPIS_RAT	27 kDa	22.339	20.878	22.322	30.063	29.539	30.629	0.00014076	0.726346821
**Acyl-CoA-binding protein Dbi**	sp|P11030|ACBP_RAT (+1)	10 kDa	7.1078	6.2634	6.7937	11.023	8.5757	8.1677	0.051944601	0.726233865
**Pyruvate dehydrogenase E1 component subunit alpha, somatic form, mitochondrial Pdha1**	sp|P26284|ODPA_RAT	43 kDa	13.2	12.527	14.558	20.042	18.104	17.356	0.007085131	0.7258297
**Nucleosome assembly protein 1-like 4 Nap1l4**	sp|Q5U2Z3|NP1L4_RAT	44 kDa	5.077	4.1756	5.8232	6.0125	7.6229	7.1467	0.047838968	0.725422359
**Uncharacterized protein**	tr|M0R5B4|M0R5B4_RAT	39 kDa	39.601	40.712	44.644	54.113	55.266	63.3	0.00824638	0.723637501
**Ras-related protein Rab-8B Rab8b**	sp|P70550|RAB8B_RAT	24 kDa	4.0616	2.0878	1	3.0063	3.8114	3.0629	0.384364236	0.72357954
**Isoform 3 of SH3 domain-containing kinase-binding protein 1 Sh3kbp1**	sp|Q925Q9-3|SH3K1_RAT (+2)	47 kDa	1	2.0878	1.9411	3.0063	1.9057	2.0419	0.257163848	0.723176922
**DnaJ (Hsp40) homolog, subfamily A, member 4 Dnaja4**	tr|Q4QR73|Q4QR73_RAT	62 kDa	1	2.0878	1.9411	3.0063	1.9057	2.0419	0.257163848	0.723176922
**Transcription factor Pur-beta Purb**	tr|F1LSL1|F1LSL1_RAT	34 kDa	4.0616	3.1317	2.9116	6.0125	2.8586	5.1048	0.267058125	0.723023204
**Cullin-associated NEDD8-dissociated protein 1 Cand1**	sp|P97536|CAND1_RAT	136 kDa	9.1386	4.1756	14.558	9.0188	15.246	14.293	0.374777899	0.722868006
**Junction plakoglobin Jup**	sp|Q6P0K8|PLAK_RAT	82 kDa	3.0462	1	3.8821	6.0125	1.9057	3.0629	0.532798329	0.721995064
**V-type proton ATPase subunit B, brain isoform Atp6v1b2**	sp|P62815|VATB2_RAT	57 kDa	33.508	30.273	33.968	44.092	46.69	44.922	0.000813289	0.720310381
**Receptor expression-enhancing protein 5 Reep5**	sp|B2RZ37|REEP5_RAT	21 kDa	2.0308	1	1.9411	2.0042	2.8586	2.0419	0.20988407	0.720074732
**NAD-dependent protein deacetylase sirtuin-2 Sirt2**	sp|Q5RJQ4|SIR2_RAT	39 kDa	18.277	20.878	24.263	27.056	28.586	32.671	0.026240816	0.718104922
**Dihydrolipoyl dehydrogenase, mitochondrial Dld**	sp|Q6P6R2|DLDH_RAT	54 kDa	24.37	19.834	22.322	31.065	34.303	27.566	0.019911511	0.71584135
**Protein Map6d1 Map6d1**	tr|D3ZT16|D3ZT16_RAT	20 kDa	4.0616	3.1317	4.8526	6.0125	5.7171	5.1048	0.047486543	0.715552678
**Isoform 2 of Flotillin-2 Flot2**	sp|Q9Z2S9-2|FLOT2_RAT (+2)	47 kDa	2.0308	1	1.9411	2.0042	1.9057	3.0629	0.249807418	0.713042106
**Protein Fsd1 Fsd1**	tr|M0R7H8|M0R7H8_RAT	14 kDa	2.0308	1	1.9411	2.0042	1.9057	3.0629	0.249807418	0.713042106
**Glutathione S-transferase A6 Gsta6**	sp|Q6AXY0|GSTA6_RAT	26 kDa	3.0462	2.0878	1.9411	4.0083	2.8586	3.0629	0.127186948	0.712511833
**Isoform Cytoplasmic+peroxisomal of Peroxiredoxin-5, mitochondrial Prdx5**	sp|Q9R063-2|PRDX5_RAT (+2)	17 kDa	10.154	11.483	13.587	15.031	18.104	16.335	0.023850406	0.712027491
**Asparagine-linked glycosylation 2 homolog (Yeast, alpha-1,3-mannosyltransferase), isoform CRA_a Alg2**	tr|G3V6U3|G3V6U3_RAT	47 kDa	7.1078	2.0878	1.9411	4.0083	8.5757	3.0629	0.565699094	0.71175121
**6-phosphofructokinase Pfkm**	tr|Q52KS1|Q52KS1_RAT	85 kDa	20.308	16.703	21.352	29.061	28.586	24.503	0.017142157	0.710444309
**UV excision repair protein RAD23 homolog B Rad23b**	sp|Q4KMA2|RD23B_RAT	43 kDa	3.0462	5.2195	2.9116	6.0125	6.67	3.0629	0.318555847	0.70987717
**Calcium/calmodulin-dependent protein kinase type II subunit alpha Camk2a**	sp|P11275|KCC2A_RAT	54 kDa	8.1232	5.2195	7.7642	11.023	9.5286	9.1887	0.055207115	0.709707031
**Ndufa7 protein Ndufa7**	tr|A9UMV9|A9UMV9_RAT	13 kDa	4.0616	3.1317	1.9411	5.0104	3.8114	4.0839	0.152633291	0.707780283
**CLIP-associating protein 2 Clasp2**	sp|Q99JD4|CLAP2_RAT	141 kDa	5.077	5.2195	5.8232	8.0167	7.6229	7.1467	0.002830333	0.707429464
**Peptidyl-prolyl cis-trans isomerase FKBP4 Fkbp4**	sp|Q9QVC8|FKBP4_RAT	51 kDa	6.0924	5.2195	4.8526	8.0167	6.67	8.1677	0.020705867	0.707281749
**Myotrophin Mtpn**	sp|P62775|MTPN_RAT	13 kDa	3.0462	3.1317	2.9116	4.0083	4.7643	4.0839	0.007245054	0.706996461
**Isoform 2 of Gephyrin Gphn**	sp|Q03555-3|GEPH_RAT (+4)	84 kDa	9.1386	3.1317	8.7347	10.021	9.5286	10.21	0.208688991	0.705822659
**Protein-arginine deiminase type-2 Padi2**	sp|P20717|PADI2_RAT	75 kDa	12.185	12.527	13.587	18.038	20.963	15.314	0.033898464	0.705127497
**Protein Ptprd (Fragment) Ptprd**	tr|F1M678|F1M678_RAT (+3)	210 kDa	4.0616	2.0878	2.9116	4.0083	4.7643	4.0839	0.111203989	0.704779683
**Myosin-10 Myh10**	sp|Q9JLT0|MYH10_RAT	229 kDa	22.339	29.229	23.293	35.073	32.397	38.797	0.021190084	0.704461404
**Tubulin alpha-1C chain Tuba1c**	sp|Q6AYZ1|TBA1C_RAT	50 kDa	63.97	67.854	77.642	102.21	98.144	96.992	0.002565535	0.704452052
**Monoacylglycerol lipase ABHD12 Abhd12**	sp|Q6AYT7|ABD12_RAT	45 kDa	1	1	2.9116	2.0042	1.9057	3.0629	0.404005175	0.704394218
**NADP-dependent malic enzyme Me1**	sp|P13697|MAOX_RAT	64 kDa	10.154	11.483	14.558	16.033	19.057	16.335	0.035084138	0.703840544
**Glutaminyl-tRNA synthetase Qars**	tr|Q66H61|Q66H61_RAT	88 kDa	2.0308	3.1317	3.8821	4.0083	4.7643	4.0839	0.097148315	0.703504064
**Tubulin alpha-8 chain Tuba8**	sp|Q6AY56|TBA8_RAT	50 kDa	41.631	39.668	47.556	60.125	60.983	62.279	0.001767202	0.702639773
**NADH dehydrogenase (Ubiquinone) Fe-S protein 3 (Predicted), isoform CRA_c Ndufs3**	tr|D3ZG43|D3ZG43_RAT	30 kDa	9.1386	8.3513	6.7937	13.027	12.387	9.1887	0.066350293	0.701783387
**Protein LOC100912618 LOC100912618**	tr|D3ZFY8|D3ZFY8_RAT	16 kDa	3.0462	2.0878	3.8821	4.0083	4.7643	4.0839	0.088588083	0.701287287
**Ubiquitin-conjugating enzyme E2 variant 2 Ube2v2**	sp|Q7M767|UB2V2_RAT	16 kDa	3.0462	2.0878	3.8821	4.0083	4.7643	4.0839	0.088588083	0.701287287
**Epsin-1 Epn1**	sp|O88339|EPN1_RAT	60 kDa	3.0462	3.1317	2.9116	5.0104	2.8586	5.1048	0.153488799	0.700604295
**Histidine triad nucleotide-binding protein 1 Hint1**	sp|P62959|HINT1_RAT	14 kDa	7.1078	4.1756	6.7937	10.021	7.6229	8.1677	0.094091424	0.700347906
**Chaperonin subunit 8 (Theta) (Predicted), isoform CRA_a Cct8**	tr|D4ACB8|D4ACB8_RAT	60 kDa	9.1386	8.3513	9.7053	12.025	10.481	16.335	0.096617812	0.700167349
**Microtubule-associated protein 1B Map1b**	tr|F1LRL9|F1LRL9_RAT	270 kDa	56.863	45.932	59.202	80.167	69.559	81.677	0.014402064	0.70006439
**Histidine triad nucleotide-binding protein 3 Hint3**	tr|F1M9G8|F1M9G8_RAT (+1)	20 kDa	5.077	1	2.9116	4.0083	4.7643	4.0839	0.343830446	0.699148291
**Isoform Cytoplasmic of Fumarate hydratase, mitochondrial Fh**	sp|P14408-2|FUMH_RAT	50 kDa	11.169	9.3952	10.676	16.033	13.34	15.314	0.009632265	0.699089221
**Ras-related protein Rab-3B Rab3b**	sp|Q63941|RAB3B_RAT	25 kDa	1	1	4.8526	5.0104	3.8114	1	0.601860507	0.697692887
**Bis(5'-nucleosyl)-tetraphosphatase [asymmetrical] Nudt2**	sp|Q6PEC0|AP4A_RAT	17 kDa	2.0308	2.0878	4.8526	3.0063	4.7643	5.1048	0.31576179	0.696770586
**4-nitrophenylphosphatase domain and non-neuronal SNAP25-like protein homolog 1 (C. elegans), isoform CRA_b Nipsnap1**	tr|G3V728|G3V728_RAT	33 kDa	7.1078	6.2634	8.7347	10.021	10.481	11.231	0.016409225	0.696621813
**Cortactin Cttn**	tr|Q66HL2|Q66HL2_RAT	57 kDa	3.0462	4.1756	3.8821	6.0125	3.8114	6.1258	0.122107874	0.696182373
**Protein LOC100909944 (Fragment) LOC100909944**	tr|F1M9J9|F1M9J9_RAT	34 kDa	5.077	1	1	1	1	8.1677	0.726828476	0.696027617
**Dynein light chain 2, cytoplasmic Dynll2**	sp|Q78P75|DYL2_RAT	10 kDa	4.0616	4.1756	4.8526	6.0125	6.67	6.1258	0.003967378	0.695958699
**Phosphoglycerate mutase 1 Pgam1**	sp|P25113|PGAM1_RAT	29 kDa	19.293	19.834	22.322	31.065	26.68	30.629	0.005888612	0.695328943
**Growth factor receptor-bound protein 2 Grb2**	sp|P62994|GRB2_RAT	25 kDa	4.0616	3.1317	3.8821	7.0146	3.8114	5.1048	0.171566327	0.695219324
**Ena/VASP-like protein (Fragment) Evl**	tr|F1M8I7|F1M8I7_RAT	42 kDa	2.0308	1	1	1	3.8114	1	0.584087715	0.69360223
**Phosphatidylinositol 5-phosphate 4-kinase type-2 beta Pip4k2b**	sp|O88377|PI42B_RAT	47 kDa	5.077	2.0878	3.8821	3.0063	4.7643	8.1677	0.403347036	0.693104032
**Phosphoribosyl pyrophosphate synthase-associated protein 2 Prpsap2**	sp|O08618|KPRB_RAT	41 kDa	3.0462	2.0878	1	3.0063	3.8114	2.0419	0.309735958	0.692356314
**Neurofascin Nfasc**	tr|D3ZNW5|D3ZNW5_RAT	132 kDa	18.277	14.615	21.352	26.054	24.774	27.566	0.018792816	0.69194071
**Protein Trim36 Trim36**	tr|F1LY63|F1LY63_RAT	83 kDa	1	2.0878	1	3.0063	1.9057	1	0.424293686	0.691441137
**Alpha-centractin Actr1a**	sp|P85515|ACTZ_RAT	43 kDa	10.154	8.3513	8.7347	13.027	16.199	10.21	0.088599768	0.690739426
**L-lactate dehydrogenase B chain Ldhb**	sp|P42123|LDHB_RAT	37 kDa	29.447	19.834	33.968	36.075	46.69	37.776	0.079316364	0.690628085
**Alpha-soluble NSF attachment protein Napa**	sp|P54921|SNAA_RAT	33 kDa	11.169	14.615	14.558	23.048	18.104	17.356	0.045991583	0.689512545
**Protein Sh3bgrl3 Sh3bgrl3**	tr|B2RZ27|B2RZ27_RAT	10 kDa	2.0308	1	1	2.0042	2.8586	1	0.392430303	0.687521321
**Aconitate hydratase, mitochondrial Aco2**	sp|Q9ER34|ACON_RAT	85 kDa	47.724	37.581	47.556	68.142	62.889	62.279	0.006280287	0.687295018
**Ras-related protein Ral-B Ralb**	sp|P36860|RALB_RAT	23 kDa	6.0924	4.1756	6.7937	8.0167	7.6229	9.1887	0.047063302	0.687187604
**NEDD8-activating enzyme E1 catalytic subunit Uba3**	sp|Q99MI7|UBA3_RAT	52 kDa	1	2.0878	1	2.0042	1.9057	2.0419	0.163795943	0.686817433
**Carbonic anhydrase 2 Ca2**	sp|P27139|CAH2_RAT	29 kDa	5.077	3.1317	6.7937	7.0146	6.67	8.1677	0.118216997	0.686536429
**Intersectin-1 Itsn1**	tr|F1M823|F1M823_RAT (+1)	137 kDa	3.0462	2.0878	1	2.0042	2.8586	4.0839	0.329285734	0.685615925
**Eukaryotic translation initiation factor 4B Eif4b**	tr|Q5RKG9|Q5RKG9_RAT	69 kDa	4.0616	4.1756	1.9411	6.0125	4.7643	4.0839	0.16510978	0.6849139
**LOC685144 protein Sec24c**	tr|B5DEG8|B5DEG8_RAT	119 kDa	1	2.0878	1	1	1.9057	3.0629	0.42015325	0.684884227
**Cytochrome c oxidase subunit 5A, mitochondrial Cox5a**	sp|P11240|COX5A_RAT	16 kDa	9.1386	7.3073	10.676	15.031	12.387	12.252	0.034613308	0.683687925
**Rab GDP dissociation inhibitor alpha Gdi1**	sp|P50398|GDIA_RAT	51 kDa	28.431	24.01	26.204	37.077	37.161	40.839	0.002411271	0.683411976
**ATPase, H+ transporting, V1 subunit E isoform 1, isoform CRA_a Atp6v1e1**	tr|G3V7L8|G3V7L8_RAT	26 kDa	8.1232	12.527	12.617	17.035	13.34	18.377	0.071059438	0.682376108
**Tubulin beta-3 chain Tubb3**	sp|Q4QRB4|TBB3_RAT	50 kDa	91.386	72.03	87.347	121.25	121.97	124.56	0.002853485	0.681828811
**Uncharacterized protein (Fragment)**	tr|F1LUV3|F1LUV3_RAT	37 kDa	4.0616	1	3.8821	6.0125	1	6.1258	0.514958014	0.68073495
**N(G),N(G)-dimethylarginine dimethylaminohydrolase 1 Ddah1**	sp|O08557|DDAH1_RAT	31 kDa	7.1078	5.2195	5.8232	7.0146	10.481	9.1887	0.069403293	0.680193972
**Synaptophysin Syp**	sp|P07825|SYPH_RAT	33 kDa	2.0308	2.0878	1.9411	4.0083	2.8586	2.0419	0.172161084	0.680192619
**6-phosphogluconate dehydrogenase, decarboxylating Pgd**	sp|P85968|6PGD_RAT	53 kDa	4.0616	6.2634	5.8232	7.0146	8.5757	8.1677	0.036339661	0.679695261
**Cell adhesion molecule 4 Cadm4**	sp|Q1WIM1|CADM4_RAT (+1)	43 kDa	7.1078	5.2195	5.8232	9.0188	9.5286	8.1677	0.013975031	0.679409772
**Protein Tpd52 Tpd52**	tr|F1MAB9|F1MAB9_RAT	24 kDa	3.0462	3.1317	3.8821	5.0104	5.7171	4.0839	0.043229198	0.679206557
**Up-regulated during skeletal muscle growth protein 5 Usmg5**	sp|Q9JJW3|USMG5_RAT (+2)	6 kDa	2.0308	3.1317	2.9116	4.0083	3.8114	4.0839	0.021032885	0.678290601
**Glycine cleavage system H protein, mitochondrial Gcsh**	sp|Q5I0P2|GCSH_RAT	18 kDa	2.0308	1	1	2.0042	1.9057	2.0419	0.137873689	0.677240499
**Uncharacterized protein**	tr|M0R5H9|M0R5H9_RAT	34 kDa	2.0308	1	1	2.0042	1.9057	2.0419	0.137873689	0.677240499
**Dynactin 1, isoform CRA_a Dctn1**	tr|D4A8U7|D4A8U7_RAT	142 kDa	15.231	16.703	22.322	27.056	28.586	24.503	0.024941184	0.676972986
**ADP-ribosylation factor-like protein 3 Arl3**	sp|P37996|ARL3_RAT (+1)	20 kDa	3.0462	2.0878	2.9116	5.0104	3.8114	3.0629	0.116773856	0.676971232
**ATP synthase subunit d, mitochondrial Atp5h**	sp|P31399|ATP5H_RAT	19 kDa	10.154	10.439	15.528	18.038	20.01	15.314	0.060305981	0.676904914
**COP9 signalosome complex subunit 8 Cops8**	sp|Q6P4Z9|CSN8_RAT	23 kDa	4.0616	2.0878	1.9411	5.0104	2.8586	4.0839	0.236399242	0.676865029
**NADH dehydrogenase (Ubiquinone) flavoprotein 1 Ndufv1**	tr|Q5XIH3|Q5XIH3_RAT	51 kDa	16.246	8.3513	13.587	14.029	20.963	21.44	0.142117761	0.676642685
**Uncharacterized protein Tnr**	tr|F1LQ63|F1LQ63_RAT	139 kDa	29.447	16.703	28.145	38.079	38.114	33.692	0.051123952	0.676115939
**Isoform A of Kinesin light chain 1 Klc1**	sp|P37285-2|KLC1_RAT (+3)	62 kDa	6.0924	9.3952	10.676	12.025	12.387	14.293	0.052893966	0.67597468
**AP-2 complex subunit mu Ap2m1**	sp|P84092|AP2M1_RAT	50 kDa	4.0616	5.2195	6.7937	9.0188	7.6229	7.1467	0.057064815	0.675741118
**Clathrin light chain A Clta**	sp|P08081|CLCA_RAT	27 kDa	3.0462	3.1317	3.8821	4.0083	4.7643	6.1258	0.074947422	0.675240294
**Glutaredoxin-3 Glrx3**	sp|Q9JLZ1|GLRX3_RAT	38 kDa	5.077	3.1317	3.8821	6.0125	4.7643	7.1467	0.094568568	0.674578068
**AP-1 complex subunit beta-1 Ap1b1**	tr|G3V9N8|G3V9N8_RAT	104 kDa	7.1078	5.2195	7.7642	10.021	8.5757	11.231	0.039971149	0.673585292
**Haloacid dehalogenase-like hydrolase domain-containing protein 2 Hdhd2**	tr|Q6QI86|Q6QI86_RAT	42 kDa	2.0308	2.0878	3.8821	4.0083	3.8114	4.0839	0.101169324	0.672124399
**Adenylate kinase isoenzyme 1 Ak1**	sp|P39069|KAD1_RAT	22 kDa	9.1386	10.439	11.646	14.029	17.151	15.314	0.011789597	0.671561922
**Isoform 2 of Syntaxin-binding protein 1 Stxbp1**	sp|P61765-2|STXB1_RAT	69 kDa	30.462	27.142	31.057	40.083	41.926	50.027	0.011717061	0.671491108

**Isoform 3 of Receptor-type tyrosine-protein phosphatase zeta Ptprz1**	sp|Q62656-3|PTPRZ_RAT (+1)	176 kDa	12.185	8.3513	12.617	14.029	18.104	17.356	0.041955066	0.669912506
**Obg-like ATPase 1 Ola1**	sp|A0JPJ7|OLA1_RAT	45 kDa	7.1078	8.3513	5.8232	11.023	9.5286	11.231	0.018071796	0.669621113
**Protein Drg2 Drg2**	tr|D3ZDC1|D3ZDC1_RAT	41 kDa	1	1	1.9411	1	2.8586	2.0419	0.353387548	0.667926447
**Isoform 2 of Cell adhesion molecule 2 Cadm2**	sp|Q1WIM2-2|CADM2_RAT (+1)	44 kDa	1	1	1.9411	1	2.8586	2.0419	0.353387548	0.667926447
**Transcriptional activator protein Pur-alpha Pura**	tr|F1LPS8|F1LPS8_RAT	34 kDa	7.1078	6.2634	5.8232	10.021	9.5286	9.1887	0.002071953	0.667903112
**Myelin protein P0 Mpz**	sp|P06907|MYP0_RAT	28 kDa	7.1078	7.3073	6.7937	12.025	9.5286	10.21	0.009805805	0.667707691
**Ras-related protein Rab-7a Rab7a**	sp|P09527|RAB7A_RAT	24 kDa	7.1078	5.2195	6.7937	11.023	10.481	7.1467	0.077566166	0.667383345
**Protein Pcbp4 Pcbp4**	tr|D3ZCS3|D3ZCS3_RAT	41 kDa	1	1	1.9411	3.0063	1.9057	1	0.375533185	0.666627199
**Dipeptidyl peptidase 3 Dpp3**	sp|O55096|DPP3_RAT	83 kDa	1	2.0878	4.8526	3.0063	3.8114	5.1048	0.364779624	0.666001258
**Hydroxyacyl glutathione hydrolase Hagh**	tr|F1LQI1|F1LQI1_RAT	34 kDa	5.077	5.2195	4.8526	8.0167	7.6229	7.1467	0.000738796	0.664833694
**Stress-induced-phosphoprotein 1 Stip1**	sp|O35814|STIP1_RAT	63 kDa	13.2	14.615	17.469	21.044	26.68	20.419	0.031758961	0.66454368
**Tricarboxylate transport protein, mitochondrial Slc25a1**	sp|P32089|TXTP_RAT	34 kDa	3.0462	4.1756	3.8821	5.0104	7.6229	4.0839	0.167813401	0.664220085
**Platelet-activating factor acetylhydrolase IB subunit beta Pafah1b2**	sp|O35264|PA1B2_RAT	26 kDa	7.1078	3.1317	4.8526	9.0188	7.6229	6.1258	0.146452269	0.662879104
**Cysteinyl-tRNA synthetase (Predicted), isoform CRA_b Cars**	tr|G3V9K0|G3V9K0_RAT	86 kDa	1	1	1.9411	2.0042	1.9057	2.0419	0.101471334	0.662169428
**BAG family molecular chaperone regulator 5 Bag5**	sp|Q5QJC9|BAG5_RAT	51 kDa	4.0616	3.1317	1.9411	4.4083	5.7171	4.0839	0.133339421	0.661467272
**Isoform 2 of Long-chain-fatty-acid--CoA ligase 6 Acsl6**	sp|P33124-2|ACSL6_RAT (+1)	81 kDa	4.0616	1	6.7937	5.0104	4.7643	8.1677	0.36776431	0.660742153
**Superoxide dismutase [Mn], mitochondrial Sod2**	sp|P07895|SODM_RAT	25 kDa	9.1386	8.3513	10.676	16.033	13.34	13.273	0.013201897	0.660458191
**Arginine--tRNA ligase, cytoplasmic Rars**	sp|P40329|SYRC_RAT	76 kDa	1	1	1.9411	1	1.9057	3.0629	0.372979578	0.660305599
**Ubiquitin carboxyl-terminal hydrolase Usp9x**	tr|D3ZC84|D3ZC84_RAT	289 kDa	1	1	1.9411	1	1.9057	3.0629	0.372979578	0.660305599
**Calcyclin-binding protein Cacybp**	sp|Q6AYK6|CYBP_RAT	27 kDa	4.0616	4.1756	4.8526	9.0188	5.7171	5.1048	0.143836191	0.659744868
**N-acetylneuraminic acid synthase Nans**	tr|B1WC26|B1WC26_RAT	40 kDa	4.0616	3.1317	3.8821	7.0146	5.7171	4.0839	0.09917081	0.658638407
**Ermin Ermn**	sp|Q5RJL0|ERMIN_RAT	32 kDa	10.154	9.3952	12.617	17.035	11.434	20.419	0.116842171	0.657956963
**Isoform 1 of Neuroplastin Nptn**	sp|P97546-1|NPTN_RAT (+3)	31 kDa	5.077	3.1317	2.9116	6.0125	4.7643	6.1258	0.077162202	0.657904701
**Heat shock 70kDa protein 12A (Predicted), isoform CRA_a Hspa12a**	tr|D3ZC55|D3ZC55_RAT	75 kDa	16.246	12.527	19.411	25.052	24.774	23.482	0.01496895	0.657281606
**STE20/SPS1-related proline-alanine-rich protein kinase Stk39**	sp|O88506|STK39_RAT	60 kDa	4.0616	3.1317	3.8821	5.0104	5.7171	6.1258	0.01124044	0.657165066
**Protein Rhog Rhog**	tr|Q32PX6|Q32PX6_RAT	21 kDa	9.1386	4.1756	8.7347	10.021	13.34	10.21	0.116214154	0.656784129
**Erythrocyte protein band 4.1-like 3, isoform CRA_d Epb41l3**	tr|Q9JMB3|Q9JMB3_RAT	122 kDa	29.447	17.746	30.086	43.09	39.067	35.734	0.040665556	0.655512295
**Tropomodulin-2 Tmod2**	sp|P70566|TMOD2_RAT	39 kDa	7.1078	9.3952	8.7347	12.025	14.293	12.252	0.010951035	0.654334975
**Protein-L-isoaspartate(D-aspartate) O-methyltransferase Pcmt1**	sp|P22062|PIMT_RAT	25 kDa	7.1078	4.1756	8.7347	11.023	11.434	8.1677	0.103653229	0.653658648
**Ubiquitin-conjugating enzyme E2 N Ube2n**	sp|Q9EQX9|UBE2N_RAT	17 kDa	6.0924	6.2634	5.8232	12.025	7.6229	8.1677	0.082162584	0.653554121
**Serine/threonine-protein phosphatase 2A catalytic subunit alpha isoform Ppp2ca**	sp|P63331|PP2AA_RAT	36 kDa	6.0924	7.3073	6.7937	11.023	7.6229	12.252	0.066921378	0.653552507
**Protein Atp6v1h Atp6v1h**	tr|E9PTI1|E9PTI1_RAT	51 kDa	4.0616	2.0878	4.8526	6.0125	5.7171	5.1048	0.087750867	0.653542746
**Erythrocyte protein band 4.1-like 3, isoform CRA_b Epb41l3**	tr|G3V874|G3V874_RAT	107 kDa	31.477	19.834	32.027	46.096	43.831	37.776	0.034358844	0.652592343
**Ethylmalonyl-CoA decarboxylase Echdc1**	sp|Q6AYG5|ECHD1_RAT	33 kDa	1	1	1.9411	3.0063	1	2.0419	0.346436404	0.651615357
**Syntaxin-binding protein 1 Stxbp1**	sp|P61765|STXB1_RAT	68 kDa	29.447	26.098	31.057	41.086	42.879	49.006	0.005309654	0.651284867
**ATPase, H+ transporting, V1 subunit G isoform 2 Atp6v1g2**	tr|Q8R2H0|Q8R2H0_RAT	14 kDa	5.077	5.2195	5.8232	8.0167	8.5757	8.1677	0.00052626	0.651035335
**ArfGAP with dual PH domains 1 Adap1**	tr|O88768|O88768_RAT	43 kDa	7.1078	3.1317	5.8232	9.0188	9.5286	6.1258	0.143371374	0.651018109
**Fructosamine-3-kinase-related protein Fn3krp**	tr|B2RYN1|B2RYN1_RAT	34 kDa	2.0308	2.0878	1	4.0083	2.8586	1	0.38724188	0.650650193
**Pyruvate dehydrogenase E1 component subunit beta, mitochondrial Pdhb**	sp|P49432|ODPB_RAT	39 kDa	17.262	14.615	18.44	28.058	24.774	24.503	0.004985676	0.65063684
**Protein Napg Napg**	tr|D4A0E2|D4A0E2_RAT	26 kDa	4.0616	5.2195	6.7937	9.0188	7.6229	8.1677	0.030732806	0.647931832
**WW domain-binding protein 2 Wbp2**	sp|Q8R478|WBP2_RAT (+1)	28 kDa	2.0308	3.1317	1.9411	3.0063	2.8586	5.1048	0.191222008	0.647565567
**Protein LOC100363782 LOC100363782**	tr|G3V6H0|G3V6H0_RAT	22 kDa	5.077	4.1756	1	4.0083	5.7171	6.1258	0.252310248	0.646802766
**Clathrin heavy chain 1 Cltc**	sp|P11442|CLH1_RAT	192 kDa	42.647	32.361	54.349	66.138	64.794	69.426	0.021933696	0.645629324
**Mitochondrial import receptor subunit TOM22 homolog Tomm22**	sp|Q75Q41|TOM22_RAT	15 kDa	2.0308	2.0878	1	1	2.8586	4.0839	0.383975639	0.644457035
**Dynamin-1-like protein Dnm1l**	sp|O35303|DNM1L_RAT	84 kDa	14.216	14.615	20.381	25.052	24.774	26.545	0.011864074	0.64438072
**Ras-related protein Rab-14 Rab14**	sp|P61107|RAB14_RAT (+1)	24 kDa	5.077	3.1317	5.8232	7.0146	6.67	8.1677	0.047362847	0.642124628
**Microtubule-associated protein 6 Map6**	sp|Q63560|MAP6_RAT	100 kDa	29.447	21.922	27.175	40.083	45.737	36.755	0.012995496	0.640783194
**Microtubule-associated protein Mapt**	tr|E9PT48|E9PT48_RAT	39 kDa	15.231	11.483	12.617	20.042	20.963	20.419	0.0029711	0.640319745
**Tubulin alpha-4A chain Tuba4a**	sp|Q5XIF6|TBA4A_RAT	50 kDa	63.97	63.678	68.907	101.21	103.86	102.1	3.87417E-05	0.639889963
**Complement component 1 Q subcomponent-binding protein, mitochondrial C1qbp**	sp|O35796|C1QBP_RAT	31 kDa	3.0462	4.1756	2.9116	6.0125	4.7643	5.1048	0.024856969	0.638059138
**Crk-like protein Crkl**	sp|Q5U2U2|CRKL_RAT	34 kDa	2.0308	2.0878	1	5.0104	1	2.0419	0.478511666	0.635669312
**Endophilin-B2 Sh3glb2**	sp|Q5PPJ9|SHLB2_RAT (+1)	45 kDa	9.1386	4.1756	10.676	9.0188	12.387	16.335	0.187188123	0.635656902
**Isoform 5 of Dynamin-1-like protein Dnm1l**	sp|O35303-5|DNM1L_RAT	83 kDa	14.216	14.615	20.381	26.054	23.821	27.566	0.014304824	0.635477331
**Isoform 5 of Myelin basic protein S Mbp**	sp|P02688-5|MBP_RAT	17 kDa	109.66	98.127	98.023	169.35	159.13	155.14	0.000659534	0.634961173
**Proteasome subunit beta type-3 Psmb3**	sp|P40112|PSB3_RAT (+1)	23 kDa	1	4.1756	2.9116	3.0063	6.67	3.0629	0.36628507	0.634827933
**Isoform Non-brain of Clathrin light chain A Clta**	sp|P08081-2|CLCA_RAT	24 kDa	3.0462	3.1317	3.8821	4.0083	5.7171	6.1258	0.051132439	0.634652266
**Myelin basic protein S Mbp**	sp|P02688|MBP_RAT	22 kDa	121.85	112.74	112.58	192.4	179.14	175.61	0.00036354	0.634506077
**Isoform 3 of Myelin basic protein S Mbp**	sp|P02688-3|MBP_RAT	17 kDa	116.77	104.39	106.76	184.38	166.75	166.42	0.000852397	0.633600618
**Casein kinase II subunit alpha Csnk2a1**	sp|P19139|CSK21_RAT	45 kDa	3.0462	6.2634	5.8232	9.0188	5.7171	9.1887	0.124925597	0.632520502
**NADH dehydrogenase [ubiquinone] 1 alpha subcomplex subunit 10, mitochondrial Ndufa10**	sp|Q561S0|NDUAA_RAT	40 kDa	7.1078	7.3073	8.7347	11.023	12.387	13.273	0.005587092	0.631077066
**Beta-synuclein Sncb**	sp|Q63754|SYUB_RAT	15 kDa	9.1386	6.2634	11.646	13.027	10.481	19.398	0.160647587	0.630401342
**Neurofilament medium polypeptide Nefm**	sp|P12839|NFM_RAT	96 kDa	92.402	89.776	100.93	143.3	130.54	175.61	0.016001348	0.629898765
**Peroxiredoxin 3 Prdx3**	tr|G3V7I0|G3V7I0_RAT	28 kDa	8.1232	6.2634	6.7937	10.021	12.387	11.231	0.009138919	0.629635245
**Serine/threonine-protein phosphatase 2A catalytic subunit beta isoform Ppp2cb**	sp|P62716|PP2AB_RAT	36 kDa	5.077	7.3073	5.8232	10.021	6.67	12.252	0.110272201	0.629081298
**Tenascin-R Tnr**	sp|Q05546|TENR_RAT	149 kDa	26.4	12.527	23.293	35.073	35.256	28.587	0.061326515	0.629018561
**26S proteasome non-ATPase regulatory subunit 2 Psmd2**	sp|Q4FZT9|PSMD2_RAT	100 kDa	6.0924	5.2195	4.8526	8.0167	9.5286	8.1677	0.006260788	0.628650877
**Eukaryotic initiation factor 4A-II Eif4a2**	sp|Q5RKI1|IF4A2_RAT	46 kDa	1	6.2634	5.8232	6.0125	6.67	8.1677	0.224418188	0.627648656
**Ac1164 Pdhx**	tr|Q7TQ85|Q7TQ85_RAT	59 kDa	5.077	3.1317	4.8526	7.0146	6.67	7.1467	0.014771672	0.627003596
**Microtubule-associated protein 1 A, isoform CRA_c Map1a**	tr|G3V7U2|G3V7U2_RAT	300 kDa	58.893	56.371	69.878	95.198	100.05	100.05	0.001177401	0.626966657
**Isocitrate dehydrogenase [NAD] subunit alpha, mitochondrial Idh3a**	sp|Q99NA5|IDH3A_RAT (+1)	40 kDa	13.2	10.439	13.587	21.044	19.057	19.398	0.003115793	0.625657574
**Microtubule-associated protein Map2**	tr|F1LNK0|F1LNK0_RAT	202 kDa	46.708	26.098	48.526	63.131	69.559	61.258	0.033629707	0.625590364
**Contactin-1 Cntn1**	sp|Q63198|CNTN1_RAT	113 kDa	24.37	16.703	32.027	35.073	39.067	42.88	0.042019993	0.624679542
**Isoform 6 of Breast carcinoma-amplified sequence 1 homolog Bcas1**	sp|Q3ZB98-6|BCAS1_RAT	57 kDa	17.262	14.615	14.558	28.058	23.821	22.461	0.008172282	0.624630078
**NADH-ubiquinone oxidoreductase 75 kDa subunit, mitochondrial Ndufs1**	sp|Q66HF1|NDUS1_RAT	79 kDa	20.308	20.878	19.411	29.061	32.397	35.734	0.003478064	0.623477241
**Phosphorylase Pygb**	tr|G3V6Y6|G3V6Y6_RAT	97 kDa	34.524	28.185	41.733	54.113	53.36	60.237	0.009248159	0.622753563
**Mitochondrial import receptor subunit TOM34 Tomm34**	sp|Q3KRD5|TOM34_RAT	34 kDa	6.0924	4.1756	5.8232	10.021	6.67	9.1887	0.049622801	0.621769186
**ADP-ribosylation factor-like protein 2 Arl2**	sp|O08697|ARL2_RAT (+1)	21 kDa	1	1	2.9116	3.0063	2.8586	2.0419	0.229256095	0.621186827
**Valine--tRNA ligase Vars**	sp|Q04462|SYVC_RAT	140 kDa	1	1	2.9116	3.0063	2.8586	2.0419	0.229256095	0.621186827
**Aa2-258 Ndufa8**	tr|Q7TP78|Q7TP78_RAT	22 kDa	4.0616	3.1317	3.8821	7.0146	5.7171	5.1048	0.023335854	0.620940207
**Microtubule-associated protein Map2**	tr|F1MAQ5|F1MAQ5_RAT	199 kDa	45.693	26.098	48.526	63.131	69.559	61.258	0.030541395	0.620357003
**Phosphatidylinositol 5-phosphate 4-kinase type-2 alpha Pip4k2a**	sp|Q9R0I8|PI42A_RAT	46 kDa	6.0924	4.1756	3.8821	6.0125	7.6229	9.1887	0.065665792	0.619963109
**Alpha-tubulin N-acetyltransferase Atat1**	sp|Q6MG11|ATAT_RAT	47 kDa	1	1	1	1	2.8586	1	0.373900966	0.61746182
**Protein Abhd11 Abhd11**	tr|D3ZXK4|D3ZXK4_RAT	33 kDa	1	1	1	1	2.8586	1	0.373900966	0.61746182
**ARP10 actin-related protein 10 homolog (S. cerevisiae) Actr10**	tr|Q5M9F7|Q5M9F7_RAT	46 kDa	1	1	1	1	2.8586	1	0.373900966	0.61746182
**Kinesin family member 3B (Predicted) Kif3b**	tr|D3ZI07|D3ZI07_RAT	85 kDa	1	1	1	1	2.8586	1	0.373900966	0.61746182
**Isoform 2 of ADP-ribosylation factor GTPase-activating protein 1 Arfgap1**	sp|Q62848-2|ARFG1_RAT (+1)	41 kDa	1	1	1	1	2.8586	1	0.373900966	0.61746182
**2-oxoisovalerate dehydrogenase subunit beta, mitochondrial Bckdhb**	sp|P35738|ODBB_RAT	43 kDa	1	1	1	1	2.8586	1	0.373900966	0.61746182
**Ubiquitin-conjugating enzyme E2 D2 Ube2d2**	sp|P62839|UB2D2_RAT	17 kDa	1	1	1	1	2.8586	1	0.373900966	0.61746182
**Acylphosphatase Acyp1**	tr|D4A6X4|D4A6X4_RAT	11 kDa	1	1	1	1	2.8586	1	0.373900966	0.61746182
**Protein Rab22a Rab22a**	tr|D4A9H0|D4A9H0_RAT	21 kDa	1	1	1	1	2.8586	1	0.373900966	0.61746182
**Neurofascin Nfasc**	sp|P97685|NFASC_RAT	138 kDa	15.231	11.483	20.381	26.054	25.727	24.503	0.020641902	0.617364061
**Glutathione S-transferase Mu 5 Gstm5**	sp|Q9Z1B2|GSTM5_RAT	27 kDa	6.0924	3.1317	4.8526	7.0146	7.6229	8.1677	0.034189171	0.617258345
**Microtubule-associated protein Mapt**	tr|D3ZNA6|D3ZNA6_RAT (+2)	81 kDa	17.262	13.571	15.528	25.052	27.633	22.461	0.006383751	0.616945679
**6-phosphogluconolactonase Pgls**	sp|P85971|6PGL_RAT (+1)	27 kDa	4.0616	4.1756	5.8232	9.0188	6.67	7.1467	0.033034839	0.615725515
**Isocitrate dehydrogenase [NAD] subunit beta, mitochondrial Idh3B**	sp|Q68FX0|IDH3B_RAT	42 kDa	13.2	10.439	13.587	19.04	20.01	21.44	0.003061408	0.615407505
**Cytochrome c, somatic Cycs**	sp|P62898|CYC_RAT (+1)	12 kDa	3.0462	1	3.8821	5.0104	3.8114	4.0839	0.148928289	0.614325453
**Guanidinoacetate N-methyltransferase Gamt**	sp|P10868|GAMT_RAT (+1)	26 kDa	2.0308	2.0878	1.9411	3.0063	3.8114	3.0629	0.008376671	0.613292715
**F-box only protein 2 Fbxo2**	tr|G3V774|G3V774_RAT	34 kDa	9.1386	7.3073	9.7053	13.027	13.34	16.335	0.012505109	0.612411597
**Phosphatidylethanolamine-binding protein 1 Pebp1**	sp|P31044|PEBP1_RAT	21 kDa	17.262	11.483	17.469	24.05	21.916	29.608	0.031571707	0.611506603
**Tyrosine--tRNA ligase, cytoplasmic Yars**	sp|Q4KM49|SYYC_RAT	59 kDa	6.0924	5.2195	8.7347	11.023	9.5286	12.252	0.032074872	0.611109756
**Cytochrome c oxidase subunit 6A1, mitochondrial Cox6a1**	sp|P10818|CX6A1_RAT	12 kDa	1	1	1	2.0042	1.9057	1	0.117173399	0.611010408
**Ubiquitin-like modifier-activating enzyme 5 Uba5**	sp|Q5M7A4|UBA5_RAT	45 kDa	1	1	1	2.0042	1.9057	1	0.117173399	0.611010408
**Enabled homolog (Drosophila) Enah**	tr|Q5XHX3|Q5XHX3_RAT	59 kDa	1	1	1	2.0042	1.9057	1	0.117173399	0.611010408
**Membrane protein, palmitoylated 2 (MAGUK p55 subfamily member 2), isoform CRA_a Mpp2**	tr|D3ZAA9|D3ZAA9_RAT	62 kDa	1	1	1	2.0042	1.9057	1	0.117173399	0.611010408
**Prefoldin 1 (Predicted) Pfdn1**	tr|D3ZX38|D3ZX38_RAT	14 kDa	1	1	1	2.0042	1.9057	1	0.117173399	0.611010408
**Carnitine O-palmitoyltransferase 2, mitochondrial Cpt2**	tr|G3V7N5|G3V7N5_RAT (+1)	74 kDa	1	1	1	2.0042	1.9057	1	0.117173399	0.611010408
**Protein Tcp11l1 Tcp11l1**	tr|F1M9Y7|F1M9Y7_RAT	56 kDa	1	1	1	2.0042	1.9057	1	0.117173399	0.611010408
**Isoform 4 of Mitochondrial peptide methionine sulfoxide reductase Msra**	sp|Q923M1-2|MSRA_RAT (+2)	22 kDa	1	1	1	2.0042	1.9057	1	0.117173399	0.611010408
**Protein LOC100910929 Vps37c**	tr|B5DFF4|B5DFF4_RAT	38 kDa	1	1	1	2.0042	1.9057	1	0.117173399	0.611010408
**Protein Cdh13 (Fragment) Cdh13**	tr|F1M7X3|F1M7X3_RAT	72 kDa	1	1	1	2.0042	1.9057	1	0.117173399	0.611010408
**Isoform 2 of Leukocyte surface antigen CD47 Cd47**	sp|P97829-2|CD47_RAT (+1)	35 kDa	1	1	1	2.0042	1.9057	1	0.117173399	0.611010408
**Adenylosuccinate lyase (Predicted) Adsl**	tr|D3ZW08|D3ZW08_RAT	55 kDa	1	1	1	2.0042	1.9057	1	0.117173399	0.611010408
**Regulator of G-protein-signaling 6 Rgs6**	tr|F1LS67|F1LS67_RAT	55 kDa	1	1	1	2.0042	1.9057	1	0.117173399	0.611010408
**60S ribosomal protein L36a Rpl36a**	sp|P83883|RL36A_RAT	12 kDa	1	1	1	2.0042	1.9057	1	0.117173399	0.611010408
**Nucleosome assembly protein 1-like 5 Nap1l5**	sp|Q5PPG6|NP1L5_RAT	17 kDa	1	1	1	2.0042	1.9057	1	0.117173399	0.611010408
**Hematological and neurological expressed 1 protein Hn1**	sp|Q6AXU6|HN1_RAT	16 kDa	1	1	1	2.0042	1.9057	1	0.117173399	0.611010408
**Protein Lrsam1 Lrsam1**	tr|D3ZI42|D3ZI42_RAT	84 kDa	1	1	1	2.0042	1.9057	1	0.117173399	0.611010408
**Neuronal pentraxin-1 Nptx1**	sp|P47971|NPTX1_RAT	47 kDa	1	1	1	2.0042	1.9057	1	0.117173399	0.611010408
**Protein Sdr39u1 Sdr39u1**	tr|D3ZYY0|D3ZYY0_RAT	32 kDa	1	1	1	2.0042	1.9057	1	0.117173399	0.611010408
**Synaptosomal-associated protein 23 Snap23**	sp|O70377|SNP23_RAT (+1)	23 kDa	1	1	1	2.0042	1.9057	1	0.117173399	0.611010408
**Disks large homolog 4 Dlg4**	sp|P31016|DLG4_RAT	80 kDa	1	3.1317	1.9411	3.0063	2.8586	4.0839	0.150477208	0.610405275
**Protein Endod1 Endod1**	tr|D3ZIP8|D3ZIP8_RAT	55 kDa	4.0616	1	5.8232	3.0063	6.67	8.1677	0.327686333	0.609997758

**Protein Ctnna2 (Fragment) Ctnna2**	tr|D4A6H8|D4A6H8_RAT	102 kDa	1	4.1756	1	3.0063	1	6.1258	0.510711177	0.609508394
**NADH dehydrogenase (Ubiquinone) 1 beta subcomplex 8 Ndufb8**	tr|B2RYS8|B2RYS8_RAT	22 kDa	2.0308	2.0878	1.9411	2.0042	2.8586	5.1048	0.232087085	0.607939725
**NADH dehydrogenase [ubiquinone] flavoprotein 3, mitochondrial Ndufv3**	sp|Q6PCU8|NDUV3_RAT	12 kDa	1	1	1	1	1.9057	2.0419	0.118058126	0.606354596
**Protein LOC100361144 LOC100361144**	tr|D4A4P3|D4A4P3_RAT	11 kDa	1	1	1	1	1.9057	2.0419	0.118058126	0.606354596
**Ubiquitin carboxyl-terminal hydrolase Usp8**	tr|D3ZN39|D3ZN39_RAT	123 kDa	1	1	1	1	1.9057	2.0419	0.118058126	0.606354596
**Endonuclease G-like 1 (Predicted), isoform CRA_d Exog**	tr|D3ZTW9|D3ZTW9_RAT	41 kDa	1	1	1	1	1.9057	2.0419	0.118058126	0.606354596
**Tyrosine-protein kinase Yes Yes1**	tr|Q6AXQ3|Q6AXQ3_RAT	54 kDa	1	1	1	1	1.9057	2.0419	0.118058126	0.606354596
**Protein Uba6 Uba6**	tr|D4A8H3|D4A8H3_RAT	118 kDa	1	1	1	1	1.9057	2.0419	0.118058126	0.606354596
**Protein Adam22 Adam22**	tr|M0R8N9|M0R8N9_RAT (+9)	107 kDa	1	1	1	1	1.9057	2.0419	0.118058126	0.606354596
**Mitochondrial import receptor subunit TOM20 homolog Tomm20**	sp|Q62760|TOM20_RAT	16 kDa	1	1	1	1	1.9057	2.0419	0.118058126	0.606354596
**Phosphatidylinositide phosphatase SAC1 Sacm1l**	sp|Q9ES21|SAC1_RAT	67 kDa	1	1	1	1	1.9057	2.0419	0.118058126	0.606354596
**Protein piccolo (Fragment) Pclo**	tr|D3Z9C7|D3Z9C7_RAT (+1)	427 kDa	1	1	1	1	1.9057	2.0419	0.118058126	0.606354596
**Protein LOC100911356 LOC100360976**	tr|G3V907|G3V907_RAT	32 kDa	1	1	1	1	1.9057	2.0419	0.118058126	0.606354596
**Complexin-2 Cplx2**	sp|P84087|CPLX2_RAT	15 kDa	1	1	1	1	1.9057	2.0419	0.118058126	0.606354596
**2',3'-cyclic-nucleotide 3'-phosphodiesterase Cnp**	sp|P13233|CN37_RAT	47 kDa	50.77	42.8	56.29	85.177	84.804	78.614	0.001792465	0.602827893
**Amylo-1, 6-glucosidase, 4-alpha-glucanotransferase (Glycogen debranching enzyme, glycogen storage disease type III) (Predicted), isoform CRA_a Agl**	tr|D4AEH9|D4AEH9_RAT	174 kDa	4.0616	3.1317	10.676	9.0188	10.481	10.21	0.177830235	0.601461471
**2-oxoglutarate dehydrogenase, mitochondrial Ogdh**	sp|Q5XI78|ODO1_RAT	116 kDa	10.154	12.527	18.44	24.05	21.916	22.461	0.023255189	0.600946995
**Calcium/calmodulin-dependent protein kinase type II subunit beta Camk2b**	sp|P08413|KCC2B_RAT (+1)	60 kDa	12.185	11.483	12.617	20.042	20.963	19.398	0.000138374	0.600715196
**Cytochrome b-c1 complex subunit 1, mitochondrial Uqcrc1**	sp|Q68FY0|QCR1_RAT	53 kDa	14.216	8.3513	15.528	21.044	20.963	21.44	0.018751708	0.600427128
**Cytochrome b-c1 complex subunit 7 Uqcrb**	tr|B2RYS2|B2RYS2_RAT	14 kDa	5.077	7.3073	4.8526	11.023	9.5286	8.1677	0.028174942	0.600185241
**Ras-related protein Rab-6A Rab6a**	sp|Q9WVB1|RAB6A_RAT	24 kDa	4.0616	4.1756	4.8526	9.0188	6.67	6.1258	0.034296598	0.600047674
**Ubiquitin carboxyl-terminal hydrolase isozyme L1 Uchl1**	sp|Q00981|UCHL1_RAT	25 kDa	22.339	14.615	19.411	28.058	34.303	31.65	0.012210273	0.599557499
**Protein CutA Cuta**	sp|Q6MGD0|CUTA_RAT (+1)	19 kDa	1	1	1	3.0063	1	1	0.373900966	0.599244951
**Sulfotransferase 4A1 Sult4a1**	sp|P63047|ST4A1_RAT	33 kDa	1	1	1	3.0063	1	1	0.373900966	0.599244951
**Protein Grsf1 Grsf1**	tr|F1LRK4|F1LRK4_RAT	53 kDa	1	1	1	3.0063	1	1	0.373900966	0.599244951
**Protein Umps Umps**	tr|Q4QQS7|Q4QQS7_RAT	52 kDa	1	1	1	3.0063	1	1	0.373900966	0.599244951
**Tryptophan--tRNA ligase, cytoplasmic Wars**	tr|F8WFH8|F8WFH8_RAT	53 kDa	9.1386	8.3513	9.7053	14.029	18.104	13.273	0.017331338	0.598934062
**ProSAAS Pcsk1n**	sp|Q9QXU9|PCSK1_RAT	27 kDa	6.0924	4.1756	5.8232	8.0167	7.6229	11.231	0.049575133	0.598840368
**Saccharopine dehydrogenase-like oxidoreductase Sccpdh**	sp|Q6AY30|SCPDL_RAT	47 kDa	5.077	2.0878	2.9116	6.0125	5.7171	5.1048	0.072715479	0.598560091
**D-beta-hydroxybutyrate dehydrogenase, mitochondrial Bdh1**	sp|P29147|BDH_RAT	38 kDa	4.0616	3.1317	2.9116	7.0146	4.7643	5.1048	0.044910171	0.598500329
**Casein kinase II subunit beta Csnk2b**	sp|P67874|CSK2B_RAT	25 kDa	3.0462	2.0878	1.9411	4.0083	4.7643	3.0629	0.057805894	0.597786321
**Alpha-internexin Ina**	tr|G3V8Q2|G3V8Q2_RAT	56 kDa	42.647	36.537	47.556	66.138	69.559	76.572	0.00298857	0.597072582
**ES1 protein homolog, mitochondrial**	sp|P56571|ES1_RAT	28 kDa	9.1386	7.3073	9.7053	14.029	11.434	18.377	0.051831546	0.596514599
**Proteasome (Prosome, macropain) 26S subunit, non-ATPase, 6 Psmd6**	tr|Q6PCT9|Q6PCT9_RAT	46 kDa	1	2.0878	1	1	3.8114	2.0419	0.362242916	0.596471773
**Guanine nucleotide-binding protein G(i) subunit alpha-1 Gnai1**	sp|P10824|GNAI1_RAT	40 kDa	10.154	6.2634	7.7642	13.027	14.293	13.273	0.010268884	0.59570862
**Peptidylprolyl cis/trans isomerase, NIMA-interacting 1 Pin1**	tr|B0BNL2|B0BNL2_RAT	18 kDa	6.0924	5.2195	5.8232	8.0167	9.5286	11.231	0.015774744	0.595458763
**Aldehyde dehydrogenase family 5, subfamily A1 Aldh5a1**	tr|G3V945|G3V945_RAT	56 kDa	10.154	8.3513	11.646	19.04	14.293	17.356	0.015301049	0.594829253
**Methylosome protein 50 Wdr77**	sp|Q4QR85|MEP50_RAT (+2)	37 kDa	1	1	1	2.0042	1	2.0419	0.116251538	0.594518539
**Mammary tumor virus receptor 2, isoform CRA_a Sssca1**	tr|D3ZG88|D3ZG88_RAT	21 kDa	1	1	1	2.0042	1	2.0419	0.116251538	0.594518539
**Protein Dnajb2 Dnajb2**	tr|D4ABC2|D4ABC2_RAT	29 kDa	1	1	1	2.0042	1	2.0419	0.116251538	0.594518539
**Voltage-dependent calcium channel subunit alpha-2/delta-1 Cacna2d1**	tr|D3ZKP9|D3ZKP9_RAT (+1)	120 kDa	1	1	1	2.0042	1	2.0419	0.116251538	0.594518539
**Protein Sypl1 Sypl1**	tr|Q66H18|Q66H18_RAT	29 kDa	1	1	1	2.0042	1	2.0419	0.116251538	0.594518539
**Synapsin-3 Syn3**	sp|O70441|SYN3_RAT	63 kDa	1	1	1	2.0042	1	2.0419	0.116251538	0.594518539
**Protein Acot13 Acot13**	tr|D3ZA93|D3ZA93_RAT	15 kDa	1	1	1	2.0042	1	2.0419	0.116251538	0.594518539
**Protein Srgap3 Srgap3**	tr|F1M5M9|F1M5M9_RAT	124 kDa	1	1	1	2.0042	1	2.0419	0.116251538	0.594518539
**Cul1 protein Cul1**	tr|B1WBY1|B1WBY1_RAT	90 kDa	1	1	1	2.0042	1	2.0419	0.116251538	0.594518539
**Alcohol dehydrogenase class-3 Adh5**	sp|P12711|ADHX_RAT	40 kDa	5.077	4.1756	4.8526	8.0167	8.5757	7.1467	0.002937965	0.594175853
**Isoform Beta-II of Protein kinase C beta type Prkcb**	sp|P68403-2|KPCB_RAT	77 kDa	1	3.1317	2.9116	3.0063	4.7643	4.0839	0.131764834	0.594145683
**Uncharacterized protein (Fragment) LOC100360985**	tr|F1LX07|F1LX07_RAT	74 kDa	6.0924	4.1756	9.7053	9.0188	12.387	12.252	0.080449438	0.593422624
**Kinesin family member 21B (Predicted) Kif21b**	tr|F1M5N7|F1M5N7_RAT	183 kDa	1	1	1	1	1	3.0629	0.373900966	0.592545774
**Protein Ubqln4 Ubqln4**	tr|D4A3P1|D4A3P1_RAT	64 kDa	1	1	1	1	1	3.0629	0.373900966	0.592545774
**Protein phosphatase 1H Ppm1h**	sp|Q5M821|PPM1H_RAT	56 kDa	1	1	1	1	1	3.0629	0.373900966	0.592545774
**Vesicle-fusing ATPase Nsf**	sp|Q9QUL6|NSF_RAT	83 kDa	37.57	30.273	34.939	59.123	57.171	57.174	0.000454326	0.59251274
**ADP-ribosylation factor 5 Arf5**	sp|P84083|ARF5_RAT	21 kDa	6.0924	1	5.8232	5.0104	7.6229	9.1887	0.22202967	0.591861424
**Catechol-O-methyltransferase domain containing 1 (Predicted), isoform CRA_a Comtd1**	tr|D3ZM21|D3ZM21_RAT	29 kDa	2.0308	2.0878	2.9116	4.0083	3.8114	4.0839	0.005366113	0.590594442
**Dynamin-like 120 kDa protein, mitochondrial Opa1**	tr|D4A8U5|D4A8U5_RAT (+2)	117 kDa	10.154	7.3073	7.7642	15.031	12.387	15.314	0.010450447	0.590318731
**Isocitrate dehydrogenase 3 (NAD), gamma Idh3g**	tr|Q5XIJ3|Q5XIJ3_RAT	43 kDa	8.1232	7.3073	9.7053	15.031	14.293	13.273	0.002585805	0.590083809
**Alpha-internexin Ina**	sp|P23565|AINX_RAT	56 kDa	44.678	38.625	49.497	71.148	74.323	80.656	0.001784451	0.587280599
**Neurofilament heavy polypeptide Nefh**	tr|F1LRZ7|F1LRZ7_RAT	114 kDa	76.155	58.459	79.583	120.25	112.44	133.75	0.004932977	0.584534985
**Argininosuccinate synthase Ass1**	sp|P09034|ASSY_RAT	46 kDa	2.0308	1	1	2.0042	2.8586	2.0419	0.096326105	0.583776268
**Myeloid leukemia factor 2 (Predicted), isoform CRA_a Mlf2**	tr|D3ZPN3|D3ZPN3_RAT	28 kDa	2.0308	3.1317	2.9116	6.0125	4.7643	3.0629	0.104576263	0.583401374
**Isoform 1 of Cytosolic acyl coenzyme A thioester hydrolase Acot7**	sp|Q64559-1|BACH_RAT (+2)	38 kDa	6.0924	4.1756	8.7347	11.023	11.434	10.21	0.029231375	0.58170937
**Pyridoxine-5'-phosphate oxidase Pnpo**	sp|O88794|PNPO_RAT	30 kDa	2.0308	1	1	3.0063	1.9057	2.0419	0.116512939	0.579645954
**Isochorismatase domain-containing protein 1 Isoc1**	tr|F2Z3T7|F2Z3T7_RAT	32 kDa	2.0308	1	1	3.0063	1.9057	2.0419	0.116512939	0.579645954
**Neuron-specific protein PEP-19 Pcp4**	tr|Q8CHN7|Q8CHN7_RAT	11 kDa	2.0308	1	1	3.0063	1.9057	2.0419	0.116512939	0.579645954
**Ubiquitin carboxyl-terminal hydrolase Usp5**	tr|D3ZVQ0|D3ZVQ0_RAT	96 kDa	9.1386	7.3073	15.528	13.027	22.869	19.398	0.110897177	0.578252613
**Hexokinase-1 Hk1**	sp|P05708|HXK1_RAT	102 kDa	14.216	10.439	23.293	28.058	29.539	25.524	0.042467633	0.576845803
**Glioblastoma amplified sequence Gbas**	tr|Q5RK08|Q5RK08_RAT	33 kDa	4.0616	2.0878	2.9116	4.0083	7.6229	4.0839	0.168873471	0.576579214
**AU RNA binding protein/enoyl-coenzyme A hydratase (Predicted), isoform CRA_a Auh**	tr|F1LU71|F1LU71_RAT	33 kDa	5.077	2.0878	4.8526	8.0167	5.7171	7.1467	0.065280801	0.575532195
**Cytoplasmic dynein 1 heavy chain 1 Dync1h1**	tr|F1LRT9|F1LRT9_RAT (+1)	532 kDa	38.585	24.01	59.202	56.117	76.229	79.635	0.075080913	0.574565645
**Lactoylglutathione lyase Glo1**	sp|Q6P7Q4|LGUL_RAT	21 kDa	4.0616	4.1756	4.8526	8.0167	7.6229	7.1467	0.000784902	0.574459215
**Delta-aminolevulinic acid dehydratase Alad**	sp|P06214|HEM2_RAT	36 kDa	1	1	1.9411	3.0063	2.8586	1	0.246053175	0.574094306
**Dihydropyrimidinase-related protein 4 Dpysl4**	tr|F1LNT0|F1LNT0_RAT	62 kDa	7.1078	4.1756	6.7937	11.023	12.387	8.1677	0.044149792	0.572464112
**Propionyl coenzyme A carboxylase, beta polypeptide Pccb**	tr|Q68FZ8|Q68FZ8_RAT	59 kDa	5.077	5.2195	1	7.0146	7.6229	5.1048	0.148917226	0.572197768
**Protein Rap2c Rap2c**	tr|D3ZK56|D3ZK56_RAT	21 kDa	2.0308	2.0878	1	1	2.8586	5.1048	0.359129118	0.571055626
**Synapsin-1 Syn1**	sp|P09951|SYN1_RAT	74 kDa	25.385	19.834	35.909	50.104	40.973	51.048	0.023496258	0.57082146
**NAD(P)H-hydrate epimerase Apoa1bp**	sp|B0BNM1|NNRE_RAT	31 kDa	1	1	1.9411	2.0042	2.8586	2.0419	0.078166139	0.570785117
**Cytochrome b-c1 complex subunit 8 Uqcrq**	sp|Q7TQ16|QCR8_RAT	10 kDa	3.0462	1	3.8821	4.0083	3.8114	6.1258	0.151150943	0.568520311
**Calcium/calmodulin-dependent protein kinase type II subunit beta Camk2b**	tr|F1LNI8|F1LNI8_RAT	65 kDa	12.185	10.439	11.646	19.04	20.963	20.419	0.000348211	0.567177518
**Drebrin Dbn1**	sp|Q07266|DREB_RAT	77 kDa	6.0924	4.1756	4.8526	11.023	9.5286	6.1258	0.068280435	0.566794365
**Trypa5|PromTArt5 Promega trypsin artifact 5 K to R mods (2239.1, 2914)**	zz|ZZ_FGCZCont0172|	6 kDa	1	1	1.9411	3.0063	1.9057	2.0419	0.098101362	0.566746718
**Phosphoglucomutase 3 (Predicted), isoform CRA_a Pgm3**	tr|D3ZFX4|D3ZFX4_RAT	61 kDa	1	2.0878	1.9411	1	3.8114	4.0839	0.28416945	0.565343496
**Mitochondrial import inner membrane translocase subunit Tim8 A Timm8a**	sp|Q9WVA1|TIM8A_RAT	11 kDa	1	1	1.9411	2.0042	1.9057	3.0629	0.105791077	0.565210532
**Nck-associated protein 1 Nckap1**	sp|P55161|NCKP1_RAT (+1)	129 kDa	1	1	1.9411	2.0042	1.9057	3.0629	0.105791077	0.565210532
**ATP synthase-coupling factor 6, mitochondrial Atp5j**	sp|P21571|ATP5J_RAT	12 kDa	1	2.0878	1.9411	4.0083	2.8586	2.0419	0.123448521	0.5644868
**S-phase kinase-associated protein 1 Skp1**	tr|Q6PEC4|SKP1_RAT	19 kDa	7.1078	5.2195	7.7642	11.023	11.434	13.273	0.00716828	0.562314582
**Pyridoxal kinase Pdxk**	sp|O35331|PDXK_RAT	35 kDa	8.1232	7.3073	10.676	14.029	17.151	15.314	0.007510877	0.561502559
**Cytoplasmic dynein 1 heavy chain 1 Dync1h1**	sp|P38650|DYHC1_RAT	532 kDa	36.554	24.01	58.232	55.115	77.181	79.635	0.070523143	0.560540931
**Pls1 protein Pls1**	tr|B5DEY0|B5DEY0_RAT	52 kDa	1	1	2.9116	3.0063	4.7643	1	0.36515232	0.560007297
**Voltage-gated potassium channel subunit beta-2 Kcnab2**	sp|P62483|KCAB2_RAT	41 kDa	4.0616	5.2195	6.7937	10.021	9.5286	9.1887	0.006985575	0.55935111
**Mitochondrial import inner membrane translocase subunit Tim9 Timm9**	sp|Q9WV97|TIM9_RAT	10 kDa	3.0462	3.1317	1	4.0083	3.8114	5.1048	0.075924895	0.555371581
**cAMP-dependent protein kinase type II-beta regulatory subunit Prkar2b**	sp|P12369|KAP3_RAT	46 kDa	5.077	4.1756	2.9116	9.0188	4.7643	8.1677	0.086690621	0.554157479
**Mitochondrial import receptor subunit TOM70 Tomm70a**	sp|Q75Q39|TOM70_RAT	67 kDa	4.0616	1	4.8526	4.0083	5.7171	8.1677	0.18947842	0.554079506
**Protein Tom1l2 Tom1l2**	tr|D4A6C9|D4A6C9_RAT	31 kDa	1	1	2.9116	5.0104	2.8586	1	0.374956155	0.553794114
**Protein Ndufa13 LOC100911483**	tr|D3ZE15|D3ZE15_RAT	17 kDa	3.0462	7.3073	7.7642	11.023	10.481	11.231	0.032652371	0.553465709
**Inosine triphosphate pyrophosphatase Itpa**	sp|D3ZW55|ITPA_RAT	22 kDa	2.0308	2.0878	1.9411	4.0083	2.8586	4.0839	0.014988298	0.553356832
**Alpha-aminoadipic semialdehyde dehydrogenase Aldh7a1**	sp|Q64057|AL7A1_RAT	59 kDa	3.0462	3.1317	4.8526	7.0146	4.7643	8.1677	0.062456106	0.553001514
**Ras-related protein Rab-2A Rab2a**	sp|P05712|RAB2A_RAT	24 kDa	3.0462	1	6.7937	5.0104	8.5757	6.1258	0.212675895	0.549916548
**Protein Serpinb6 Serpinb6**	tr|Q6P9U0|Q6P9U0_RAT	43 kDa	3.0462	3.1317	1.9411	4.0083	5.7171	5.1048	0.023774629	0.547463959
**Neurotrimin Ntm**	tr|G3V964|G3V964_RAT	38 kDa	2.0308	1	2.9116	4.0083	3.8114	3.0629	0.057375396	0.546045982
**Isoform 2 of Calcineurin subunit B type 1 Ppp3r1**	sp|P63100-2|CANB1_RAT (+1)	25 kDa	1	1	2.9116	3.0063	1.9057	4.0839	0.202955994	0.545982058
**RCG45400 Tubb4b**	tr|G3V7C6|G3V7C6_RAT	50 kDa	119.82	1	113.55	146.3	140.07	143.96	0.16625851	0.544628541
**AP-2 complex subunit alpha-2 Ap2a2**	sp|P18484|AP2A2_RAT	104 kDa	13.2	10.439	13.587	24.05	20.963	23.482	0.001610505	0.543484926
**Protein Tln2 Tln2**	tr|D3ZA84|D3ZA84_RAT	237 kDa	4.0616	6.2634	10.676	13.027	17.151	12.252	0.038882876	0.54281876
**Prostaglandin E synthase 3 Ptges3**	sp|P83868|TEBP_RAT	19 kDa	3.0462	1	2.9116	5.0104	3.8114	4.0839	0.058187991	0.539126123
**Proteasome subunit beta type-6 Psmb6**	sp|P28073|PSB6_RAT	25 kDa	1	2.0878	3.8821	6.0125	2.8586	4.0839	0.184174831	0.538008491
**Cytoplasmic dynein 1 light intermediate chain 2 Dync1li2**	sp|Q62698|DC1L2_RAT	55 kDa	2.0308	3.1317	3.8821	6.0125	5.7171	5.1048	0.012400043	0.537268926
**Pirin Pir**	sp|Q5M827|PIR_RAT	32 kDa	3.0462	2.0878	3.8821	7.0146	5.7171	4.0839	0.059039482	0.536174743
**Isoform 2 of AP-2 complex subunit beta Ap2b1**	sp|P62944-2|AP2B1_RAT (+1)	106 kDa	7.1078	8.3513	12.617	18.038	18.104	16.335	0.009985006	0.535017246
**Glucose-6-phosphate isomerase Gpi**	sp|Q6P6V0|G6PI_RAT	63 kDa	14.216	12.527	20.381	28.058	31.444	28.587	0.006360496	0.534958962
**4-aminobutyrate aminotransferase, mitochondrial Abat**	sp|P50554|GABT_RAT	56 kDa	12.185	15.659	21.352	35.073	29.539	27.566	0.014805677	0.533706524
**Microtubule-associated protein RP/EB family member 3 Mapre3**	sp|Q5XIT1|MARE3_RAT	32 kDa	4.0616	7.3073	7.7642	11.023	8.5757	16.335	0.094821819	0.532455606
**Neurofilament light polypeptide Nefl**	sp|P19527|NFL_RAT	61 kDa	77.171	65.766	98.994	141.29	144.83	169.48	0.00567242	0.531016242
**Glycine--tRNA ligase Gars**	tr|G3V7G8|G3V7G8_RAT	82 kDa	4.0616	5.2195	4.8526	8.0167	10.481	8.1677	0.008554669	0.530038927
**Rho-related GTP-binding protein RhoB Rhob**	sp|P62747|RHOB_RAT	22 kDa	4.0616	2.0878	4.8526	7.0146	7.6229	6.1258	0.024923013	0.529877235
**Dihydropyrimidinase-related protein 5 Dpysl5**	sp|Q9JHU0|DPYL5_RAT	62 kDa	19.293	12.527	18.44	31.065	31.444	32.671	0.002361378	0.528052112
**ATPase, H+ transporting, V1 subunit D, isoform CRA_c Atp6v1d**	tr|Q6P503|Q6P503_RAT	28 kDa	1	3.1317	5.8232	7.0146	5.7171	6.1258	0.109600413	0.527901366
**Serine/threonine-protein kinase PAK 1 Pak1**	sp|P35465|PAK1_RAT	61 kDa	6.0924	5.2195	5.8232	11.023	12.387	9.1887	0.005850546	0.525637525
**Secretogranin-2 Scg2**	sp|P10362|SCG2_RAT (+1)	71 kDa	5.077	2.0878	5.8232	9.0188	8.5757	7.1467	0.037055447	0.524954327
**COP9 signalosome complex subunit 4 Cops4**	sp|Q68FS2|CSN4_RAT	46 kDa	5.077	3.1317	5.8232	10.021	8.5757	8.1677	0.012327575	0.524274783
**Gamma-synuclein Sncg**	sp|Q63544|SYUG_RAT (+1)	13 kDa	9.1386	5.2195	9.7053	15.031	10.481	20.419	0.084973856	0.523903246

**Creatine kinase, mitochondrial 1, ubiquitous Ckmt1b**	tr|Q5BJT9|Q5BJT9_RAT	47 kDa	12.185	14.615	23.293	32.067	32.397	31.65	0.010491133	0.521183178
**Gamma-enolase Eno2**	sp|P07323|ENOG_RAT	47 kDa	27.416	27.142	31.057	54.113	55.266	55.132	3.67E-05	0.520421127
**Cytochrome b-c1 complex subunit 6, mitochondrial Uqcrh**	sp|Q5M9I5|QCR6_RAT	10 kDa	1	3.1317	1	6.0125	2.8586	1	0.386386809	0.519871139
**Adaptor protein complex AP-2, alpha 1 subunit (Predicted) Ap2a1**	tr|D3ZUY8|D3ZUY8_RAT	108 kDa	12.185	9.3952	14.558	24.05	20.963	24.503	0.003933461	0.519854422
**NADH dehydrogenase [ubiquinone] iron-sulfur protein 4, mitochondrial Ndufs4**	sp|Q5XIF3|NDUS4_RAT	20 kDa	4.0616	5.2195	2.9116	6.0125	11.434	6.1258	0.11780208	0.517246938
**Eukaryotic translation initiation factor 5A2 (Predicted) Eif5a2**	tr|G3V7J7|G3V7J7_RAT	17 kDa	1	1	1	1	3.8114	1	0.373900966	0.516226727
**Uncharacterized protein**	tr|M0R608|M0R608_RAT	82 kDa	1	1	1	1	3.8114	1	0.373900966	0.516226727
**Syntenin-1 Sdcbp**	sp|Q9JI92|SDCB1_RAT	32 kDa	1	1	1	1	3.8114	1	0.373900966	0.516226727
**Contactin-associated protein 1 Cntnap1**	sp|P97846|CNTP1_RAT	156 kDa	1	1	1	1	3.8114	1	0.373900966	0.516226727
**Protein Sbf1 Sbf1**	tr|D3ZNN0|D3ZNN0_RAT (+1)	209 kDa	1	1	1	1	3.8114	1	0.373900966	0.516226727
**Pyruvate carboxylase, mitochondrial Pc**	sp|P52873|PYC_RAT	130 kDa	10.154	9.3952	14.558	23.048	22.869	20.419	0.004115437	0.514158225
**Phosphorylase Pygm**	tr|G3V8V3|G3V8V3_RAT	97 kDa	23.354	14.615	25.234	43.09	38.114	41.86	0.005169139	0.513578301
**Ataxin-10 Atxn10**	sp|Q9ER24|ATX10_RAT	54 kDa	1	1	1	2.0042	2.8586	1	0.150267827	0.511700894
**HBS1-like protein Hbs1l**	sp|Q6AXM7|HBS1L_RAT (+1)	75 kDa	1	1	1	2.0042	2.8586	1	0.150267827	0.511700894
**Protein RGD1564809 (Fragment) Fsd1l**	tr|D3ZC90|D3ZC90_RAT (+1)	52 kDa	1	1	1	2.0042	2.8586	1	0.150267827	0.511700894
**Protein Agk Agk**	tr|D3Z9L0|D3Z9L0_RAT	47 kDa	1	1	1	2.0042	2.8586	1	0.150267827	0.511700894
**Phosphohistidine phosphatase 1 (Predicted), isoform CRA_a Phpt1**	tr|D3ZP47|D3ZP47_RAT	14 kDa	1	1	1	2.0042	2.8586	1	0.150267827	0.511700894
**Calcium-binding mitochondrial carrier protein SCaMC-2 Slc25a25**	sp|Q8K3P6|SCMC2_RAT	53 kDa	1	1	1	2.0042	2.8586	1	0.150267827	0.511700894
**Methylthioribose-1-phosphate isomerase Mri1**	sp|Q5HZE4|MTNA_RAT	40 kDa	1	1	1	1	2.8586	2.0419	0.146645143	0.508431489
**NADH dehydrogenase (Ubiquinone) Fe-S protein 7 Ndufs7**	tr|Q5RJN0|Q5RJN0_RAT	24 kDa	3.0462	3.1317	3.8821	7.0146	6.67	6.1258	0.000933341	0.507814077
**Citrate lyase subunit beta-like protein, mitochondrial Clybl**	sp|Q5I0K3|CLYBL_RAT	37 kDa	1	1	1	3.0063	1.9057	1	0.169576053	0.50744249
**Protein LZIC Lzic**	sp|Q5PQN7|LZIC_RAT	21 kDa	1	1	1	3.0063	1.9057	1	0.169576053	0.50744249
**Ubiquitin carboxyl-terminal hydrolase 7 Usp7**	sp|Q4VSI4|UBP7_RAT (+1)	128 kDa	1	1	1	3.0063	1.9057	1	0.169576053	0.50744249
**Protein Wdr37 Wdr37**	tr|D3ZQ02|D3ZQ02_RAT	50 kDa	3.0462	1	1	3.0063	2.8586	4.0839	0.105429615	0.507216951
**Phytanoyl-CoA hydroxylase-interacting protein-like Phyhipl**	sp|Q6AYN4|PHIPL_RAT	42 kDa	3.0462	3.1317	4.8526	8.0167	7.6229	6.1258	0.012197605	0.506790594
**Guanine nucleotide-binding protein G(s) subunit alpha isoforms short Gnas**	sp|P63095|GNAS2_RAT	46 kDa	2.0308	1	1.9411	3.0063	4.7643	2.0419	0.134690842	0.506690446
**Secernin-1 Scrn1**	sp|Q6AY84|SCRN1_RAT	46 kDa	7.1078	7.3073	8.7347	15.031	13.34	17.356	0.00408543	0.506261071
**Inter-alpha-trypsin inhibitor heavy chain H3 Itih3**	sp|Q63416|ITIH3_RAT	99 kDa	3.0462	3.1317	3.8821	7.0146	5.7171	7.1467	0.003439492	0.506076948
**Thy-1 membrane glycoprotein Thy1**	sp|P01830|THY1_RAT	18 kDa	3.0462	2.0878	3.8821	5.0104	5.7171	7.1467	0.022261978	0.504419778
**Synaptotagmin-1 Syt1**	sp|P21707|SYT1_RAT	47 kDa	1	1	1	2.0042	1.9057	2.0419	1.72021E-05	0.504049195
**Sepiapterin reductase Spr**	sp|P18297|SPRE_RAT	28 kDa	1	1	1	2.0042	1.9057	2.0419	1.72021E-05	0.504049195
**Acylphosphatase-2 Acyp2**	sp|P35745|ACYP2_RAT (+1)	11 kDa	1	1	1	2.0042	1.9057	2.0419	1.72021E-05	0.504049195
**Prolyl endopeptidase Prep**	sp|O70196|PPCE_RAT	81 kDa	1	1	1	2.0042	1.9057	2.0419	1.72021E-05	0.504049195
**NADH dehydrogenase [ubiquinone] 1 alpha subcomplex subunit 11 Ndufa11**	sp|Q80W89|NDUAB_RAT	15 kDa	1	1	1	2.0042	1.9057	2.0419	1.72021E-05	0.504049195
**ATPase, H transporting, lysosomal V1 subunit G1 Atp6v1g1**	tr|B2GUV5|B2GUV5_RAT	14 kDa	1	1	1	2.0042	1.9057	2.0419	1.72021E-05	0.504049195
**GrpE protein homolog 1, mitochondrial Grpel1**	sp|P97576|GRPE1_RAT	24 kDa	1	1	1	2.0042	1.9057	2.0419	1.72021E-05	0.504049195
**Cytoplasmic polyadenylation element binding protein 4 (Predicted) Cpeb4**	tr|D3ZKL3|D3ZKL3_RAT	79 kDa	1	1	1	2.0042	1.9057	2.0419	1.72021E-05	0.504049195
**Protein Trappc5 Trappc5**	tr|B0BNE3|B0BNE3_RAT	21 kDa	1	1	1	2.0042	1.9057	2.0419	1.72021E-05	0.504049195
**Protein phosphatase 1E Ppm1e**	sp|Q80Z30|PPM1E_RAT	83 kDa	1	1	1	2.0042	1.9057	2.0419	1.72021E-05	0.504049195
**LOC681996 protein LOC681996**	tr|B0BN63|B0BN63_RAT	38 kDa	3.0462	2.0878	2.9116	5.0104	3.8114	7.1467	0.06079683	0.503841939
**Guanine nucleotide binding protein, alpha q polypeptide, isoform CRA_a Gnaq**	tr|D4AE68|D4AE68_RAT	42 kDa	3.0462	2.0878	3.8821	6.0125	4.7643	7.1467	0.026131667	0.503032332
**Small VCP/p97-interacting protein Svip**	sp|P0C0A9|SVIP_RAT	8 kDa	1	1	1	1	1.9057	3.0629	0.172746145	0.502630433
**Importin 7 (Predicted), isoform CRA_c Ipo7**	tr|D4AE96|D4AE96_RAT	119 kDa	1	1	1	1	1.9057	3.0629	0.172746145	0.502630433
**Protein Ube2m Ube2m**	tr|D3ZNQ6|D3ZNQ6_RAT	21 kDa	2.0308	1	1.9411	2.0042	3.8114	4.0839	0.087975894	0.502237487
**Elongation factor Ts, mitochondrial Tsfm**	sp|Q9QYU2|EFTS_RAT	35 kDa	2.0308	1	2.9116	4.0083	4.7643	3.0629	0.056678775	0.502082717
**Myelin-associated glycoprotein Mag**	sp|P07722|MAG_RAT	69 kDa	12.185	8.3513	11.646	20.042	25.727	18.377	0.013520459	0.501703925
**Endophilin-A3 Sh3gl3**	sp|O35180|SH3G3_RAT (+1)	39 kDa	1	1	1.9411	2.0042	3.8114	2.0419	0.124701311	0.501571747
**Leucyl-tRNA synthetase Lars**	tr|Q5PPJ6|Q5PPJ6_RAT	134 kDa	1	1	1.9411	4.0083	2.8586	1	0.232506808	0.500972429
**Epidermal growth factor receptor pathway substrate 15-like 1 Eps15l1**	tr|D3ZJR1|D3ZJR1_RAT	96 kDa	3.0462	1	1.9411	5.0104	2.8586	4.0839	0.081604079	0.50090773
**Brevican, isoform CRA_a Bcan**	tr|G3V8G4|G3V8G4_RAT	96 kDa	12.185	8.3513	12.617	23.048	23.821	19.398	0.004564447	0.500298791
**Protein Ppp2r1a Ppp2r1a**	tr|Q5XI34|Q5XI34_RAT	65 kDa	9.1386	10.439	13.587	24.05	21.916	20.419	0.002801009	0.499579724
**Ras-related protein M-Ras Mras**	sp|P97538|RASM_RAT (+1)	24 kDa	2.0308	1	1	4.0083	1	3.0629	0.23019341	0.499405293
**Protein Mrpl12 Mrpl12**	tr|D3ZXF9|D3ZXF9_RAT	29 kDa	1	1	1	4.0083	1	1	0.373900966	0.499309289
**Glycogen synthase kinase-3 beta Gsk3b**	sp|P18266|GSK3B_RAT	47 kDa	1	1	1	4.0083	1	1	0.373900966	0.499309289
**gi|88044|pir||S01068 keratin 4, type II, cytoskeletal - human (fragmen**	zz|ZZ_FGCZCont0085|	46 kDa	1	1	1	4.0083	1	1	0.373900966	0.499309289
**Guanine nucleotide-binding protein subunit beta-5 Gnb5**	sp|P62882|GBB5_RAT	39 kDa	2.0308	1	2.9116	4.0083	3.8114	4.0839	0.023609191	0.499210323
**Stathmin Stmn1**	sp|P13668|STMN1_RAT	17 kDa	7.1078	7.3073	3.8821	15.031	11.434	10.21	0.028321711	0.498901159
**2-hydroxyacylsphingosine 1-beta-galactosyltransferase Ugt8**	sp|Q09426|CGT_RAT	61 kDa	1	1	1.9411	3.0063	2.8586	2.0419	0.038179671	0.498444377
**Acyl-coenzyme A thioesterase THEM4 Them4**	sp|Q566R0|THEM4_RAT	26 kDa	1	1	1.9411	3.0063	2.8586	2.0419	0.038179671	0.498444377
**Mitochondrial dicarboxylate carrier Slc25a10**	tr|O89035|O89035_RAT	31 kDa	1	1	1.9411	3.0063	2.8586	2.0419	0.038179671	0.498444377
**CB1 cannabinoid receptor-interacting protein 1 Cnrip1**	sp|Q5M7A7|CNRP1_RAT	19 kDa	1	1	2.9116	4.0083	3.8114	2.0419	0.138263924	0.498053054
**Long-chain fatty acid transport protein 1 Slc27a1**	tr|Q6GMM8|Q6GMM8_RAT	71 kDa	1	1	1.9411	2.0042	2.8586	3.0629	0.042223825	0.497255763
**Protein Camk1d (Fragment) Camk1d**	tr|F1LVR4|F1LVR4_RAT	43 kDa	1	1	1.9411	2.0042	2.8586	3.0629	0.042223825	0.497255763
**Phosphodiesterase 6D, cGMP-specific, rod, delta (Predicted), isoform CRA_b Pde6d**	tr|D3ZRD3|D3ZRD3_RAT	15 kDa	1	1	1	3.0063	1	2.0419	0.154308774	0.496015343
**Ubiquitin carboxyl-terminal hydrolase isozyme L3 Uchl3**	sp|Q91Y78|UCHL3_RAT (+1)	26 kDa	1	1	1	3.0063	1	2.0419	0.154308774	0.496015343
**Protein Ccdc92 Ccdc92**	tr|D3ZBX9|D3ZBX9_RAT	35 kDa	1	1	1	3.0063	1	2.0419	0.154308774	0.496015343
**Uncharacterized protein Lss**	tr|G3V998|G3V998_RAT	82 kDa	1	1	1	3.0063	1	2.0419	0.154308774	0.496015343
**Protein TSSC1 Tssc1**	sp|Q5PPK9|TSSC1_RAT	43 kDa	1	1	1	3.0063	1	2.0419	0.154308774	0.496015343
**Cystatin-B Cstb**	sp|P01041|CYTB_RAT	11 kDa	1	1	1	2.0042	1	3.0629	0.161190631	0.494470175
**Isoform 2 of Actin-related protein 2/3 complex subunit 5-like protein Arpc5l**	sp|A1L108-2|ARP5L_RAT (+2)	11 kDa	1	1	1	2.0042	1	3.0629	0.161190631	0.494470175
**Tripeptidyl-peptidase 2 Tpp2**	sp|Q64560|TPP2_RAT	138 kDa	1	1	1	2.0042	1	3.0629	0.161190631	0.494470175
**Centrin 2, isoform CRA_a Cetn2**	tr|G3V9W0|G3V9W0_RAT	20 kDa	1	1	1	2.0042	1	3.0629	0.161190631	0.494470175
**Synapsin-2 Syn2**	sp|Q63537|SYN2_RAT	63 kDa	12.185	11.483	15.528	27.056	25.727	26.545	0.00051291	0.494100444
**Ras-related protein Rab-35 Rab35**	sp|Q5U316|RAB35_RAT	23 kDa	1	1	1	1	1	4.0839	0.373900966	0.493104752
**ELAV (Embryonic lethal, abnormal vision, Drosophila)-like 3 (Hu antigen C), isoform CRA_b Elavl3**	tr|Q76IJ9|Q76IJ9_RAT	40 kDa	1	1	1	1	1	4.0839	0.373900966	0.493104752
**Isoform 2 of Ethanolamine-phosphate cytidylyltransferase Pcyt2**	sp|O88637-2|PCY2_RAT (+1)	43 kDa	1	1	1.9411	2.0042	1.9057	4.0839	0.156852459	0.49301959
**6-phosphofructokinase, liver type Pfkl**	sp|P30835|K6PL_RAT	85 kDa	3.0462	5.2195	2.9116	8.0167	8.5757	6.1258	0.021705974	0.491997605
**Guanylate kinase 1, isoform CRA_a Guk1**	tr|E9PTV0|E9PTV0_RAT	24 kDa	1	1	1.9411	6.0125	1	1	0.469406994	0.491868955
**Advillin Avil**	sp|Q9WU06|AVIL_RAT	93 kDa	1	1	3.8821	3.0063	2.8586	6.1258	0.228781469	0.49055518
**Isoform 2 of Protein kinase C and casein kinase substrate in neurons 2 protein Pacsin2**	sp|Q9QY17-2|PACN2_RAT (+3)	52 kDa	2.0308	5.2195	1	6.0125	5.7171	5.1048	0.092173204	0.490085777
**Cell adhesion molecule 3 Cadm3**	sp|Q1WIM3|CADM3_RAT	43 kDa	4.0616	3.1317	3.8821	6.0125	9.5286	7.1467	0.022710835	0.488165446
**Protein LOC688963 LOC688963**	tr|F1LPG5|F1LPG5_RAT	15 kDa	3.0462	3.1317	2.9116	6.0125	7.6229	5.1048	0.012131931	0.485026841
**Phosphoglucomutase 1 Pgm1**	tr|Q499Q4|Q499Q4_RAT	61 kDa	3.0462	2.0878	8.7347	9.0188	11.434	8.1677	0.098646756	0.484572247
**Tubulin polymerization-promoting protein family member 3 Tppp3**	sp|Q5PPN5|TPPP3_RAT	19 kDa	2.0308	4.1756	3.8821	7.0146	6.67	7.1467	0.006435969	0.484295267
**Synaptosomal-associated protein 25 Snap25**	sp|P60881|SNP25_RAT	23 kDa	10.154	7.3073	6.7937	17.035	16.199	17.356	0.001338664	0.479442578
**Hdac11 protein Hdac11**	tr|B2GUW3|B2GUW3_RAT	39 kDa	2.0308	3.1317	1.9411	6.0125	4.7643	4.0839	0.019268155	0.478012476
**Ubiquitin-like modifier-activating enzyme 1 Uba1**	sp|Q5U300|UBA1_RAT	118 kDa	10.154	11.483	17.469	25.052	32.397	24.503	0.013644157	0.477181765
**Protein Cops7a Cops7a**	tr|F1MAA2|F1MAA2_RAT (+1)	30 kDa	3.0462	2.0878	2.9116	4.0083	5.7171	7.1467	0.036943703	0.476858245
**Hyaluronan and proteoglycan link protein 2 Hapln2**	tr|G3V8G6|G3V8G6_RAT	38 kDa	9.1386	5.2195	6.7937	15.031	14.293	15.314	0.002666265	0.473851875
**Isoform 2 of Serine/threonine-protein phosphatase 2B catalytic subunit alpha isoform Ppp3ca**	sp|P63329-2|PP2BA_RAT	58 kDa	6.0924	3.1317	4.8526	12.025	9.5286	8.1677	0.021280697	0.473623294
**Microtubule-associated protein RP/EB family member 2 Mapre2**	sp|Q3B8Q0|MARE2_RAT (+1)	37 kDa	6.0924	3.1317	5.8232	11.023	7.6229	13.273	0.041227898	0.471422887
**Ras-related protein Rab-2A (Fragment) Rab2a**	tr|F1LP82|F1LP82_RAT	22 kDa	1	1	7.7642	6.0125	8.5757	6.1258	0.20367651	0.471381674
**Isoform 2 of Echinoderm microtubule-associated protein-like 1 Eml1**	sp|Q4V8C3-2|EMAL1_RAT (+1)	87 kDa	2.0308	2.0878	1.9411	4.0083	4.7643	4.0839	0.000750212	0.471333567
**Ectonucleotide pyrophosphatase/phosphodiesterase family member 6 Enpp6**	sp|B0BND0|ENPP6_RAT	51 kDa	8.1232	7.3073	10.676	17.035	19.057	19.398	0.001451398	0.470472157
**Proteasome subunit beta type-7 Psmb7**	sp|Q9JHW0|PSB7_RAT	30 kDa	2.0308	2.0878	1.9411	5.0104	3.8114	4.0839	0.003350212	0.469536716
**Isoform 2 of Kinesin-like protein KIF2A Kif2a**	sp|Q9WV63-2|KIF2A_RAT (+1)	84 kDa	4.0616	3.1317	3.8821	7.0146	9.5286	7.1467	0.008276159	0.467515692
**Ubiquitin carboxyl-terminal hydrolase Usp14**	tr|Q5U2N2|Q5U2N2_RAT	56 kDa	2.0308	4.1756	4.8526	8.0167	9.5286	6.1258	0.031924907	0.467194173
**Heat shock protein 105 kDa Hsph1**	sp|Q66HA8|HS105_RAT	96 kDa	13.2	8.3513	15.528	27.056	23.821	28.587	0.005102124	0.466617588
**NADH dehydrogenase (Ubiquinone) 1 alpha subcomplex, 12 (Predicted), isoform CRA_b Ndufa12**	tr|F1LXA0|F1LXA0_RAT	17 kDa	3.0462	4.1756	4.8526	9.0188	5.7171	11.231	0.05159259	0.464991971
**Protein RUFY3 Rufy3**	sp|Q5FVJ0|RUFY3_RAT	53 kDa	9.1386	5.2195	5.8232	12.025	16.199	15.314	0.011472254	0.463533006
**Protein Nars Nars**	tr|F1LPV0|F1LPV0_RAT	64 kDa	2.0308	4.1756	3.8821	6.0125	7.6229	8.1677	0.013829153	0.462709431
**Fam49b protein Fam49b**	tr|B2GUZ9|B2GUZ9_RAT	37 kDa	3.0462	1	1.9411	3.0063	3.8114	6.1258	0.104127679	0.462571947
**Acyl-protein thioesterase 2 Lypla2**	sp|Q9QYL8|LYPA2_RAT	25 kDa	1	2.0878	1	3.0063	3.8114	2.0419	0.06419201	0.461397806
**Phospholysine phosphohistidine inorganic pyrophosphate phosphatase Lhpp**	sp|Q5I0D5|LHPP_RAT	29 kDa	1	1	3.8821	5.0104	5.7171	2.0419	0.195818034	0.460640281
**Protein LOC684352 LOC684352**	tr|B0BMY7|B0BMY7_RAT	39 kDa	1	2.0878	1	2.0042	3.8114	3.0629	0.066395313	0.460415611
**Protein Ubqln2 Ubqln2**	tr|D4AA63|D4AA63_RAT	67 kDa	4.0616	3.1317	1.9411	7.0146	5.7171	7.1467	0.00942082	0.459513844
**Protein LOC100909742 (Fragment) RGD1566085**	tr|D3Z8P5|D3Z8P5_RAT	35 kDa	1	1	9.7053	1	13.34	11.231	0.389216386	0.457756834
**Phosphoribosyl pyrophosphate synthase-associated protein 1 Prpsap1**	sp|Q63468|KPRA_RAT (+1)	39 kDa	2.0308	1	1	4.0083	3.8114	1	0.196321281	0.457022348
**COP9 signalosome complex subunit 1 Gps1**	sp|P97834|CSN1_RAT	53 kDa	1	2.0878	1	2.0042	2.8586	4.0839	0.082906894	0.455905898
**Protein kinase C and casein kinase substrate in neurons protein 1 Pacsin1**	sp|Q9Z0W5|PACN1_RAT	50 kDa	10.154	8.3513	12.617	23.048	21.916	23.482	0.000709551	0.454698595
**Serine--tRNA ligase, cytoplasmic Sars**	sp|Q6P799|SYSC_RAT	59 kDa	5.077	5.2195	7.7642	15.031	11.434	13.273	0.005980202	0.454494439
**Cysteine and histidine-rich domain-containing protein 1 Chordc1**	sp|D4A4T9|CHRD1_RAT	37 kDa	1	4.1756	1.9411	6.0125	5.7171	4.0839	0.060269076	0.450039523
**Isoform 6 of Dynamin-1 Dnm1**	sp|P21575-6|DYN1_RAT	96 kDa	21.323	26.098	32.998	58.121	60.03	61.258	0.00071323	0.448243957
**Neurocalcin-delta Ncald**	sp|Q5PQN0|NCALD_RAT	22 kDa	1	1	1.9411	2.0042	4.7643	2.0419	0.168264865	0.447323618
**NADH dehydrogenase [ubiquinone] 1 alpha subcomplex subunit 5 Ndufa5**	sp|Q63362|NDUA5_RAT	13 kDa	3.0462	3.1317	5.8232	10.021	7.6229	9.1887	0.01270423	0.447258186
**Protein RGD1559896 RGD1559896**	tr|D3ZY47|D3ZY47_RAT	36 kDa	3.0462	2.0878	1.9411	4.0083	5.7171	6.1258	0.016422536	0.446344756
**Metaxin 2 Mtx2**	tr|Q5U1Z9|Q5U1Z9_RAT	30 kDa	1	1	1.9411	3.0063	3.8114	2.0419	0.052316213	0.444839496
**Cytochrome c oxidase subunit 6C-2 Cox6c2**	sp|P11951|CX6C2_RAT	8 kDa	3.0462	4.1756	2.9116	8.0167	7.6229	7.1467	0.000875735	0.444714587
**Gamma-aminobutyric acid receptor-associated protein-like 2 Gabarapl2**	sp|P60522|GBRL2_RAT	14 kDa	1	1	1.9411	2.0042	3.8114	3.0629	0.054437873	0.443892549
**Propionyl-CoA carboxylase beta chain, mitochondrial Pccb**	sp|P07633|PCCB_RAT	59 kDa	1	6.2634	1.9411	8.0167	7.6229	5.1048	0.107409978	0.44371011
**Leucine zipper transcription factor-like protein 1 Lztfl1**	sp|Q562C6|LZTL1_RAT	35 kDa	1	1	1	2.0042	3.8114	1	0.196941018	0.440166676
**Protein Cpne6 Cpne6**	tr|D4ACG7|D4ACG7_RAT	62 kDa	3.0462	1	2.9116	7.0146	4.7643	4.0839	0.054858019	0.438623698

**Protein Pithd1 Pithd1**	tr|D4ABS5|D4ABS5_RAT	25 kDa	3.0462	1	2.9116	6.0125	4.7643	5.1048	0.01722682	0.438104473
**Protein Mrps36 Mrps36**	tr|M0R776|M0R776_RAT	11 kDa	2.0308	2.0878	1.9411	6.0125	4.7643	3.0629	0.038780544	0.437849086
**NADH dehydrogenase [ubiquinone] iron-sulfur protein 2, mitochondrial Ndufs2**	sp|Q641Y2|NDUS2_RAT	53 kDa	2.0308	2.0878	5.8232	8.0167	8.5757	6.1258	0.04314315	0.437613895
**HtrA serine peptidase 2 Htra2**	tr|B0BNB9|B0BNB9_RAT	49 kDa	1	1	1	3.0063	2.8586	1	0.116697092	0.437005637
**Protein Tstd3 Tstd3**	tr|G3V8W9|G3V8W9_RAT	18 kDa	1	1	1	3.0063	2.8586	1	0.116697092	0.437005637
**Protein Adam23 Adam23**	tr|D3ZT36|D3ZT36_RAT	91 kDa	1	1	1	2.0042	2.8586	2.0419	0.00952083	0.434486654
**Coiled-coil domain-containing protein 104 Ccdc104**	sp|Q4V8E4|CC104_RAT	40 kDa	1	1	1	2.0042	2.8586	2.0419	0.00952083	0.434486654
**1-acyl-sn-glycerol-3-phosphate acyltransferase delta Agpat4**	sp|Q924S1|PLCD_RAT	44 kDa	1	1	1	2.0042	2.8586	2.0419	0.00952083	0.434486654
**Liprin-alpha-3 Ppfia3**	tr|F1LSE6|F1LSE6_RAT	133 kDa	1	1	1	2.0042	2.8586	2.0419	0.00952083	0.434486654
**Protein Mblac2 Mblac2**	tr|D4A249|D4A249_RAT	31 kDa	1	1	1	2.0042	2.8586	2.0419	0.00952083	0.434486654
**Ubiquitin-like protein 4A Ubl4a**	sp|B2GV38|UBL4A_RAT	18 kDa	1	1	1	2.0042	2.8586	2.0419	0.00952083	0.434486654
**ZW10 interactor Zwint**	sp|Q8VIL3|ZWINT_RAT	30 kDa	1	1	1	2.0042	2.8586	2.0419	0.00952083	0.434486654
**Isoform 4 of G-protein-signaling modulator 1 Gpsm1**	sp|Q9R080-4|GPSM1_RAT (+2)	72 kDa	1	1	1	2.0042	2.8586	2.0419	0.00952083	0.434486654
**Vesicle-associated membrane protein 3 Vamp3**	sp|P63025|VAMP3_RAT	11 kDa	1	1	1	1	2.8586	3.0629	0.117194964	0.43343206
**Protein Ttc1 Ttc1**	tr|Q66H09|Q66H09_RAT	33 kDa	1	1	1	1	2.8586	3.0629	0.117194964	0.43343206
**LanC lantibiotic synthetase component C-like 2 (Bacterial) Lancl2**	tr|Q68FQ9|Q68FQ9_RAT	51 kDa	1	1	1	1	2.8586	3.0629	0.117194964	0.43343206
**Acyl carrier protein LOC683884**	tr|D3ZF13|D3ZF13_RAT	18 kDa	1	1	1	3.0063	1.9057	2.0419	0.019026655	0.431412589
**Leucine zipper protein 1 Luzp1**	sp|Q9ESV1|LUZP1_RAT (+1)	117 kDa	1	1	1	3.0063	1.9057	2.0419	0.019026655	0.431412589
**Peptidyl-prolyl cis-trans isomerase D Ppid**	sp|Q6DGG0|PPID_RAT	41 kDa	4.0616	4.1756	5.8232	10.021	12.387	10.21	0.002857719	0.431062603
**Amphiphysin Amph**	sp|O08838|AMPH_RAT (+1)	75 kDa	9.1386	7.3073	8.7347	20.042	20.01	18.377	0.000143573	0.430960653
**Protein RGD1560383 RGD1560383**	tr|D4AC62|D4AC62_RAT	34 kDa	1	1	1	2.0042	1.9057	3.0629	0.023268509	0.430243231
**Protein LOC682937 LOC682937**	tr|M0R7F3|M0R7F3_RAT	55 kDa	1	1	1	2.0042	1.9057	3.0629	0.023268509	0.430243231
**Glycogen synthase kinase-3 alpha Gsk3a**	sp|P18265|GSK3A_RAT (+1)	51 kDa	1	1	1	2.0042	1.9057	3.0629	0.023268509	0.430243231
**Malic enzyme Me3**	tr|F1M5N4|F1M5N4_RAT	67 kDa	1	1	1	2.0042	1.9057	3.0629	0.023268509	0.430243231
**Beta-soluble NSF attachment protein Napb**	tr|F8WFM2|F8WFM2_RAT	34 kDa	9.1386	9.3952	12.617	24.05	22.869	25.524	0.000532409	0.430004279
**Ras-related protein Rab-3A Rab3a**	sp|P63012|RAB3A_RAT	25 kDa	4.0616	1	3.8821	7.0146	6.67	7.1467	0.016758283	0.429339504
**ELAV-like protein 4 Elavl4**	sp|O09032|ELAV4_RAT (+2)	41 kDa	1	1	1	1	1.9057	4.0839	0.219841678	0.429209111
**Protein LOC500726 LOC500726**	tr|D4A1G8|D4A1G8_RAT	172 kDa	1	1	1	1	1.9057	4.0839	0.219841678	0.429209111
**Isoform 9 of Dynamin-3 Dnm3**	sp|Q08877-10|DYN3_RAT (+8)	97 kDa	8.1232	7.3073	15.528	26.054	21.916	24.503	0.00861704	0.427172878
**Anamorsin Ciapin1**	sp|Q5XID1|CPIN1_RAT	33 kDa	1	1	1	3.0063	1	3.0629	0.11619347	0.424376167
**Syntaxin-1B Stx1b**	sp|P61265|STX1B_RAT	33 kDa	4.0616	5.2195	5.8232	12.025	11.434	12.252	0.000274657	0.422959312
**Kinesin heavy chain isoform 5C (Fragment) Kif5c**	tr|G3V6L4|G3V6L4_RAT	104 kDa	6.0924	5.2195	7.7642	17.035	15.246	13.273	0.002588609	0.418757958
**Protein LOC361414 LOC361414**	tr|D3ZE32|D3ZE32_RAT	46 kDa	3.0462	2.0878	6.7937	9.0188	9.5286	10.21	0.019120533	0.414769764
**Adaptin ear-binding coat-associated protein 1 Necap1**	sp|P69682|NECP1_RAT	30 kDa	3.0462	1	2.9116	5.0104	6.67	5.1048	0.018397373	0.414519934
**Dynamin-1 Dnm1**	sp|P21575|DYN1_RAT	97 kDa	21.323	24.01	31.057	58.121	61.936	64.321	0.000459892	0.414311903
**Protein Stxbp6 Stxbp6**	tr|D3ZCI5|D3ZCI5_RAT	24 kDa	2.0308	1	1	4.0083	4.7643	1	0.185929487	0.412459325
**Protein Ppa2 Ppa2**	tr|D4A830|D4A830_RAT	38 kDa	2.0308	1	1	3.0063	4.7643	2.0419	0.090543908	0.410782166
**NADH dehydrogenase (Ubiquinone) Fe-S protein 5 Ndufs5**	tr|B5DEL8|B5DEL8_RAT	13 kDa	2.0308	4.1756	1.9411	6.0125	5.7171	8.1677	0.021119686	0.409477668
**Importin subunit beta-1 Kpnb1**	tr|F2Z3Q8|F2Z3Q8_RAT (+1)	97 kDa	3.0462	3.1317	3.8821	6.0125	10.481	8.1677	0.020929473	0.407928244
**Citrate synthase Cs**	tr|G3V936|G3V936_RAT	52 kDa	5.077	3.1317	5.8232	12.025	11.434	11.231	0.001189309	0.404494091
**Arylsulfatase A Arsa**	tr|Q32KK2|Q32KK2_RAT	54 kDa	1	1	1.9411	3.0063	3.8114	3.0629	0.008258989	0.398872538
**Calretinin Calb2**	sp|P47728|CALB2_RAT	31 kDa	6.0924	3.1317	7.7642	15.031	14.293	13.273	0.004132311	0.398814471
**Aldehyde dehydrogenase X, mitochondrial Aldh1b1**	sp|Q66HF8|AL1B1_RAT (+1)	58 kDa	1	1	1.9411	2.0042	3.8114	4.0839	0.051742724	0.398111016
**Protein Rnf14 Rnf14**	tr|Q3ZAU6|Q3ZAU6_RAT	54 kDa	1	1	1.9411	3.0063	2.8586	4.0839	0.015799118	0.396138228
**Isoform 7 of Formin-binding protein 1 Fnbp1**	sp|Q8R511-7|FNBP1_RAT (+7)	65 kDa	3.0462	1	1	5.0104	3.8114	4.0839	0.027505296	0.391005525
**Isoform 6 of Disks large homolog 2 Dlg2**	sp|Q63622-6|DLG2_RAT (+4)	93 kDa	2.0308	1	1.9411	4.0083	5.7171	3.0629	0.036648438	0.388785061
**gi|1363944|pir||JC4313 type I keratin 16 - human gi|1195531|bbs|172338**	zz|ZZ_FGCZCont0188|	51 kDa	6.0924	1	4.8526	20.042	3.8114	7.1467	0.287448037	0.385321338
**Contactin 2 Cntn2**	tr|G3V758|G3V758_RAT	113 kDa	4.0616	1	4.8526	10.021	8.5757	7.1467	0.021417038	0.385116185
**V-type proton ATPase subunit F Atp6v1f**	sp|P50408|VATF_RAT	13 kDa	2.0308	4.1756	2.9116	10.021	8.5757	5.1048	0.037475429	0.38470139
**Protein Sptbn4 Sptbn4**	tr|F1LSL8|F1LSL8_RAT	289 kDa	1	1	1	2.0042	3.8114	2.0419	0.053220631	0.381800827
**Isoform 2 of Stathmin-4 Stmn4**	sp|P63043-2|STMN4_RAT (+3)	20 kDa	1	1	1	4.0083	2.8586	1	0.137821804	0.381344621
**Kinesin heavy chain isoform 5C (Fragment) Kif5c**	sp|P56536|KIF5C_RAT	27 kDa	1	1	1	1	3.8114	3.0629	0.125410573	0.380986246
**Stathmin-2 Stmn2**	sp|P21818|STMN2_RAT	21 kDa	1	1	1	1	3.8114	3.0629	0.125410573	0.380986246
**Protein Kif21a (Fragment) Kif21a**	tr|D3ZYN2|D3ZYN2_RAT	179 kDa	8.1232	5.2195	7.7642	16.033	20.01	19.398	0.001739659	0.380709222
**Isoform 2 of WD repeat-containing protein 7 Wdr7**	sp|Q9ERH3-2|WDR7_RAT (+1)	160 kDa	1	1	1	3.0063	2.8586	2.0419	0.005492378	0.379420246
**Peptidyl-prolyl cis-trans isomerase F, mitochondrial Ppif**	sp|P29117|PPIF_RAT	22 kDa	1	1	1	3.0063	2.8586	2.0419	0.005492378	0.379420246
**Eukaryotic translation initiation factor 6 Eif6**	sp|Q3KRD8|IF6_RAT	27 kDa	1	1	1	3.0063	2.8586	2.0419	0.005492378	0.379420246
**Mevalonate kinase Mvk**	sp|P17256|KIME_RAT	42 kDa	1	1	1	3.0063	2.8586	2.0419	0.005492378	0.379420246
**Fatty acid-binding protein, heart Fabp3**	sp|P07483|FABPH_RAT	15 kDa	1	1	1	3.0063	2.8586	2.0419	0.005492378	0.379420246
**Diphosphomevalonate decarboxylase Mvd**	sp|Q62967|MVD1_RAT (+1)	44 kDa	1	1	1	2.0042	2.8586	3.0629	0.007162678	0.378515462
**Protein Otud7b Otud7b**	tr|D3ZH40|D3ZH40_RAT	92 kDa	1	1	1	2.0042	2.8586	3.0629	0.007162678	0.378515462
**gi|71536|pir|| KRHU2 keratin, 67K type II cytoskeletal - human (fragme**	zz|ZZ_FGCZCont0050|	53 kDa	1	1	14.558	21.044	10.481	12.252	0.179033622	0.378235146
**Mitochondrial fission regulator 1-like Mtfr1l**	sp|Q5XII9|MFR1L_RAT	32 kDa	1	1	1	1	2.8586	4.0839	0.13996452	0.377714825
**Protein Snx4 Snx4**	tr|E9PU13|E9PU13_RAT	52 kDa	1	1	1	1	2.8586	4.0839	0.13996452	0.377714825
**Protein FAM162A Fam162a**	sp|Q4QQV3|F162A_RAT	18 kDa	1	1	1	4.0083	1.9057	2.0419	0.071837595	0.377078646
**Coiled-coil and C2 domain-containing protein 1B Cc2d1b**	sp|Q5FVK6|C2D1B_RAT	94 kDa	1	1	1	4.0083	1.9057	2.0419	0.071837595	0.377078646
**Protein Vta1 Vta1**	tr|Q4KM55|Q4KM55_RAT	27 kDa	1	1	1	3.0063	1.9057	3.0629	0.011666577	0.376180266
**Protein Stard10 Stard10**	tr|Q5BJN1|Q5BJN1_RAT	33 kDa	1	1	1	3.0063	1.9057	3.0629	0.011666577	0.376180266
**Leucine-rich repeat LGI family, member 3 (Predicted), isoform CRA_b Lgi3**	tr|D3ZN61|D3ZN61_RAT	62 kDa	3.0462	2.0878	4.8526	10.021	7.6229	9.1887	0.006367738	0.3721816
**Epb4.9 protein Epb4.9**	tr|B2GUY4|B2GUY4_RAT (+1)	40 kDa	2.0308	1	1	3.0063	4.7643	3.0629	0.027851071	0.372068122
**Mthfd1l protein Mthfd1l**	tr|B2GUZ3|B2GUZ3_RAT	106 kDa	2.0308	1	1	4.0083	3.8114	3.0629	0.007010005	0.370389429
**Isoform 2 of SRC kinase signaling inhibitor 1 Srcin1**	sp|Q9QXY2-2|SRCN1_RAT (+1)	127 kDa	3.0462	1	5.8232	8.0167	8.5757	10.21	0.021699765	0.368228218
**Dual specificity mitogen-activated protein kinase kinase 1 Map2k1**	sp|Q01986|MP2K1_RAT	43 kDa	3.0462	2.0878	3.8821	7.0146	8.5757	9.1887	0.003175798	0.363860527
**Protein LOC684112 LOC684112**	tr|M0RD40|M0RD40_RAT	151 kDa	1	1	1.9411	2.0042	4.7643	4.0839	0.060313329	0.363154694
**Adenylosuccinate synthetase Adss**	tr|D4AEP0|D4AEP0_RAT	50 kDa	1	1	1.9411	3.0063	3.8114	4.0839	0.006749746	0.361515741
**Tumor protein p63-regulated gene 1-like protein Tprg1l**	sp|A8WCF8|TPRGL_RAT	30 kDa	3.0462	1	1	3.0063	3.8114	7.1467	0.107792566	0.361361748
**Nuclear migration protein nudC Nudc**	sp|Q63525|NUDC_RAT (+1)	38 kDa	3.0462	2.0878	1.9411	6.0125	8.5757	5.1048	0.018470664	0.359269791
**AH receptor-interacting protein Aip**	sp|Q5FWY5|AIP_RAT	38 kDa	1	1	1.9411	4.0083	1.9057	5.1048	0.075664288	0.357670527
**Isoform 2 of Vesicle-associated membrane protein 1 Vamp1**	sp|Q63666-2|VAMP1_RAT (+4)	13 kDa	1	1	1.9411	4.0083	1.9057	5.1048	0.075664288	0.357670527
**COP9 signalosome complex subunit 2 Cops2**	sp|P61203|CSN2_RAT	52 kDa	1	2.0878	2.9116	5.0104	6.67	5.1048	0.009612804	0.357422015
**COP9 (Constitutive photomorphogenic) homolog, subunit 5 (Arabidopsis thaliana) Cops5**	tr|Q4KM69|Q4KM69_RAT	38 kDa	1	3.1317	2.9116	8.0167	7.6229	4.0839	0.041040159	0.357101934
**Protein RGD1559864 RGD1559864**	tr|D3ZBS6|D3ZBS6_RAT (+1)	45 kDa	1	1	2.9116	2.0042	5.7171	6.1258	0.110532126	0.354702429
**Rabphilin-3A Rph3a**	tr|F1LPB9|F1LPB9_RAT (+1)	76 kDa	1	1	6.7937	7.0146	7.6229	10.21	0.068860471	0.353906832
**Protein Omg Omg**	tr|F7EYB9|F7EYB9_RAT	50 kDa	1	1	2.9116	5.0104	3.8114	5.1048	0.016845387	0.35267761
**Unconventional myosin-Id Myo1d**	sp|Q63357|MYO1D_RAT	116 kDa	7.1078	3.1317	9.7053	14.029	18.104	24.503	0.027297848	0.352157638
**Protein Anln1 (Fragment) Anln1**	tr|M0R9N8|M0R9N8_RAT	107 kDa	4.0616	3.1317	7.7642	15.031	15.246	12.252	0.005815997	0.351701192
**Pabpc4 protein Pabpc4**	tr|G3V9N0|G3V9N0_RAT	71 kDa	1	1	1	1	6.67	1	0.373900966	0.346020761
**Visinin-like protein 1 Vsnl1**	sp|P62762|VISL1_RAT	22 kDa	4.0616	2.0878	4.8526	9.0188	9.5286	13.273	0.011588948	0.345753039
**Inactive phospholipase C-like protein 1 Plcl1**	sp|Q62688|PLCL1_RAT	123 kDa	3.0462	2.0878	1.9411	8.0167	8.5757	4.0839	0.03568019	0.342184046
**Leukotriene A-4 hydrolase Lta4h**	tr|Q499P2|Q499P2_RAT	69 kDa	1	1	1	4.0083	3.8114	1	0.116571628	0.340147624
**Inositol monophosphatase 1 Impa1**	tr|F1M978|F1M978_RAT	30 kDa	1	2.0878	1.9411	5.0104	5.7171	4.0839	0.005010704	0.339529011
**Influenza virus NS1A binding protein (Predicted), isoform CRA_a Ivns1abp**	tr|Q0IKU7|Q0IKU7_RAT	33 kDa	1	1	1	3.0063	3.8114	2.0419	0.018801311	0.338615739
**Oxysterol-binding protein-related protein 1 Osbpl1a**	sp|Q8K4M9|OSBL1_RAT (+1)	108 kDa	1	2.0878	1.9411	5.0104	4.7643	5.1048	0.000764433	0.337975066
**Protein Dmxl2-ps1 (Fragment) Dmxl2-ps1**	tr|F1M164|F1M164_RAT (+1)	248 kDa	1	1	1	2.0042	3.8114	3.0629	0.020160881	0.337894915
**RAP1, GTPase activating protein 1, isoform CRA_a Rap1ga1**	tr|F1LV89|F1LV89_RAT	81 kDa	1	1	1	4.0083	2.8586	2.0419	0.025976715	0.33674569
**Serine/threonine-protein phosphatase 5 Ppp5c**	sp|P53042|PPP5_RAT	57 kDa	1	1	1	4.0083	2.8586	2.0419	0.025976715	0.33674569
**Uncharacterized protein (Fragment)**	tr|M0RBJ0|M0RBJ0_RAT	7 kDa	1	1	1	3.0063	2.8586	3.0629	5.37985E-06	0.336029033
**Protein Grcc10 LOC100911713**	tr|D3ZMS0|D3ZMS0_RAT	13 kDa	1	1	1	3.0063	2.8586	3.0629	5.37985E-06	0.336029033
**Adaptor-related protein complex 3, delta 1 subunit, isoform CRA_b Ap3d1**	tr|B5DFK6|B5DFK6_RAT	136 kDa	1	1	1	3.0063	2.8586	3.0629	5.37985E-06	0.336029033
**Protein Irgq Irgq**	tr|M0R686|M0R686_RAT	59 kDa	1	1	1	3.0063	2.8586	3.0629	5.37985E-06	0.336029033
**PEX5-related protein Pex5l**	tr|F1LMT5|F1LMT5_RAT (+1)	66 kDa	1	1	1	3.0063	2.8586	3.0629	5.37985E-06	0.336029033
**Acyl-CoA synthetase short-chain family member 2 (Predicted) Acss2**	tr|G3V9U0|G3V9U0_RAT	79 kDa	1	1	1	2.0042	2.8586	4.0839	0.030375158	0.335319168
**Alanine--tRNA ligase, cytoplasmic Aars**	sp|P50475|SYAC_RAT	107 kDa	7.1078	4.1756	12.617	22.046	24.774	24.503	0.00381555	0.335100879
**Calbindin Calb1**	sp|P07171|CALB1_RAT	30 kDa	1	1	1	4.0083	1.9057	3.0629	0.030591487	0.33419109
**Isoform 2 of Limbic system-associated membrane protein Lsamp**	sp|Q62813-2|LSAMP_RAT (+1)	40 kDa	2.0308	1	1.9411	5.0104	4.7643	5.1048	0.000664942	0.334144292
**Low molecular weight phosphotyrosine protein phosphatase Acp1**	sp|P41498|PPAC_RAT	18 kDa	3.0462	1	3.8821	10.021	7.6229	6.1258	0.020555435	0.33354649
**Synaptotagmin-2 Syt2**	sp|P29101|SYT2_RAT (+1)	47 kDa	1	1	2.9116	5.0104	6.67	3.0629	0.055086265	0.333141156
**gi|547751|sp|Q04695| K1CQ_HUMAN KERATIN, TYPE I CYTOSKELETAL 17 (CYTOK**	zz|ZZ_FGCZCont0083|	48 kDa	1	1	1	6.0125	1	2.0419	0.256836539	0.331330624
**Monoglyceride lipase Mgll**	sp|Q8R431|MGLL_RAT	33 kDa	1	1	1.9411	4.0083	2.8586	5.1048	0.020547101	0.329201367
**Acylamino-acid-releasing enzyme Apeh**	sp|P13676|ACPH_RAT	81 kDa	1	3.1317	1	6.0125	5.7171	4.0839	0.018628995	0.324513865
**Isoform Syn2 of Alpha-synuclein Snca**	sp|P37377-2|SYUA_RAT (+1)	16 kDa	3.0462	1	3.8821	8.0167	8.5757	8.1677	0.003004008	0.320204684
**Acetyl-CoA acetyltransferase, cytosolic Acat2**	sp|Q5XI22|THIC_RAT	41 kDa	6.0924	5.2195	6.7937	19.04	19.057	19.398	9.77012E-06	0.314907383
**Plastin-3 Pls3**	sp|Q63598|PLST_RAT (+1)	71 kDa	2.0308	3.1317	3.8821	9.0188	9.5286	10.21	0.000503809	0.314513829
**Beta-adducin Add2**	sp|Q05764|ADDB_RAT (+1)	81 kDa	3.0462	4.1756	6.7937	15.031	14.293	15.314	0.000892561	0.313981361
**DnaJ (Hsp40) homolog, subfamily C, member 6 (Predicted) Dnajc6**	tr|D4A0I5|D4A0I5_RAT	99 kDa	1	1	3.8821	6.0125	5.7171	7.1467	0.014780306	0.311612975
**LanC-like protein 1 Lancl1**	sp|Q9QX69|LANC1_RAT	45 kDa	1	1	1.9411	4.0083	5.7171	3.0629	0.024436048	0.308180133
**Isoform 2 of Prolyl endopeptidase-like Prepl**	sp|Q5HZA6-2|PPCEL_RAT (+2)	73 kDa	1	1	1.9411	5.0104	4.7643	3.0629	0.012540942	0.306996635
**Mitogen-activated protein kinase 10 Mapk10**	sp|P49187|MK10_RAT (+1)	53 kDa	1	1	1.9411	4.0083	4.7643	4.0839	0.001674432	0.306545327
**Cdc42 effector protein 2 Cdc42ep2**	sp|Q5PQP4|BORG1_RAT	23 kDa	1	1	1	5.0104	3.8114	1	0.128256373	0.305442994
**Acad9 protein Acad9**	tr|B1WC61|B1WC61_RAT	69 kDa	1	1	1	2.0042	4.7643	3.0629	0.047226808	0.30514474
**NADH dehydrogenase (Ubiquinone) 1 beta subcomplex, 6 (Predicted) Ndufb6**	tr|D3ZZ21|D3ZZ21_RAT	16 kDa	1	1	1	1	4.7643	4.0839	0.120028115	0.304624195
**Protein Arhgap44 Arhgap44**	tr|F1LST2|F1LST2_RAT	88 kDa	1	1	1	4.0083	3.8114	2.0419	0.021616072	0.30421027

**Dynactin 6 (Predicted), isoform CRA_b Dctn6**	tr|D4ADD8|D4ADD8_RAT	21 kDa	1	1	1	4.0083	3.8114	2.0419	0.021616072	0.30421027
**Cyclin-dependent kinase 5 Cdk5**	sp|Q03114|CDK5_RAT	33 kDa	1	1	1	3.0063	3.8114	3.0629	0.000903991	0.303625286
**Serine/threonine-protein kinase BRSK2 Brsk2**	sp|D3ZML2|BRSK2_RAT	81 kDa	1	1	1	2.0042	3.8114	4.0839	0.02435665	0.303045608
**Pyridoxal phosphate phosphatase Pdxp**	sp|Q8VD52|PLPP_RAT (+2)	33 kDa	1	1	1	4.0083	2.8586	3.0629	0.00285259	0.302120889
**Isoform 2 of GRIP1-associated protein 1 Gripap1**	sp|Q9JHZ4-2|GRAP1_RAT (+2)	91 kDa	1	1	1	2.0042	2.8586	5.1048	0.065928162	0.30097516
**Calpain-2 catalytic subunit Capn2**	sp|Q07009|CAN2_RAT	80 kDa	3.0462	2.0878	9.7053	17.035	17.151	15.314	0.009438228	0.299783838
**Protein LOC686590 LOC686590**	tr|M0R4G7|M0R4G7_RAT (+2)	108 kDa	3.0462	1	1	6.0125	5.7171	5.1048	0.005831354	0.299755263
**Syntaphilin Snph**	sp|B5DF41|SNPH_RAT	54 kDa	1	1	1	3.0063	1.9057	5.1048	0.067298189	0.299496845
**1-phosphatidylinositol 4,5-bisphosphate phosphodiesterase beta-1 Plcb1**	sp|P10687|PLCB1_RAT	138 kDa	1	1	1	3.0063	1.9057	5.1048	0.067298189	0.299496845
**Isoform 2 of Neuronal-specific septin-3 Sept3**	sp|Q9WU34-2|SEPT3_RAT (+3)	40 kDa	1	1	3.8821	8.0167	6.67	5.1048	0.022148215	0.297203345
**Caskin-1 Caskin1**	sp|Q8VHK2|CSKI1_RAT (+1)	150 kDa	4.0616	2.0878	3.8821	9.0188	11.434	14.293	0.007519776	0.288711154
**Leucine-rich PPR motif-containing protein, mitochondrial Lrpprc**	sp|Q5SGE0|LPPRC_RAT (+1)	157 kDa	1	1	1.9411	4.0083	4.7643	5.1048	0.001830459	0.28399412
**Uncharacterized protein Anln**	tr|D4A0V6|D4A0V6_RAT	123 kDa	1	1	8.7347	13.027	13.34	12.252	0.023228536	0.277964215
**Protein RGD1307235 RGD1307235**	tr|D3ZJ01|D3ZJ01_RAT	135 kDa	1	1	1	3.0063	4.7643	3.0629	0.010601538	0.276918817
**Serine/threonine-protein phosphatase 2B catalytic subunit beta isoform Ppp3cb**	sp|P20651|PP2BB_RAT (+1)	59 kDa	1	1	2.9116	7.0146	5.7171	5.1048	0.007146412	0.275367925
**Neurosecretory protein VGF Vgf**	sp|P20156|VGF_RAT (+1)	68 kDa	1	1	1	3.0063	3.8114	4.0839	0.001238163	0.275188963
**ATP synthase subunit e, mitochondrial Atp5i**	sp|P29419|ATP5I_RAT	8 kDa	1	2.0878	1	6.0125	4.7643	4.0839	0.005882624	0.275074525
**Isoform 2 of Hepatocyte growth factor-regulated tyrosine kinase substrate Hgs**	sp|Q9JJ50-2|HGS_RAT (+1)	86 kDa	1	1	1	4.0083	2.8586	4.0839	0.002603287	0.273952588
**Protein RGD1304884 RGD1304884**	tr|D4A3C2|D4A3C2_RAT	46 kDa	1	1	1	4.0083	2.8586	4.0839	0.002603287	0.273952588
**NCK interacting protein with SH3 domain (Predicted), isoform CRA_a Nckipsd**	tr|D3ZWX4|D3ZWX4_RAT	55 kDa	1	1	1	4.0083	2.8586	4.0839	0.002603287	0.273952588
**Isoform 2 of Protein NDRG4 Ndrg4**	sp|Q9Z2L9-2|NDRG4_RAT (+4)	42 kDa	1	1	1	3.0063	2.8586	5.1048	0.021535553	0.273480587
**Isoform 2 of Aggrecan core protein Acan**	sp|P07897-2|PGCA_RAT (+3)	225 kDa	2.0308	1	1	6.0125	4.7643	4.0839	0.005466497	0.271238905
**ATP citrate lyase, isoform CRA_a Acly**	tr|G3V888|G3V888_RAT (+1)	121 kDa	5.077	7.3073	10.676	31.065	28.586	25.524	0.000820665	0.270740241
**Isoform L of Band 4.1-like protein 1 Epb41l1**	sp|Q9WTP0-2|E41L1_RAT	171 kDa	4.0616	4.1756	7.7642	21.044	20.963	17.356	0.001096213	0.269551741
**Synaptojanin-1 Synj1**	sp|Q62910|SYNJ1_RAT (+2)	173 kDa	6.0924	3.1317	9.7053	24.05	21.916	24.503	0.001132611	0.268620244
**Isoform 2 of Clathrin coat assembly protein AP180 Snap91**	sp|Q05140-2|AP180_RAT	91 kDa	1	3.1317	4.8526	13.027	13.34	7.1467	0.023756249	0.268078428
**Ankyrin 1, erythroid Ank1**	tr|D3Z9Z0|D3Z9Z0_RAT	187 kDa	3.0462	2.0878	5.8232	13.027	15.246	13.273	0.001521267	0.263736581
**Protein Npepps Npepps**	tr|F1M9V7|F1M9V7_RAT	103 kDa	2.0308	1	7.7642	16.033	12.387	13.273	0.012255891	0.258916365
**Complexin-1 Cplx1**	sp|P63041|CPLX1_RAT	15 kDa	1	1	3.8821	7.0146	7.6229	8.1677	0.00516388	0.257928016
**Farnesyl pyrophosphate synthase Fdps**	sp|P05369|FPPS_RAT (+1)	41 kDa	1	1	1	2.0042	5.7171	4.0839	0.052345013	0.254125301
**Isoform Cytoplasmic of Phospholipid hydroperoxide glutathione peroxidase, mitochondrial Gpx4**	sp|P36970-2|GPX41_RAT (+3)	19 kDa	1	2.0878	1.9411	7.0146	6.67	6.1258	0.000324714	0.253851512
**Protein Ppa1 Ppa1**	tr|F7EPH4|F7EPH4_RAT	33 kDa	3.0462	2.0878	1.9411	9.0188	7.6229	11.231	0.003306912	0.253836191
**Protein Kif1a LOC100362928**	tr|F1M4A4|F1M4A4_RAT	192 kDa	1	1	1	3.0063	4.7643	4.0839	0.004486922	0.253068455
**Protein Map7d2 Map7d2**	tr|D4A1J8|D4A1J8_RAT (+1)	66 kDa	1	1	1	5.0104	3.8114	3.0629	0.00642086	0.252425387
**NAD-dependent protein deacylase sirtuin-5, mitochondrial Sirt5**	sp|Q68FX9|SIR5_RAT	34 kDa	1	1	1	2.0042	3.8114	6.1258	0.066858731	0.251226824
**Heme binding protein 2 (Predicted), isoform CRA_b Hebp2**	tr|D3ZHC4|D3ZHC4_RAT	23 kDa	1	1	1	5.0104	2.8586	4.0839	0.008718275	0.250985117
**Eukaryotic translation initiation factor 4H Eif4h**	sp|Q5XI72|IF4H_RAT	27 kDa	2.0308	1	1.9411	9.0188	5.7171	5.1048	0.017027395	0.250590957
**Leucine-rich glioma-inactivated protein 1 Lgi1**	sp|Q8K4Y5|LGI1_RAT	64 kDa	1	1	1.9411	6.0125	4.7643	5.1048	0.001220518	0.248155098
**Hyaluronan and proteoglycan link protein 1 Hapln1**	sp|P03994|HPLN1_RAT	40 kDa	1	1	2.9116	7.0146	6.67	6.1258	0.001950313	0.24793038
**Branched-chain-amino-acid aminotransferase, cytosolic Bcat1**	sp|P54690|BCAT1_RAT	46 kDa	1	1	3.8821	11.023	6.67	7.1467	0.019757623	0.236802377
**Cytoplasmic dynein 1 intermediate chain 1 Dync1i1**	tr|G3V792|G3V792_RAT	61 kDa	1	1	3.8821	10.021	5.7171	9.1887	0.017656184	0.235974935
**Protein Rap1gds1 Rap1gds1**	tr|F1M7Y3|F1M7Y3_RAT	52 kDa	3.0462	2.0878	4.8526	14.029	13.34	15.314	0.000395819	0.23397137
**Protein Ube2o (Fragment) Ube2o**	tr|F1M403|F1M403_RAT	141 kDa	1	1	1	3.0063	4.7643	5.1048	0.007167591	0.23300247
**Protein LOC100912599 LOC679739**	tr|D3ZCZ9|D3ZCZ9_RAT	13 kDa	1	1	1	6.0125	3.8114	3.0629	0.020422933	0.23279635
**gi|1346349|sp|P48669| K2CF_HUMAN KERATIN, TYPE II CYTOSKELETAL 6F (CYT**	zz|ZZ_FGCZCont0233|	60 kDa	1	1	1	11.023	1	1	0.373900966	0.230361668
**Fatty acid synthase Fasn**	sp|P12785|FAS_RAT	273 kDa	9.1386	6.2634	19.411	54.113	58.124	44.922	0.001870658	0.221514517
**AP2-associated protein kinase 1 Aak1**	tr|F1LRI7|F1LRI7_RAT	104 kDa	4.0616	1	6.7937	20.042	17.151	16.335	0.002326454	0.221478479
**Cyclin-dependent kinase 18 Cdk18**	sp|O35832|CKD18_RAT	52 kDa	1	1	1.9411	6.0125	5.7171	6.1258	0.000160607	0.220723143
**Hyaluronan and proteoglycan link protein 4 (Predicted) Hapln4**	tr|D3Z9H2|D3Z9H2_RAT	43 kDa	1	1	1	4.0083	5.7171	4.0839	0.002950494	0.2172449
**Ras-related protein Rab-3C Rab3c**	sp|P62824|RAB3C_RAT	26 kDa	1	1	1	4.0083	3.8114	6.1258	0.007901869	0.215123158
**Isoform IIb of Synapsin-2 Syn2**	sp|Q63537-2|SYN2_RAT	52 kDa	1	1	13.587	25.052	23.821	24.503	0.010224178	0.212426406
**Protein Map2k4 (Fragment) Map2k4**	tr|F1LP57|F1LP57_RAT	41 kDa	1	1	1	6.0125	6.67	2.0419	0.05390362	0.203743446
**Protein Ap3b2 Ap3b2**	tr|D4AE00|D4AE00_RAT	119 kDa	1	1	1	6.0125	4.7643	4.0839	0.002191099	0.201874743
**Unconventional myosin-Va Myo5a**	sp|Q9QYF3|MYO5A_RAT	212 kDa	2.0308	1	4.8526	9.0188	14.293	16.335	0.012695055	0.198840764
**RNA-binding protein Nova-1 (Fragment) Nova1**	sp|Q80WA4|NOVA1_RAT (+1)	48 kDa	1	1	1.9411	8.0167	5.7171	6.1258	0.002377779	0.198448106
**Protein Pgp Pgp**	tr|D3ZDK7|D3ZDK7_RAT	35 kDa	1	1	2.9116	9.0188	7.6229	8.1677	0.000929017	0.197973349
**Oxidation resistance protein 1 Oxr1**	sp|Q4V8B0|OXR1_RAT	93 kDa	1	1	3.8821	11.023	9.5286	9.1887	0.002034361	0.197782134
**Kinesin light chain 2 (Predicted), isoform CRA_b Klc2**	tr|B2GV74|B2GV74_RAT	69 kDa	1	1	4.8526	12.025	11.434	11.231	0.002073631	0.197538195
**Uncharacterized protein (Fragment)**	tr|F1LTQ2|F1LTQ2_RAT	34 kDa	1	1	1	1	13.34	1	0.373900966	0.195567145
**Neurochondrin Ncdn**	sp|O35095|NCDN_RAT	79 kDa	3.0462	1	4.8526	13.027	20.963	12.252	0.014232691	0.192439773
**SH3-containing GRB2-like protein 3-interacting protein 1 Sgip1**	sp|P0DJJ3|SGIP1_RAT	89 kDa	1	1	1	3.0063	8.5757	4.0839	0.068538484	0.191498733
**Spectrin beta chain, non-erythrocytic 2 Sptbn2**	sp|Q9QWN8|SPTN2_RAT (+1)	271 kDa	1	3.1317	1	11.023	5.7171	10.21	0.015494813	0.190414878
**Protein IMPACT Impact**	sp|Q5GFD9|IMPCT_RAT	36 kDa	1	1	2.9116	7.0146	8.5757	11.231	0.006213919	0.183123115
**Carboxypeptidase M (Predicted) Cpm**	tr|D4A9Q5|D4A9Q5_RAT	50 kDa	1	1	1.9411	6.0125	9.5286	6.1258	0.007803698	0.181894964
**Protein Rab6b Rab6b**	tr|F1LVC3|F1LVC3_RAT	20 kDa	1	1	1	5.0104	5.7171	6.1258	0.000143857	0.178006681
**Protein LOC688905 LOC688905**	tr|D3ZNK1|D3ZNK1_RAT	35 kDa	1	1	1	7.0146	5.7171	5.1048	0.000926337	0.168194433
**Glutaminase kidney isoform, mitochondrial Gls**	sp|P13264|GLSK_RAT	74 kDa	1	1	3.8821	13.027	12.387	11.231	0.000724354	0.160515759
**Uncharacterized protein**	tr|M0RBT1|M0RBT1_RAT	54 kDa	1	1	1	18.038	1	1	0.373900966	0.14971554
**Microtubule-associated protein Mapt**	tr|D3ZRX4|D3ZRX4_RAT	52 kDa	1	1	1	1	1	20.419	0.373900966	0.133815068
**Calcium-dependent secretion activator 1 Cadps**	sp|Q62717|CAPS1_RAT	146 kDa	1	1	1	8.0167	7.6229	7.1467	1.25686E-05	0.131658058
**Isoform 2 of Cytoplasmic dynein 1 intermediate chain 1 Dync1i1**	sp|Q63100-3|DC1I1_RAT (+1)	71 kDa	1	1	1	9.0188	4.7643	9.1887	0.01003288	0.130594903
**Isoform 3 of Tropomyosin alpha-3 chain Tpm3**	sp|Q63610-3|TPM3_RAT	29 kDa	1	1	1	21.044	1	1	0.373900966	0.130185732
**Kinesin heavy chain isoform 5A Kif5a**	tr|F1M8F2|F1M8F2_RAT	117 kDa	1	1	1	8.0167	8.5757	8.1677	1.67835E-06	0.121162677
**Clathrin coat assembly protein AP180 Snap91**	tr|F1LRK0|F1LRK0_RAT	94 kDa	1	1	1	12.025	13.34	1	0.117374896	0.113787218
**Serine/threonine-protein kinase PAK 3 Pak3**	sp|Q62829|PAK3_RAT	61 kDa	1	1	1	9.0188	6.67	11.231	0.003755128	0.111442136
**Oxoglutarate dehydrogenase-like (Predicted), isoform CRA_a Ogdhl**	tr|D3ZQD3|D3ZQD3_RAT	117 kDa	1	1	1	5.0104	12.387	10.21	0.019973618	0.108666517
**Hydroxymethylglutaryl-CoA synthase, cytoplasmic Hmgcs1**	sp|P17425|HMCS1_RAT	57 kDa	1	1	1	11.023	10.481	11.231	1.54731E-06	0.091645028
**Glyceraldehyde-3-phosphate dehydrogenase RGD1564688**	tr|E9PTN6|E9PTN6_RAT	36 kDa	1	1	1	23.048	25.727	1	0.117419656	0.06027122
**Guanine nucleotide-binding protein G(o) subunit alpha Gnao1**	sp|P59215|GNAO_RAT	40 kDa	1	1	1	14.029	16.199	20.419	0.001066794	0.059233518
**Isoform S-MAG of Myelin-associated glycoprotein Mag**	sp|P07722-2|MAG_RAT (+1)	64 kDa	1	1	1	20.042	25.727	17.356	0.001249929	0.047524752

Supplementary Table S2

Protein Name	Short Name	Control1	Control2	Control3	Injured1	Injured2	Injured3	t-test p value	Corrected p value q=0.0288	Average spectral counts	Molecular Mass (kDa)	Average spectral counts/ Molecular Mass
Aggrecan	Acan	6	5	4	2	0	0	0.0083	0.0196	5.7	225	0.03
Agrin	Agrn	3	3	4	8	9	5	0.0421	0.0326	10.9	217	0.05
Asporin	Aspn	5	0	3	26	31	10	0.0386	0.0304	24.8	43	0.58
Biglycan	Bgn	16	13	14	33	32	26	0.0031	0.0120	44.8	42	1.07
Brevican	Bcan	23	24	20	12	8	13	0.0046	0.0152	33.5	96	0.35
Clusterin	Clu	6	7	4	12	13	12	0.0013	0.0087	17.9	51	0.35
Collagen alpha-1(I)	Col1a1	18	14	12	28	40	28	0.0204	0.0250	46.8	138	0.34
Collagen alpha-1(III)	Col3a1	0	0	0	2	3	0	0.1338	0.0435	1.7	139	0.01
Collagen alpha-1(V)	Col5a1	0	0	0	2	3	2	0.0034	0.0130	2.4	162	0.01
Collagen alpha-1(VI)	Col6a1	18	17	21	25	31	18	0.2234	0.0457	43.1	109	0.40
Collagen alpha-1(XII)	Col12a1	9	9	10	16	18	12	0.0349	0.0293	24.8	210	0.12
Collagen alpha-1(XV)	Col15a1	3	3	3	5	9	6	0.0465	0.0337	9.9	141	0.07
Collagen alpha-1(XVIII)	Col18a1	0	0	0	5	7	5	0.0018	0.0098	5.8	135	0.04
Collagen alpha-1(XXV)	Col25a1	0	2	0	2	0	2	0.4943	0.0489	2.0	65	0.03
Collagen alpha-2(I)	Col1a2	9	8	9	17	19	18	0.0002	0.0022	26.8	130	0.21
Collagen alpha-2(IV)	Col4a2	0	0	0	0	2	2	0.1166	0.0424	1.4	166	0.01
Collagen alpha-2(VI)	Col6a2	16	18	13	14	18	19	0.6304	0.0500	33.0	110	0.30
Decorin	Dcn	15	12	13	30	31	25	0.0023	0.0109	41.7	40	1.04
Dermatopontin	Dpt	0	0	0	5	6	3	0.0083	0.0185	4.8	20	0.24
Dystroglycan	Dag1	2	2	0	6	9	6	0.0117	0.0217	8.5	97	0.09
Fibromodulin	Fmod	2	2	5	15	12	8	0.0241	0.0272	14.7	43	0.34
Fibronectin	Fn1	0	2	2	36	39	19	0.0094	0.0207	32.6	270	0.12
Galectin-1	Lgals1	14	13	14	19	19	18	0.0008	0.0043	32.5	15	2.16
Galectin-3	Lgals3	0	2	0	13	17	20	0.0012	0.0065	17.3	27	0.64
Galectin-3 binding protein	Galectin-3-BP	0	0	0	4	3	5	0.0013	0.0076	4.1	67	0.06
Hyaluronan and proteoglycan link protein 1	Hapln1	7	7	6	0	0	3	0.0050	0.0163	7.7	40	0.19
Laminin, alpha 1	Lama1	0	0	0	7	9	3	0.0270	0.0283	6.5	338	0.02
Laminin, alpha 2	Lama2	13	12	7	17	28	16	0.0863	0.0402	31.2	281	0.11
Laminin, alpha 4	Lama4	0	0	0	9	7	2	0.0470	0.0348	6.2	196	0.03
Laminin, alpha 5	Lama5	0	0	0	4	8	5	0.0119	0.0228	5.8	404	0.01
Laminin, beta 1	Lamb1	3	4	2	9	16	6	0.0685	0.0380	13.3	203	0.07
Laminin, gamma 1	Lamc1	14	14	17	29	35	30	0.0011	0.0054	46.4	177	0.26
Lumican	Lum	7	7	7	20	24	17	0.0040	0.0141	27.2	38	0.72
Microfibrillar associated protein 5	MFAP5	0	0	0	4	4	0	0.1162	0.0413	2.8	19	0.15
Mimecan/Osteoglycin	Ogn	11	13	11	26	27	19	0.0128	0.0239	35.9	34	1.06
Neurocan	Ncan	3	8	6	9	2	15	0.4788	0.0478	14.4	136	0.11
Nidogen-1	Nid1	17	13	17	38	43	26	0.0210	0.0261	51.4	137	0.38
Nidogen-2	Nid2	3	4	2	9	23	9	0.0858	0.0391	16.8	153	0.11
Osteonectin	SPARC	0	0	0	4	4	4	0.0000	0.0011	4.1	34	0.12
Osteopontin	Spp1	0	0	0	0	0	3	0.3739	0.0467	1.0	35	0.03
Periostin	Postn	0	0	0	20	30	6	0.0551	0.0359	18.7	90	0.21
Perlecan (Hspg2)	Hspg2	6	8	11	38	48	34	0.0020	0.0106	48.3	230	0.21
Prolargin	Prelp	14	16	14	34	44	30	0.0073	0.0174	51.3	43	1.19
Tenascin-R	Tnr	36	36	29	27	13	24	0.0615	0.0370	54.4	149	0.36
Transforming growth factor-beta-induced protein ig-h3	Tgfbi	8	10	4	24	27	13	0.0406	0.0315	28.6	75	0.38
Versican	Vcan	18	23	23	20	14	20	0.2096	0.0446	39.0	300	0.13
Vitronectin	Vtn	0	0	0	4	5	4	0.0005	0.0033	4.4	55	0.08

Supplementary Table S2: ECM proteins identified by proteomics.
47 extracellular matrix proteins identified by proteomics in 3 intact (Control) and 3 injured T10 (injury epicentre) spinal cord segments, 8 weeks post contusion. Protein levels were estimated by spectral counting. Statistical analysis was performed using t-test and p values were corrected using the Benjamini-Hochberg false discovery rate estimation procedure (q) . Identified proteins are sorted alphabetically. "0" indicates no identified protein spectra.

Supplementary Table S3

A

Gene Name	Protein Name	Uninjured1	Uninjured2	Uninjured3	Injured1	Injured2	Injured3	Fold change Injured / Uninjured	t-test p value	Corrected p value q=0.05
ARPC1B	Actin related protein 2/3 complex, subunit 1B	111	176	211	450	553	614	3.2	0.0027	0.015
ANXA1	Annexin A1	83	163	178	402	403	411	2.9	0.0009	0.009
ANXA2	Annexin A2	357	576	621	950	1139	1050	2.0	0.0058	0.021
APOE	Apolipoprotein E	3140	3330	3321	6814	7092	6206	2.1	0.0002	0.004
B2M	Beta-2-microglobulin	965	1013	1029	1549	1482	1737	1.6	0.0017	0.014
BGN	Biglycan	312	483	542	693	807	741	1.7	0.0169	0.039
CALR	Calreticulin	1030	980	932	1122	1157	1212	1.2	0.0090	0.029
CTSB	Cathepsin B	964	959	1141	2297	2340	2279	2.3	0.0000	0.001
CTSD	Cathepsin D	851	722	989	3150	3616	2460	3.6	0.0030	0.016
CTSH	Cathepsin H	225	288	305	538	511	673	2.1	0.0056	0.020
CD9	CD9 antigen	1006	1112	1292	1591	2128	1731	1.6	0.0199	0.042
CP	Ceruloplasmin	19	41	44	262	320	264	8.2	0.0003	0.006
CLU	Clusterin	2615	2770	3080	4173	5552	4782	1.7	0.0088	0.026
COL1A1	Collagen alpha-1(I)	303	550	813	1277	1691	1349	2.6	0.0105	0.031
COL15A1	Collagen alpha-1(XV)	44	62	84	109	152	133	2.1	0.0167	0.038
COL1A2	Collagen alpha-2(I)	293	509	632	866	1042	903	2.0	0.0151	0.036
CST3	Cystatin C	3666	3698	3484	4371	3925	4246	1.2	0.0191	0.040
DMD	Dystrophin	51	55	46	75	68	81	1.5	0.0060	0.022
ERP29	Endoplasmic reticulum protein 29	350	374	453	602	648	495	1.5	0.0260	0.045
EEF1A1	Eukaryotic translation elongation factor 1 alpha 1	2093	2335	2294	2643	2861	3108	1.3	0.0149	0.035
EIF5	Eukaryotic translation initiation factor 5	65	64	66	99	92	92	1.4	0.0003	0.005
EZR	Ezrin	157	165	192	289	284	293	1.7	0.0004	0.007
FN1	Fibronectin	27	66	128	218	370	273	3.9	0.0162	0.037
LGALS3	Galectin-3	193	312	272	2046	1989	2382	8.3	0.0001	0.003
LGALS3BP	Galectin-3 binding protein	182	207	251	592	555	465	2.5	0.0016	0.013
GJA1	Gap junction protein, alpha 1	191	202	297	313	369	391	1.6	0.0349	0.049
GFAP	Glial fibrillary acidic protein	1544	1397	1717	2611	3009	3096	1.9	0.0015	0.012
GRN	Granulins	283	291	368	1215	1088	1103	3.6	0.0001	0.002
HMGB1	High mobility group box 1	43	37	42	47	45	50	1.2	0.0398	0.051
ITGAM	Integrin, alpha M (CD11b)	39	35	36	87	77	93	2.4	0.0005	0.008
LAMA1	Laminin, alpha 1	16	15	17	30	26	26	1.7	0.0009	0.010
LAMB2	Laminin, beta 2	109	116	147	183	250	185	1.7	0.0311	0.048
LAMC1	Laminin, gamma 1	349	318	341	424	446	398	1.3	0.0067	0.024
LAMP1	Lysosomal-associated membrane protein 1	294	310	282	388	430	360	1.3	0.0115	0.033
MSN	Moesin	92	112	114	159	213	186	1.8	0.0097	0.030
MYH1	Myosin, heavy chain 1	272	310	253	383	365	366	1.3	0.0063	0.023
P4HB	Prolyl 4-hydroxylase, beta polypeptide	253	347	275	382	446	421	1.4	0.0210	0.043
RPL10	Ribosomal protein L10	1556	1467	1757	2166	2310	2019	1.4	0.0089	0.028
RPL10A	Ribosomal protein L10A	815	855	1014	1236	1276	1060	1.3	0.0302	0.047
RPL12	Ribosomal protein L12	842	766	1094	1277	1477	1138	1.4	0.0470	0.052
RPL15	Ribosomal protein L15	833	870	876	1198	1503	1247	1.5	0.0088	0.027
RPL18	Ribosomal protein L18	790	802	893	1083	1253	1064	1.4	0.0111	0.032
RPS6	Ribosomal protein S6	732	769	864	980	1044	1096	1.3	0.0082	0.025
SSR4	Signal sequence receptor, delta	324	319	428	504	562	476	1.4	0.0226	0.044
SLC3A2	Solute carrier family 3, member 2	460	417	579	627	732	643	1.4	0.0360	0.050
TLN1	Talin 1	422	457	539	689	751	708	1.5	0.0035	0.017
TPM4	Tropomyosin 4	264	244	359	515	571	496	1.8	0.0048	0.018
VIM	Vimentin	638	1001	1258	2075	2790	2527	2.6	0.0055	0.019
**TNC*	*Tenascin C*	*40*	*42*	*36*	*65*	*64*	*58*	*1.6*	*0.0015*	*0.011*
**CD14*	*Cluster of differentiation 14*	*83*	*107*	*79*	*125*	*145*	*156*	*1.6*	*0.0147*	*0.034*
**TLR4*	*Toll-like receptor 4*	*47*	*51*	*41*	*80*	*58*	*71*	*1.5*	*0.0278*	*0.046*
**MD1*	*Myeloid differentiation factor 1*	*121*	*137*	*128*	*172*	*148*	*160*	*1.2*	*0.0194*	*0.041*

Supplementary Table S3

B

Gene Name	Protein Name	Uninjured1	Uninjured2	Uninjured3	Injured1	Injured2	Injured3	Fold change Injured / Uninjured	t-test p value	Corrected p value q=0.05
ARPC1B	Actin related protein 2/3 complex, subunit 1B	0	0	0	9	7	6	7.5	0.0015	0.016
ANXA1	Annexin A1	0	0	0	23	21	20	21.1	0.0000	0.004
ANXA2	Annexin A2	12	13	16	26	34	24	2.1	0.0121	0.031
APOE	Apolipoprotein E	4	0	9	31	32	29	6.4	0.0007	0.010
B2M	Beta-2-microglobulin	2	2	0	4	3	4	2.3	0.0285	0.045
BGN	Biglycan	16	13	14	33	32	26	2.0	0.0031	0.020
CALR	Calreticulin	15	18	18	26	21	25	1.4	0.0155	0.035
CTSB	Cathepsin B	5	7	4	12	16	14	2.6	0.0024	0.019
CTSD	Cathepsin D	6	8	5	14	13	18	2.4	0.0065	0.024
CTSH	Cathepsin H	0	0	0	2	2	2	2.0	0.0000	0.002
CD9	CD9 antigen	4	5	4	5	6	7	1.4	0.0350	0.048
CP	Ceruloplasmin	3	4	3	27	27	27	8.1	0.0000	0.001
CLU	Clusterin	6	7	4	12	13	12	2.2	0.0013	0.015
COL1A1	Collagen alpha-1(I)	18	14	12	28	40	28	2.1	0.0204	0.038
COL15A1	Collagen alpha-1(XV)	3	3	3	5	9	6	2.2	0.0380	0.049
COL1A2	Collagen alpha-2(I)	9	8	9	17	19	18	2.1	0.0002	0.006
CST3	Cystatin C	4	5	5	7	6	7	1.5	0.0069	0.026
DMD	Dystrophin	2	0	0	6	5	6	4.3	0.0022	0.018
ERP29	Endoplasmic reticulum protein 29	6	5	5	9	9	7	1.6	0.0255	0.041
EEF1A1	Eukaryotic translation elongation factor 1 alpha 1	29	31	33	44	39	42	1.3	0.0044	0.022
EIF5	Eukaryotic translation initiation factor 5	4	4	2	5	5	5	1.5	0.0498	0.050
EZR	Ezrin	5	10	9	26	28	21	3.1	0.0034	0.021
FN1	Fibronectin	0	2	2	36	39	19	18.8	0.0094	0.027
LGALS3	Galectin-3	0	2	0	13	17	20	12.7	0.0012	0.013
LGALS3BP	Galectin-3 binding protein	0	0	0	4	3	5	4.1	0.0013	0.014
GJA1	Gap junction protein, alpha 1	0	2	2	9	5	13	5.5	0.0266	0.042
GFAP	Glial fibrillary acidic protein	149	147	191	269	226	289	1.6	0.0137	0.033
GRN	Granulins	0	0	0	4	3	3	3.4	0.0007	0.009
HMGB1	High mobility group box 1	6	6	7	11	14	9	1.8	0.0289	0.046
ITGAM	Integrin, alpha M (CD11b)	0	0	0	5	5	2	4.1	0.0188	0.036
LAMA1	Laminin, alpha 1	0	0	0	7	9	3	6.5	0.0270	0.044
LAMB2	Laminin, beta 2	12	11	8	26	34	22	2.6	0.0112	0.030
LAMC1	Laminin, gamma 1	14	14	17	29	35	30	2.1	0.0011	0.011
LAMP1	Lysosomal-associated membrane protein 1	0	0	0	6	7	7	6.8	0.0000	0.005
MSN	Moesin	15	19	18	42	44	38	2.4	0.0003	0.007
MYH1	Myosin, heavy chain 1	0	0	0	23	39	26	29.1	0.0045	0.023
P4HB	Prolyl 4-hydroxylase, beta polypeptide	15	16	16	25	30	27	1.7	0.0016	0.017
RPL10	Ribosomal protein L10	6	7	5	9	7	9	1.4	0.0267	0.043
RPL10A	Ribosomal protein L10A	4	5	2	6	7	7	1.9	0.0240	0.040
RPL12	Ribosomal protein L12	0	2	3	5	6	6	2.9	0.0132	0.032
RPL15	Ribosomal protein L15	3	3	5	6	6	7	1.7	0.0227	0.039
RPL18	Ribosomal protein L18	5	5	4	10	7	8	1.8	0.0150	0.034
RPS6	Ribosomal protein S6	4	5	4	8	6	6	1.6	0.0303	0.047
SSR4	Signal sequence receptor, delta	0	0	0	3	3	3	3.1	0.0000	0.003
SLC3A2	Solute carrier family 3, member 2	8	9	9	14	12	15	1.6	0.0096	0.028
TLN1	Talin 1	9	13	13	46	68	43	4.4	0.0066	0.025
TPM4	Tropomyosin 4	14	13	11	20	22	18	1.6	0.0099	0.029
VIM	Vimentin	52	54	58	155	178	145	2.9	0.0004	0.008

Supplementary Table 3A-B: Molecules differentially regulated both at the mRNA and protein level.
48 genes were upregulated in the injury epicentre both at the transcript (**A**) and protein (**B**) level. mRNA was quantified by microarray gene expression profiling (study **E-GEOD-2599**, ArrayExpress, EBI) 5 weeks post spinal contusion (**A**). This study was preformed by Aimone and colleagues (see reference 28 in Manuscript). Protein levels were estimated by proteomics using spectral counting, 8 weeks post spinal contusion (**B**). Statistical analysis was performed using t-test and p values were corrected using the Benjamini-Hochberg false discovery rate estimation procedure (q) Identified transcripts (**A**) and proteins (**B**) are sorted alphabetically. "0" indicates no identified protein spectra.

** TNC, CD14, TLR4 and MD1 microarray gene expression profiles (from **E-GEOD-2599**) were added on the list of regulated transcripts although they were not identified by proteomics.*
